# Thioether-enabled palladium-catalyzed atroposelective C–H olefination for N–C and C–C axial chirality†

**DOI:** 10.1039/d2sc00748g

**Published:** 2022-03-10

**Authors:** Yanjun Li, Yan-Cheng Liou, Xinran Chen, Lutz Ackermann

**Affiliations:** Institut für Organische und Biomolekulare Chemie, Georg-August-Universität Göttingen Tammannstraße 2 37077 Göttingen Germany Lutz.Ackermann@chemie.uni-goettingen.de; Department of Chemistry, Zhejiang University Hangzhou 310027 China

## Abstract

Thioethers allowed for highly atroposelective C–H olefinations by a palladium/chiral phosphoric acid catalytic system under ambient air. Both N–C and C–C axial chiral (hetero)biaryls were successfully constructed, leading to a broad range of axially chiral *N*-aryl indoles and biaryls with excellent enantioselectivities up to 99% ee. Experimental and computational studies were conducted to unravel the walking mode for the atroposelective C–H olefination. A plausible chiral induction model for the enantioselectivity-determining step was established by detailed DFT calculations.

## Abstract 2

Thioethers allowed for highly atroposelective C–H olefinations by a palladium/chiral phosphoric acid catalytic system under ambient air.

## Graphical abstract

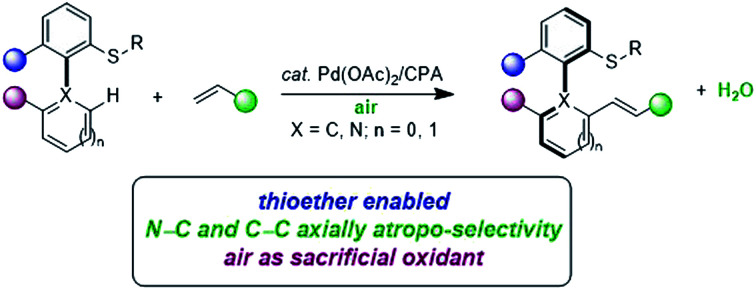

## Introduction

**Scheme 1:**
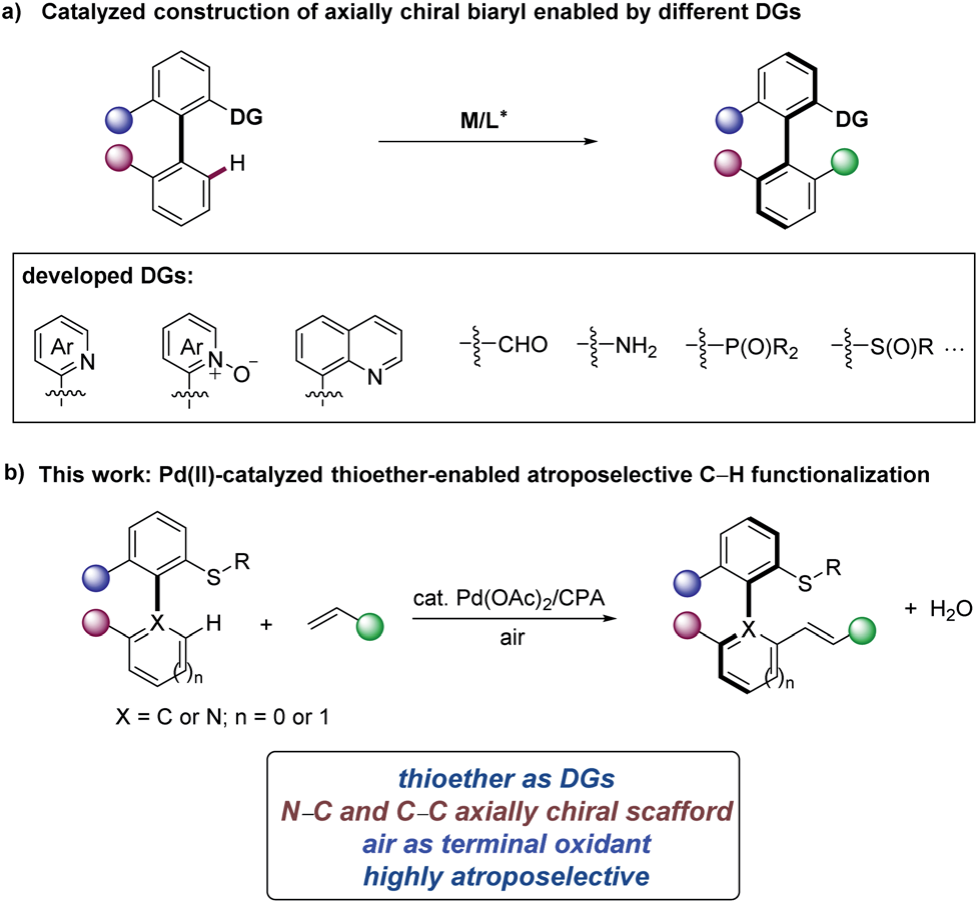
Atroposelective C–H activation for axial chirality.

Axially chiral compounds are ubiquitous structural motifs in biologically active natural products,^1^ privileged catalysts,^2^ chiral ligands^3^ and material sciences.^4^ In recent years, transition-metal-catalyzed asymmetric C–H activation^5^ has become an efficient and powerful synthesis platform to construct diverse axial chirality.^6,7^ Atroposelective *ortho*-C–H functionalization of (hetero)biaryl precursors is one of the attractive approaches to diversified chiral (hetero)biaryls.^8^ Based on this approach, numerous directing groups (DGs) have been identified to provide the required steric congestion and reactivity (Scheme 1a).^9–15^ For example, isoquinolines and pyridines were employed in rhodium-catalyzed C–H functionalization for axially chiral biaryl compounds synthesis by Murai,^9^ You,^10*a*^ and Lassaletta.^11^ Pyridine *N*-oxides were applied to palladium-catalyzed asymmetric C–H iodination by You.^10*b*^ Chiral sulfoxides as DGs were elegantly utilized for diastereoselective C–H activation by Wencel-Delord/Colobert.^12^ In contrast, phosphine-based DGs enabled palladium-catalyzed C–H olefinations to prepare chiral phosphineolefin compounds.^13^ The Shi group found free amines^14*a*,*b*^ and quinolines^14*c*^ as efficient DGs for synthesizing axially chiral biaryl compounds *via* palladium/chiral phosphoric acid (CPA) catalytic system. Likewise, Shi group developed the atroposelective C–H functionalizations of biaryl aldehydes to prepare axially chiral aldehydes through chiral transient directing groups (cTDGs) strategy.^15^ Despite these significant advances in the synthesis of axially chiral compounds, the exploration of other DGs and catalytic systems to expand more structurally diverse axially chiral biaryls continue to be in high demand.

N–C axial chirality is the key element of atropisomeric natural products and chiral catalysts,^16,17^ and unexplored compared with C–C axial chirality and remains a major challenge.^18^ This is largely due to the increased distance between the *ortho*-substituents next to the N–C chiral axis, leading to relatively low rotational barrier and atropostability.^18*d*^ Recent representative contributions for the construction of N–C axially chiral scaffolds hail from the Wencel-Delord/Colobert,^19^ Xie,^20^ Shi,^21^ among others.^22^ Asymmetric C–H functionalization of *N*-aryl heterocycles or *N*-aryl amides is a useful synthetic strategy to construct N–C axial chirality.^20,21,22*a*,*g*^ However, this strategy was thus far unfortunately restricted to the use of superstoichiometric amounts of cost-intensive silver salts, jeopardizing the inherent atom-economy of the C–H activation strategy.

Recently, aryl alkyl thioethers have been reported as DGs for C–H functionalization.^23^ However, the use of thioethers as DGs in asymmetric C–H activation has remained elusive.^24^ Within our program on sustainable C–H activation,^25^ we have now unravelled a thioether-directed strategy for the enantioselective synthesis of N–C and C–C axially chiral molecules with air as the oxidant, thereby only giving H_2_O as the sole byproduct (Scheme 1b). Salient features of our findings include (a) thioether-directed atroposelective C–H functionalization, (b) construction of N–C axially chiral scaffolds in the absence of toxic oxidants, and (c) key mechanistic insights into the mode of enantio-induction by DFT calculations.

## Results and discussion

**Scheme 2:**
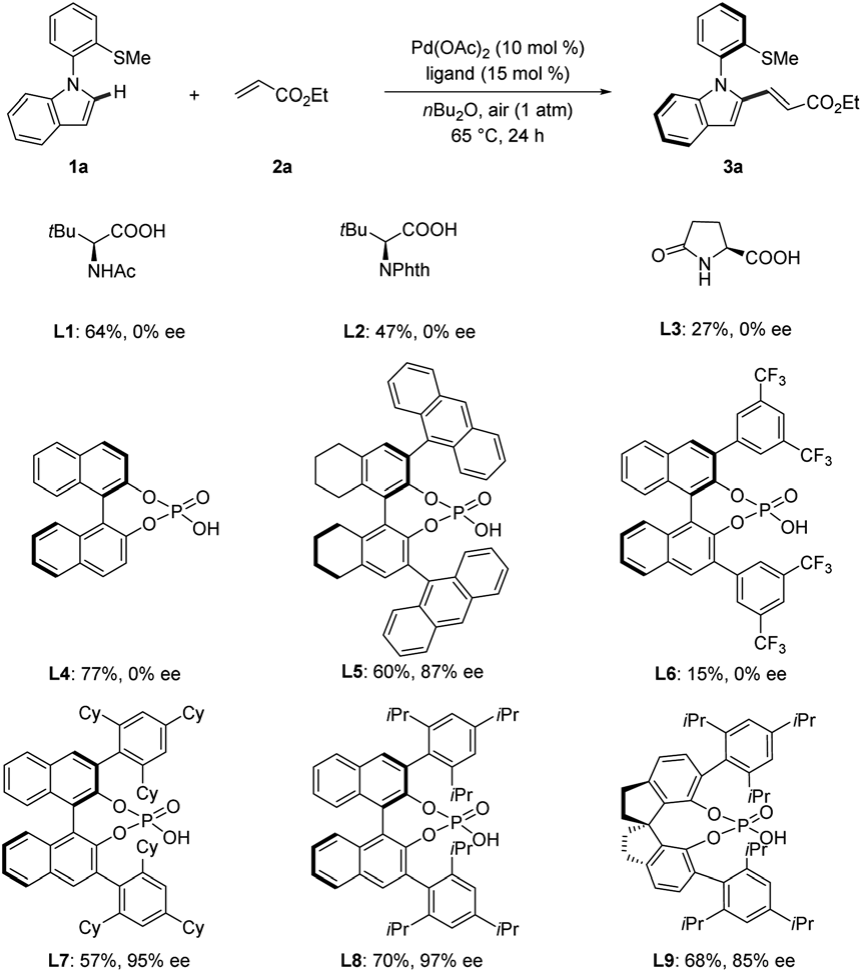
Optimization of the N–C atroposelective C–H olefination. Reaction conditions: 1a (0.10 mmol), 2a (0.30 mmol), Pd(OAc)_2_ (10 mol%), ligand (15 mol%), *n*Bu_2_O (2.0 mL), 65 °C, under air (1 atm). Yield was determined by ^1^H NMR. The ee value was determined by HPLC analysis.

We first chose *N*-arylindoles 1a bearing a thiomethyl group as the model substrate for the synthesis of N–C axially chiral motifs (Schemes 2 and S1 in the ESI†). We selected Pd(OAc)_2_ as the catalyst and air as the oxidant to test various chiral acids. Several *N*-protected amino acids were first probed in the presence of ethyl acrylate (2a) in *n*Bu_2_O at 65 °C for 24 h. L1–L3 afforded product 3a in moderate yield albeit without enantioselectivity control. Next, simple chiral phosphoric acid (CPA, L4) was examined but no enantioinduction was detected. To our delight, when H8-Binol CPA L5 bearing 9-anthracenyl substituents was examined, product 3a was obtained in 60% yield with 87% ee. Further optimization of CPAs L6–L9 indicated that L8 was superior, leading to excellent enantiocontrol of 97% ee. Overall, the optimized reaction conditions were viable with ligand L8 under air in *n*Bu_2_O at 65 °C.

**Scheme 3:**
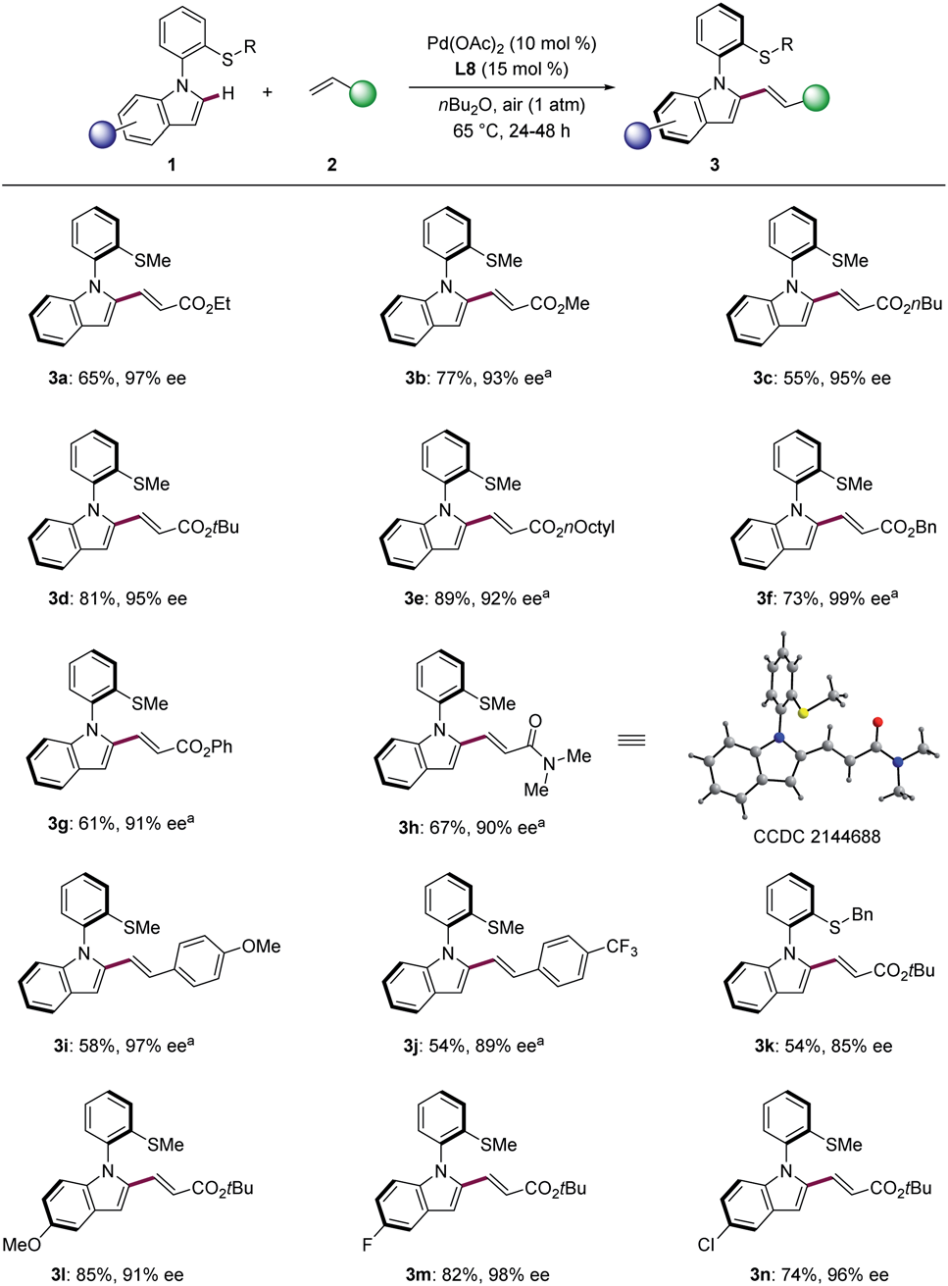
N–C atroposelective C–H olefination of *N*-aryl indoles. ^*a*^ Under oxygen atmosphere.

With the optimized reaction conditions in hand, we next explored the generality of the palladium-catalyzed N–C atroposelective olefination (Scheme 3). A broad range of alkenes 2 provided the desired products 3a–3n with excellent enantioselectivity up to 99% ee. Acrylates with different groups were well compatible with the catalytic system (Scheme 3, 3a–3g). Specially, benzyl acrylate (2f) afforded the desired olefinated products 3f with 99% ee. Acrylamide provided the desired olefinated product heterobiaryl 3h in 67% yield with 90% ee. The absolute configuration of 3h was unanimously assigned by single-crystal X-ray diffraction analysis (CCDC 2144688†), featuring a *R* configuration. Styrenes were also suitable partners for this transformation under 1 atm of oxygen. The reaction of 4-methoxystyrene proceeded to give the olefinated products 3i in 58% yield with 97% ee. Electron-deficient CF_3_-substituted styrene delivered product 3j with slightly reduced enantiocontrol of 89% ee. Next, a variety of *N*-arylindoles 1k–1n were tested. Thioether DG bearing benzyl substituent was well compatible with this transformation (3k). Indole 1l with an electron-donating methoxy group provided the corresponding olefinated product 3l with 91% ee. The fluoro- and chloro-substituted indoles (1m and 1n) were likewise tolerated in the N–C atropo-selective alkenylation with excellent enantioselectivity (3m, 98% ee; 3n, 96% ee).

**Scheme 4:**
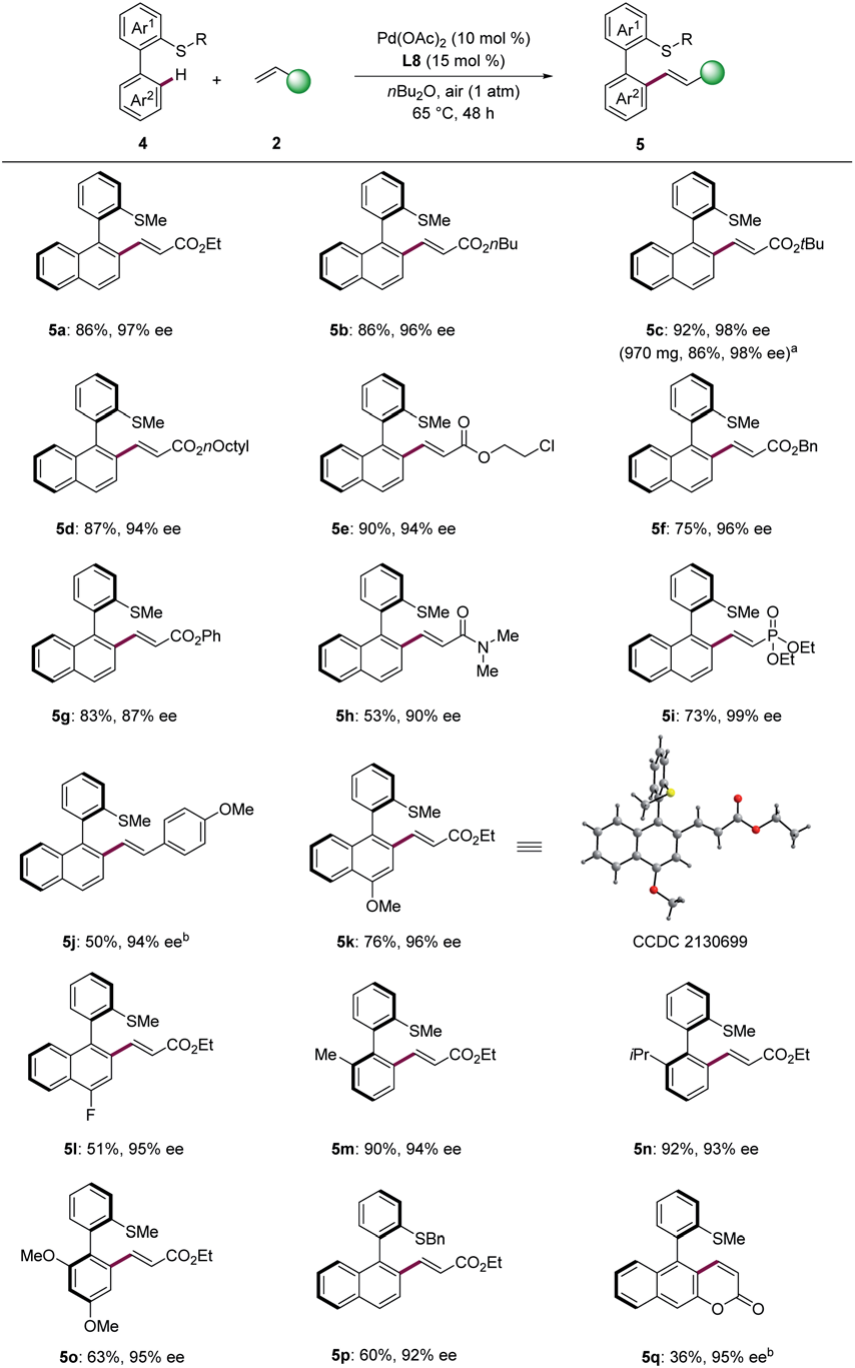
C–C atroposelective C–H olefination of biaryls. ^*a*^ Pd(OAc)_2_ (7.5 mol%), L8 (12 mol%). ^*b*^ Under oxygen atmosphere.

To further illustrate the diversity of the thioether-directed atroposelective C–H activation,^26^ the construction of C–C axial chirality was next explored (Scheme 4). Various acrylates were well-tolerated with high enantiocontrol (5a–5g, 87–98% ee). When the aerobic olefination was performed on gram scale, the product 5c was obtained in 86% yield and 98% ee. Acrylamide 2h provided the desired olefinated product 5h in 53% yield with 90% ee. In addition, the olefination with vinylphosphonate proceeded efficiently to give the product 5i with excellent enantiocontrol (99% ee). High enantioselectivity (5j, 94% ee) was obtained when 4-methoxystyrene was employed as the olefination reagent. Next, we investigated the scope of biaryl thioethers. Biaryls with substituents on the naphthalene were well tolerated, giving the desired products (5k, 96% ee; 5l, 95% ee). The absolute configuration of compound 5k was assigned by single-crystal X-ray diffraction analysis (CCDC 2130699†), featuring a *R* configuration. In addition, substituted biaryls proved also feasible with enantioselectivity and furnished the corresponding products 5m–5o with high ee. Substrates containing methoxy at the *ortho*- and *para*-position likewise gave the desired product 5o in 63% yield with 95% ee. Thioether DGs bearing benzyl substituents was found compatible (5p, 60% yield, 92% ee). Interestingly, substrate with an acrylate substituent provided the desired intramolecular olefinated product 5q with the coumarin scaffold in good enantioselectivity. This approach set the stage for the synthesis of coumarin scaffolds with axial chirality.

**Scheme 5:**
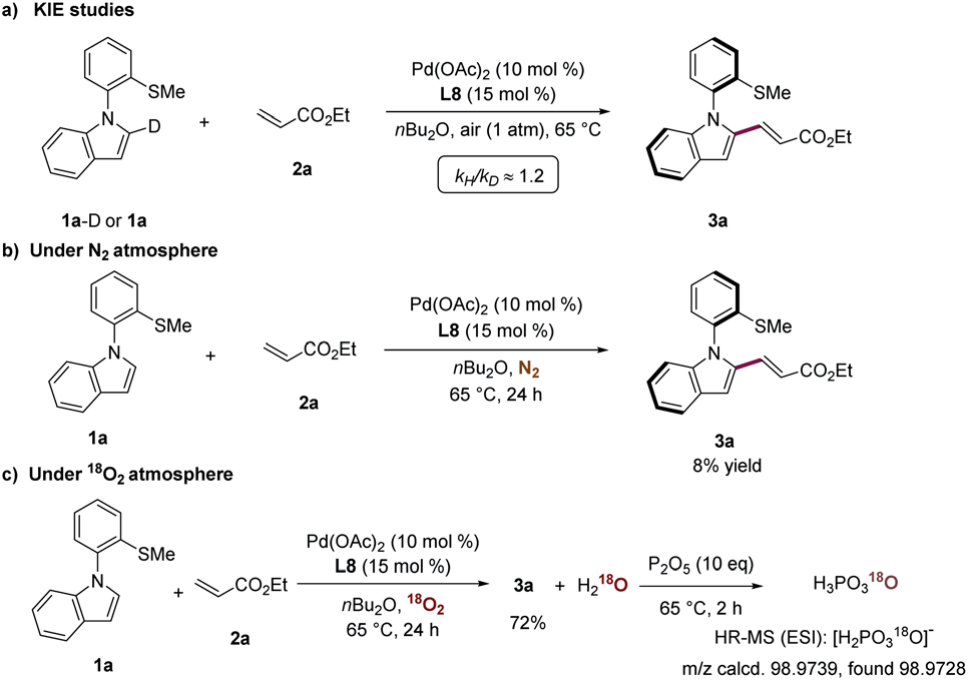
Key mechanistic findings.

In order to shed light on the operative catalysis of this atroposelective C–H olefination, the kinetic isotope effect (KIE) experiment was performed by parallel reactions of substrates 1a-D and 1a with 2a (Scheme 5a). The KIE of *k*_H_/*k*_D_ ≈ 1.2 was indicative of C–H activation not being the rate-determining step. Next, the reaction of substrates 1a and 2a under a nitrogen atmosphere provided product 3a with a low yield of <10%, highlighting that catalytic turnover did not occur (Scheme 5b). The reaction under an atmosphere of isotopically-labeled ^18^O_2_ atmosphere led to the selective formation of H_2_^18^O, which was trapped by P_2_O_5_ to afford the ^18^O-containing phosphoric acid (Scheme 5c). These control experiments clearly showed that oxygen in the air was the oxidant for this aerobic transformation.

**Fig. 1:**
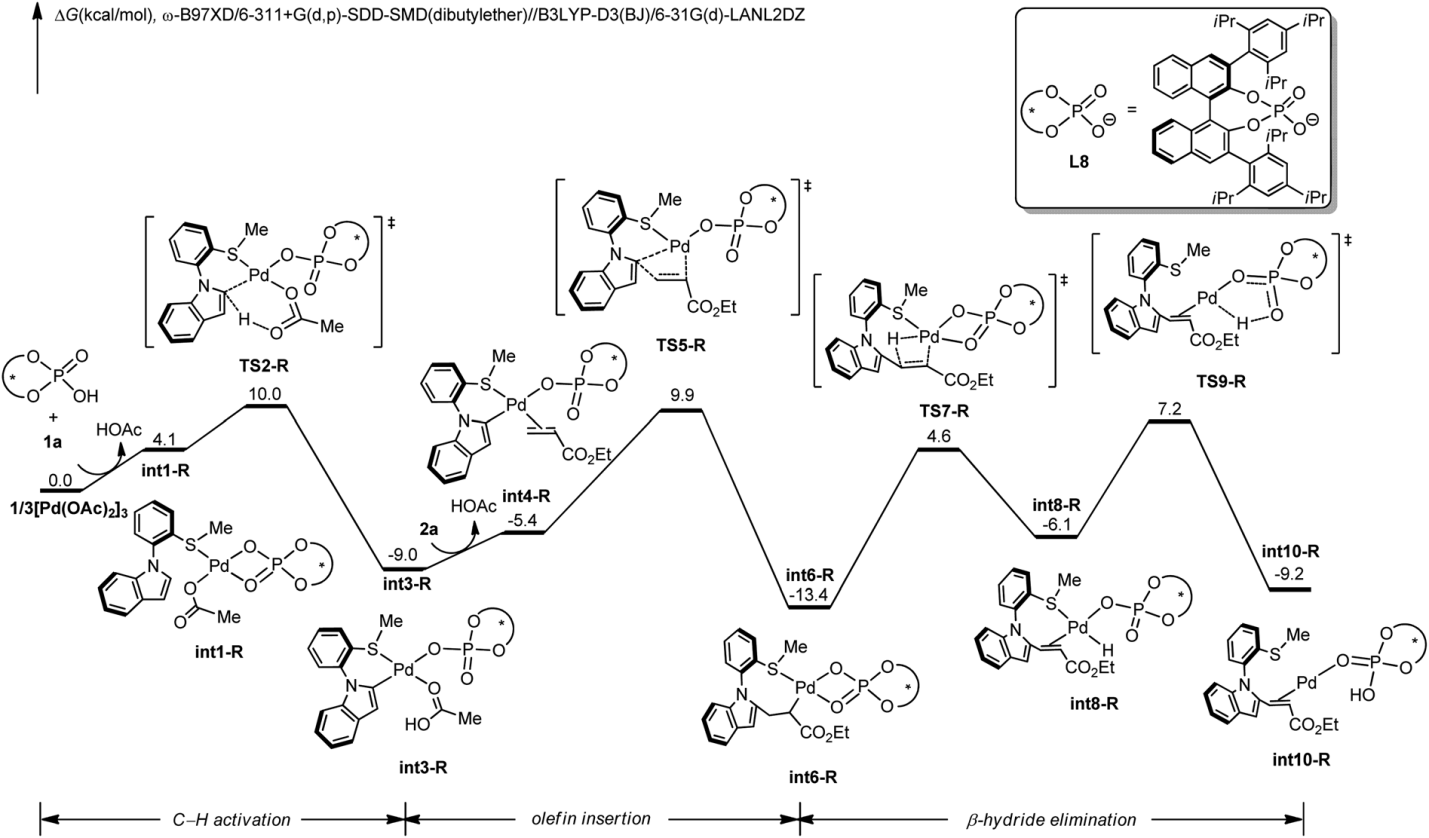
DFT-computed free energy profile of palladium-catalyzed N–C atroposelective C–H alkenylation for *R*-enantiomer. Computational methods: ω-B97XD/6-311+G(d,p)-SDD-SMD(dibutylether)//B3LYP-D3(BJ)/6-31G(d)-LANL2DZ.

**Fig. 2:**
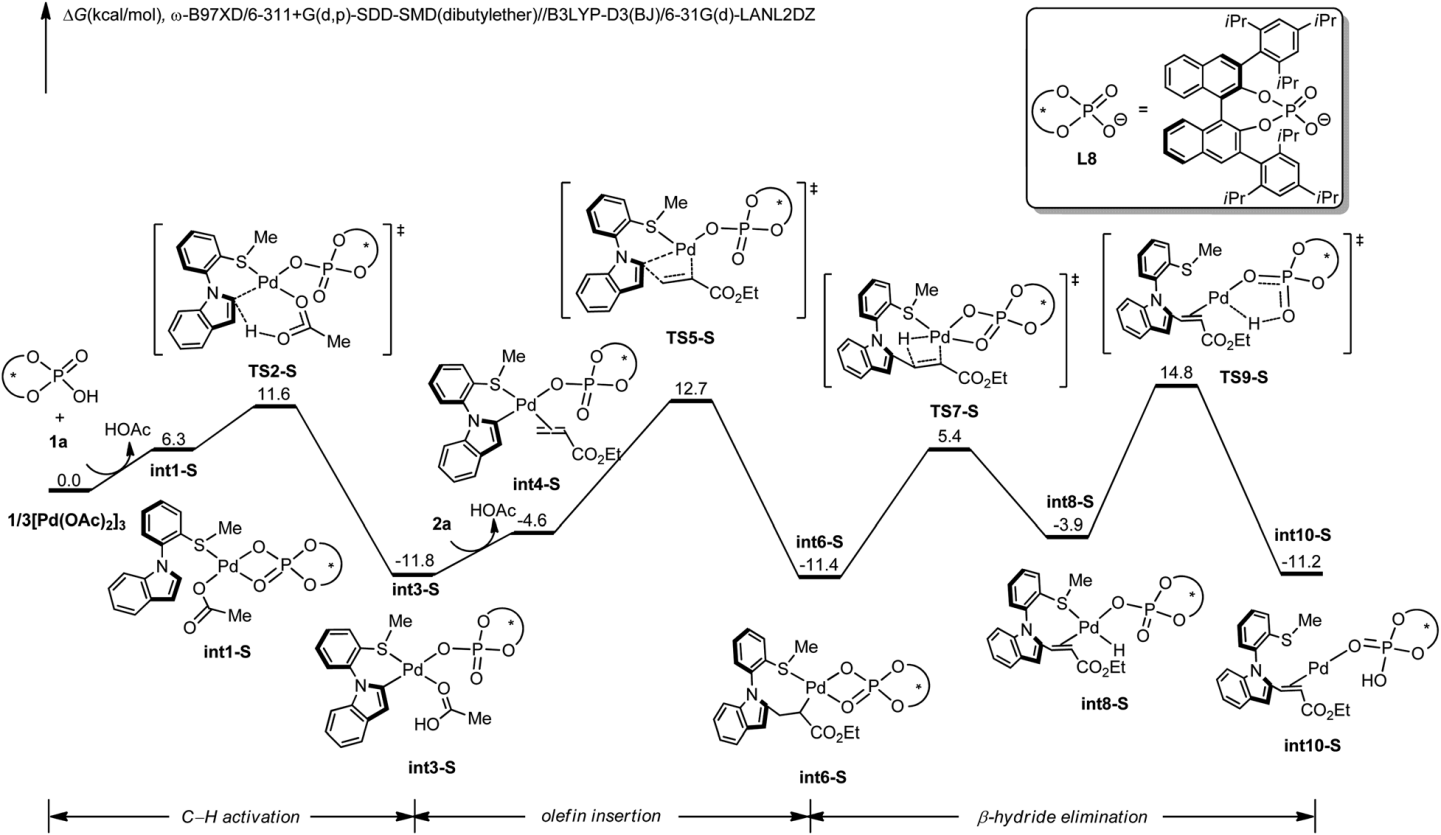
DFT-computed free energy profile of palladium-catalyzed N–C atroposelective C–H alkenylation for *S*-enantiomer. Computational methods: ω-B97XD/6-311+G(d,p)-SDD-SMD(dibutylether)//B3LYP-D3(BJ)/6-31G(d)-LANL2DZ.

To gain insights into the N–C atroposelective C–H alkenylation, the reaction mechanism was probed by means of DFT calculations.^27^ Free energy changes for the generation of *R*-configuration and *S*-configuration product are depicted in Fig. 1 and 2. Starting from the complexation of L8 to Pd(OAc)_2_ and the coordination of 1a, intermediate int1-R is formed. Subsequently, int1-R undergoes a facile C–H activation step to form the arylpalladium species int3-R. This relatively low C–H activation barrier is consistent with the experimental KIE of 1.2 (Scheme 5a). The alkene coordination and the following insertion through TS5-R generates the alkylpalladium intermediate int6-R. Then, int6-R undergoes a β-hydride elimination to form a palladium hydride species int8-R, leading to the reduced palladium(0) complex int10-R with product coordination. Based on the most favorable pathway for the palladium-catalyzed N–C atroposelective C–H alkenylation, the rate-determining step is the β-hydride elimination, with a barrier of 21.6 kcal mol^−1^. As for the *S*-enantiomer, the initial C–H activation step is also facile. However, the olefin insertion step requires a barrier of 24.5 kcal mol^−1^, which is significantly higher than the barrier for the *R*-enantiomer. Likewise, we also confirmed that the racemization of the axial chirality of the alkylpalladium intermediate int6-R is not feasible after the olefin insertion step (Fig. S3 in ESI†). Thereby, the olefin insertion is identified as the enantioselectivity-determining step.

**Fig. 3:**
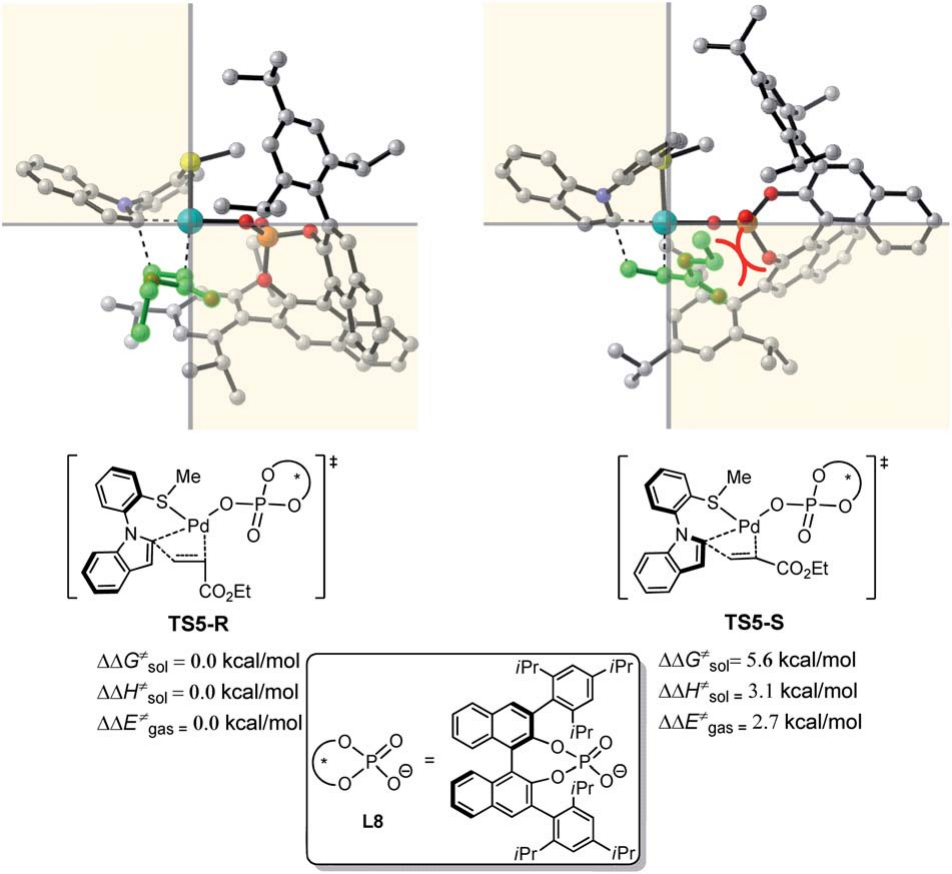
DFT computed transition states involved in the enantioselectivity-determining step (olefin migratory insertion). In the transition state structures, non-participating hydrogens are removed for clarity.

To reveal the exact origins of enantioselectivity, we directed our attention to the nature of the migratory insertion transition states. Fig. 3 displays the optimized structures and relative free energies of the two competing enantioselectivity-determining transition states TS5-R and TS5-S. Thus, when comparing the two competitive transition states, the sterically demanding L8 occupies the first and fourth quadrants. The substrate 1a is in similar positions in the two transition states (second quadrant). The olefin 2a (highlighted in green), however, is positioned in different quadrants. In the favoured transition state TS5-R, the ester group is in the third quadrant, which is distant from the bulky L8. In the disfavoured transition state TS5-S, the same ester group is positioned in the fourth quadrant, which leads to steric repulsions with the sterically congested ligand L8, being responsible for the destabilization of such transition state.

**Scheme 6:**
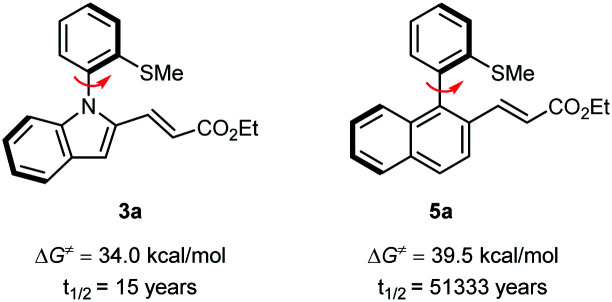
Calculated rotational barriers for the racemization of products 3a and 5a and the corresponding half-life at 65 °C.

To evaluate the atropostability of the N–C axially chiral and the C–C axially chiral compounds, the rotational barriers and half-lifes for racemization of 3a and 5a were determined as depicted in Scheme 6. The results suggest that the C–C axially chiral compound 5a is more atropostable than N–C axially chiral compound 3a.

**Scheme 7:**
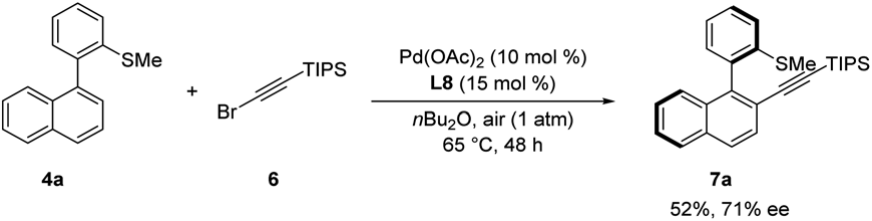
Atroposelective palladium-catalyzed C–H alkynylation.

Encouraged by our results, we wondered whether the thioether-directed palladium-catalyzed atroposelective C–H functionalization might enable C–H alkynylation to prepare chiral molecules containing an alkynyl moiety. To our delight, and otherwise identical reaction conditions as the atroposelective C–H olefination, the reaction of biaryl substrate 4a and TIPS protected alkynyl bromide 6 afforded product 7a in 52% yield with moderate enantioselectivity (71% ee, Scheme 7).

## Conclusion

In summary, we have reported on thioether-enabled atroposelective C–H olefination *via* a palladium/chiral phosphoric acid catalytic system. Both N–C and C–C axial chiralities were successfully established, leading to a broad range of axially chiral *N*-aryl indoles and biaryls with excellent enantioselectivities up to 99% ee. Notably, the catalytic system used air as the terminal oxidant instead of environmentally-unfriendly and expensive silver salts. Experimental and computational studies were conducted to illuminate the mechanism, which involves C–H activation, olefin insertion, and β-hydride elimination. The chiral induction mode of the enantioselectivity-determining step was identified by detailed DFT calculations.

## Data availability

All experimental data, procedures for data analysis and pertinent data sets are provided in the ESI.†

## Author contributions

Y. L. and L. A. conceived the project. Y. L. and Y.-C. L. performed the experiments, analyzed and interpreted the experimental data. X. C. performed DFT calculations. Y. L. and L. A. wrote the manuscript. All of the authors discussed the results and contributed to the preparation of the final manuscript.

## Conflicts of interest

There are no conflicts to declare.

## Supplementary Material

SC-013-D2SC00748G-s001

SC-013-D2SC00748G-s002
