# Supplementary material for: Thioether-enabled palladium-catalyzed atroposelective C–H olefination for N–C and C–C axial chirality
Source: Chem Sci. 2022 Mar 10;13(14):4088–94. doi: 10.1039/d2sc00748g (PMC8985512; doi:10.1039/d2sc00748g)
Supplement: SC-013-D2SC00748G-s001 [file SC-013-D2SC00748G-s001.pdf]

Electronic Supplementary Material (ESI) for Chemical Science.
This journal is © The Royal Society of Chemistry 2022

Supporting Information

Thioether-Enabled Palladium-Catalyzed Atroposelective C–H Olefination for N−C and C−C Axial Chirality

Yanjun Li,‡[a] Yan-Cheng Liou,‡[a] Xinran Chen‡[ab] and Lutz Ackermann*[a]

[a]Institut für Organische und Biomolekulare Chemie
Georg-August-Universität
Tammannstraße 2, 37077 Göttingen, Germany
[b]Department of Chemistry, Zhejiang University
Hangzhou, 310027, China

* Fax: 0551-39-66777; Email: Lutz.Ackermann@chemie.uni-goettingen.de

‡These authors contributed equally to this work.

Table of Contents

1. General Remarks S3

2. Synthesis of Substrates S4

3. Optimization of the Reaction Conditions.. S8

4. Substrate Scope for Atroposelective C–H Olefination................................. S9

5. Atroposelective Palladium-Catalyzed C−H Alkynylation. S51

6. X-Ray Analysis S54

7. Key Mechanistic Findings S59

8. Computational Methods S63

9. Reference.. S158

10. NMR-Spectra.. S159

1. General Remarks

Catalytic reactions were carried out in Schlenk tubes under air or O_2 atmosphere using pre-dried glassware. Substrates **4a**, **4l**, **4m**, **4n** and **4p** were prepared by previously reported methods.[1] Other chemicals were obtained from commercial sources and were used without further purification. Yields refer to isolated compounds, estimated to be >95% pure as determined by ^{1}H-NMR. TLC: Macherey-Nagel, TLC plates Alugram®Sil G/UV254. Detection under UV light at 254 nm. Chromatography: Separations were carried out on Merck Silica 60 (0.040–0.063 mm, 70–230 mesh ASTM). All IR spectra were recorded on a BRUKER ALPHA-P spectrometer. ESI-MS: Finnigan LCQ. High resolution mass spectrometry (HRMS): APEX IV 7T FTICR, Bruker Daltonic. HPLC chromatograms were recorded on an Agilent 1290 Infinity using CHIRALPAK® IB-3, IC-3, IF-3 and AD-3 columns (3.0 µm particle size; Ø: 4.6 mm and 250 mm length). Optical rotations were measured with Perkin Elmer 343 polarimeter at the stated temperature under a Na/Hg lamp, λ = 589 nm (c in g/100 ml). ^{1}H, ^{13}C, ^{19}F and ^{31}P NMR-spectra were recorded at 400 MHz (^{1}H), 101 MHz [^{13}C, APT (Attached Proton Test)], 377 MHz (19F) and 162 MHz (^{31}P) respectively, on Varian Bruker Avance III 400, Bruker Avance III HD 400 instruments in $CDCl_3$. If not otherwise specified, chemical shifts (δ) are given in ppm

2. Synthesis of Substrates

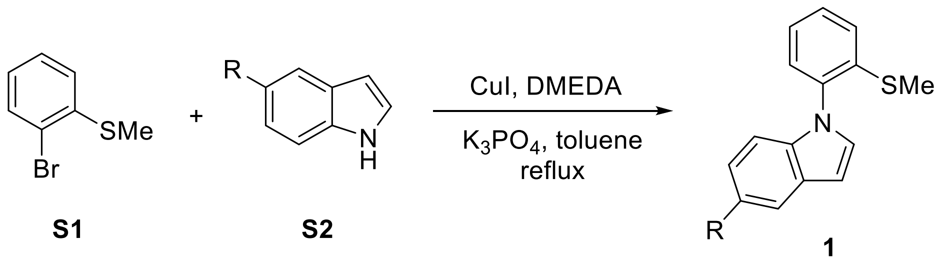

S1 **S2** **1**

A 50 ml round bottom flask was charged with **S1** (5.0 mmol), **S2** (5.0 mmol), CuI (10 mol%), DMEDA (20 mol%), K_3PO_4 (13.0 mmol) in toluene (15 mL). The reaction mixture was stirred at reflux for 24 h. After cooling to room temperature and filter over a silica pad washing with ethyl acetate. Organic layer was concentrated, and the residue was purified by silica gel column chromatography.

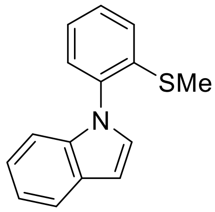

1-(2-(Methylthio)phenyl)-1*H*-indole (1a)

^{1}H NMR (400 MHz, $CDCl_3$): δ = 7.75 – 7.69 (m, 1H), 7.46 (ddd, *J* = 8.5, 7.1, 1.6 Hz, 1H), 7.42 – 7.34 (m, 2H), 7.31 (dd, *J* = 7.2, 1.5 Hz, 1H), 7.29 – 7.25 (m, 1H), 7.24 – 7.16 (m, 2H), 7.15 – 7.10 (m, 1H), 6.73 (dd, *J* = 3.2, 0.9 Hz, 1H), 2.31 (s, 3H). ^{13}C NMR (101 MHz, $CDCl_3$): δ = 138.0 (C_q), 136.7 (C_q), 136.7 (C_q), 128.7 (CH), 128.7 (CH), 128.5 (C_q), 128.3 (CH), 126.2 (CH), 125.3 (CH), 122.1 (CH), 120.9 (CH), 120.1 (CH), 110.5 (CH), 102.8 (CH), 15.1 (CH_3). MS (ESI) *m*/*z* (relative intensity): 240 (100) $[M + H]^+$. HR-MS (ESI): *m*/*z* calcd. for $[C_{15}H_{13}NS + H]^+$ 240.0841, found 240.0841.

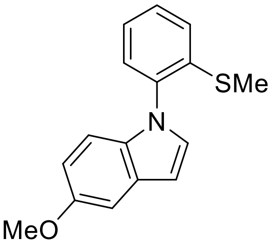

5-Methoxy-1-(2-(methylthio)phenyl)-1H-indole (1l)

^{1}H NMR (400 MHz, $CDCl_3$): δ = 7.49 – 7.27 (m, 4H), 7.24 (d, *J* = 3.2 Hz, 1H), 7.18 (d, *J* = 2.5 Hz, 1H), 7.02 (d, *J* = 8.9 Hz, 1H), 6.87 (dd, *J* = 8.9, 2.5 Hz, 1H), 6.65 (d, *J* = 3.2 Hz, 1H), 3.89 (s, 3H), 2.31 (s, 3H). ^{13}C NMR (101 MHz, $CDCl_3$): δ = 154.5 (C_q), 137.9 (C_q), 136.8 (C_q), 132.0 (C_q), 129.2 (CH), 128.7 (C_q), 128.6 (CH), 128.3 (CH),

126.2 (CH), 125.3 (CH), 112.2 (CH), 111.3 (CH), 102.5 (CH), 102.5 (CH), 55.8 (CH_3), 15.1 (CH_3). MS (ESI) *m*/*z* (relative intensity): 270 (100) $[M + H]^+$. HR-MS (ESI): *m*/*z* calcd. for $[C_{16}H_{15}NOS + H]^+$ 270.0947, found 270.0943.

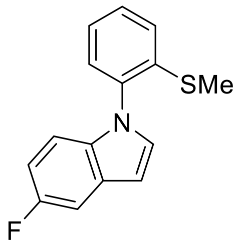

5-Fluoro-1-(2-(methylthio)phenyl)-1*H*-indole (1m)

^{1}H NMR (400 MHz, $CDCl_3$): δ = 7.59 (dd, *J* = 8.6, 5.3 Hz, 1H), 7.46 (ddd, *J* = 8.1, 6.8, 1.9 Hz, 1H), 7.38 (dd, *J* = 8.0, 1.4 Hz, 1H), 7.34 – 7.27 (m, 2H), 7.21 (d, *J* = 3.3 Hz, 1H), 6.92 (ddd, *J* = 9.5, 8.6, 2.3 Hz, 1H), 6.77 (dd, *J* = 9.8, 2.3 Hz, 1H), 6.67 (dd, *J* = 3.2, 0.9 Hz, 1H). ^{13}C NMR (101 MHz, $CDCl_3$): δ = 161.3 (C_q), 158.9 (C_q), 138.0 (C_q), 136.8 (C_q, d, *J* = 12.3 Hz), 136.2 (C_q), 129.2 (d, *J* = 3.7 Hz), 128.3 (CH), 126.2 (CH), 125.4 (CH), 124.7 (C_q), 121.6 (CH, d, *J* = 10.0 Hz), 108.9 (CH, d, *J* = 24.6 Hz), 102.9 (CH), 97.0 (CH, d, *J* = 26.6 Hz), 15.0 (CH_3). ^{19}F NMR (377 MHz, $CDCl_3$): δ = -120.5 (td, *J* = 9.6, 5.3 Hz). MS (ESI) *m*/*z* (relative intensity): 258 (100) $[M + H]^+$. HR-MS (ESI): *m*/*z* calcd. for $[C_{15}H_{12}FNS + H]^+$ 258.0747, found 258.0751.

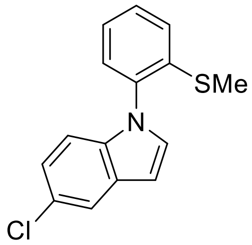

5-Chloro-1-(2-(methylthio)phenyl)-1*H*-indole (1n)

^{1}H NMR (400 MHz, $CDCl_3$): δ = 7.69 (d, *J* = 2.0 Hz, 1H), 7.48 (ddd, *J* = 8.0, 6.7, 2.1 Hz, 1H), 7.40 (dd, *J* = 8.0, 1.4 Hz, 1H), 7.35 – 7.30 (m, 2H), 7.28 (d, *J* = 3.2 Hz, 1H), 7.16 (dd, *J* = 8.7, 2.0 Hz, 1H), 7.07 – 7.00 (m, 1H), 6.67 (dd, *J* = 3.2, 0.9 Hz, 1H), 2.32 (s, 3H). ^{13}C NMR (101 MHz, $CDCl_3$): δ = 137.9 (C_q), 136.1 (C_q), 135.1 (C_q), 130.0 (CH), 129.3 (C_q), 129.0 (CH), 128.3 (CH), 126.2 (CH), 125.8 (C_q), 125.4 (CH), 122.3 (CH), 120.2 (CH), 111.6 (CH), 102.4 (CH), 15.0 (CH_3). MS (ESI) *m*/*z* (relative intensity): 274 (100) $[M + H]^+$. HR-MS (ESI): *m*/*z* calcd. for $[C_{15}H_{12}ClNS + H]^+$ 274.0452, found 274.0451.

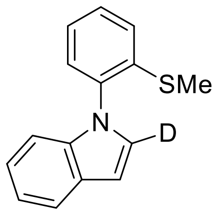

1-(2-(methylthio)phenyl)-1*H*-indole-2-*d* (1a-D)

^{1}H NMR (400 MHz, $CDCl_3$): δ = 7.79 – 7.71 (m, 1H), 7.48 (ddd, J = 8.6, 7.1, 1.6 Hz, 1H), 7.44 – 7.36 (m, 2H), 7.35 – 7.27 (m, 1H), 7.25 – 7.21 (m, 2H), 7.18 – 7.13 (m, 1H), 6.76 (d, J = 0.9 Hz, 1H), 2.32 (s, 3H). ^{13}C NMR (101 MHz, $CDCl_3$): δ = 137.9 (C_q), 136.6 (C_q), 136.6 (C_q), 128.7 (CH), 128.4 (CH), 128.3 (C_q), 126.2 (CH), 125.3 (CH), 122.0 (CH), 120.8 (CH), 120.1 (CH), 110.5 (CH), 102.8 (CH), 102.6 (CH), 15.0 (CH_3). MS (ESI) *m*/*z* (relative intensity): 241 (100) $[M + H]^+$, 263 (60) $[M + Na]^+$. HR-MS (ESI): *m*/*z* calcd. for $[C_{15}H_{12}DNS + H]^+$ 241.0904, found 241.0904.

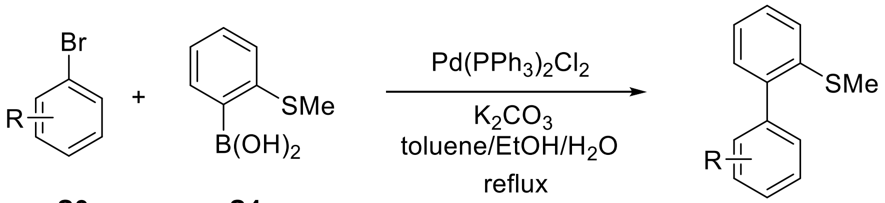

S3 **S4** **4**

A 100 ml round bottom flask was charged with **S3** (5.0 mmol), **S4** (7.0 mmol), $Pd(PPh_3)_2Cl_2$ (5 mol%), K_2CO_3 (25.0 mmol) in toluene (10 mL), EtOH (5 mL), H_2O (5 mL). The reaction mixture was stirred at reflux for 12 h. After cooling to room temperature, the reaction mixture was diluted with H_2O (10 mL) and extracted with EtOAc (3 × 10 mL). Organic layer was dried over Na_2SO_4, filtered, concentrated, and the residue was purified by silica gel column chromatography.

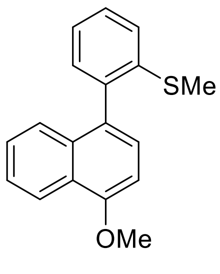

(2-(4-Methoxynaphthalen-1-yl)phenyl)(methyl)sulfane (4k)

^{1}H NMR (400 MHz, $CDCl_3$): δ = 8.36 (dt, J = 8.5, 1.2 Hz, 1H), 7.52 – 7.40 (m, 4H), 7.36 – 7.31 (m, 2H), 7.28 – 7.26 (m, 2H), 6.91 (d, J = 7.9 Hz, 1H), 4.07 (s, 3H), 2.33 (s, 3H). ^{13}C NMR (101 MHz, $CDCl_3$): δ = 155.3 (C_q), 139.2 (C_q), 138.9 (C_q), 132.7 (C_q), 131.1 (CH), 130.4 (C_q), 128.0 (CH), 127.2 (CH), 126.5 (CH), 125.7 (CH), 125.5 (C_q), 125.1 (CH), 124.4 (CH), 124.3 (CH), 122.2 (CH), 103.2 (CH), 55.5 (CH_3), 15.6

(CH_3). MS (ESI) *m*/*z* (relative intensity): 281 (100) $[M + H]^+$. HR-MS (ESI): *m*/*z* calcd. for $[C_{18}H_{16}OS + H]^+$ 281.0995, found 281.0998.

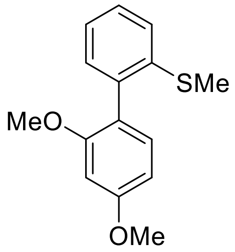

(2',4'-Dimethoxy-[1,1'-biphenyl]-2-yl)(methyl)sulfane (4o)

^{1}H NMR (400 MHz, $CDCl_3$): δ = 7.32 – 7.21 (m, 2H), 7.17 – 7.12 (m, 2H), 7.10 – 7.04 (m, 1H), 6.57 – 6.50 (m, 2H), 3.83 (s, 3H), 3.72 (s, 3H), 2.33 (s, 3H). ^{13}C NMR (101 MHz, $CDCl_3$): δ = 160.7 (C_q), 157.9 (C_q), 138.6 (C_q), 137.4 (C_q), 131.4 (CH), 130.6 (CH), 127.7 (CH), 125.1 (CH), 124.4 (CH), 122.1 (C_q), 104.1 (CH), 98.8 (CH), 55.6 (CH_3), 55.3 (CH_3), 16.0 (CH_3). MS (ESI) *m*/*z* (relative intensity): 261 (100) $[M + H]^+$. HR-MS (ESI): *m*/*z* calcd. for $[C_{15}H_{16}O_2S + H]^+$ 261.0944, found 261.0946.

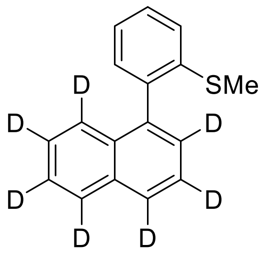

Methyl(2-(naphthalen-1-yl-*d*$_7$)phenyl)sulfane (4a-D$_7$)

^{1}H NMR (400 MHz, $CDCl_3$): δ = 7.52 – 7.43 (m, 1H), 7.41 – 7.36 (m, 1H), 7.33 – 7.28 (m, 2H), 2.34 (s, 3H). ^{13}C NMR (101 MHz, $CDCl_3$): δ = 138.8 (C_q), 138.7 (C_q), 138.0 (C_q), 133.4 (C_q), 131.7 (C_q), 130.6 (CH), 128.2 (CH), 127.9 (t), 127.5 (t), 126.8 (t), 125.5 (t), 125.0 (t), 124.6 (CH), 124.8 – 124.4 (m), 124.3, 15.6 (CH_3). MS (ESI) *m*/*z* (relative intensity): 258 (100) $[M + H]^+$. HR-MS (ESI): *m*/*z* calcd. for $[C_{17}H_7D_7S + H]^+$ 258.1328, found 258.1331.

3. Optimization of the Reaction Conditions

General Procedure: To an oven-dried 25 mL Schlenk tube was added substrate **1a** (0.10 mmol), **2a** (0.30 mmol), $Pd(OAc)_2$ (2.3 mg, 0.010 mmol), ligand (0.0150 mmol), n-Bu_2O (2.0 mL). The mixture was stirred for 24 h at 65 °C under air. Yield was determined by ^1H NMR. The ee value was determined by HPLC analysis.

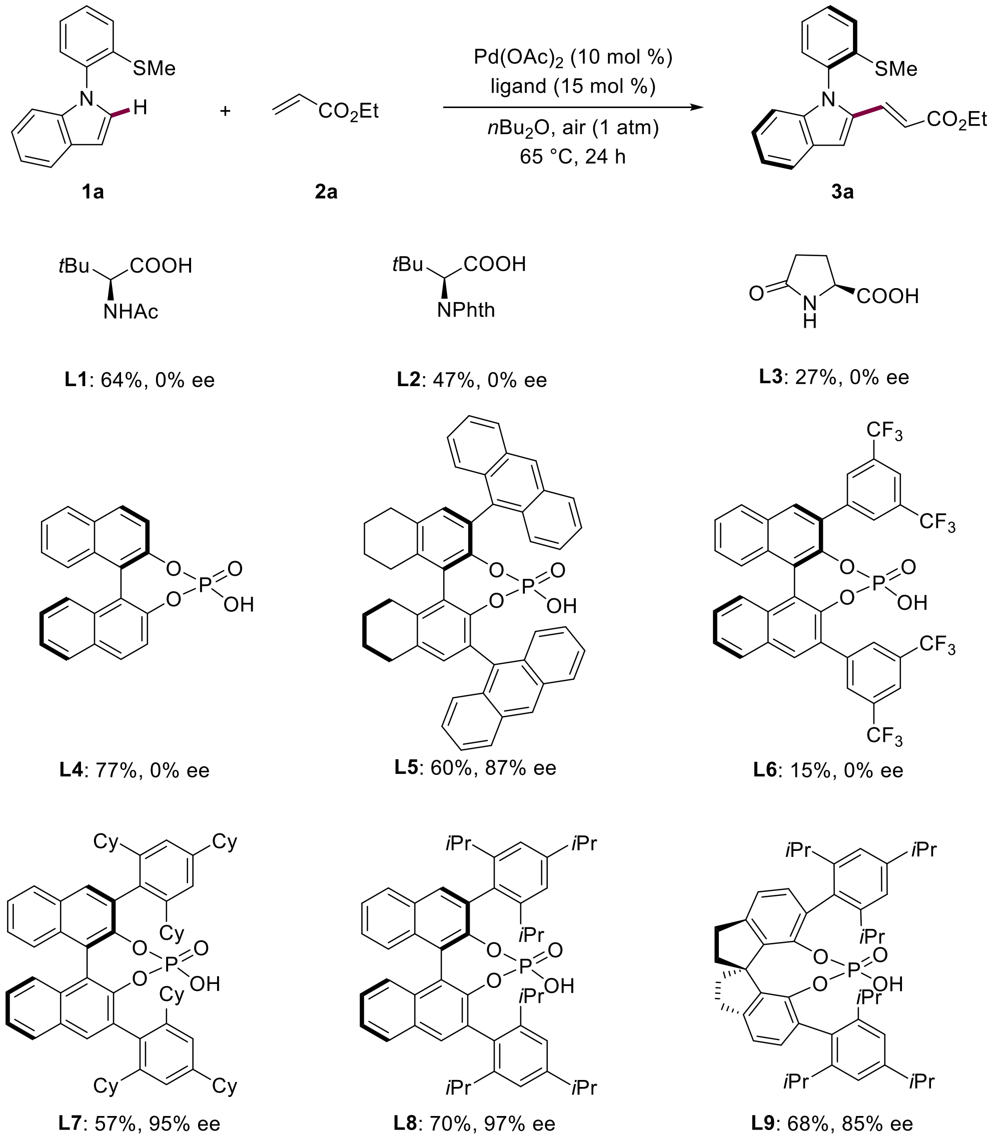

Scheme S1. Optimization of the N–C atroposelective C–H olefination. Reaction conditions: **1a** (0.10 mmol), **2a** (0.30 mmol), $Pd(OAc)_2$ (10 mol %), ligand (15 mol %), nBu_2O (2.0 mL), 65 °C, under air (1 atm).

4. Substrate Scope for Atroposelective C–H Olefination

General Procedure: To an oven-dried 25 mL Schlenk tube was added substrate **1** or **4** (0.10 mmol), **2** (0.30 mmol), $Pd(OAc)_2$ (2.3 mg, 0.010 mmol), **L8** (11.3 mg, 0.0150 mmol), *n*-Bu_2O (2.0 mL). The mixture was stirred for 24-48 h at 65 °C under air. The resulting mixture was purified by column chromatography on silica gel.

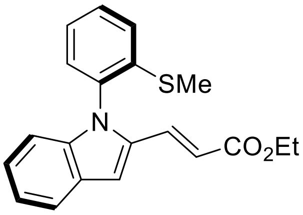

Ethyl (*E*)-3-(1-(2-(methylthio)phenyl)-1H-indol-2-yl)acrylate (3a)

The general procedure was followed using 1-(2-(methylthio)phenyl)-1*H*-indole (23.9 mg, 0.10 mmol), ethyl acrylate (32 µL, 0.30 mmol) at 65 °C for 24 h. Purification by column chromatography on silica gel (*n*-hexane/EtOAc: 15/1) yielded **3a** (22.0 mg, 65%) as a yellow oil. ^{1}H NMR (400 MHz, $CDCl_3$): δ = 7.72 – 7.65 (m, 1H), 7.53 (ddd, J = 8.0, 7.2, 1.7 Hz, 1H), 7.41 – 7.27 (m, 4H), 7.23 – 7.14 (m, 2H), 7.12 (s, 1H), 6.95 – 6.87 (m, 1H), 6.17 (d, J = 16.0 Hz, 1H), 4.18 (q, J = 7.1 Hz, 2H), 2.31 (s, 3H), 1.28 (t, J = 7.1 Hz, 3H). ^{13}C NMR (101 MHz, $CDCl_3$): δ = 167.0 (C_q), 140.0 (C_q), 139.3 (C_q), 135.2 (C_q), 133.9 (C_q), 133.2 (CH), 129.9 (CH), 129.9 (CH), 127.6 (C_q), 125.8 (CH), 125.5 (CH), 124.1 (CH), 121.3 (CH), 121.1 (CH), 117.6 (CH), 110.7 (CH), 105.6 (CH), 60.3 (CH_2), 14.6 (CH_3), 14.3 (CH_3). IR (ATR): 2959, 2922, 2855, 1707, 1628, 1475, 1268, 1173, 1141, 749 cm^{-1}. MS (ESI) *m/z* (relative intensity): 360 (100) $[M + Na]^+$, 338 (40) $[M + H]^+$. HR-MS (ESI): *m/z* calcd. for $[C_{20}H_{19}NO_2S + Na]^+$ 360.1029, found 360.1025. $[\alpha]_D^{20}$ = +20.5 (c = 0.2, $CHCl_3$). HPLC separation (Chiralpak® IB-3, *n*-hexane/*i*-PrOH 90:10, 1.0 mL/min, detection at 250 nm): t_r (major) = 6.3 min, t_r (minor) = 8.5 min, 97% ee.

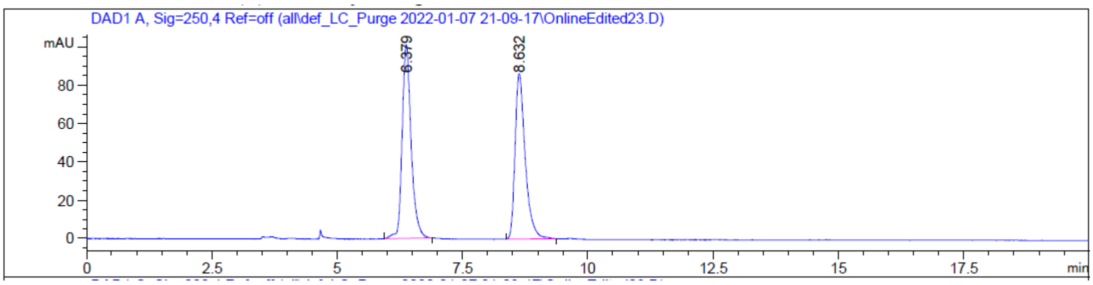

Peak #	RetTime [min]	Type	Width [min]	Area [mAU*s]	Height [mAU]	Area %
1	6.379	BB	0.1891	1265.03418	101.04815	51.0115
2	8.632	BB	0.2100	1214.86731	86.41243	48.9885

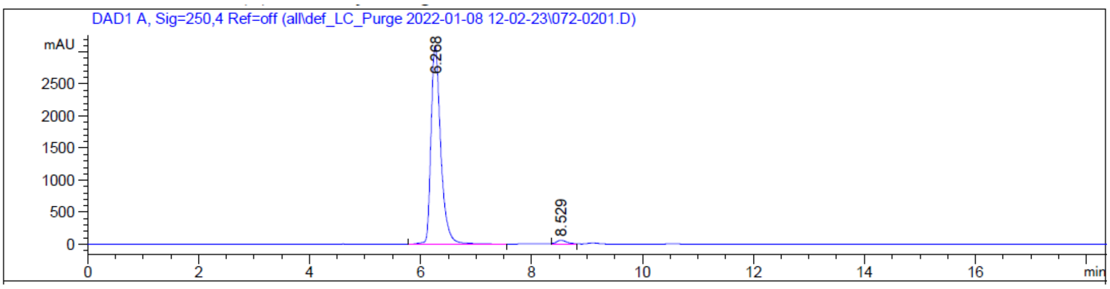

Peak #	RetTime [min]	Type	Width [min]	Area [mAU*s]	Height [mAU]	Area %
1	6.268	BB	0.1551	3.79587e4	3094.98706	98.2721
2	8.529	PM R	0.2007	667.40851	53.61182	1.7279

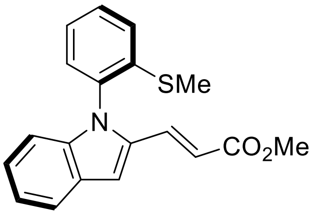

Methyl (*E*)-3-(1-(2-(methylthio)phenyl)-1H-indol-2-yl)acrylate (3b)

The general procedure was followed using 1-(2-(methylthio)phenyl)-1*H*-indole (23.9 mg, 0.10 mmol), methyl acrylate (28 µL, 0.30 mmol) under oxygen atmosphere (O_2 balloon) at 65 °C for 24 h. Purification by column chromatography on silica gel (*n*-hexane/EtOAc: 15/1) yielded **3b** (24.9 mg, 77%) as a yellow oil. ^{1}H NMR (400 MHz, $CDCl_3$): δ = 7.72 – 7.63 (m, 1H), 7.54 (ddd, J = 7.9, 7.2, 1.6 Hz, 1H), 7.41 – 7.31 (m, 3H), 7.31 – 7.23 (m, 1H), 7.23 – 7.14 (m, 2H), 7.12 (s, 1H), 6.90 (dd, J = 8.1, 1.2 Hz, 1H), 6.14 (d, J = 16.0 Hz, 1H), 3.72 (s, 3H), 2.31 (s, 3H). ^{13}C NMR (101 MHz, $CDCl_3$): δ = 167.4 (C_q), 140.0 (C_q), 139.4 (C_q), 135.0 (C_q), 133.9 (C_q), 133.5 (CH), 129.9 (CH), 129.9 (CH), 127.5 (C_q), 125.7 (CH), 125.5 (CH), 124.2 (CH), 121.4 (CH), 121.2 (CH), 117.0 (CH), 110.7 (CH), 105.9 (CH), 51.6 (CH_3), 14.6 (CH_3). IR (ATR): 2950, 2922, 1715, 1629, 1475, 1435, 1342, 1272, 1169, 971, 749 cm^{-1}. MS (ESI) *m*/*z* (relative intensity): 669 (100) [2M + Na]$^+$, 346 (56) [M + Na]$^+$. HR-MS (ESI): *m*/*z* calcd. for [$C_{19}H_{17}NO_2S$ + Na]$^+$ 346.0872, found 346.0867. $[\alpha]_D^{20}$ = +26.4 (c = 0.11, $CHCl_3$). HPLC separation (Chiralpak® IB-3, *n*-hexane/*i*-PrOH 90:10, 1.0 mL/min, detection at 250 nm): t_r (major) = 7.0 min, t_r (minor) = 10.2 min, 93% ee.

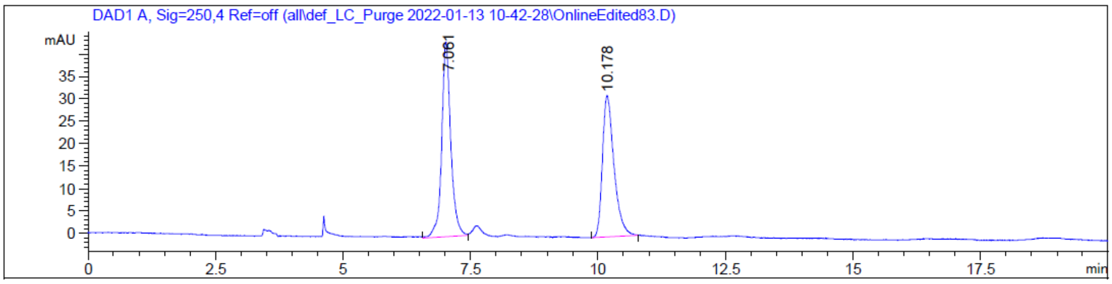

Peak #	RetTime [min]	Type	Width [min]	Area [mAU*s]	Height [mAU]	Area %
1	7.061	MM R	0.2909	537.40460	30.78758	51.9395
2	10.178	BB	0.2363	497.26877	31.47963	48.0605

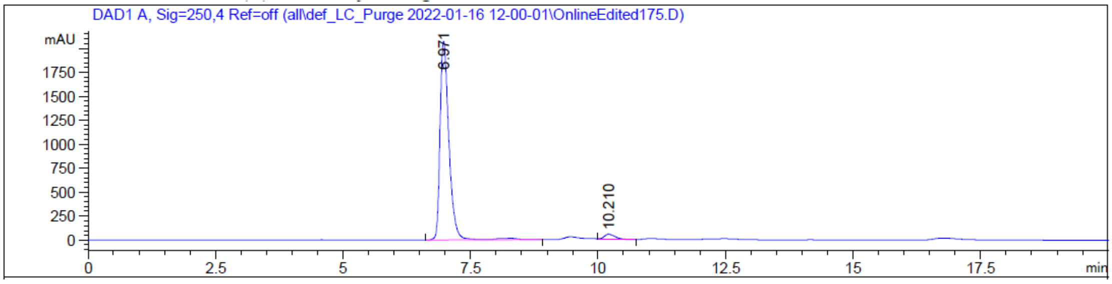

Peak #	RetTime [min]	Type	Width [min]	Area [mAU*s]	Height [mAU]	Area %
1	6.971	BV R	0.1801	2.53329e4	2072.72388	96.5394
2	10.210	VB	0.2362	908.08746	57.21055	3.4606

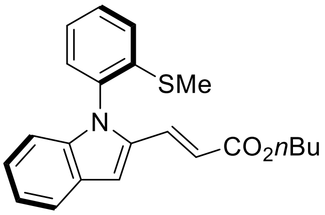

Butyl (*E*)-3-(1-(2-(methylthio)phenyl)-1H-indol-2-yl)acrylate (3c)

The general procedure was followed using 1-(2-(methylthio)phenyl)-1*H*-indole (23.9 mg, 0.10 mmol), *n*-butyl acrylate (45 µL, 0.30 mmol) at 65 °C for 24 h. Purification by column chromatography on silica gel (*n*-hexane/EtOAc: 15/1) yielded **3c** (20.1 mg, 55%) as a yellow oil. ^{1}H NMR (400 MHz, $CDCl_3$): δ = 7.68 (dd, J = 8.1, 1.3 Hz, 1H), 7.53 (ddd, J = 8.5, 7.4, 1.7 Hz, 1H), 7.41 – 7.24 (m, 4H), 7.18 (td, J = 7.4, 1.5 Hz, 2H), 7.12 (s, 1H), 6.94 – 6.87 (m, 1H), 6.19 (d, J = 16.0 Hz, 1H), 4.13 (t, J = 6.7 Hz, 2H), 2.30 (s, 3H), 1.63 (dq, J = 8.4, 6.7 Hz, 2H), 1.44 – 1.33 (m, 2H), 0.93 (t, J = 7.4 Hz,

3H). ^{13}C NMR (101 MHz, $CDCl_3$): δ = 167.1 (C_q), 140.0 (C_q), 139.3 (C_q), 135.2 (C_q), 133.9 (C_q), 133.2 (CH), 129.9 (CH), 129.9 (CH), 127.6 (C_q), 125.7 (CH), 125.5 (CH), 124.1 (CH), 121.3 (CH), 121.1 (CH), 117.6 (CH), 110.7 (CH), 105.4 (CH), 64.3 (CH_2), 30.7 (CH_2), 19.1 (CH_2), 14.6 (CH_3), 13.7 (CH_3). IR (ATR): 2959, 2922, 2867, 1709, 1629, 1476, 1275, 1260, 1168, 750 cm^{-1}. MS (ESI) *m*/*z* (relative intensity): 753 (100) $[2M + Na]^+$, 388 (99) $[M + Na]^+$, 366 (33) $[M + H]^+$. HR-MS (ESI): *m*/*z* calcd. for $[C_{22}H_{23}NO_2S + Na]^+$ 388.1342, found 388.1330. $[\alpha]^{20}_{D}$ = +20.8 (c = 0.12, $CHCl_3$). HPLC separation (Chiralpak® IB-3, *n*-hexane/*i*-PrOH 90:10, 1.0 mL/min, detection at 250 nm): t_r (major) = 6.0 min, t_r (minor) = 8.0 min, 95% ee.

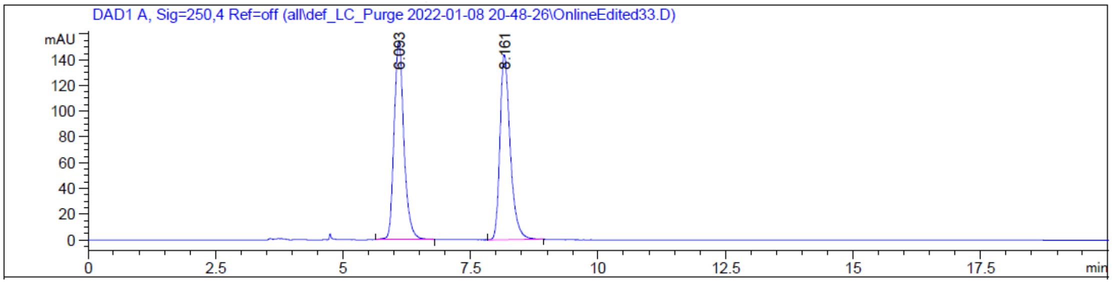

Peak #	RetTime [min]	Type	Width [min]	Area [mAU*s]	Height [mAU]	Area %
1	6.093	BB	0.2065	2068.11401	154.18657	50.6314
2	8.161	BB	0.2115	2016.53052	143.87329	49.3686

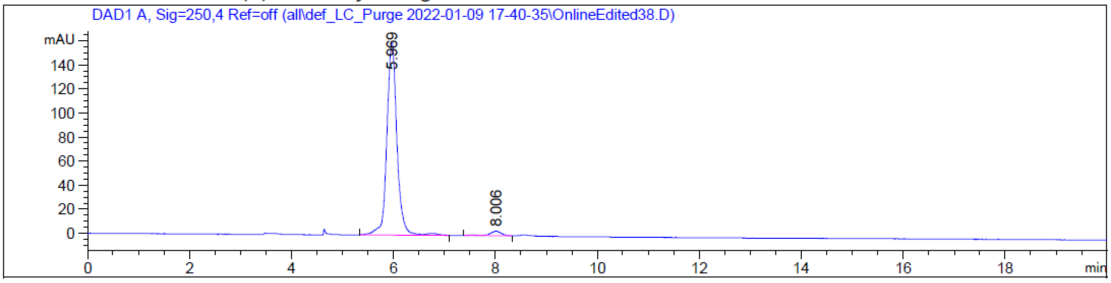

Peak #	RetTime [min]	Type	Width [min]	Area [mAU*s]	Height [mAU]	Area %
1	5.969	BV R	0.2073	2208.63403	161.23804	97.2800
2	8.006	VB R	0.1992	61.75480	3.74823	2.7200

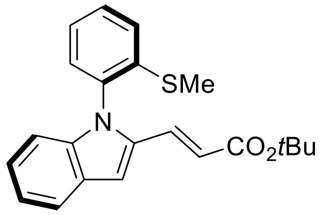

Tert-butyl (_E_)-3-(1-(2-(methylthio)phenyl)-1H-indol-2-yl)acrylate (3d)

The general procedure was followed using 1-(2-(methylthio)phenyl)-1*H*-indole (23.9 mg, 0.10 mmol), *tert*-butyl acrylate (44 µL, 0.30 mmol) at 65 °C for 24 h. Purification by column chromatography on silica gel (*n*-hexane/EtOAc: 15/1) yielded **3d** (29.6 mg, 81%) as a yellow oil. ^{1}H NMR (400 MHz, $CDCl_3$): δ = 7.70 – 7.64 (m, 1H), 7.52 (ddd, *J* = 7.9, 7.2, 1.7 Hz, 1H), 7.37 (dd, *J* = 8.0, 1.3 Hz, 1H), 7.34 – 7.29 (m, 1H), 7.28 – 7.21 (m, 2H), 7.21 – 7.14 (m, 2H), 7.11 (s, 1H), 6.92 – 6.87 (m, 1H), 6.21 (d, *J* = 15.9 Hz, 1H), 2.31 (s, 3H), 1.48 (s, 9H). ^{13}C NMR (101 MHz, $CDCl_3$): δ = 166.3 (C_q), 140.0 (C_q), 139.1 (C_q), 135.4 (C_q), 133.8 (C_q), 132.2 (CH), 129.9 (CH), 129.8 (CH), 127.6 (C_q), 125.7 (CH), 125.5 (CH), 123.9 (CH), 121.2 (CH), 121.0 (CH), 119.7 (CH), 110.7 (CH), 104.7 (CH), 80.3 (C_q), 28.1 (CH_3), 14.6 (CH_3). IR (ATR): 3057, 2957, 2923, 1702, 1628, 1476, 1351, 1139, 972, 749 cm^{-1}. MS (ESI) *m*/*z* (relative intensity): 388 (100) [M + Na]$^+$, 388 (60) [2M + Na]$^+$, 366 (33) [M + H]$^+$. HR-MS (ESI): *m*/*z* calcd. for $[C_{22}H_{23}NO_2S + Na]^+$ 388.1342, found 388.1336. $[\alpha]_D^{20}$ = +12.6 (c = 0.23, $CHCl_3$). HPLC separation (Chiralpak® IC-3, *n*-hexane/*i*-PrOH 97:3, 1.0 mL/min, detection at 250 nm): t_r (major) = 6.8 min, t_r (minor) = 7.5 min, 95% ee.

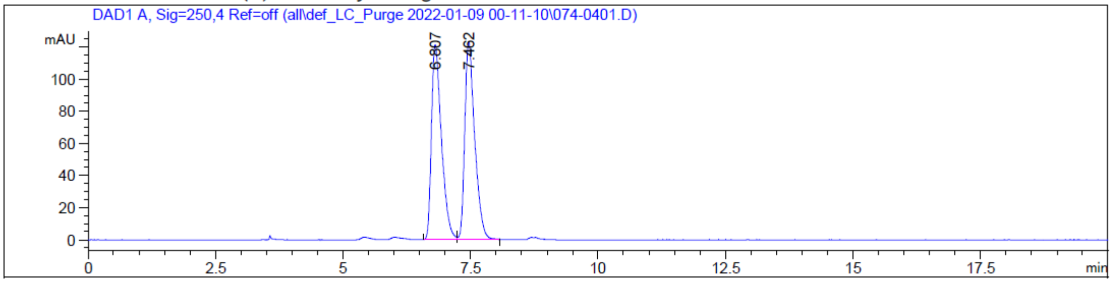

Peak #	RetTime [min]	Type	Width [min]	Area [mAU*s]	Height [mAU]	Area %
1	6.807	BV	0.2023	1647.62732	121.40546	49.7088
2	7.462	VB	0.1998	1666.92920	123.26158	50.2912

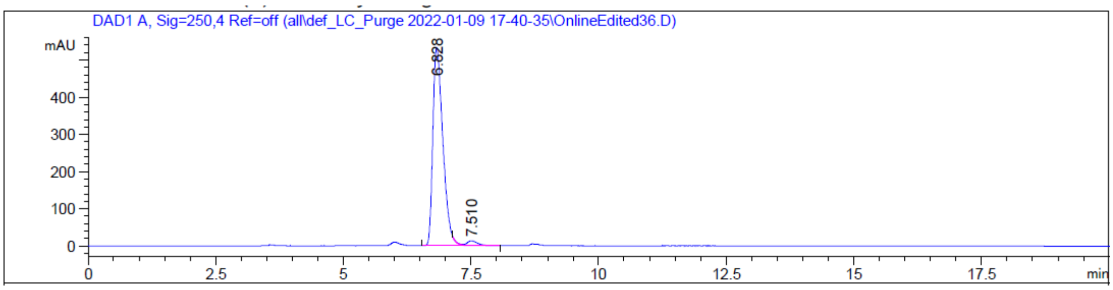

Peak #	RetTime [min]	Type	Width [min]	Area [mAU*s]	Height [mAU]	Area %
1	6.828	BV R	0.2076	7280.73193	532.24390	97.3355
2	7.510	VB E	0.2034	199.30746	13.18425	2.6645

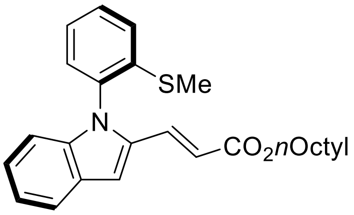

Octyl (*E*)-3-(1-(2-(methylthio)phenyl)-1H-indol-2-yl)acrylate (3e)

The general procedure was followed using 1-(2-(methylthio)phenyl)-1*H*-indole (23.9 mg, 0.10 mmol), *n*-octyl acrylate (55.3 mg, 0.30 mmol) under oxygen atmosphere (O_2 balloon) at 65 °C for 24 h. Purification by column chromatography on silica gel (*n*-hexane/EtOAc: 20/1) yielded **3e** (37.4 mg, 89%) as a yellow oil. ^{1}H NMR (400 MHz, $CDCl_3$): δ = 7.70 – 7.65 (m, 1H), 7.53 (ddd, J = 8.6, 7.3, 1.7 Hz, 1H), 7.41 – 7.30 (m, 3H), 7.29 – 7.25 (m, 1H), 7.23 – 7.14 (m, 2H), 7.13 (s, 1H), 6.93 – 6.87 (m, 1H), 6.20 (d, J = 16.0 Hz, 1H), 4.12 (t, J = 6.7 Hz, 2H), 2.31 (s, 3H), 1.69 – 1.61 (m, 2H), 1.37 – 1.24 (m, 10H), 0.93 – 0.87 (m, 3H). ^{13}C NMR (101 MHz, $CDCl_3$): δ = 167.1 (C_q), 140.0 (C_q), 139.3 (C_q), 135.2 (C_q), 133.9 (C_q), 133.1 (CH), 129.9 (CH), 129.9 (CH), 127.6 (C_q), 125.8 (CH), 125.5 (CH), 124.1 (CH), 121.3 (CH), 121.1 (CH), 117.7 (CH), 110.7 (CH), 105.4 (CH), 64.6 (CH_2), 31.8 (CH_2), 29.2 (CH_2), 29.2 (CH_2), 28.6 (CH_2), 25.9 (CH_2), 22.6 (CH_2), 14.6 (CH_3), 14.1 (CH_3). IR (ATR): 2953, 2924, 2855, 1709, 1629, 1476, 1268, 1167, 971, 749 cm^{-1}. MS (ESI) *m*/*z* (relative intensity): 444 (100) [M + Na]$^+$, 865 (85) [2M + Na]$^+$, 422 (46) [M + H]$^+$. HR-MS (ESI): *m*/*z* calcd. for $[C_{26}H_{31}NO_2S + Na]^+$ 444.1968, found 444.1963. $[\alpha]_D^{20}$ = +15.5 (c = 0.65, $CHCl_3$). HPLC separation (Chiralpak® IB-3, *n*-hexane/*i*-PrOH 90:10, 1.0 mL/min, detection at 250 nm): t_r (major) = 5.5 min, t_r (minor) = 7.4 min, 92% ee.

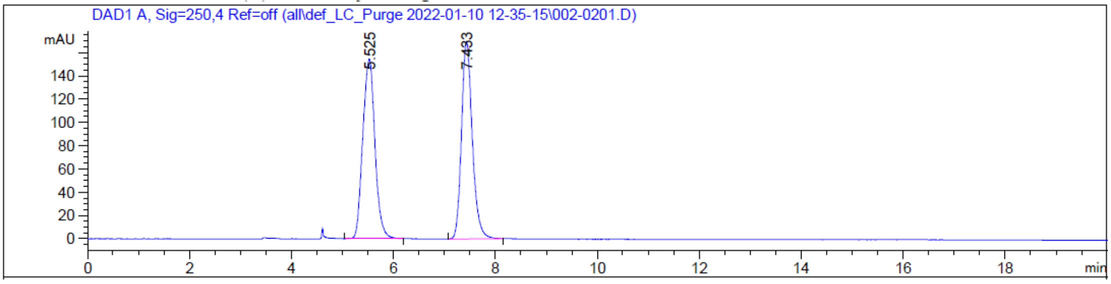

Peak #	RetTime [min]	Type	Width [min]	Area [mAU*s]	Height [mAU]	Area %
1	5.525	BB	0.2635	2559.94482	154.33507	50.7491
2	7.433	VV R	0.2229	2484.37231	169.60330	49.2509

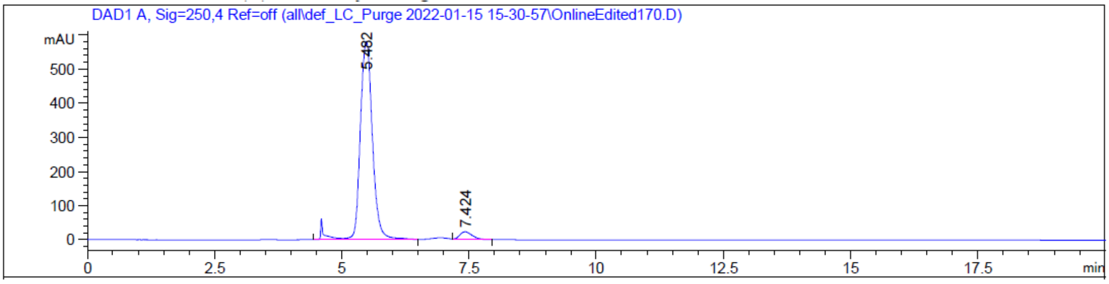

Peak #	RetTime [min]	Type	Width [min]	Area [mAU*s]	Height [mAU]	Area %
1	5.482	VB R	0.2585	9825.46875	581.22021	96.1532
2	7.424	VB	0.2427	393.08371	22.95905	3.8468

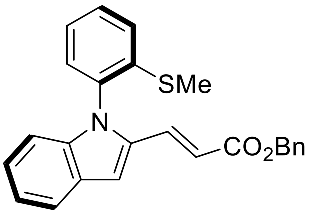

Benzyl (*E*)-3-(1-(2-(methylthio)phenyl)-1H-indol-2-yl)acrylate (3f)

The general procedure was followed using 1-(2-(methylthio)phenyl)-1*H*-indole (23.9 mg, 0.10 mmol), benzyl acrylate (45 μL, 0.30 mmol) under oxygen atmosphere (O_2 balloon) at 65 °C for 24 h. Purification by column chromatography on silica gel (*n*-hexane/EtOAc: 10/1) yielded **3f** (29.3 mg, 73%) as a yellow oil. ^{1}H NMR (400 MHz, $CDCl_3$): δ = 7.72 – 7.64 (m, 1H), 7.53 (ddd, J = 8.4, 7.3, 1.7 Hz, 1H), 7.46 – 7.26 (m, 9H), 7.23 – 7.14 (m, 2H), 7.13 (s, 1H), 6.95 – 6.85 (m, 1H), 6.24 (d, J = 15.9 Hz, 1H), 5.18 (s, 2H), 2.30 (s, 3H). ^{13}C NMR (101 MHz, $CDCl_3$): δ = 166.8 (C_q), 140.0 (C_q), 139.4 (C_q), 136.1 (C_q), 135.0 (C_q), 133.9 (CH), 133.8 (C_q), 129.9 (CH), 129.9 (CH),

128.5 (CH), 128.1 (CH), 127.5 (C_q), 125.8 (CH), 125.5 (CH), 124.2 (CH), 121.4 (CH), 121.2 (CH), 117.0 (CH), 110.8 (CH), 105.8 (CH), 66.1 (CH_2), 14.5 (CH_3). IR (ATR): 3060, 2955, 2917, 1714, 1626, 1476, 1267, 1611, 1016, 749 cm^{-1}. MS (ESI) *m*/*z* (relative intensity): 422 (100) $[M + Na]^+$, 821 (60) $[2M + Na]^+$, 400 (27) $[M + H]^+$. HR-MS (ESI): *m*/*z* calcd. for $[C_{25}H_{21}NO_2S + Na]^+$ 422.1185, found 422.1189. $[\alpha]_D^{20}$ = +13.0 (c = 0.55, $CHCl_3$). HPLC separation (Chiralpak® IB-3, *n*-hexane/*i*-PrOH 90:10, 1.0 mL/min, detection at 250 nm): t_r (major) = 8.0 min, t_r (minor) = 13.3 min, 99% ee.

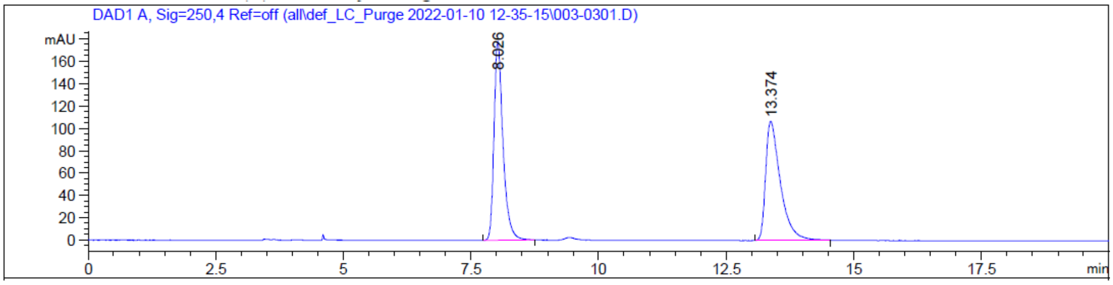

Peak #	RetTime [min]	Type	Width [min]	Area [mAU*s]	Height [mAU]	Area %
1	8.026	BV R	0.1840	2180.56519	177.92290	50.5262
2	13.374	BB	0.2990	2135.15039	106.45121	49.4738

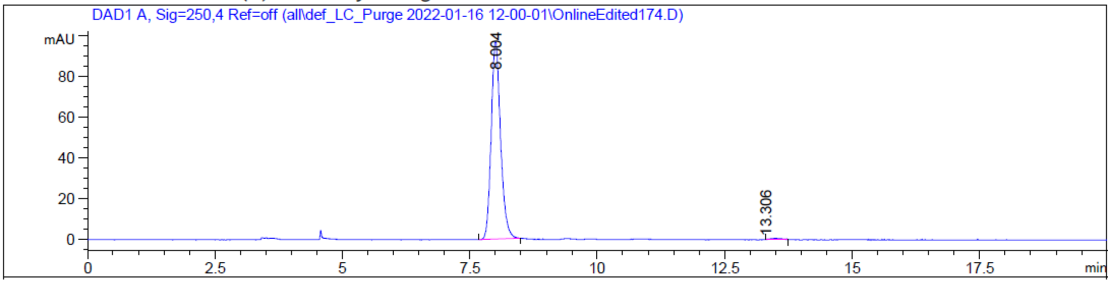

Peak #	RetTime [min]	Type	Width [min]	Area [mAU*s]	Height [mAU]	Area %
1	8.004	BB	0.1950	1240.00696	97.11630	99.2898
2	13.306	MM R	0.2317	8.86943	3.51406e-2	0.7102

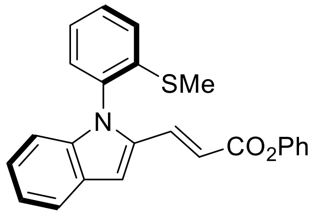

Phenyl (*E*)-3-(1-(2-(methylthio)phenyl)-1H-indol-2-yl)acrylate (3g)

The general procedure was followed using 1-(2-(methylthio)phenyl)-1*H*-indole (23.9 mg, 0.10 mmol), phenyl acrylate (42 µL, 0.30 mmol) under oxygen atmosphere (O_2 balloon) at 65 °C for 24 h. Purification by column chromatography on silica gel (*n*-hexane/EtOAc: 15/1) yielded **3g** (23.4 mg, 61%) as a yellow oil. ^{1}H NMR (400 MHz, $CDCl_3$): δ = 7.75 – 7.69 (m, 1H), 7.57 – 7.51 (m, 2H), 7.41 – 7.31 (m, 5H), 7.25 – 7.18 (m, 4H), 7.11 (dd, J = 8.5, 1.2 Hz, 2H), 6.93 (dd, J = 8.1, 1.2 Hz, 1H), 6.32 (d, J = 16.0 Hz, 1H), 2.33 (s, 3H). ^{13}C NMR (101 MHz, $CDCl_3$): δ = 165.4 (C_q), 150.8 (C_q), 140.0 (C_q), 139.6 (C_q), 135.1 (CH), 134.8 (C_q), 133.8 (C_q), 130.0 (CH), 129.9 (CH), 129.3 (CH), 127.6 (C_q), 125.8 (CH), 125.6 (CH), 125.6 (CH), 124.5 (CH), 121.6 (CH), 121.6 (CH), 121.3 (CH), 116.2 (CH), 110.8 (CH), 106.7 (CH), 14.6 (CH_3). IR (ATR): 3062, 2922, 1726, 1625, 1590, 1476, 1343, 1192, 1129, 971, 748 cm^{-1}. MS (ESI) *m*/*z* (relative intensity): 408 (100) [M + Na]$^+$, 386 (50) [M + H]$^+$. HR-MS (ESI): *m*/*z* calcd. for [$C_{24}H_{29}NO_2S$ + Na]$^+$ 408.1029, found 408.1025. $[\alpha]_D^{20}$ = +3.7 (c = 0.6, $CHCl_3$). HPLC separation (Chiralpak® IB-3, *n*-hexane/*i*-PrOH 95:5, 1.0 mL/min, detection at 250 nm): t_r (major) = 10.4 min, t_r (minor) = 11.3 min, 91% ee.

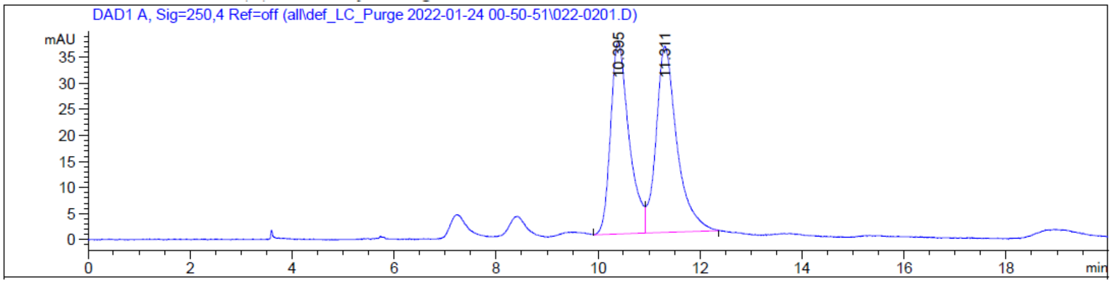

Peak #	RetTime [min]	Type	Width [min]	Area [mAU*s]	Height [mAU]	Area %
1	10.395	BV	0.3359	926.39337	36.89547	47.7106
2	11.311	VB	0.3450	1015.29968	35.68132	52.2894

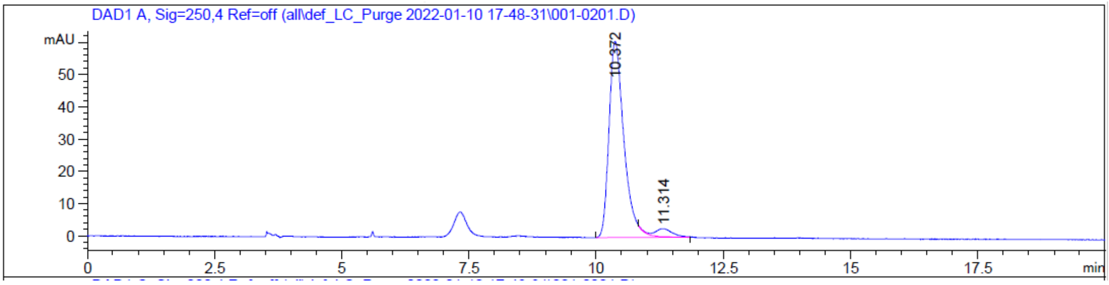

Peak #	RetTime [min]	Type	Width [min]	Area [mAU*s]	Height [mAU]	Area %
1	10.372	BV R	0.3050	1245.51929	60.79882	95.5737
2	11.314	VB E	0.2679	57.68383	2.54070	4.4263

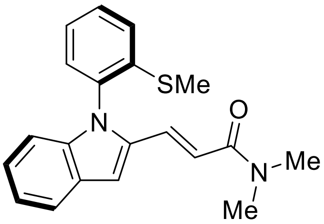

(*E*)-*N*,*N*-dimethyl-3-(1-(2-(methylthio)phenyl)-1H-indol-2-yl)acrylamide (3h)

The general procedure was followed using 1-(2-(methylthio)phenyl)-1*H*-indole (23.9 mg, 0.10 mmol), *N*,*N*-dimethylacrylamide (32 µL, 0.30 mmol) under oxygen atmosphere (O_2 balloon) at 65 °C for 24 h. Purification by column chromatography on silica gel (*n*-hexane/EtOAc: 15/1) yielded **3h** (22.4 mg, 67%) as a yellow solid. ^{1}H NMR (400 MHz, $CDCl_3$): δ = 7.69 – 7.64 (m, 1H), 7.50 (ddd, J = 7.6, 6.7, 2.1 Hz, 1H), 7.43 – 7.35 (m, 2H), 7.33 – 7.27 (m, 2H), 7.20 – 7.12 (m, 2H), 7.05 (s, 1H), 6.91 – 6.87 (m, 1H), 6.51 (d, J = 15.5 Hz, 1H), 2.98 (s, 3H), 2.93 (s, 3H), 2.30 (s, 3H). ^{13}C NMR (101 MHz, $CDCl_3$): δ = 166.4 (C_q), 140.1 (C_q), 139.2 (C_q), 136.0 (C_q), 134.5 (C_q), 131.2 (CH), 130.0 (CH), 129.7 (CH), 127.6 (C_q), 125.6 (CH), 125.5 (CH), 123.7 (CH), 121.1 (CH), 120.9 (CH), 116.8 (CH), 110.6 (CH), 105.4 (CH), 37.0 (CH_3), 35.8 (CH_3), 14.6 (CH_3). IR (ATR): 3056, 2922, 2855, 1645, 1601, 1475, 1393, 1131, 968, 748 cm^{-1}. MS (ESI) *m*/*z* (relative intensity): 337 (100) $[M + H]^+$, 359 (33) $[M + Na]^+$. HR-MS (ESI): *m*/*z* calcd. for $[C_{20}H_{20}N_2OS + H]^+$ 337.1369, found 337.1367. $[\alpha]_D^{20}$ = +29.6 (c = 0.45, $CHCl_3$). HPLC separation (Chiralpak® IB-3, *n*-hexane/*i*-PrOH 80:20, 1.0 mL/min, detection at 250 nm): t_r (major) = 12.7 min, t_r (minor) = 15.9 min, 90% ee.

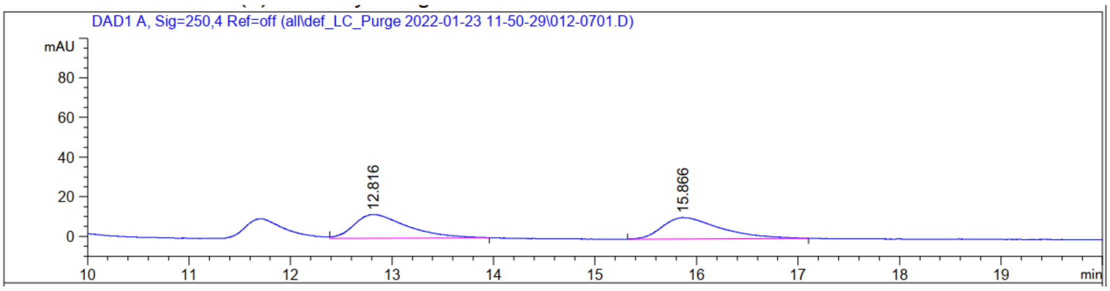

Peak #	RetTime [min]	Type	Width [min]	Area [mAU*s]	Height [mAU]	Area %
1	12.816	VB	0.4263	431.45480	11.89013	50.3067
2	15.866	BV R	0.4650	426.19315	10.78649	49.6933

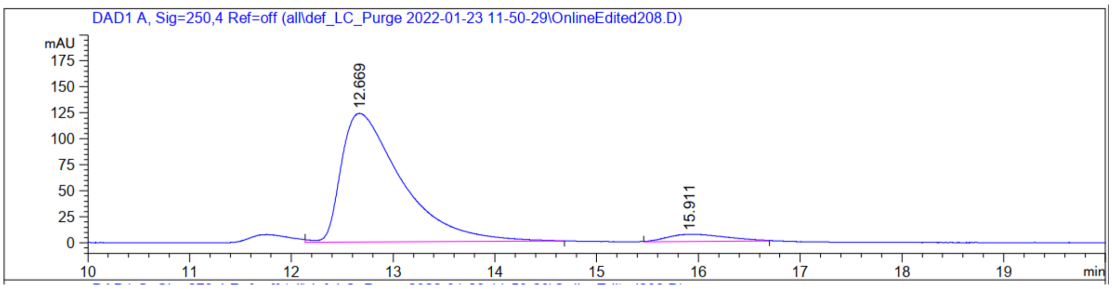

Peak #	RetTime [min]	Type	Width [min]	Area [mAU*s]	Height [mAU]	Area %
1	12.669	FM R	0.6853	5086.93018	123.72156	94.9439
2	15.911	MM R	0.6615	270.89563	6.82530	5.0561

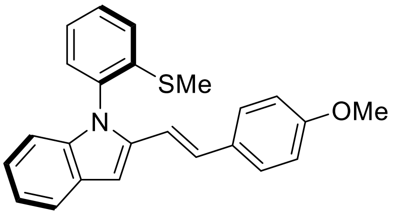

(*E*)-2-(4-methoxystyryl)-1-[2-(methylthio)phenyl]-1H-indole (3i)

The general procedure was followed using 1-(2-(methylthio)phenyl)-1*H*-indole (23.9 mg, 0.10 mmol), 1-methoxy-4-vinylbenzene (40.3 mg, 0.30 mmol) under oxygen atmosphere (O_2 balloon) at 65 °C for 48 h. Purification by column chromatography on silica gel (*n*-hexane/EtOAc: 30/1) yielded **3i** (21.5 mg, 58%) as a yellow solid. ^{1}H NMR (400 MHz, $CDCl_3$): δ = 7.67 – 7.60 (m, 1H), 7.53 (ddd, J = 8.0, 5.1, 3.8 Hz, 1H), 7.44 – 7.38 (m, 1H), 7.35 – 7.30 (m, 2H), 7.30 – 7.24 (m, 2H), 7.13 (tt, J = 7.1, 5.5 Hz, 2H), 7.01 (d, J = 16.4 Hz, 1H), 6.95 (s, 1H), 6.91 – 6.87 (m, 1H), 6.83 (d, J = 8.8 Hz, 2H), 6.55 (dd, J = 16.3, 0.7 Hz, 1H), 3.80 (s, 3H), 2.31 (s, 3H). ^{13}C NMR (101 MHz, $CDCl_3$):

δ = 159.3 (C_q), 140.2 (C_q), 138.8 (C_q), 138.2 (C_q), 134.7 (C_q), 130.2 (CH), 130.0 (C_q), 129.6 (CH), 129.4 (CH), 128.3 (C_q), 127.7 (CH), 125.6 (CH), 125.3 (CH), 122.0 (CH), 120.6 (CH), 120.3 (CH), 115.5 (CH), 114.0 (CH), 110.2 (CH), 99.5 (CH), 55.3 (CH_3), 14.7 (CH_3). MS (ESI) *m/z* (relative intensity): 372 (100) $[M + H]^+$, 394 (70) $[M + Na]^+$. HR-MS (ESI): *m/z* calcd. for $[C_{24}H_{21}NOS + H]^+$ 372.1417, found 372.1428. $[\alpha]_D^{20}$ = -5.0 (c = 0.4, $CHCl_3$). HPLC separation (Chiralpak® IB-3, *n*-hexane/*i*-PrOH 99.5:0.5, 1.0 mL/min, detection at 250 nm): t_r (major) = 17.1 min, t_r (minor) = 18.9 min, 97% ee.

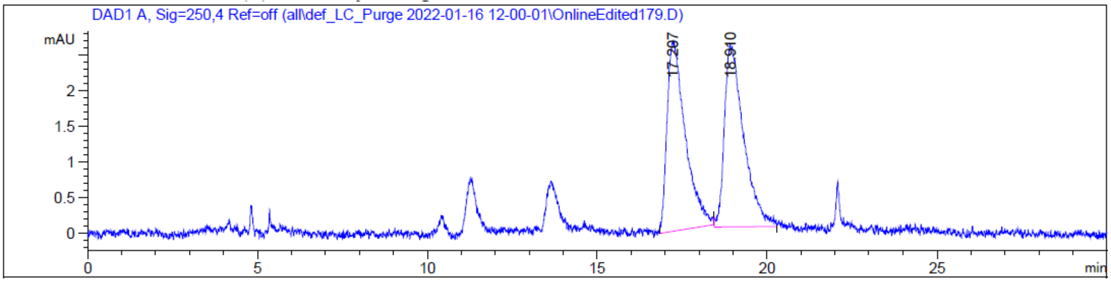

Peak #	RetTime [min]	Type	Width [min]	Area [mAU*s]	Height [mAU]	Area %
1	17.207	PM R	0.6236	99.80242	2.66723	49.7758
2	18.910	MP R	0.6534	100.70139	2.56877	50.2242

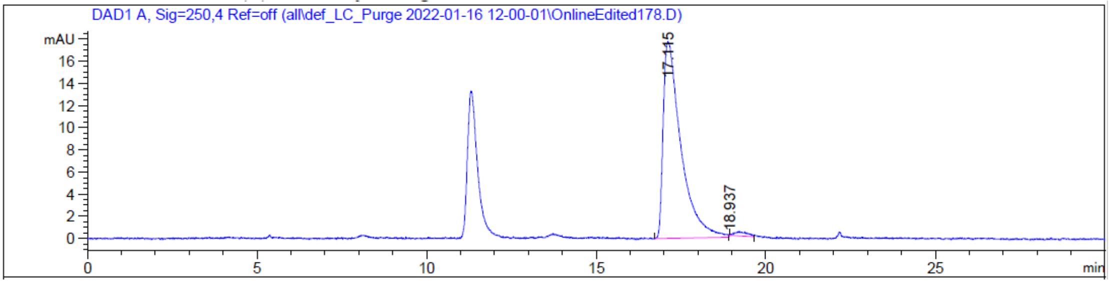

Peak #	RetTime [min]	Type	Width [min]	Area [mAU*s]	Height [mAU]	Area %
1	17.115	PM R	0.6052	645.85883	17.78571	98.5623
2	18.937	MM R	0.4362	9.42072	7.97025e-2	1.4377

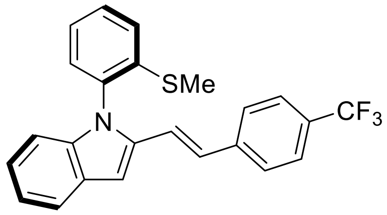

(*E*)-1-(2-(methylthio)phenyl)-2-(4-(trifluoromethyl)styryl)-1H-indole (3j)

The general procedure was followed using 1-(2-(methylthio)phenyl)-1*H*-indole (23.9 mg, 0.10 mmol), 1-(trifluoromethyl)-4-vinylbenzene (51.7 mg, 0.30 mmol) under oxygen atmosphere (O_2 balloon) at 65 °C for 48 h. Purification by column chromatography on silica gel (*n*-hexane/EtOAc: 30/1) yielded **3j** (22.0 mg, 54%) as a yellow solid. ^{1}H NMR (400 MHz, $CDCl_3$): δ = 7.71 – 7.65 (m, 1H), 7.58 – 7.49 (m, 3H), 7.44 – 7.38 (m, 3H), 7.37 – 7.32 (m, 2H), 7.20 – 7.13 (m, 2H), 7.06 – 6.98 (m, 2H), 6.95 – 6.88 (m, 1H), 6.78 (d, J = 16.3 Hz, 1H), 2.32 (s, 3H). ^{13}C NMR (101 MHz, $CDCl_3$): δ = 140.7 (C_q), 140.2 (C_q), 138.6 (C_q), 137.6 (C_q), 134.4 (C_q), 130.1 (CH), 129.7 (CH), 129.1 (C_q, q, J = 32.5 Hz), 128.0 (C_q), 127.9 (CH), 126.4 (CH), 125.6 (CH), 125.5 (C_q, q, J = 4.0 Hz), 125.4 (CH), 122.8 (CH), 120.9 (CH), 120.7 (CH), 120.1 (CH), 110.4 (CH), 101.4 (CH), 14.6 (CH_3). IR (ATR): 3055, 2923, 1612, 1476, 1322, 1164, 1211, 1066, 819, 747 cm^{-1}. MS (ESI) *m*/*z* (relative intensity): 410 (100) [M + H]$^+$, 432 (20) [M + Na]$^+$. HR-MS (ESI): *m*/*z* calcd. for $[C_{24}H_{18}F_3NS + H]^+$ 410.1185, found 410.1186. $[\alpha]_D^{20}$ = -12.0 (c = 0.25, $CHCl_3$). HPLC separation (Chiralpak® IB-3, *n*-hexane/*i*-PrOH 99.5:0.5, 1.0 mL/min, detection at 250 nm): t_r (major) = 8.2 min, t_r (minor) = 6.6 min, 89% ee.

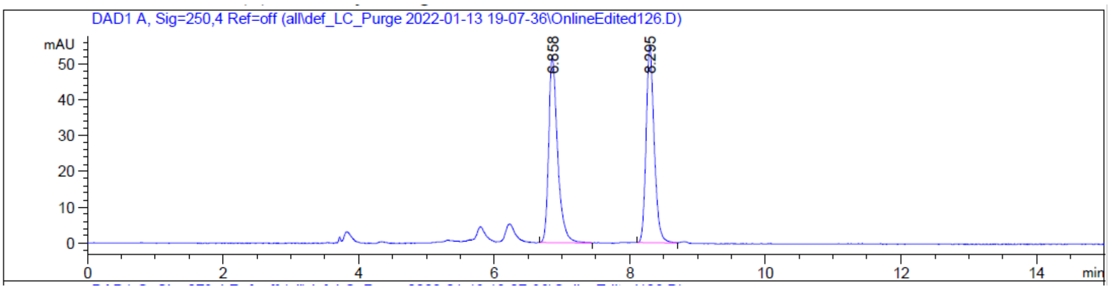

Peak #	RetTime [min]	Type	Width [min]	Area [mAU*s]	Height [mAU]	Area %
1	6.858	BB	0.1362	477.57465	51.95231	51.1114
2	8.295	BB	0.1268	456.80447	55.04470	48.8886

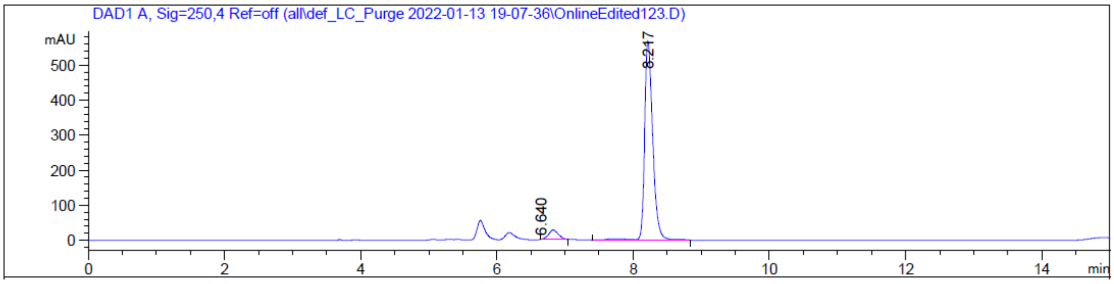

Peak #	RetTime [min]	Type	Width [min]	Area [mAU*s]	Height [mAU]	Area %
1	6.640	PM R	0.1655	271.72302	0.00000	5.4187
2	8.217	VV R	0.1245	4742.84375	569.47943	94.5813

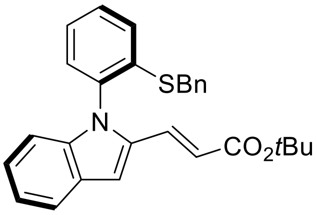

Tert-butyl (_E_)-3-(1-(2-(benzylthio)phenyl)-1H-indol-2-yl)acrylate (3k)

The general procedure was followed using 1-(2-(benzylthio)phenyl)-1*H*-indole (31.6 mg, 0.10 mmol), *tert*-butyl acrylate (44 µL, 0.30 mmol) at 65 °C for 24 h. Purification by column chromatography on silica gel (*n*-hexane/EtOAc: 20/1) yielded **3k** (23.8 mg, 54%) as a yellow oil. ^{1}H NMR (400 MHz, $CDCl_3$): δ = 7.71 – 7.63 (m, 1H), 7.47 – 7.39 (m, 2H), 7.36 – 7.30 (m, 1H), 7.29 – 7.13 (m, 9H), 7.10 (s, 1H), 6.89 – 6.83 (m, 1H), 6.19 (d, J = 15.9 Hz, 1H), 3.95 (s, 2H), 1.50 (s, 9H). ^{13}C NMR (101 MHz, $CDCl_3$): δ = 166.3 (C_q), 139.4 (C_q), 138.0 (C_q), 136.2 (C_q), 135.6 (C_q), 135.2 (C_q), 132.4 (CH), 130.1 (CH), 129.6 (CH), 129.0 (CH), 128.8 (CH), 128.5 (CH), 127.6 (C_q), 127.3 (CH), 126.7 (CH), 123.9 (CH), 121.2 (CH), 121.0 (CH), 119.7 (CH), 110.8 (CH), 104.7 (CH), 80.3 (C_q), 37.1 (CH_2), 28.2 (CH_3). IR (ATR): 3063, 2975, 2926, 1701, 1627, 1476, 1452, 1366, 1151, 748 cm^{-1}. MS (ESI) *m/z* (relative intensity): 464 (100) $[M + Na]^+$, 442 (40) $[M + H]^+$. HR-MS (ESI): *m/z* calcd. for $[C_{28}H_{27}NO_2S + Na]^+$ 464.1655, found 464.1669. $[\alpha]_D^{20}$ = -10.0 (c = 0.17, $CHCl_3$). HPLC separation (Chiralpak® IB-3, *n*-hexane/*i*-PrOH 98:2, 1.0 mL/min, detection at 250 nm): t_r (major) = 7.6 min, t_r (minor) = 8.0 min, 85% ee.

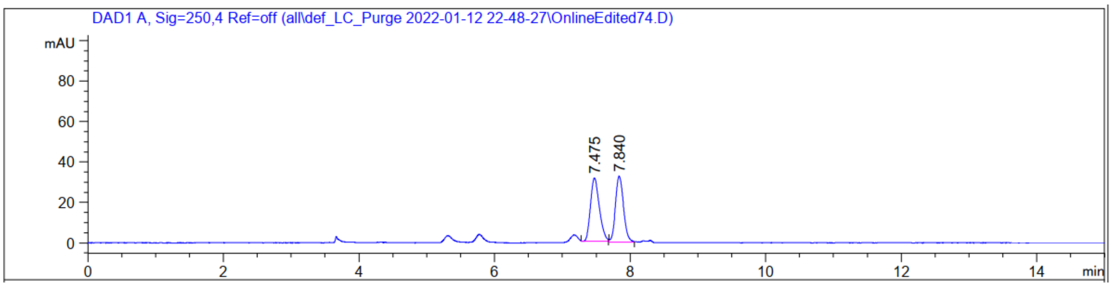

Peak #	RetTime [min]	Type	Width [min]	Area [mAU*s]	Height [mAU]	Area %
1	7.475	MM R	0.1525	286.43988	31.31384	51.3961
2	7.840	MM R	0.1387	270.87866	32.54498	48.6039

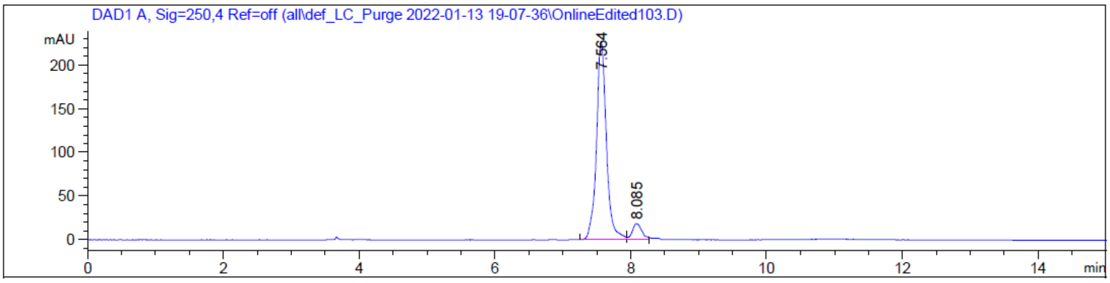

Peak #	RetTime [min]	Type	Width [min]	Area [mAU*s]	Height [mAU]	Area %
1	7.564	MF R	0.1694	2306.81763	227.00928	92.7271
2	8.085	MF R	0.1644	180.93037	18.34519	7.2729

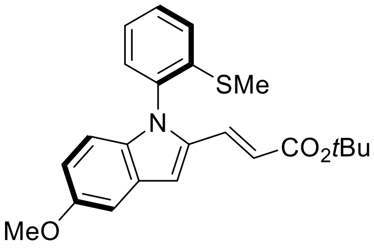

***Tert*-butyl (*E*)-3-(5-methoxy-1-(2-(methylthio)phenyl)-1H-indol-2-yl)acrylate (3l)**

The general procedure was followed using 5-methoxy-1-(2-(methylthio)phenyl)-1*H*-indole (27.0 mg, 0.10 mmol), *tert*-butyl acrylate (44 µL, 0.30 mmol) at 65 °C for 24 h. Purification by column chromatography on silica gel (*n*-hexane/EtOAc: 10/1) yielded **3l** (33.7 mg, 85%) as a red oil. ^{1}H NMR (400 MHz, $CDCl_3$): δ = 7.50 (ddd, *J* = 7.9, 7.2, 1.7 Hz, 1H), 7.36 (dd, *J* = 8.1, 1.3 Hz, 1H), 7.30 (td, *J* = 7.5, 1.3 Hz, 1H), 7.27 – 7.19 (m, 2H), 7.09 (d, *J* = 2.3 Hz, 1H), 7.02 (s, 1H), 6.85 (dd, *J* = 8.9, 2.4 Hz, 1H), 6.81 – 6.76 (m, 1H), 6.19 (d, *J* = 15.9 Hz, 1H), 3.86 (s, 3H), 2.30 (s, 3H), 1.47 (s, 9H). ^{13}C

NMR (101 MHz, $CDCl_3$): δ = 166.4 (C_q), 155.0 (C_q), 140.0 (C_q), 135.7 (C_q), 134.6 (C_q), 133.9 (C_q), 132.2 (CH), 129.9 (CH), 129.8 (CH), 127.9 (C_q), 125.7 (CH), 125.4 (CH), 119.3 (CH), 114.9 (CH), 111.6 (CH), 104.1 (CH), 101.9 (CH), 80.3 (C_q), 55.7 (CH_3), 28.2 (CH_3), 14.6 (CH_3). IR (ATR): 2977, 2929, 2831, 1699, 1617, 1477, 1308, 1204, 1147, 1033, 972 cm^{-1}. MS (ESI) *m/z* (relative intensity): 813 (100) $[2M + Na]^+$, 418 (80) $[M + Na]^+$, 396 (40) $[M + Na]^+$. HR-MS (ESI): *m/z* calcd. for $[C_{26}H_{25}NO_3S + Na]^+$ 418.1447, found 418.1443. $[\alpha]_D^{20}$ = +0.6 (c = 0.17, $CHCl_3$). HPLC separation (Chiralpak® IB-3, *n*-hexane/*i*-PrOH 90:10, 1.0 mL/min, detection at 250 nm): t_r (major) = 5.8 min, t_r (minor) = 6.4 min, 91% ee.

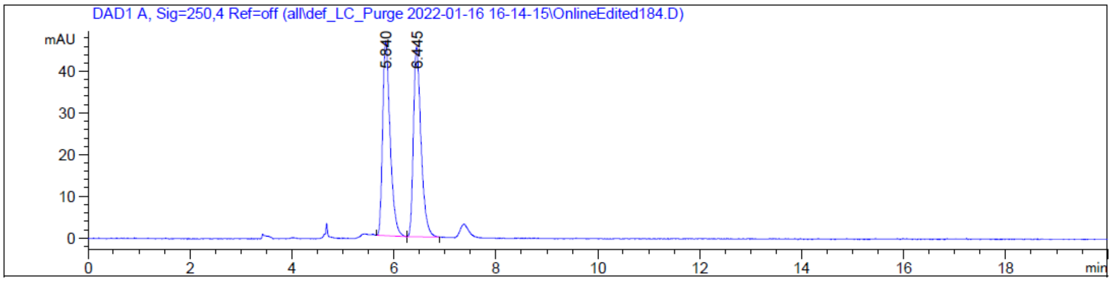

Peak #	RetTime [min]	Type	Width [min]	Area [mAU*s]	Height [mAU]	Area %
1	5.840	BB	0.1537	476.28299	46.82980	50.1537
2	6.445	BB	0.1567	473.36362	45.76543	49.8463

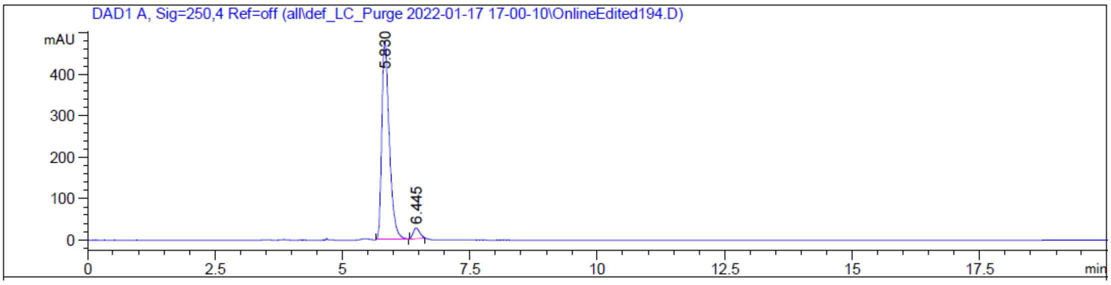

Peak #	RetTime [min]	Type	Width [min]	Area [mAU*s]	Height [mAU]	Area %
1	5.830	PM R	0.1705	4897.96777	478.90344	95.4324
2	6.445	MP R	0.1507	234.42715	25.92757	4.5676

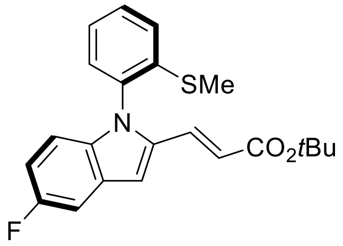

***Tert*-butyl (*E*)-3-(5-fluoro-1-(2-(methylthio)phenyl)-1H-indol-2-yl)acrylate (3m)**

The general procedure was followed using 5-fluoro-1-(2-(methylthio)phenyl)-1*H*-indole (25.8 mg, 0.10 mmol), *tert*-butyl acrylate (44 µL, 0.30 mmol) at 65 °C for 24 h. Purification by column chromatography on silica gel (*n*-hexane/EtOAc: 15/1) yielded **3m** (31.3 mg, 82%) as a yellow oil. ^{1}H NMR (400 MHz, $CDCl_3$): δ = 7.52 (ddd, J = 8.0, 7.3, 1.6 Hz, 1H), 7.36 (dd, J = 8.1, 1.4 Hz, 1H), 7.33 – 7.28 (m, 2H), 7.24 (dd, J = 7.7, 1.6 Hz, 1H), 7.22 – 7.14 (m, 1H), 7.04 (s, 1H), 6.92 (td, J = 9.0, 2.5 Hz, 1H), 6.83 – 6.76 (m, 1H), 6.22 (d, J = 16.0 Hz, 1H), 2.31 (s, 3H), 1.47 (s, 9H). ^{13}C NMR (400 MHz, $CDCl_3$): δ = 166.2 (C_q), 159.7 (C_q), 157.4 (C_q), 139.9 (C_q), 136.9 (C_q), 135.7 (C_q), 133.5 (C_q), 131.9 (CH), 129.9 (CH, d, J = 14.0 Hz), 127.8 (C_q, d, J = 10.3 Hz), 125.6 (CH, d, J = 21.9 Hz), 120.5 (CH), 112.5 (CH, d, J = 26.6 Hz), 111.6 (CH, d, J = 9.5 Hz), 105.8 (CH), 105.55 (CH), 104.1 (CH, d, J = 5.1 Hz), 80.5 (C_q), 28.1 (CH_3), 14.5 (CH_3). ^{19}F NMR (377 MHz, $CDCl_3$): δ = -122.93 (td, J = 9.2, 4.5 Hz). IR (ATR): 2977, 2924, 2858, 1702, 1618, 1477, 1391, 1310, 1148, 755 cm^{-1}. MS (ESI) *m*/*z* (relative intensity): 406 (100) [M + Na]$^+$, 789 (65) [2M + Na]$^+$, 384 (25) [M + H]$^+$. HR-MS (ESI): *m*/*z* calcd. for [$C_{22}H_{22}FNO_2S$ + Na]$^+$ 406.1247, found 406.1240. $[\alpha]_D^{20}$ = +5.1 (c = 0.45, $CHCl_3$). HPLC separation (Chiralpak® IB-3, *n*-hexane/*i*-PrOH 99:1, 0.75 mL/min, detection at 250 nm): t_r (major) = 9.7 min, t_r (minor) = 10.5 min, 98% ee.

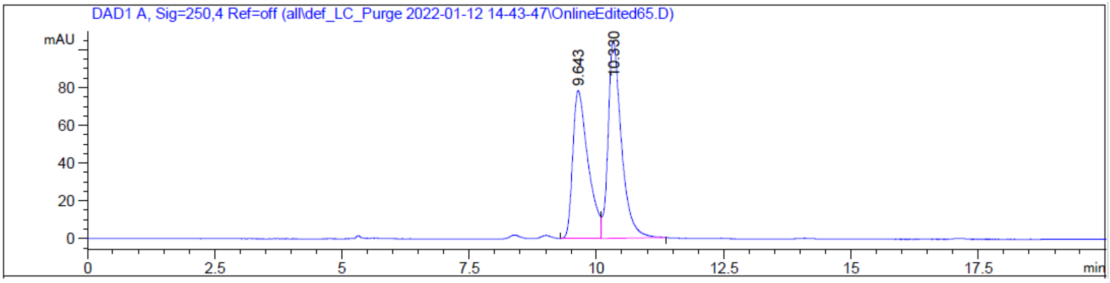

Peak #	RetTime [min]	Type	Width [min]	Area [mAU*s]	Height [mAU]	Area %
1	9.643	BV	0.2973	1636.33496	78.14676	46.2189
2	10.330	VV R	0.2716	1904.06799	104.46341	53.7811

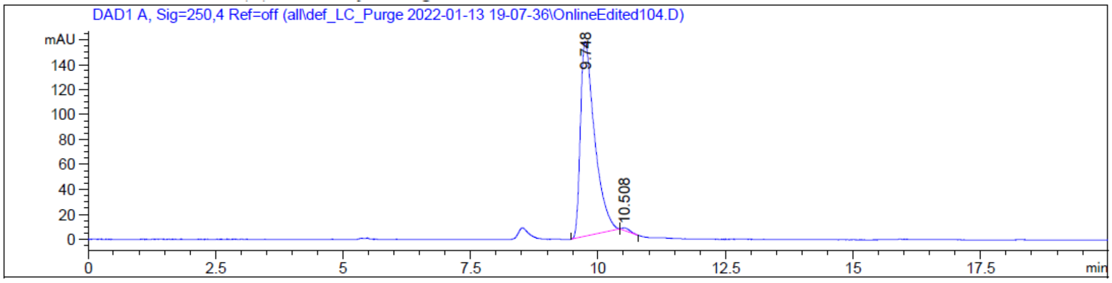

Peak #	RetTime [min]	Type	Width [min]	Area [mAU*s]	Height [mAU]	Area %
1	9.748	BB	0.2856	3019.56323	156.17316	99.2387
2	10.508	BB	0.1523	23.16408	1.84772	0.7613

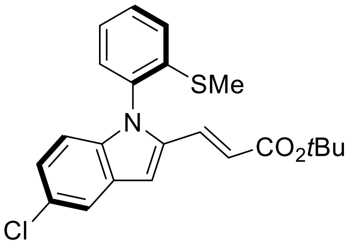

Tert-butyl (_E_)-3-(5-chloro-1-(2-(methylthio)phenyl)-1H-indol-2-yl)acrylate (3n)

The general procedure was followed using 5-chloro-1-(2-(methylthio)phenyl)-1*H*-indole (27.4 mg, 0.10 mmol), *tert*-butyl acrylate (44 µL, 0.30 mmol) at 65 °C for 24 h. Purification by column chromatography on silica gel (*n*-hexane/EtOAc: 15/1) yielded **3n** (29.6 mg, 74%) as a yellow oil. ^{1}H NMR (400 MHz, $CDCl_3$): δ = 7.63 (d, J = 1.9 Hz, 1H), 7.52 (ddd, J = 8.0, 7.4, 1.6 Hz, 1H), 7.36 (dd, J = 8.0, 1.4 Hz, 1H), 7.31 (td, J = 7.5, 1.4 Hz, 1H), 7.24 (dd, J = 7.7, 1.5 Hz, 1H), 7.19 (dd, J = 15.9, 0.6 Hz, 1H), 7.11 (dd, J = 8.7, 2.0 Hz, 1H), 7.01 (s, 1H), 6.80 (d, J = 8.8 Hz, 1H), 6.23 (d, J = 15.9 Hz, 1H), 2.31 (s, 3H), 1.47 (s, 9H). ^{13}C NMR (101 MHz, $CDCl_3$): δ = 166.2 (C_q), 140.0 (C_q), 137.5 (C_q), 136.7 (C_q), 133.4 (C_q), 131.8 (CH), 130.2 (CH), 129.9 (CH), 128.6 (C_q), 126.8 (C_q), 125.8 (CH), 125.6 (CH), 124.2 (CH), 120.8 (CH), 120.5 (CH), 111.9 (CH), 103.7 (CH), 80.6 (C_q), 28.2 (CH_3), 14.6 (CH_3). IR (ATR): 2977, 2925, 1702, 1631, 1477, 1337, 1309, 1145, 971, 754 cm^{-1}. MS (ESI) *m*/*z* (relative intensity): 422 (100) [M + Na]$^+$, 821 (67) [2M + Na]$^+$, 384 (30) [M + H]$^+$. HR-MS (ESI): *m*/*z* calcd.

for $[C_{22}H_{22}ClNO_2S + Na]^+$ 422.0952, found 422.0949. $[\alpha]^{20}_{D}$ = +17.0 (c = 0.2, $CHCl_3$). HPLC separation (Chiralpak® IB-3, *n*-hexane/*i*-PrOH 95:5, 1.0 mL/min, detection at 250 nm): t_r (major) = 9.3 min, t_r (minor) = 10.6 min, 96% ee.

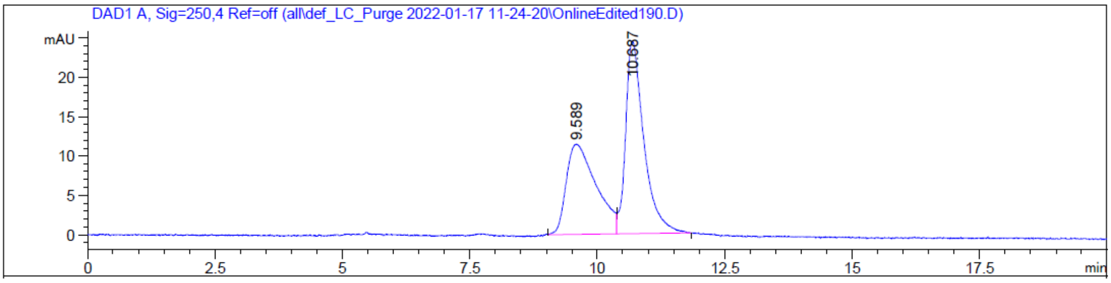

Peak #	RetTime [min]	Type	Width [min]	Area [mAU*s]	Height [mAU]	Area %
1	9.589	BV	0.4791	466.43890	11.45455	43.2956
2	10.687	VV R	0.2977	610.89667	24.51513	56.7044

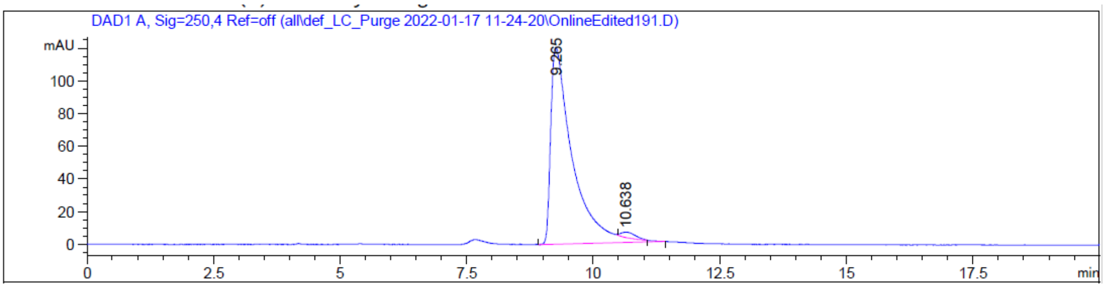

Peak #	RetTime [min]	Type	Width [min]	Area [mAU*s]	Height [mAU]	Area %
1	9.265	BV R	0.4029	3593.15283	120.50101	98.1376
2	10.638	VB E	0.2434	68.18778	3.35137	1.8624

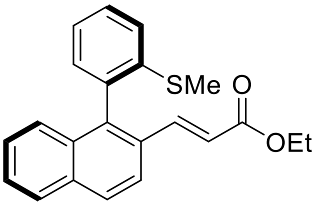

Ethyl (*E*)-3-(1-(2-(methylthio)phenyl)naphthalen-2-yl)acrylate (5a)

The general procedure was followed using methyl(2-(naphthalen-1-yl)phenyl)sulfane (25.1 mg, 0.10 mmol), ethyl acrylate (32 μL, 0.30 mmol) at 65 °C for 48 h. Purification by column chromatography on silica gel (*n*-hexane/EtOAc: 15/1) yielded **5a** (30.1 mg, 86%) as a colorless oil. ^{1}H NMR (400 MHz, $CDCl_3$): δ = 7.92 – 7.81 (m, 3H), 7.53 – 7.46 (m, 3H), 7.40 – 7.32 (m, 3H), 7.30 (td, J = 7.4, 1.2 Hz, 1H), 7.15 (dd, J = 7.5, 1.6

Hz, 1H), 6.49 (d, J = 15.9 Hz, 1H), 4.18 (q, J = 7.1 Hz, 2H), 2.30 (s, 3H), 1.27 (t, J = 7.1 Hz, 3H). ^{13}C NMR (101 MHz, $CDCl_3$): δ = 166.9 (C_q), 142.9 (CH), 139.5 (C_q), 139.1 (C_q), 135.6 (C_q), 134.1 (C_q), 132.5 (C_q), 130.9 (CH), 130.4 (C_q), 128.9 (CH), 128.6 (CH), 128.0 (CH), 127.0 (CH), 126.9 (CH), 126.7 (CH), 124.8 (CH), 124.7 (CH), 122.7 (CH), 119.1 (CH), 60.3 (CH_2), 15.4 (CH_3), 14.2 (CH_3). IR (ATR): 2957, 2926, 2864, 1713, 1631, 1567, 1416, 1250, 1170, 753 cm^{-1}. MS (ESI) m/z (relative intensity): 371 (100) $[M + Na]^+$, 349 (16) $[M + H]^+$. HR-MS (ESI): m/z calcd. for $[C_{22}H_{20}O_2S + Na]^+$ 371.1076, found 371.1075. $[\alpha]_D^{20}$ = -84.4 (c = 0.5, $CHCl_3$). HPLC separation (Chiralpak® AD-3, n-hexane/i-PrOH 95:5, 1.0 mL/min, detection at 250 nm): t_r (major) = 7.8 min, t_r (minor) = 9.2 min, 97% ee.

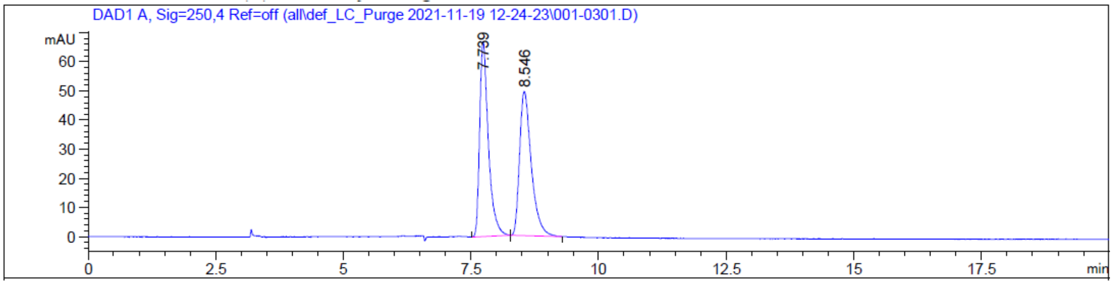

Peak #	RetTime [min]	Type	Width [min]	Area [mAU*s]	Height [mAU]	Area %
1	7.739	BB	0.1776	795.53827	66.94102	50.0946
2	8.546	BB	0.2387	792.53394	49.53605	49.9054

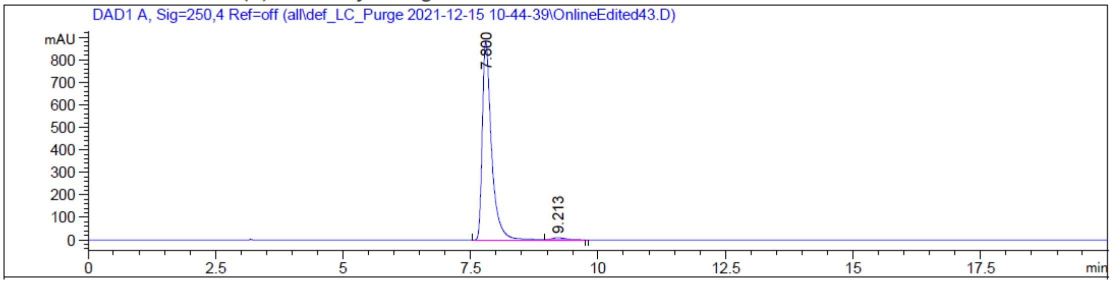

Peak #	RetTime [min]	Type	Width [min]	Area [mAU*s]	Height [mAU]	Area %
1	7.800	BV R	0.1861	1.11159e4	881.71521	98.5592
2	9.213	VB E	0.2080	162.49573	9.62460	1.4408

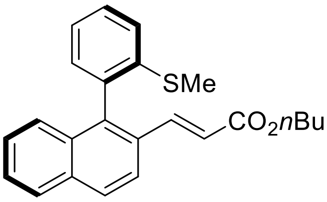

Butyl (*E*)-3-(1-(2-(methylthio)phenyl)naphthalen-2-yl)acrylate (5b)

The general procedure was followed using methyl(2-(naphthalen-1-yl)phenyl)sulfane (25.1 mg, 0.10 mmol), *n*-butyl acrylate (45 µL, 0.30 mmol) at 65 °C for 48 h. Purification by column chromatography on silica gel (*n*-hexane/EtOAc: 20/1) yielded **5b** (32.3 mg, 86%) as a colorless oil. ^{1}H NMR (400 MHz, $CDCl_3$): δ = 7.94 – 7.78 (m, 3H), 7.56 – 7.43 (m, 3H), 7.42 – 7.34 (m, 3H), 7.30 (td, J = 7.4, 1.3 Hz, 1H), 7.15 (dd, J = 7.5, 1.5 Hz, 1H), 6.49 (d, J = 16.0 Hz, 1H), 4.12 (t, J = 6.5 Hz, 2H), 2.30 (s, 3H), 1.67 – 1.54 (m, 2H), 1.42 – 1.29 (m, 2H), 0.92 (t, J = 7.4 Hz, 3H). ^{13}C NMR (101 MHz, $CDCl_3$): δ = 167.0 (C_q), 142.8 (CH), 139.5 (C_q), 139.1 (C_q), 135.6 (C_q), 134.1 (C_q), 132.5 (C_q), 130.9 (CH), 130.4 (C_q), 128.8 (CH), 128.6 (CH), 128.0 (CH), 127.0 (CH), 126.9 (CH), 126.7 (CH), 124.8 (CH), 124.7 (CH), 122.6(CH), 119.1 (CH), 64.2 (CH_2), 30.7 (CH_2), 19.1 (CH_2), 15.4 (CH_3), 13.7 (CH_3). IR (ATR): 2959, 2922, 2865, 1711, 1630, 1468, 1433, 1274, 1174, 752 cm^{-1}. MS (ESI) m/z (relative intensity): 399 (100) $[M + Na]^+$, 377 (15) $[M + H]^+$. HR-MS (ESI): m/z calcd. for $[C_{24}H_{24}O_2S + Na]^+$ 399.1389, found 399.1381. $[\alpha]^{20}_{D}$ = -76.2 (c = 0.65, $CHCl_3$). HPLC separation (Chiralpak® AD-3, *n*-hexane/*i*-PrOH 95:5, 1.0 mL/min, detection at 250 nm): t_r (major) = 7.4 min, t_r (minor) = 8.4 min, 96% ee.

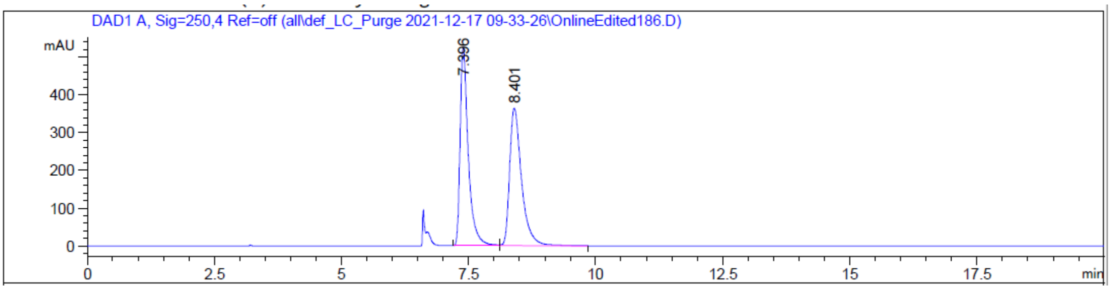

Peak #	RetTime [min]	Type	Width [min]	Area [mAU*s]	Height [mAU]	Area %
1	7.396	BV	0.1652	5859.39307	524.92371	49.6158
2	8.401	VB	0.2465	5950.14551	364.32324	50.3842

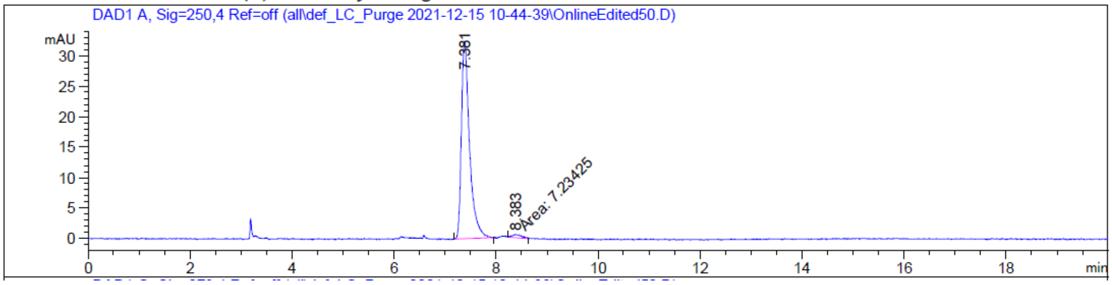

Peak #	RetTime [min]	Type	Width [min]	Area [mAU*s]	Height [mAU]	Area %
1	7.381	BB	0.1633	359.37619	32.43514	98.0267
2	8.383	MM	0.2113	7.23425	5.70561e-1	1.9733

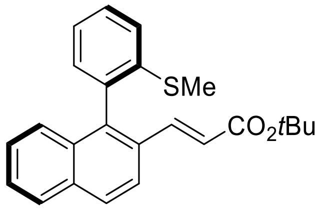

***Tert*-butyl (*E*)-3-(1-(2-(methylthio)phenyl)naphthalen-2-yl)acrylate (5c)**

The general procedure was followed using methyl(2-(naphthalen-1-yl)phenyl)sulfane (25.1 mg, 0.10 mmol), *tert*-butyl acrylate (44 µL, 0.30 mmol) at 65 °C for 48 h. Purification by column chromatography on silica gel (*n*-hexane/EtOAc: 15/1) yielded **5c** (34.6 mg, 92%) as a colorless oil. ^{1}H NMR (400 MHz, $CDCl_3$): δ = 7.92 – 7.81 (m, 3H), 7.53 – 7.46 (m, 2H), 7.42 (d, J = 16.0 Hz, 1H), 7.40 – 7.33 (m, 3H), 7.30 (td, J = 7.4, 1.2 Hz, 1H), 7.15 (dd, J = 7.5, 1.5 Hz, 1H), 6.45 (d, J = 16.0 Hz, 1H), 2.31 (s, 3H), 1.47 (s, 9H). ^{13}C NMR (101 MHz, $CDCl_3$): δ = 166.2 (C_q), 141.8 (CH), 139.3 (C_q), 139.1 (C_q), 135.7 (C_q), 134.0 (C_q), 132.5 (C_q), 131.0 (CH), 130.5 (C_q), 128.8 (CH), 128.5 (CH), 128.0 (CH), 126.9 (CH), 126.9 (CH), 126.7 (CH), 124.8 (CH), 124.7 (CH), 122.7 (CH), 120.9 (CH), 80.2 (C_q), 28.2 (CH_3), 15.5 (CH_3). IR (ATR): 3058, 2973, 2923, 2869, 1707, 1629, 1367, 1299, 1257, 1147, 983 cm^{-1}. MS (ESI) *m*/*z* (relative intensity): 399 (100) [M + Na]$^+$. HR-MS (ESI): *m*/*z* calcd. for [$C_{24}H_{24}O_2S$ + Na]$^+$ 399.1389, found 399.1380. $[\alpha]^{20}_{D}$ = -90.3 (c = 0.6, $CHCl_3$). HPLC separation (Chiralpak® IF-3, *n*-hexane/*i*-PrOH 98:2, 1.0 mL/min, detection at 250 nm): t_r (major) = 7.7 min, t_r (minor) = 8.6 min, 98% ee.

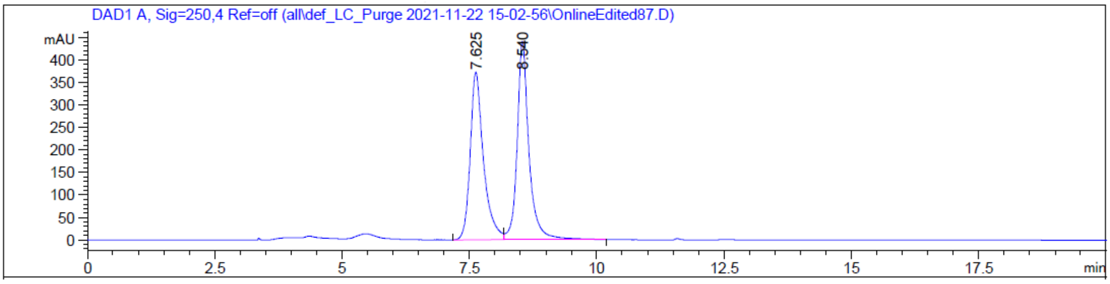

Peak #	RetTime [min]	Type	Width [min]	Area [mAU*s]	Height [mAU]	Area %
1	7.625	BV	0.2637	6743.82227	371.71301	48.6725
2	8.540	VB	0.2335	7111.69922	440.04428	51.3275

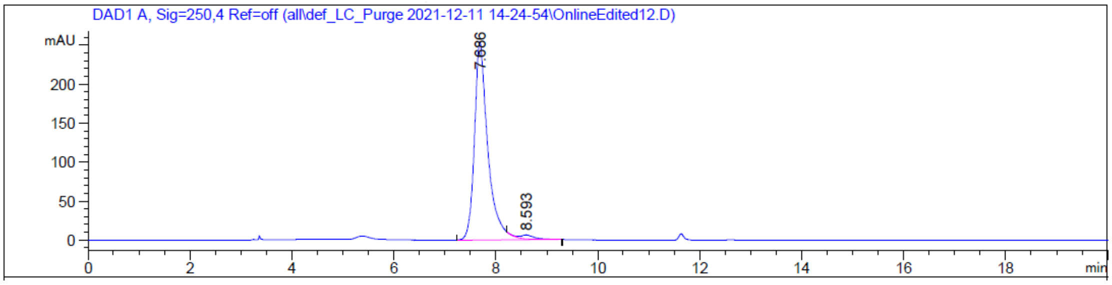

Peak #	RetTime [min]	Type	Width [min]	Area [mAU*s]	Height [mAU]	Area %
1	7.686	BV R	0.2637	4657.56445	254.30746	97.7539
2	8.593	VB E	0.2500	107.01649	5.09518	2.2461

Performed on 1 gram scale

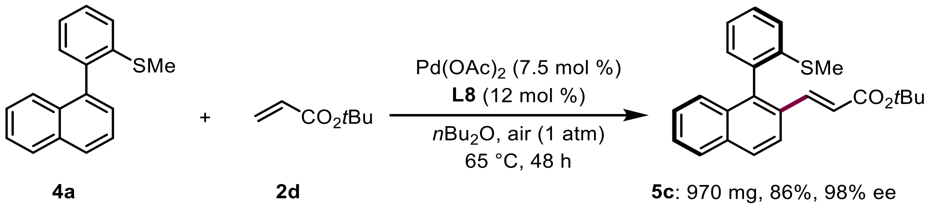

To an oven-dried 500 mL round-bottom flask was added methyl(2-(naphthalen-1-yl)phenyl)sulfane (750 mg, 3.0 mmol), *tert*-butyl acrylate (1152 mg, 9.0 mmol), $Pd(OAc)_2$ (54 mg, 0.225 mmol), **L8** (271 mg, 0.36 mmol), *n*-Bu_2O (60 mL). The mixture was stirred for 48 h at 65 °C under air. The resulting mixture was concentrated, and the residue was purified by silica gel column chromatography on silica gel (*n*-hexane/EtOAc: 15/1) yielded **5c** (970 mg, 86% yield, 98% ee) as a colorless oil.

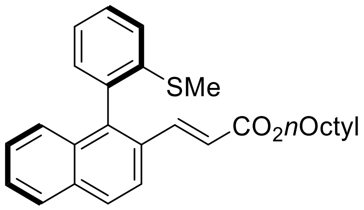

Octyl (*E*)-3-(1-(2-(methylthio)phenyl)naphthalen-2-yl)acrylate (5d)

The general procedure was followed using methyl(2-(naphthalen-1-yl)phenyl)sulfane (25.1 mg, 0.10 mmol), *n*-octyl acrylate (55.3 mg, 0.30 mmol) at 65 °C for 48 h. Purification by column chromatography on silica gel (*n*-hexane/EtOAc: 20/1) yielded **5d** (37.8 mg, 87%) as a white solid. ^{1}H NMR (400 MHz, $CDCl_3$): δ = 7.92 – 7.82 (m, 3H), 7.53 – 7.45 (m, 3H), 7.41 – 7.33 (m, 3H), 7.29 (td, J = 7.4, 1.2 Hz, 1H), 7.15 (dd, J = 7.5, 1.4 Hz, 1H), 6.49 (d, J = 15.9 Hz, 1H), 4.11 (t, J = 6.6 Hz, 2H), 2.30 (s, 3H), 1.66 – 1.59 (m, 2H), 1.38 – 1.23 (m, 10H), 0.90 (t, J = 7.0 Hz, 3H). ^{13}C NMR (101 MHz, $CDCl_3$): δ = 167.0 (C_q), 142.8 (CH), 139.5 (C_q), 139.1 (C_q), 135.6 (C_q), 134.1 (C_q), 132.4 (C_q), 130.9 (CH), 130.4 (C_q), 128.8 (CH), 128.6 (CH), 128.0 (CH), 127.0 (CH), 126.9 (CH), 126.7 (CH), 124.8 (CH), 124.7 (CH), 122.6 (CH), 119.1 (CH), 64.5 (CH_2), 31.8 (CH_2), 29.2 (CH_2), 29.2 (CH_2), 28.6 (CH_2), 25.9 (CH_2), 22.6 (CH_2), 15.4 (CH_3), 14.1 (CH_3). IR (ATR): 3057, 2954, 2854, 1712, 1630, 1467, 1433, 1296, 1173, 754 cm^{-1}. MS (ESI) m/z (relative intensity): 455 (100) $[M + Na]^+$, 887 (50) $[2M + Na]^+$. HR-MS (ESI): m/z calcd. for $[C_{28}H_{32}O_2S + Na]^+$ 455.2015, found 455.2004. $[\alpha]_D^{20}$ = -52.8 (c = 0.5, $CHCl_3$). HPLC separation (Chiralpak® IB-3, *n*-hexane/*i*-PrOH 90:10, 1.0 mL/min, detection at 250 nm): t_r (major) = 5.0 min, t_r (minor) = 6.8 min, 94% ee.

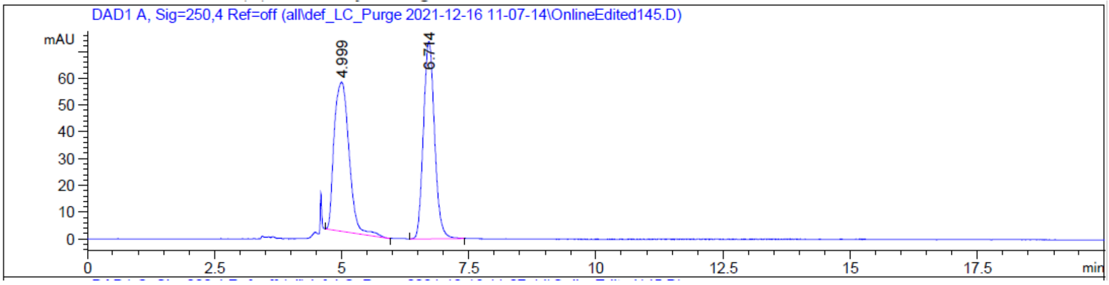

Peak #	RetTime [min]	Type	Width [min]	Area [mAU*s]	Height [mAU]	Area %
1	4.999	BB	0.2907	1136.10547	55.76574	50.3091
2	6.714	BV R	0.2366	1122.14697	73.71240	49.6909

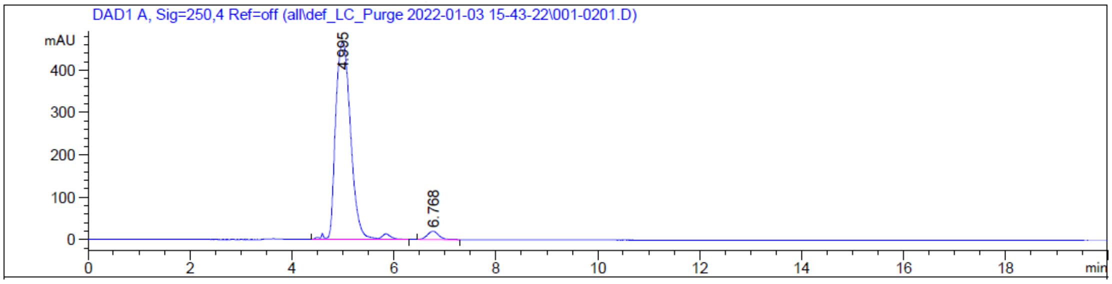

Peak #	RetTime [min]	Type	Width [min]	Area [mAU*s]	Height [mAU]	Area %
1	4.995	VV R	0.3267	9626.60938	466.33575	97.0276
2	6.768	BB	0.2190	294.90942	19.32732	2.9724

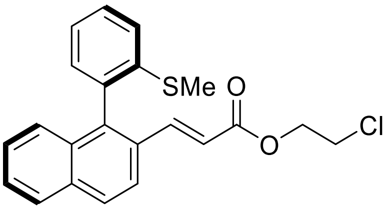

2-Chloroethyl (*E*)-3-(1-(2-(methylthio)phenyl)naphthalen-2-yl)acrylate (5e)

The general procedure was followed using methyl(2-(naphthalen-1-yl)phenyl)sulfane (25.1 mg, 0.10 mmol), 2-chloroethyl acrylate (40.4 mg, 0.30 mmol) at 65 °C for 48 h. Purification by column chromatography on silica gel (*n*-hexane/EtOAc: 10/1) yielded **5e** (34.6 mg, 90%) as a white solid. ^{1}H NMR (400 MHz, $CDCl_3$): δ = 7.94 – 7.81 (m, 3H), 7.58 – 7.46 (m, 3H), 7.42 – 7.34 (m, 3H), 7.30 (td, J = 7.4, 1.2 Hz, 1H), 7.15 (ddd, J = 7.5, 1.5, 0.5 Hz, 1H), 6.52 (d, J = 15.9 Hz, 1H), 4.40 – 4.34 (m, 2H), 3.68 (dd, J = 6.1, 5.5 Hz, 2H), 2.31 (s, 3H). ^{13}C NMR (101 MHz, $CDCl_3$): δ = 166.4 (C_q), 144.0 (CH), 139.8 (C_q), 139.1 (C_q), 135.4 (C_q), 134.2 (C_q), 132.4 (C_q), 130.9 (CH), 130.1 (C_q), 128.9 (CH), 128.6 (CH), 128.0 (CH), 127.2 (CH), 126.9 (CH), 126.8 (CH), 124.8 (CH), 124.7 (CH), 122.6 (CH), 118.0 (CH), 63.9 (C_q), 41.6 (C_q), 15.4 (CH_3). IR (ATR): 2960, 2942, 2864, 1714, 1567, 1456, 1416, 1250, 1168, 778, 748 cm^{-1}. MS (ESI) *m*/*z* (relative intensity): 405 (100) $[M + Na]^+$, 381 (60) $[M - H]^+$. HR-MS (ESI): *m*/*z* calcd. for $[C_{22}H_{19}ClO_2S + Na]^+$ 405.0686, found 405.0694. $[\alpha]_D^{20}$ = -63.6 (c = 0.5, $CHCl_3$). HPLC separation (Chiralpak® AD-3, *n*-hexane/*i*-PrOH 95:5, 1.0 mL/min, detection at 250 nm): t_r (major) = 11.7 min, t_r (minor) = 13.7 min, 94% ee.

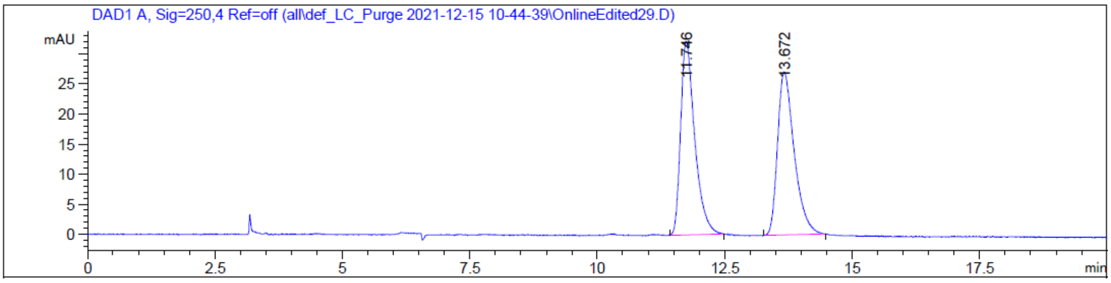

Peak #	RetTime [min]	Type	Width [min]	Area [mAU*s]	Height [mAU]	Area %
1	11.746	BB	0.2775	619.73541	32.20958	50.0906
2	13.672	BB	0.3131	617.49457	27.07166	49.9094

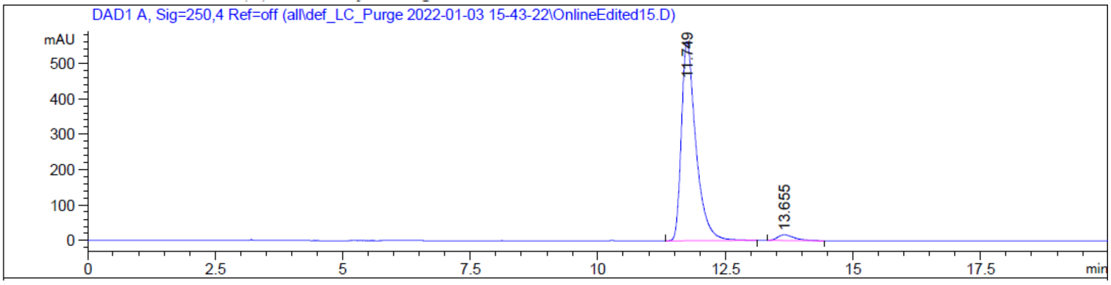

Peak #	RetTime [min]	Type	Width [min]	Area [mAU*s]	Height [mAU]	Area %
1	11.749	BB	0.2953	1.11032e4	562.35260	96.7926
2	13.655	BV R	0.2858	367.92917	16.26992	3.2074

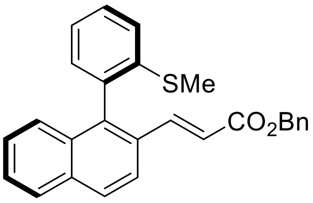

Benzyl (*E*)-3-(1-(2-(methylthio)phenyl)naphthalen-2-yl)acrylate (5f)

The general procedure was followed using methyl(2-(naphthalen-1-yl)phenyl)sulfane (25.1 mg, 0.10 mmol), benzyl acrylate (45 µL, 0.30 mmol) at 65 °C for 48 h. Purification by column chromatography on silica gel (*n*-hexane/EtOAc: 15/1) yielded **5f** (30.6 mg, 75%) as a yellow oil. ^{1}H NMR (400 MHz, $CDCl_3$): δ = 7.85 – 7.67 (m, 3H), 7.48 (d, J = 16.0 Hz, 1H), 7.41 (td, J = 8.0, 7.6, 1.4 Hz, 2H), 7.31 – 7.15 (m, 9H), 7.06 (dd, J = 7.5, 1.5 Hz, 1H), 6.45 (d, J = 16.0 Hz, 1H), 5.08 (s, 2H), 2.20 (s, 3H). ^{13}C NMR (101 MHz, $CDCl_3$): δ = 166.6 (C_q), 143.5 (CH), 139.7 (C_q), 139.1 (C_q), 136.1 (C_q), 135.5 (C_q), 134.2 (C_q), 132.4 (C_q), 130.9 (CH), 130.3 (C_q), 128.9 (CH), 128.6

(CH), 128.5 (CH), 128.0 (CH), 128.0 (CH), 127.8 (CH), 127.1 (CH), 126.9 (CH), 126.8 (CH), 124.7 (CH), 124.7 (CH), 122.6 (CH), 118.6 (CH), 66.0 (CH_2), 15.4 (CH_3). IR (ATR): 3062, 2953, 2922, 1712, 1629, 1433, 1296, 1259, 1169, 1152, 753 cm^{-1}. MS (ESI) *m*/*z* (relative intensity): 433 (100) $[M + Na]^+$, 843 (30) $[2M + H]^+$. HR-MS (ESI): *m*/*z* calcd. for $[C_{27}H_{22}O_2S + Na]^+$ 433.1233, found 433.1234. $[\alpha]^{20}_{D}$ = -92.8 (c = 0.4, $CHCl_3$). HPLC separation (Chiralpak® IB-3, *n*-hexane/*i*-PrOH 90:10, 1.0 mL/min, detection at 250 nm): t_r (major) = 7.2 min, t_r (minor) = 10.9 min, 96% ee.

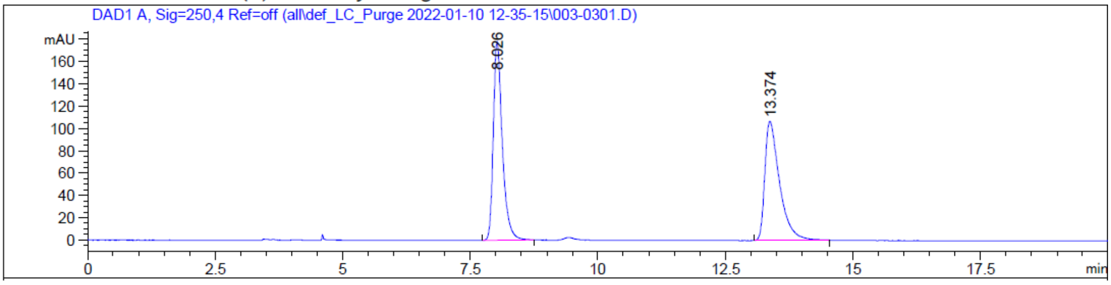

Peak #	RetTime [min]	Type	Width [min]	Area [mAU*s]	Height [mAU]	Area %
1	8.026	BV R	0.1840	2180.56519	177.92290	50.5262
2	13.374	BB	0.2990	2135.15039	106.45121	49.4738

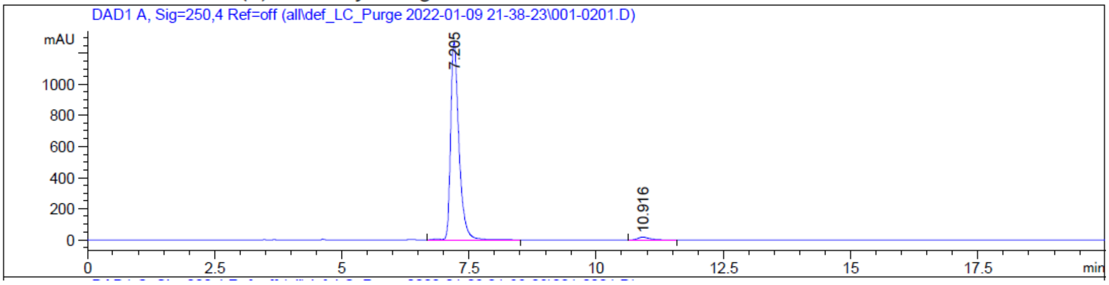

Peak #	RetTime [min]	Type	Width [min]	Area [mAU*s]	Height [mAU]	Area %
1	7.205	VV R	0.1687	1.44823e4	1276.48218	98.0318
2	10.916	BV R	0.2350	290.75903	18.23745	1.9682

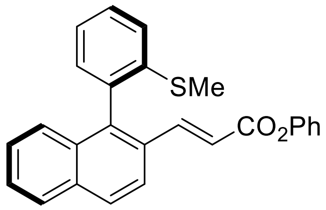

Phenyl (*E*)-3-(1-(2-(methylthio)phenyl)naphthalen-2-yl)acrylate (5g)

The general procedure was followed using methyl(2-(naphthalen-1-yl)phenyl)sulfane (25.1 mg, 0.10 mmol), phenyl acrylate (32 μL, 0.30 mmol) at 65 °C for 48 h. Purification by column chromatography on silica gel (*n*-hexane/EtOAc: 15/1) yielded **5g** (32.8 mg, 83%) as a white solid. ^{1}H NMR (400 MHz, $CDCl_3$): δ = 7.97 – 7.88 (m, 3H), 7.68 (d, J = 15.9 Hz, 1H), 7.53 (ddd, J = 8.1, 6.1, 2.0 Hz, 1H), 7.49 (ddd, J = 8.0, 7.4, 1.5 Hz, 1H), 7.43 – 7.35 (m, 5H), 7.31 (td, J = 7.5, 1.2 Hz, 1H), 7.22 (ddt, J = 8.0, 6.9, 1.2 Hz, 1H), 7.18 (dd, J = 7.5, 1.5 Hz, 1H), 7.14 – 7.09 (m, 2H), 6.70 (d, J = 15.9 Hz, 1H), 2.33 (s, 3H). ^{13}C NMR (101 MHz, $CDCl_3$): δ = 165.4 (C_q), 150.8 (C_q), 144.8 (CH), 140.0 (C_q), 139.1 (C_q), 135.4 (C_q), 134.3 (C_q), 132.5 (C_q), 130.9 (CH), 130.1 (C_q), 129.3 (CH), 129.0 (CH), 128.7 (CH), 128.1 (CH), 127.3 (CH), 127.0 (CH), 126.8 (CH), 125.6 (CH), 124.7 (CH), 124.7 (CH), 122.7 (CH), 121.6 (CH), 118.0 (CH), 15.4 (CH_3). IR (ATR): 3050, 2953, 2922, 1729, 1626, 1472, 1295, 1194, 1163, 1137, 752 cm^{-1}. MS (ESI) *m*/*z* (relative intensity): 815 (100) [2M + Na]$^+$, 419 (90) [M + Na]$^+$. HR-MS (ESI): *m*/*z* calcd. for $[C_{26}H_{20}O_2S + Na]^+$ 419.1076, found 419.1067. $[\alpha]_D^{20}$ = -94.3 (c = 0.6, $CHCl_3$). HPLC separation (Chiralpak® IF-3, *n*-hexane/*i*-PrOH 95:5, 1.0 mL/min, detection at 250 nm): t_r (major) = 8.2 min, t_r (minor) = 7.9 min, 87% ee.

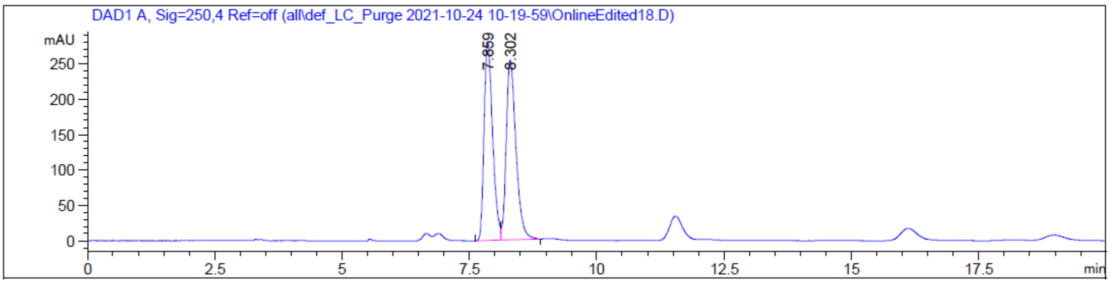

Peak #	RetTime [min]	Type	Width [min]	Area [mAU*s]	Height [mAU]	Area %
1	7.859	BV	0.1777	3263.33276	280.47906	49.1819
2	8.302	VB	0.2018	3371.90381	254.16487	50.8181

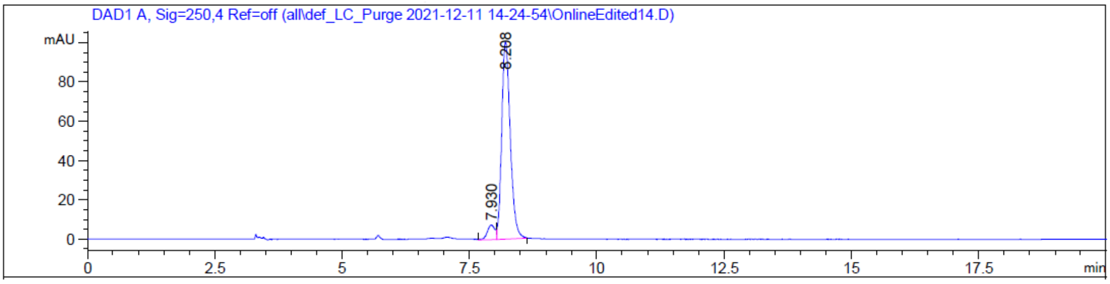

Peak #	RetTime [min]	Type	Width [min]	Area [mAU*s]	Height [mAU]	Area %
1	7.930	MF R	0.1784	79.69164	7.44545	6.3741
2	8.208	FM R	0.1938	1170.54846	100.67591	93.6259

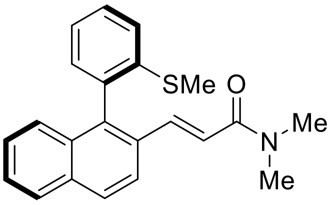

(*E*)-*N*,*N*-dimethyl-3-(1-(2-(methylthio)phenyl)naphthalen-2-yl)acrylamide (5h)

The general procedure was followed using methyl(2-(naphthalen-1-yl)phenyl)sulfane (25.1 mg, 0.10 mmol), *N*,*N*-dimethylacrylamide (32 µL, 0.30 mmol) at 65 °C for 48 h. Purification by column chromatography on silica gel (*n*-hexane/EtOAc: 1/1) yielded **5h** (18.3 mg, 53%) as a white solid. ^{1}H NMR (400 MHz, $CDCl_3$): δ = 7.92 – 7.84 (m, 2H), 7.82 (d, J = 8.8 Hz, 1H), 7.51 – 7.40 (m, 3H), 7.40 – 7.31 (m, 3H), 7.31 – 7.23 (m, 1H), 7.15 (dd, J = 7.5, 1.5 Hz, 1H), 6.84 (d, J = 15.5 Hz, 1H), 3.09 (s, 3H), 2.99 (s, 3H), 2.29 (s, 3H). ^{13}C NMR (101 MHz, $CDCl_3$): δ = 166.7 (C_q), 140.3 (CH), 139.0 (C_q), 138.6 (C_q), 135.9 (C_q), 133.7 (C_q), 132.6 (C_q), 131.3 (C_q), 130.8 (CH), 128.7 (CH), 128.4 (CH), 127.9 (CH), 126.7 (CH), 126.6 (CH), 126.6 (CH), 124.8 (CH), 123.3 (CH), 119.0 (CH), 37.4 (CH_3), 35.7 (CH_3), 15.4 (CH_3). IR (ATR): 3054, 2921, 2857, 1646, 1605, 1431, 1407, 1261, 1139, 975, 755 cm^{-1}. MS (ESI) *m*/*z* (relative intensity): 370 (100) $[M + Na]^+$, 348 (50) $[M + H]^+$. HR-MS (ESI): *m*/*z* calcd. for $[C_{22}H_{21}NOS + Na]^+$ 370.1236, found 370.1224. $[\alpha]_D^{20}$ = -58.0 (c = 0.1, $CHCl_3$). HPLC separation (Chiralpak® IB-3, *n*-hexane/*i*-PrOH 80:20, 1.0 mL/min, detection at 250 nm): t_r (major) = 11.6 min, t_r (minor) = 13.2 min, 90% ee.

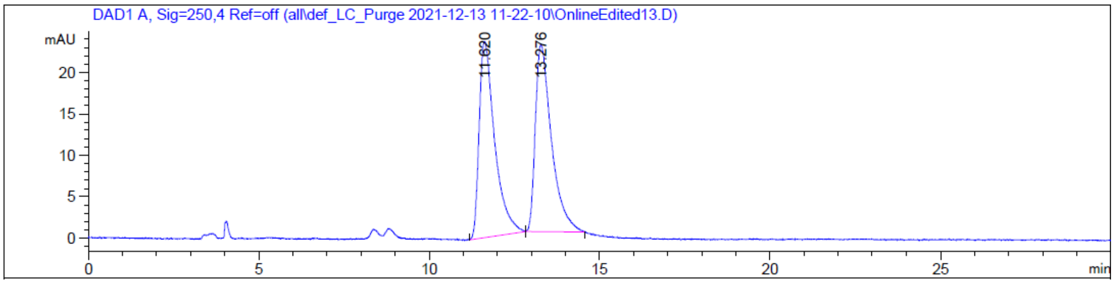

Peak #	RetTime [min]	Type	Width [min]	Area [mAU*s]	Height [mAU]	Area %
1	11.620	BB	0.4140	748.98108	23.67834	50.1360
2	13.276	BB	0.3904	744.91742	22.55124	49.8640

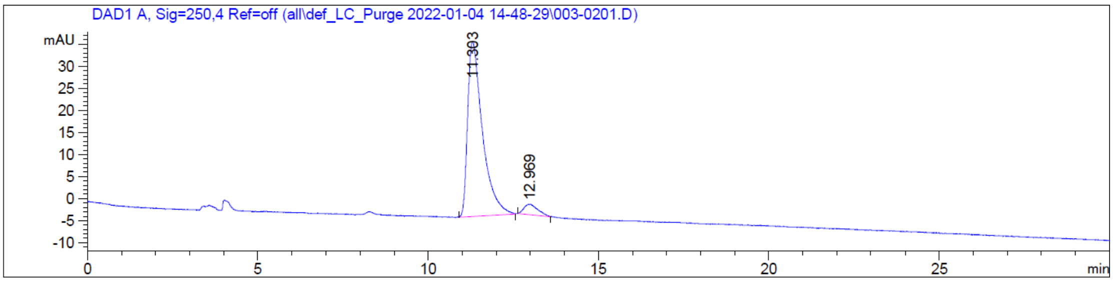

Peak #	RetTime [min]	Type	Width [min]	Area [mAU*s]	Height [mAU]	Area %
1	11.303	BB	0.3984	1193.78955	39.63005	94.6501
2	12.969	BV R	0.3387	67.47704	2.35218	5.3499

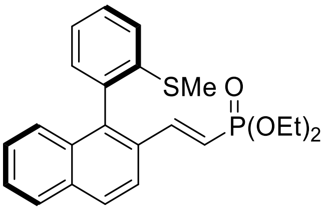

Diethyl (*E*)-(2-(1-(2-(methylthio)phenyl)naphthalen-2-yl)vinyl)phosphonate (5i)

The general procedure was followed using methyl(2-(naphthalen-1-yl)phenyl)sulfane (25.1 mg, 0.10 mmol), diethyl vinylphosphonate (46 µL, 0.30 mmol) at 65 °C for 48 h. Purification by column chromatography on silica gel (*n*-hexane/EtOAc: 1/1) yielded **5i** (30.0 mg, 73%) as a colorless oil. ^{1}H NMR (400 MHz, $CDCl_3$): δ = 7.89 (s, 1H), 7.89 – 7.83 (m, 1H), 7.79 (d, J = 8.8 Hz, 1H), 7.53 – 7.44 (m, 2H), 7.41 – 7.32 (m, 3H), 7.31 – 7.19 (m, 2H), 7.17 – 7.09 (m, 1H), 6.29 (dd, J = 18.6, 17.6 Hz, 1H), 4.07 – 3.95 (m, 4H), 2.28 (s, 3H), 1.23 (q, J = 6.9 Hz, 6H). ^{13}C NMR (101 MHz, $CDCl_3$): δ = 146.1 (CH, d, J = 7.2 Hz), 139.2 (C_q), 138.7 (C_q), 135.5 (C_q), 134.0 (C_q), 132.3 (C_q), 130.9

(CH), 130.8 (C_q, d, J = 22.9 Hz), 128.8 (CH), 128.6 (CH), 128.0 (CH), 127.0 (CH), 126.8 (CH), 126.7 (CH), 124.6 (CH), 124.6 (CH), 122.4 (CH), 115.2 (d, J = 191.0 Hz), 61.8 (CH_2, d, J = 5.5 Hz), 16.2 (CH_3), 16.2 (CH_3), 15.3 (CH_3). ^{31}P NMR (162 MHz, $CDCl_3$: δ = 19.2. IR (ATR): 3056, 2981, 2923, 1609, 1434, 1389, 1249, 1050, 1025, 963, 752 cm^{-1}. MS (ESI) m/z (relative intensity): 435 (100) [M + Na]$^+$, 413 (33) [M + H]$^+$. HR-MS (ESI): m/z calcd. for [$C_{23}H_{25}O_3PS$ + Na]$^+$ 435.1154, found 435.1143. $[\alpha]^{20}_{D}$ = -69.4 (c = 0.65, $CHCl_3$). HPLC separation (Chiralpak® IB-3, n-hexane/i-PrOH 83:17, 1.0 mL/min, detection at 250 nm): t_r (major) = 6.9 min, t_r (minor) = 7.5 min, 99% ee.

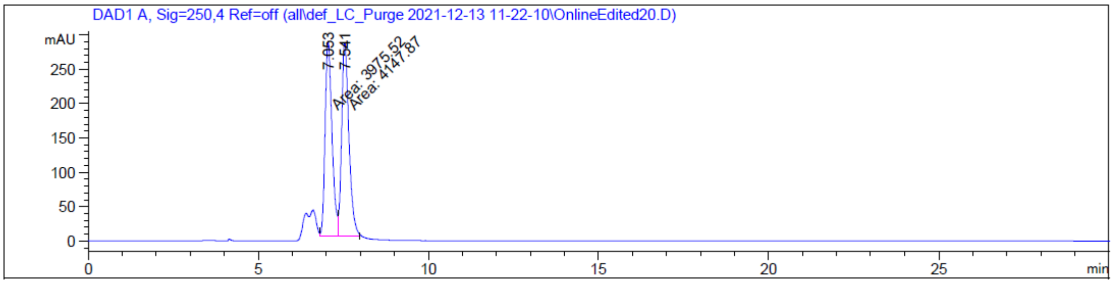

Peak #	RetTime [min]	Type	Width [min]	Area [mAU*s]	Height [mAU]	Area %
1	7.053	MF	0.2344	3975.51855	282.63440	48.9392
2	7.541	FM	0.2461	4147.86963	280.94327	51.0608

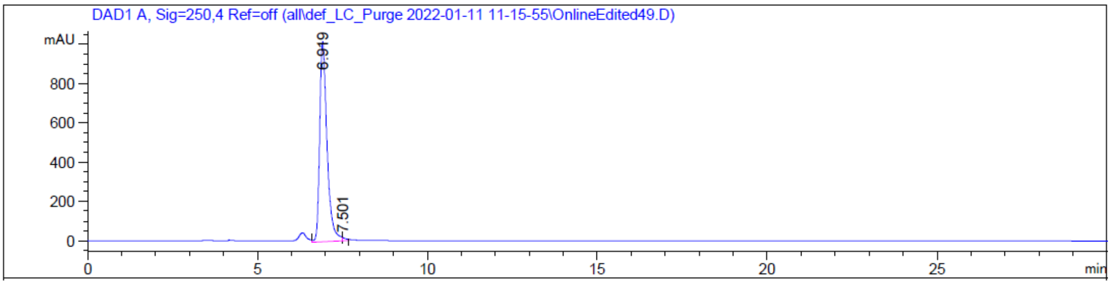

Peak #	RetTime [min]	Type	Width [min]	Area [mAU*s]	Height [mAU]	Area %
1	6.919	MM R	0.2511	1.53634e4	1019.79462	99.2445
2	7.501	FM R	0.1109	116.95916	17.57157	0.7555

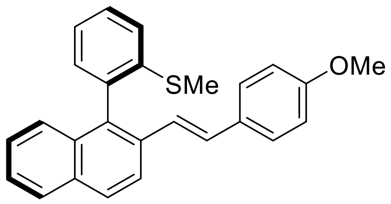

(*E*)-(2-(2-(4-methoxystyryl)naphthalen-1-yl)phenyl)(methyl)sulfane (5j)

The general procedure was followed using methyl(2-(naphthalen-1-yl)phenyl)sulfane (12.5 mg, 0.050 mmol), 1-methoxy-4-vinylbenzene (20.1 mg, 0.150 mmol), $Pd(OAc)_2$ (1.2 mg, 0.0050 mmol), **L8** (5.6 mg, 0.00750 mmol), *n*-Bu_2O (1.0 mL) under oxygen atmosphere (O_2 balloon) at 65 °C for 48 h. Purification by column chromatography on silica gel (*n*-hexane/EtOAc: 40/1) yielded **5j** (9.6 mg, 50%) as a white solid. ^{1}H NMR (400 MHz, $CDCl_3$): δ = 7.90 (d, J = 8.7 Hz, 1H), 7.86 – 7.78 (m, 2H), 7.45 (ddt, J = 8.1, 7.4, 1.3 Hz, 1H), 7.41 – 7.36 (m, 1H), 7.35 – 7.23 (m, 4H), 7.23 – 7.17 (m, 2H), 7.15 (dt, J = 7.5, 1.1 Hz, 1H), 7.09 (d, J = 16.3 Hz, 1H), 6.80 – 6.75 (m, 2H), 6.70 (d, J = 16.3 Hz, 1H), 3.74 (d, J = 0.9 Hz, 3H), 2.25 (s, 3H). ^{13}C NMR (101 MHz, $CDCl_3$): δ = 159.2 (C_q), 139.4 (C_q), 136.8 (C_q), 135.7 (C_q), 133.2 (C_q), 132.8 (C_q), 132.7 (C_q), 131.2 (CH), 130.4 (C_q), 129.1 (CH), 128.4 (CH), 128.3 (CH), 128.0 (CH), 127.8 (CH), 126.3 (CH), 126.2 (CH), 125.6 (CH), 124.9 (CH), 124.6 (CH), 124.5 (CH), 122.5 (CH), 114.0 (CH), 55.3 (CH_3), 15.4 (CH_3). MS (ESI) *m*/*z* (relative intensity): 383 (100) $[M + H]^+$, 405 (50) $[M + Na]^+$. HR-MS (ESI): *m*/*z* calcd. for $[C_{26}H_{22}OS + H]^+$ 383.1464, found 383.1452. $[\alpha]_D^{20}$ = -139.5 (c = 0.2, $CHCl_3$). HPLC separation (Chiralpak® IB-3, *n*-hexane/*i*-PrOH 99.5:0.5, 1.0 mL/min, detection at 250 nm): t_r (major) = 17.1 min, t_r (minor) = 18.9 min, 94% ee.

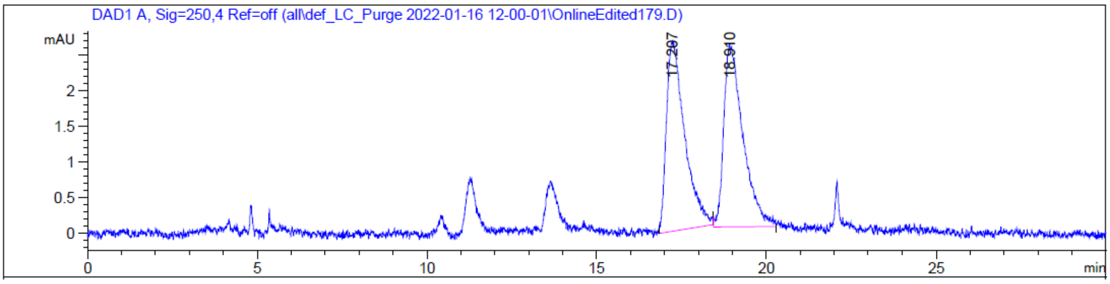

Peak #	RetTime [min]	Type	Width [min]	Area [mAU*s]	Height [mAU]	Area %
1	17.207	PM R	0.6236	99.80242	2.66723	49.7758
2	18.910	MP R	0.6534	100.70139	2.56877	50.2242

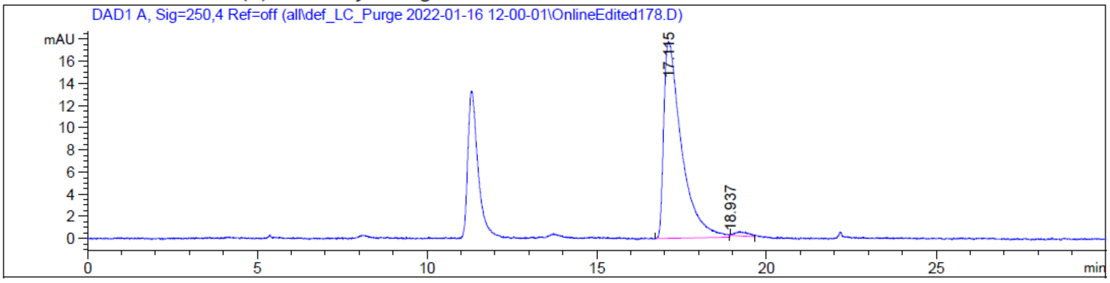

Peak #	RetTime [min]	Type	Width [min]	Area [mAU*s]	Height [mAU]	Area %
1	17.115	PM R	0.6052	645.85883	17.78571	98.5623
2	18.937	MM R	0.4362	9.42072	7.97025e-2	1.4377

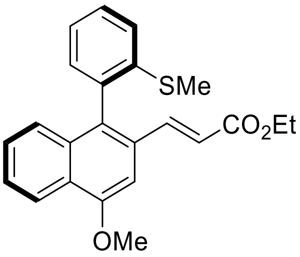

Ethyl (*E*)-3-(4-methoxy-1-(2-(methylthio)phenyl)naphthalen-2-yl)acrylate (5k)

The general procedure was followed using (2-(4-methoxynaphthalen-1-yl)phenyl)(methyl)sulfane (28.1 mg, 0.10 mmol), ethyl acrylate (32 µL, 0.30 mmol) at 65 °C for 48 h. Purification by column chromatography on silica gel (*n*-hexane/EtOAc: 10/1) yielded **5k** (28.7 mg, 76%) as a white solid. ^{1}H NMR (400 MHz, $CDCl_3$): δ = 8.31 (ddd, J = 8.4, 1.4, 0.7 Hz, 1H), 7.54 – 7.44 (m, 3H), 7.43 – 7.37 (m, 1H), 7.34 (dd, J = 8.1, 1.2 Hz, 1H), 7.31 – 7.25 (m, 2H), 7.14 (dd, J = 7.5, 1.5 Hz, 1H), 7.11 (s, 1H), 6.47 (d, J = 15.9 Hz, 1H), 4.19 (q, J = 7.1 Hz, 2H), 4.09 (s, 3H), 2.30 (s, 3H), 1.28 (t, J = 7.1 Hz, 3H). ^{13}C NMR (101 MHz, $CDCl_3$): δ = 166.9 (C_q), 155.4 (C_q), 143.4 (CH), 139.7 (C_q), 135.6 (C_q), 133.4 (C_q), 132.7 (C_q), 131.5 (CH), 130.5 (C_q), 128.8 (CH), 127.3 (CH), 126.8 (C_q), 126.7 (CH), 126.5 (CH), 124.6 (CH), 124.5 (CH), 122.2 (CH), 118.7 (CH), 99.8 (CH), 60.3 (CH_2), 55.5 (CH_3), 15.4 (CH_3), 14.3 (CH_3). IR (ATR): 2978, 2922, 1709, 1630, 1593, 1454, 1376, 1274, 1182, 1109, 764 cm^{-1}. MS (ESI) *m*/*z* (relative intensity): 401 (100) $[M + Na]^+$, 779 (25) $[2M + Na]^+$. HR-MS (ESI): *m*/*z* calcd. for $[C_{23}H_{22}O_3S + Na]^+$ 401.1182, found 401.1171. $[\alpha]_D^{20}$ = -96.6 (c = 0.5, $CHCl_3$). HPLC separation (Chiralpak® AD-3, *n*-hexane/*i*-PrOH 95:5, 1.0 mL/min, detection at 250 nm): t_r (major) = 7.8 min, t_r (minor) = 8.6 min, 96% ee.

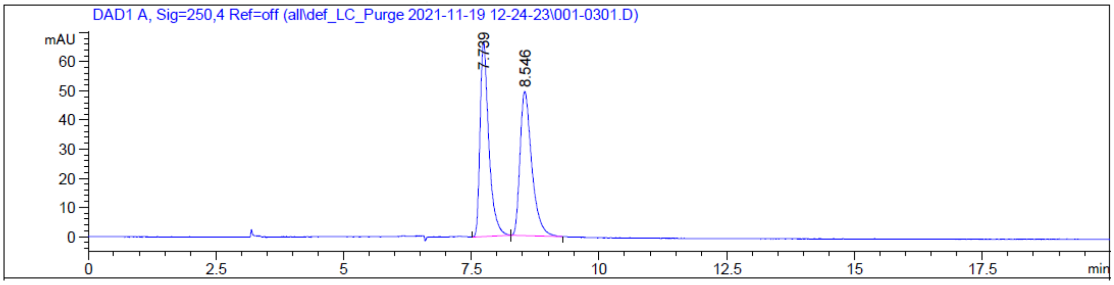

Peak #	RetTime [min]	Type	Width [min]	Area [mAU*s]	Height [mAU]	Area %
1	7.739	BB	0.1776	795.53827	66.94102	50.0946
2	8.546	BB	0.2387	792.53394	49.53605	49.9054

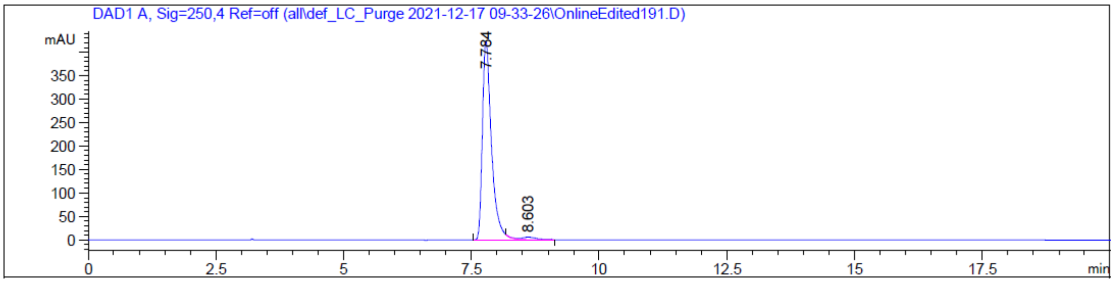

Peak #	RetTime [min]	Type	Width [min]	Area [mAU*s]	Height [mAU]	Area %
1	7.784	BV R	0.1857	5254.48877	423.74756	97.9430
2	8.603	VB E	0.2359	110.35628	5.59948	2.0570

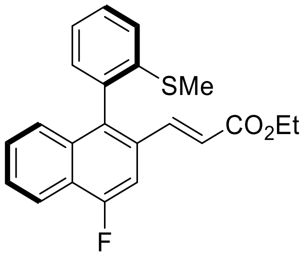

Ethyl (*E*)-3-(4-fluoro-1-(2-(methylthio)phenyl)naphthalen-2-yl)acrylate (5l)

The general procedure was followed using (2-(4-fluoronaphthalen-1-yl)phenyl)(methyl)sulfane (26.9 mg, 0.10 mmol), ethyl acrylate (32 μL, 0.30 mmol) at 65 °C for 48 h. Purification by column chromatography on silica gel (*n*-hexane/EtOAc: 15/1) yielded **5l** (18.8 mg, 51%) as a white solid. ^{1}H NMR (400 MHz, $CDCl_3$): δ = 8.17 – 8.10 (m, 1H), 7.56 (ddd, J = 8.2, 6.8, 1.2 Hz, 1H), 7.53 – 7.40 (m, 4H), 7.38 – 7.32 (m, 2H), 7.29 (td, J = 7.4, 1.2 Hz, 1H), 7.16 – 7.09 (m, 1H), 6.43 (d, J = 15.9 Hz, 1H), 4.18 (q, J = 7.2 Hz, 2H), 2.31 (s, 3H), 1.27 (t, J = 7.1 Hz, 3H). ^{13}C NMR (101 MHz,

$CDCl_3$): δ = 166.6 (C_q), 160.0 (C_q), 157.5 (C_q), 142.0 (CH, d, J = 2.8 Hz), 139.4 (C_q), 135.6 (C_q, d, J = 4.0 Hz), 134.8 (C_q), 133.9 (C_q, d, J = 5.3 Hz), 131.2 (CH), 130.9 (C_q, d, J = 8.1 Hz), 129.0 (CH), 127.7 (CH), 127.3 (CH, d, J = 1.9 Hz), 126.9 (CH, d, J = 2.9 Hz), 124.7 (CH, d, J = 1.6 Hz), 120.7 (CH, d, J = 5.1 Hz), 119.8 (CH), 106.1 (CH), 105.9 (CH), 60.4 (CH_2), 15.3 (CH_3), 14.2 (CH_3). ^{19}F NMR (377 MHz, $CDCl_3$): δ = -122.58 (dt, J = 11.6, 2.4 Hz). IR (ATR): 3062, 2981, 2923, 1712, 1631, 1377, 1298, 1261, 1180, 1065, 764 cm^{-1}. MS (ESI) m/z (relative intensity): 389 (100) $[M + Na]^+$, 755 (20) $[2M + Na]^+$. HR-MS (ESI): m/z calcd. for $[C_{22}H_{19}FO_2S + Na]^+$ 389.0982, found 389.0978. $[\alpha]_D^{20}$ = -83.7 (c = 0.3, $CHCl_3$). HPLC separation (Chiralpak® IF-3, n-hexane/i-PrOH 98:2, 1.0 mL/min, detection at 250 nm): t_r (major) = 7.3 min, t_r (minor) = 7.0 min, 95% ee.

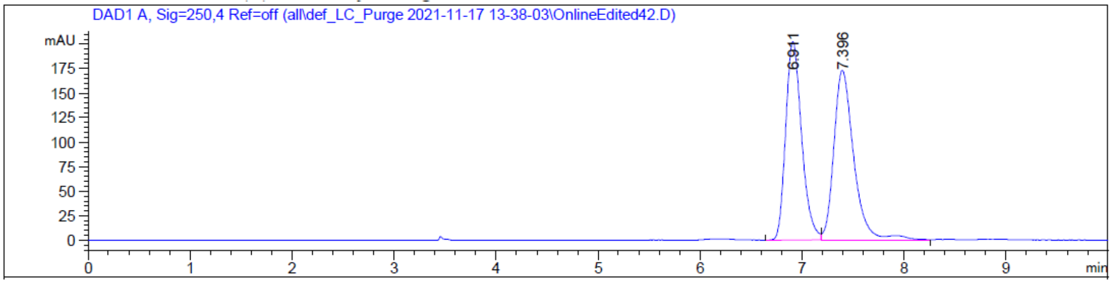

Peak #	RetTime [min]	Type	Width [min]	Area [mAU*s]	Height [mAU]	Area %
1	6.911	BV	0.1661	2204.24341	202.36226	48.3887
2	7.396	VV R	0.2029	2351.04004	172.94597	51.6113

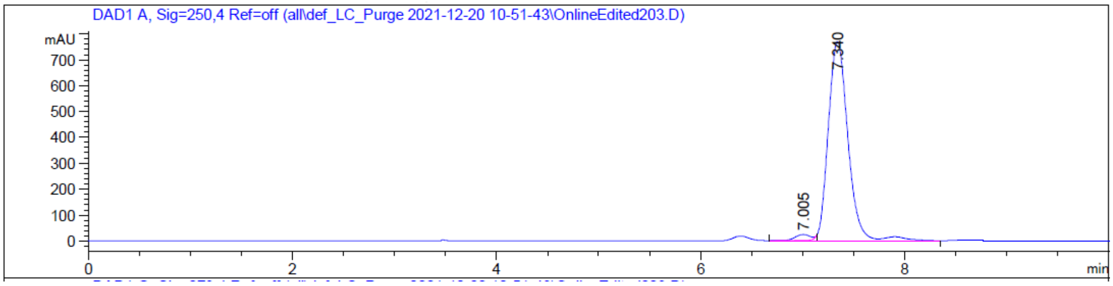

Peak #	RetTime [min]	Type	Width [min]	Area [mAU*s]	Height [mAU]	Area %
1	7.005	BV E	0.1564	239.09802	23.36212	2.2569
2	7.340	VV R	0.2054	1.03550e4	769.45044	97.7431

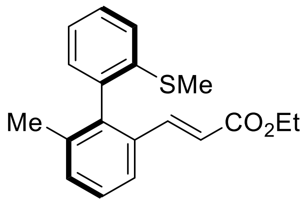

Ethyl (*E*)-3-(6-methyl-2'-(methylthio)-[1,1'-biphenyl]-2-yl)acrylate (5m)

The general procedure was followed using methyl(2'-methyl-[1,1'-biphenyl]-2-yl)sulfane (21.5 mg, 0.10 mmol), ethyl acrylate (32 µL, 0.30 mmol) at 65 °C for 48 h. Purification by column chromatography on silica gel (*n*-hexane/EtOAc: 15/1) yielded **5m** (28.1 mg, 90%) as a colorless oil. ^{1}H NMR (400 MHz, $CDCl_3$): δ = 7.58 (dd, J = 5.6, 3.6 Hz, 1H), 7.38 (td, J = 7.7, 1.5 Hz, 1H), 7.34 – 7.17 (m, 5H), 7.05 – 6.95 (m, 1H), 6.30 (d, J = 15.9 Hz, 1H), 4.13 (q, J = 7.1 Hz, 2H), 2.35 (s, 3H), 2.03 (s, 3H), 1.23 (t, J = 7.2 Hz, 3H). ^{13}C NMR (101 MHz, $CDCl_3$): δ = 166.9 (C_q), 143.2 (CH), 140.6 (C_q), 137.9 (C_q), 137.6 (C_q), 136.8 (C_q), 133.4 (C_q), 131.5 (CH), 129.6 (CH), 128.4 (CH), 128.0 (CH), 124.7 (CH), 124.4 (CH), 123.6 (CH), 118.8 (CH), 60.1 (CH_2), 20.1 (CH_3), 15.1 (CH_3), 14.2 (CH_3). IR (ATR): 3062, 2980, 2922, 1712, 1633, 1433, 1311, 1221, 1179, 1037, 750 cm^{-1}. MS (ESI) m/z (relative intensity): 335 (100) $[M + Na]^+$. HR-MS (ESI): m/z calcd. for $[C_{19}H_{20}O_2S + Na]^+$ 335.1076, found 335.1070. $[\alpha]_D^{20}$ = -94.9 (c = 0.7, $CHCl_3$). HPLC separation (Chiralpak® IC-3, *n*-hexane/*i*-PrOH 90:10, 1.0 mL/min, detection at 250 nm): t_r (major) = 7.5 min, t_r (minor) = 14.1 min, 94% ee.

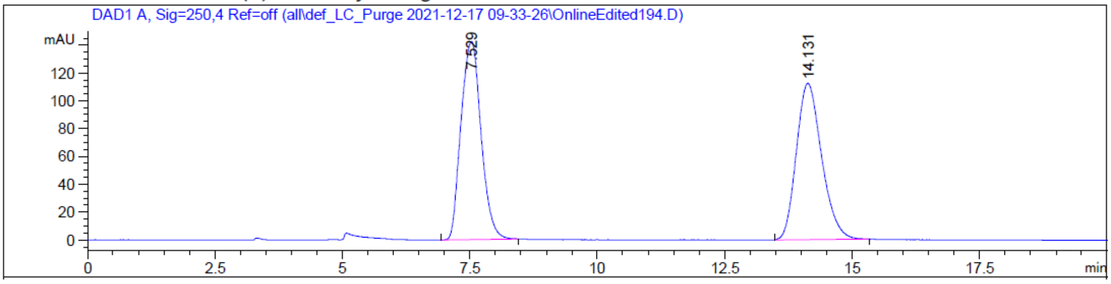

Peak #	RetTime [min]	Type	Width [min]	Area [mAU*s]	Height [mAU]	Area %
1	7.529	BB	0.4376	3889.13721	142.41327	50.0637
2	14.131	BV R	0.4859	3879.23511	112.31825	49.9363

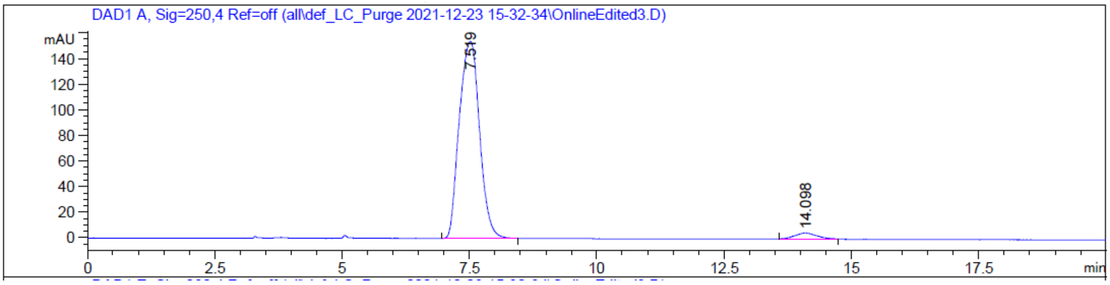

Peak #	RetTime [min]	Type	Width [min]	Area [mAU*s]	Height [mAU]	Area %
1	7.519	BB	0.4073	4252.60791	153.92145	96.8517
2	14.098	BB	0.3566	138.23807	4.58633	3.1483

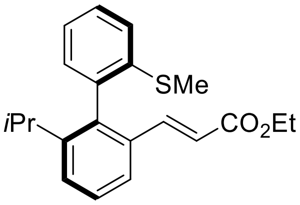

Ethyl (*E*)-3-(6-isopropyl-2'-(methylthio)-[1,1'-biphenyl]-2-yl)acrylate (5n)

The general procedure was followed using (2'-isopropyl-[1,1'-biphenyl]-2-yl)(methyl)sulfane (24.3 mg, 0.10 mmol), ethyl acrylate (32 µL, 0.30 mmol) at 65 °C for 48 h. Purification by column chromatography on silica gel (*n*-hexane/EtOAc: 15/1) yielded **5n** (31.2 mg, 92%) as a colorless oil. ^{1}H NMR (400 MHz, $CDCl_3$): δ = 7.58 (dd, J = 7.0, 1.9 Hz, 1H), 7.47 – 7.35 (m, 3H), 7.31 – 7.18 (m, 3H), 7.03 (dd, J = 7.5, 1.5 Hz, 1H), 6.29 (d, J = 16.0 Hz, 1H), 4.14 (q, J = 7.1 Hz, 2H), 2.64 – 2.51 (m, 1H), 2.36 (s, 3H), 1.28 – 1.16 (m, 6H), 1.05 (d, J = 6.8 Hz, 3H). ^{13}C NMR (101 MHz, $CDCl_3$): δ = 166.7 (C_q), 148.3 (C_q), 143.6 (CH), 139.2 (C_q), 138.6 (C_q), 136.5 (C_q), 133.3 (C_q), 130.1 (CH), 128.4 (CH), 128.4 (CH), 127.3 (CH), 124.4 (CH), 124.2 (CH), 123.6 (CH), 118.7 (CH), 60.1 (CH_2), 30.2 (CH_3), 24.6 (CH_3), 23.3 (CH_3), 15.2 (CH_3), 14.2 (CH). IR (ATR): 3061, 3961, 2923, 2867, 1713, 1633, 1435, 1309, 1172, 1037, 752 cm^{-1}. MS (ESI) m/z (relative intensity): 363 (100) $[M + Na]^{+}$. HR-MS (ESI): m/z calcd. for $[C_{21}H_{24}O_2S + Na]^{+}$ 363.1389, found 363.1387. $[\alpha]^{20}_{D}$ = -76.4 (c = 0.45, $CHCl_3$). HPLC separation (Chiralpak® IC-3, *n*-hexane/*i*-PrOH 80:20, 1.0 mL/min, detection at 250 nm): t_r (major) = 5.0 min, t_r (minor) = 8.9 min, 93% ee.

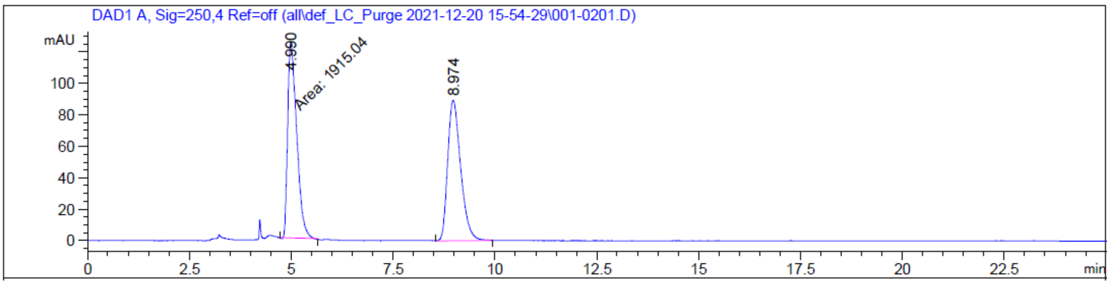

Peak #	RetTime [min]	Type	Width [min]	Area [mAU*s]	Height [mAU]	Area %
1	4.990	MM	0.2563	1915.03906	124.53154	49.3056
2	8.974	BV R	0.3337	1968.97693	89.15656	50.6944

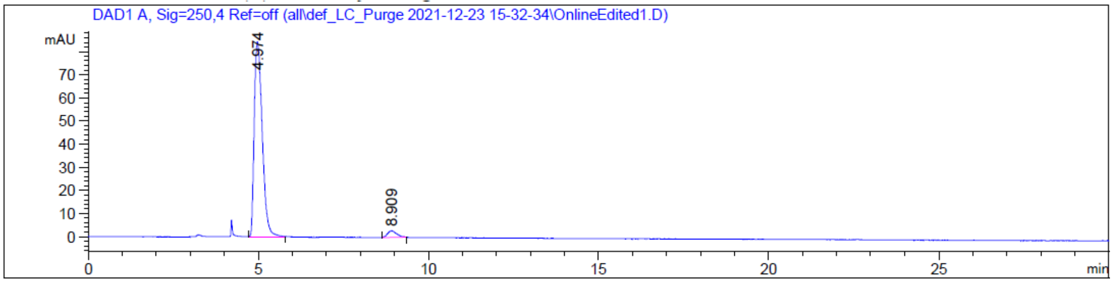

Peak #	RetTime [min]	Type	Width [min]	Area [mAU*s]	Height [mAU]	Area %
1	4.974	BB	0.2542	1376.55090	84.45389	96.2801
2	8.909	BB	0.2155	53.18546	2.93363	3.7199

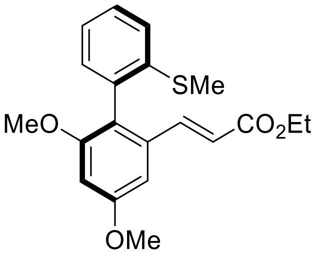

Ethyl (*E*)-3-(4,6-dimethoxy-2'-(methylthio)-[1,1'-biphenyl]-2-yl)acrylate (5o)

The general procedure was followed using (2',4'-dimethoxy-[1,1'-biphenyl]-2-yl)(methyl)sulfane (26.1 mg, 0.10 mmol), ethyl acrylate (32 μL, 0.30 mmol) at 65 °C for 48 h. Purification by column chromatography on silica gel (*n*-hexane/EtOAc: 8/1) yielded **5o** (22.7 mg, 63%) as a white solid. ^{1}H NMR (400 MHz, $CDCl_3$): δ = 7.37 (td, J = 7.6, 1.4 Hz, 1H), 7.30 (s, 1H), 7.29 – 7.25 (m, 1H), 7.20 (td, J = 7.4, 1.3 Hz, 1H), 7.05 (dd, J = 7.5, 1.5 Hz, 1H), 6.84 (d, J = 2.3 Hz, 1H), 6.60 (d, J = 2.3 Hz, 1H), 6.32 (d, J = 15.9 Hz, 1H), 4.15 (q, J = 7.1 Hz, 2H), 3.89 (s, 3H), 3.72 (s, 3H), 2.35 (s, 3H),

1.25 (t, J = 7.1 Hz, 3H). ^{13}C NMR (101 MHz, $CDCl_3$): δ = 166.7 (C_q), 160.3 (C_q), 158.5 (C_q), 142.9 (CH), 139.1 (C_q), 135.0 (C_q), 134.2 (C_q), 131.1 (CH), 128.4 (CH), 125.0 (CH), 124.6 (CH), 123.6 (C_q), 119.3 (CH), 101.3 (CH), 100.7 (CH), 60.3 (CH_2), 56.0 (CH_3), 55.4 (CH_3), 15.6 (CH_3), 14.2 (CH_3). IR (ATR): 2958, 2926, 2837, 1711, 1635, 1601, 1573, 1456, 1290, 1267, 1036 cm^{-1}. MS (ESI) m/z (relative intensity): 381 (100) $[M + Na]^+$, 359 (10) $[M + H]^+$. HR-MS (ESI): m/z calcd. for $[C_{20}H_{22}O_4S + Na]^+$ 381.1131, found 381.1119. $[\alpha]_D^{20}$ = -66.6 (c = 0.35, $CHCl_3$). HPLC separation (Chiralpak® AD-3, n-hexane/i-PrOH 90:10, 1.0 mL/min, detection at 250 nm): t_r (major) = 10.1 min, t_r (minor) = 7.1 min, 95% ee.

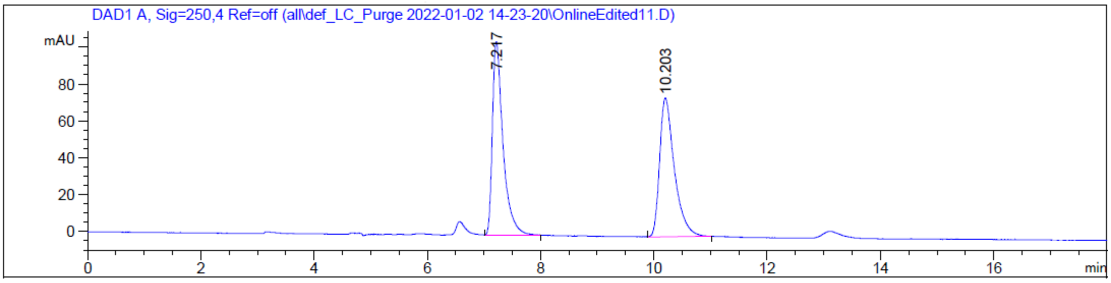

Peak #	RetTime [min]	Type	Width [min]	Area [mAU*s]	Height [mAU]	Area %
1	7.217	BV R	0.1895	1361.45618	104.85366	50.1225
2	10.203	BB	0.2641	1354.79968	75.22623	49.8775

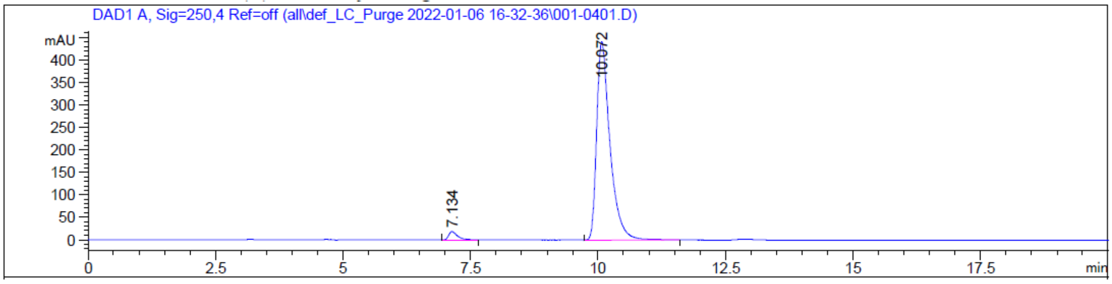

Peak #	RetTime [min]	Type	Width [min]	Area [mAU*s]	Height [mAU]	Area %
1	7.134	BB	0.1723	225.96432	18.65309	2.7459
2	10.072	BB	0.2666	8003.15039	441.33231	97.2541

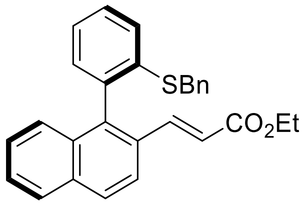

Ethyl (*E*)-3-(1-(2-(benzylthio)phenyl)naphthalen-2-yl)acrylate (5p)

The general procedure was followed using benzyl(2-(naphthalen-1-yl)phenyl)sulfane (32.7 mg, 0.10 mmol), ethyl acrylate (32 µL, 0.30 mmol) at 65 °C for 48 h. Purification by column chromatography on silica gel (*n*-hexane/EtOAc: 15/1) yielded **5p** (25.4 mg, 60%) as a colorless oil. ^{1}H NMR (400 MHz, $CDCl_3$): δ = 7.98 – 7.80 (m, 3H), 7.60 – 7.48 (m, 2H), 7.48 – 7.29 (m, 5H), 7.25 – 7.13 (m, 6H), 6.51 (d, J = 15.9 Hz, 1H), 4.22 (q, J = 7.1 Hz, 2H), 3.97 (s, 2H), 1.29 (t, J = 7.1 Hz, 3H). ^{13}C NMR (101 MHz, $CDCl_3$): δ = 166.8 (C_q), 142.9, 139.7 (C_q), 137.3 (C_q), 137.1 (C_q), 136.7 (C_q), 134.0 (C_q), 132.6 (C_q), 131.2 (CH), 130.3 (C_q), 128.7 (CH), 128.7 (CH), 128.5 (CH), 128.3 (CH), 128.1 (CH), 127.8 (CH), 127.0 (CH), 127.0 (CH), 126.9 (CH), 126.6 (CH), 125.7 (CH), 122.6 (CH), 119.1 (CH), 60.3 (CH_2), 37.6 (CH_2), 14.2 (CH_3). IR (ATR): 3056, 2923, 2852, 1710, 1629, 1297, 1258, 1177, 1154, 1037, 753 cm^{-1}. MS (ESI) m/z (relative intensity): 447 (100) $[M + Na]^+$. HR-MS (ESI): m/z calcd. for $[C_{28}H_{24}O_2S + Na]^+$ 447.1389, found 447.1382. $[\alpha]_D^{20}$ = -86.3 (c = 0.4, $CHCl_3$). HPLC separation (Chiralpak® AD-3, *n*-hexane/*i*-PrOH 95:5, 1.0 mL/min, detection at 250 nm): t_r (major) = 10.7 min, t_r (minor) = 15.1 min, 92% ee.

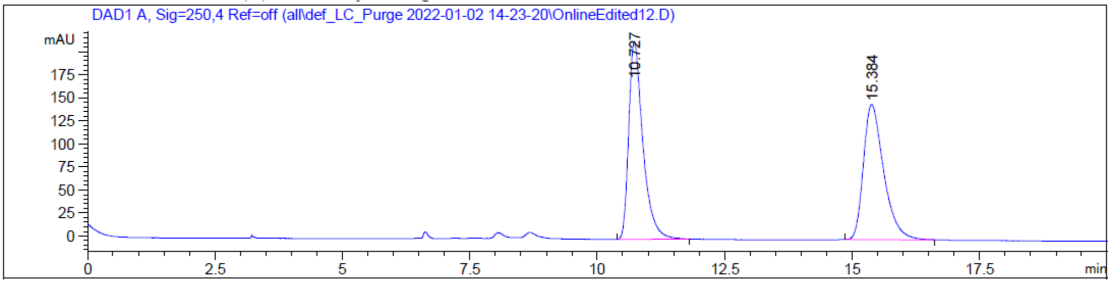

Peak #	RetTime [min]	Type	Width [min]	Area [mAU*s]	Height [mAU]	Area %
1	10.727	BB	0.2851	4139.80859	214.56870	50.4512
2	15.384	BV R	0.4146	4065.75537	146.58340	49.5488

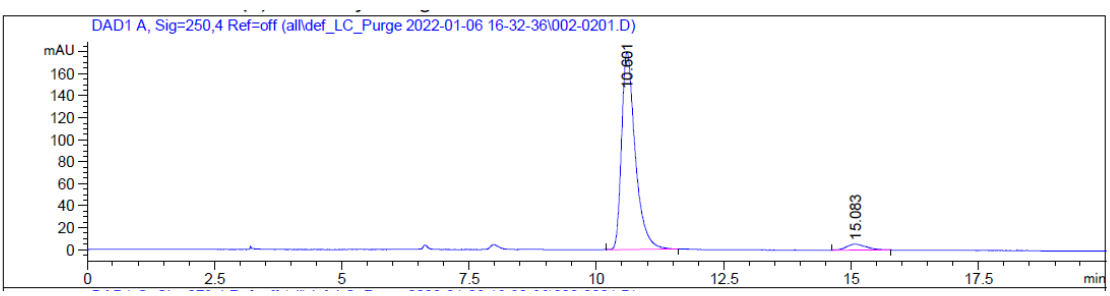

Peak #	RetTime [min]	Type	Width [min]	Area [mAU*s]	Height [mAU]	Area %
1	10.601	BB	0.2789	3345.12329	179.11914	95.8696
2	15.083	BB	0.3228	144.11821	5.30735	4.1304

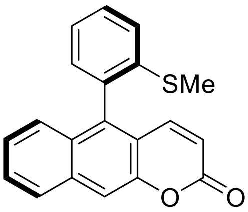

(*S*)-5-(2-(methylthio)phenyl)-2H-benzo[g]chromen-2-one (5q)

The general procedure was followed using 4-(2-(methylthio)phenyl)naphthalen-2-yl acrylate (32.0 mg, 0.10 mmol) under oxygen atmosphere (O_2 balloon) at 65 °C for 48 h. Purification by column chromatography on silica gel (*n*-hexane/EtOAc: 5/1) yielded **5q** (11.4 mg, 36%) as a yellow solid. ^{1}H NMR (400 MHz, $CDCl_3$): δ = 7.91 (d, J = 8.3 Hz, 1H), 7.78 (s, 1H), 7.59 – 7.50 (m, 2H), 7.47 – 7.30 (m, 5H), 7.19 (dt, J = 7.4, 1.2 Hz, 1H), 6.34 (dd, J = 9.9, 0.9 Hz, 1H), 2.31 (d, J = 0.9 Hz, 3H). ^{13}C NMR (101 MHz, $CDCl_3$): δ = 160.5 (C_q), 150.2 (C_q), 141.9 (CH), 139.4 (C_q), 137.8 (C_q), 134.6 (C_q), 133.6 (C_q), 131.0 (CH), 129.4 (CH), 129.1 (C_q), 128.2 (CH), 127.8 (CH), 126.7 (CH), 125.9 (CH), 124.6 (CH), 124.6 (CH), 117.5 (C_q), 117.0 (CH), 112.9 (CH), 15.2 (CH_3). IR (ATR): 3058, 2921, 1730, 1620, 1597, 1458, 1434, 1207, 1157, 1124, 749 cm^{-1}. MS (ESI) *m*/*z* (relative intensity): 659 (100) $[2M + Na]^+$, 341 (25) $[M + Na]^+$. HR-MS (ESI): *m*/*z* calcd. for $[C_{20}H_{14}O_2S + Na]^+$ 341.0607, found 341.0602. $[\alpha]_D^{20}$ = -6.0 (c = 0.1, $CHCl_3$). HPLC separation (Chiralpak® IB-3, *n*-hexane/*i*-PrOH 90:10, 1.0 mL/min, detection at 250 nm): t_r (major) = 9.7 min, t_r (minor) = 11.1 min, 95% ee.

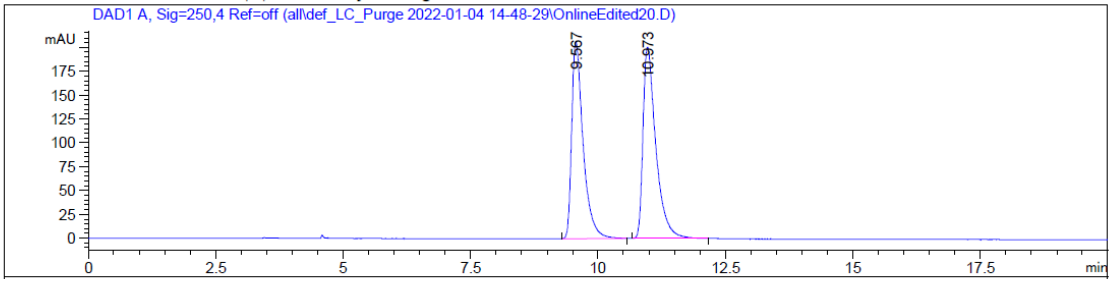

Peak #	RetTime [min]	Type	Width [min]	Area [mAU*s]	Height [mAU]	Area %
1	9.567	BB	0.2320	3211.69141	206.95892	47.7167
2	10.973	BB	0.2599	3519.05444	200.40166	52.2833

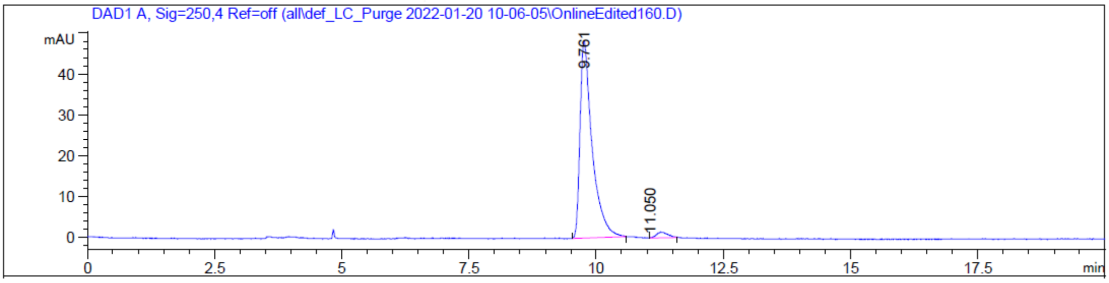

Peak #	RetTime [min]	Type	Width [min]	Area [mAU*s]	Height [mAU]	Area %
1	9.761	BB	0.2356	796.30176	48.23081	97.2660
2	11.050	MM R	0.2684	22.38261	6.09619e-2	2.7340

5. Atroposelective Palladium-Catalyzed C−H Alkynylation

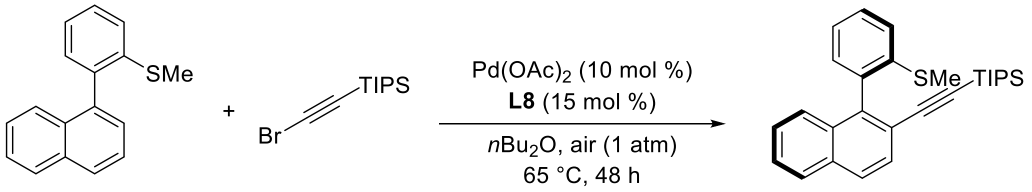

4a **6** **7a**

52%, 71% ee

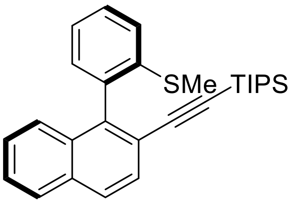

Triisopropyl((1-(2-(methylthio)phenyl)naphthalen-2-yl)ethynyl)silane (7a)

To an oven-dried 25 mL Schlenk tube was added substrate **4a** (25.1 mg, 0.10 mmol), TIPS protected alkynyl bromide **6** (78.3 mg, 0.30 mmol), $Pd(OAc)_2$ (2.3 mg, 0.010 mmol), **L8** (11.3 mg, 0.0150 mmol), *n*-Bu_2O (2.0 mL). The mixture was stirred for 48 h at 65 °C under air. Purification by column chromatography on silica gel (*n*-hexane/EtOAc: 20/1) yielded **7a** (22.4 mg, 52%) as a yellow oil. ^{1}H NMR (400 MHz, $CDCl_3$): δ = 7.86 (d, J = 8.3 Hz, 1H), 7.83 (d, J = 8.5 Hz, 1H), 7.65 (d, J = 8.5 Hz, 1H), 7.47 (ddd, J = 8.2, 5.9, 2.2 Hz, 1H), 7.43 – 7.33 (m, 4H), 7.29 – 7.20 (m, 2H), 2.29 (s, 3H), 0.95 (s, 21H). ^{13}C NMR (101 MHz, $CDCl_3$): δ = 141.7, 138.6, 138.0, 132.9, 132.0, 130.6, 128.9, 128.3, 128.0, 127.7, 126.6, 126.4, 126.2, 125.5, 124.9, 121.0, 106.2, 94.6, 18.5, 15.8, 11.1. IR (ATR): 3056, 2939, 2921, 2862, 2144, 1461, 1433, 881, 747, 671 cm^{-1}. MS (ESI) *m*/*z* (relative intensity): 431 (100) $[M + H]^+$, 453 (40) $[M + Na]^+$. HR-MS (ESI): *m*/*z* calcd. for $[C_{28}H_{34}SSi + H]^+$ 432.2223, found 431.2218. $[\alpha]_D^{20}$ = +0.6 (c = 0.65, $CHCl_3$). HPLC separation (Chiralpak® IB-3, *n*-hexane/*i*-PrOH 99.5:0.5, 1.0 mL/min, detection at 250 nm): t_r (major) = 4.3 min, t_r (minor) = 4.7 min, 71% ee.

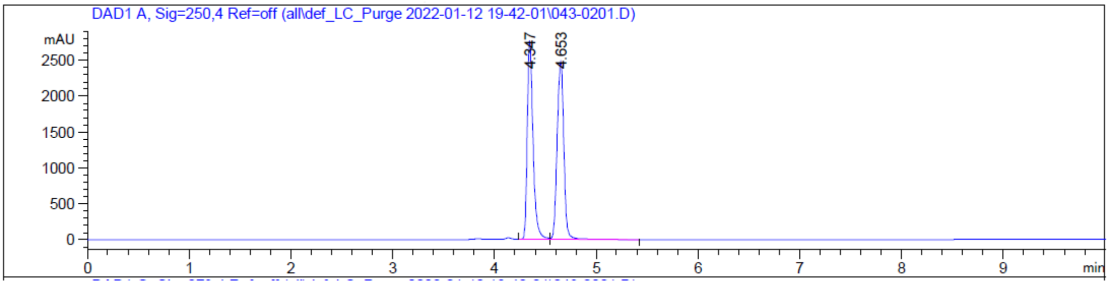

Peak #	RetTime [min]	Type	Width [min]	Area [mAU*s]	Height [mAU]	Area %
1	4.347	BV	0.0602	1.09399e4	2765.02686	49.2069
2	4.653	VB	0.0721	1.12925e4	2481.51270	50.7931

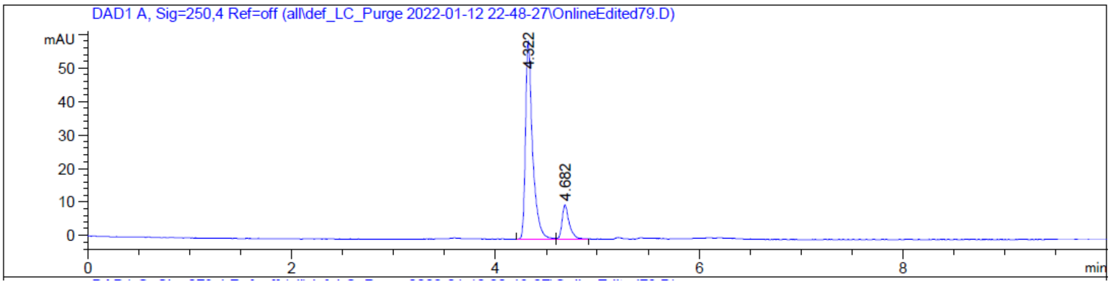

Peak #	RetTime [min]	Type	Width [min]	Area [mAU*s]	Height [mAU]	Area %
1	4.322	BV	0.0702	284.93167	59.16600	85.2550
2	4.682	VV R	0.0705	49.27941	10.18184	14.7450

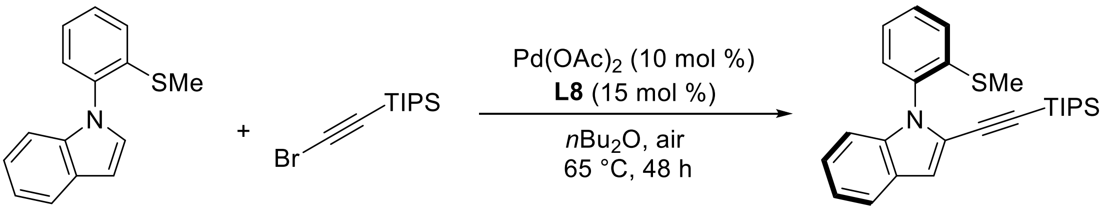

1a **6** **7b**

21%, 0% ee

To an oven-dried 25 mL Schlenk tube was added substrate **1a** (23.9 mg, 0.10 mmol), TIPS protected alkynyl bromide **6** (78.3 mg, 0.30 mmol), Pd(OAc)$_2$ (2.3 mg, 0.010 mmol), **L8** (11.3 mg, 0.0150 mmol), *n*-Bu$_2$O (2.0 mL). The mixture was stirred for 48 h at 65 °C under air. Purification by column chromatography on silica gel (*n*-hexane/EtOAc: 20/1) yielded **7b** (8.8 mg, 21%) as a colorless oil.

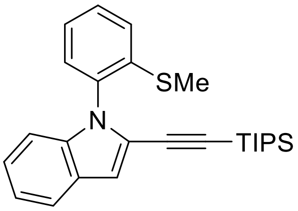

1-(2-(methylthio)phenyl)-2-((triisopropylsilyl)ethynyl)-1*H*-indole (7b)

^{1}H NMR (400 MHz, CDCl$_3$): δ = 7.72 – 7.64 (m, 1H), 7.52 – 7.43 (m, 1H), 7.42 – 7.28

(m, 3H), 7.24 – 7.17 (m, 2H), 6.99 (s, 1H), 6.95 (d, $J = 7.9$ Hz, 1H), 2.31 (s, 3H), 0.98 (s, 21H). ^{13}C NMR (101 MHz, $CDCl_3$): $\delta = 139.9$ (C_q), 137.5 (C_q), 135.3 (C_q), 130.0 (CH), 129.3 (CH), 127.0 (C_q), 126.2 (CH), 125.3 (CH), 123.5 (CH), 122.6 (C_q), 121.0 (CH), 120.7 (CH), 110.5 (CH), 108.7 (CH), 97.9 (C_q), 97.1 (C_q), 18.4 (CH_3), 15.1 (CH_3), 11.1 (CH). MS (ESI) *m/z* (relative intensity): 420 (100) $[M + H]^+$, 442 (50) $[M + Na]^+$. HR-MS (ESI): *m/z* calcd. for $[C_{26}H_{33}NSSi + H]^+$ 420.2176, found 420.2180. HPLC separation (Chiralpak® IB-3, *n*-hexane/*i*-PrOH 99.5:0.5, 1.0 mL/min, detection at 250 nm): t_r (major) = 9.4min, t_r (minor) = 10.1 min, 2% ee.

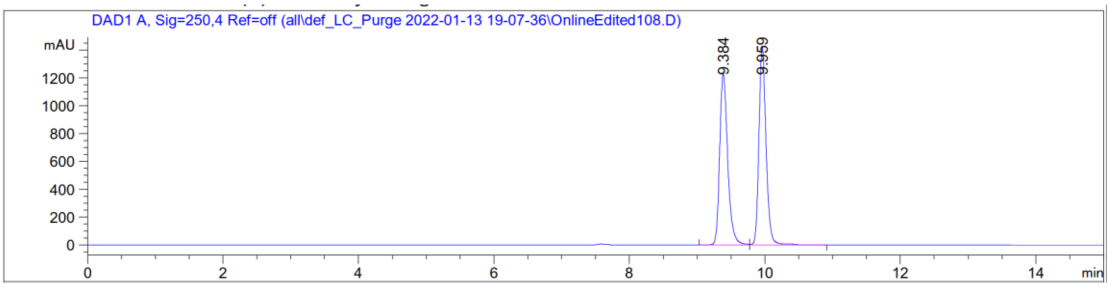

Peak #	RetTime [min]	Type	Width [min]	Area [mAU*s]	Height [mAU]	Area %
1	9.384	BV	0.1254	1.01427e4	1227.78308	49.4675
2	9.959	VB	0.1119	1.03611e4	1423.09216	50.5325

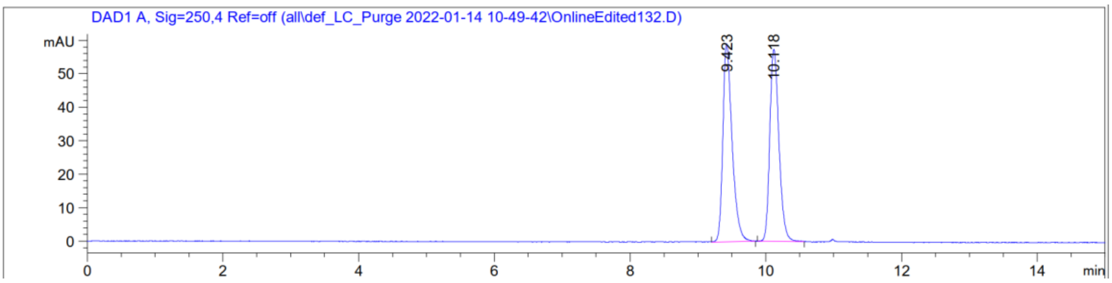

Peak #	RetTime [min]	Type	Width [min]	Area [mAU*s]	Height [mAU]	Area %
1	9.423	BB	0.1389	557.36005	59.16850	50.9763
2	10.118	BB	0.1460	536.01105	57.38251	49.0237

6. X-Ray Analysis

3h (10 mg, 90% ee) was recrystallized from *n*-hexane/EtOAc/CH_2Cl_2 at RT by slow evaporation to obtain suitable crystals for X-Ray crystallographic analysis.

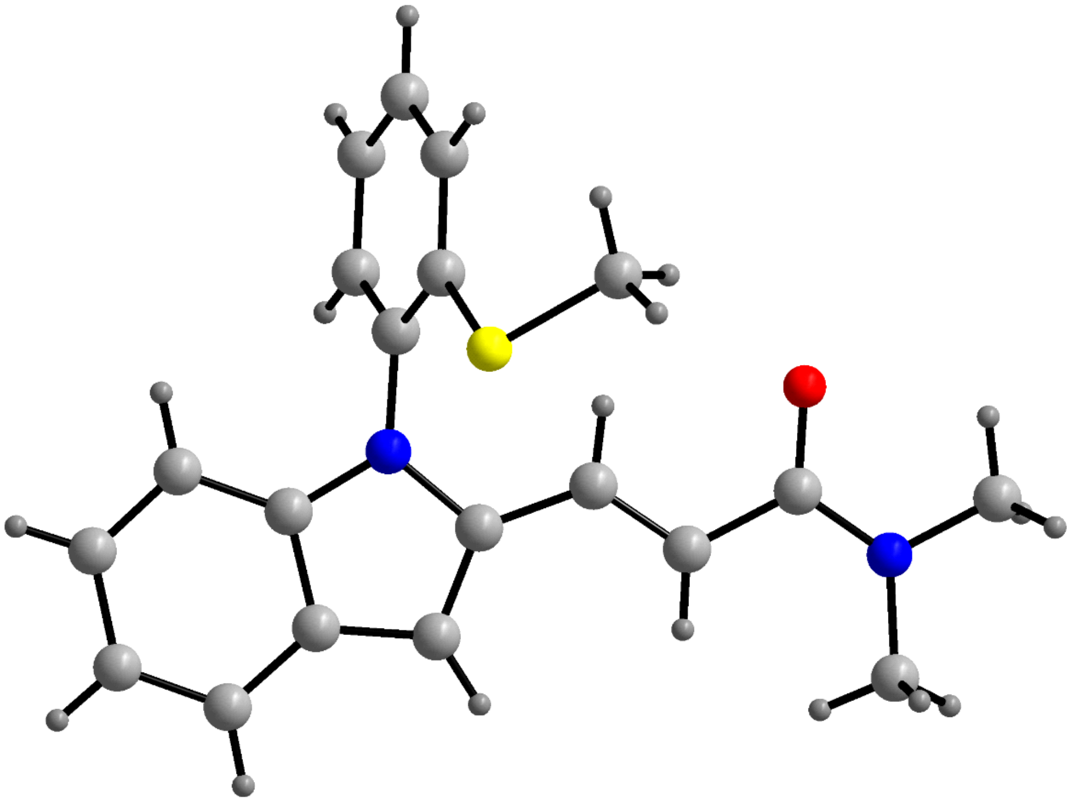

Fig. S1 X-Ray crystallographic data of **3h.**

compound	**3h**
CCDC number	CCDC 2144688
Empirical formula	$C_{20}H_{20}N_2OS$
Formula weight	336.44
Temperature [K]	100.00
Crystal system	monoclinic
Space group (number)	$P2_1$ (4)
a [Å]	11.0831(4)
b [Å]	6.8389(3)
c [Å]	12.0729(5)
α [°]	90
β [°]	109.142(2)
γ [°]	90
Volume [Å^3]	864.48(6)

Z	2
ρ_{calc} [gcm^{-3}]	1.292
μ [mm^{-1}]	0.196
F(000)	356
Crystal size [mm^3]	0.413×0.198×0.168
Crystal colour	colourless
Crystal shape	block
Radiation	MoK_α (λ=0.71073 Å)
2Θ range [°]	3.57 to 66.92 (0.64 Å)
Index ranges	-17 ≤ h ≤ 16, -10 ≤ k ≤ 10, -18 ≤ l ≤ 18
Reflections collected	30575
Independent reflections	6087 [R_{int} = 0.0157, R_{sigma} = 0.0123]
Data / Restraints / Parameters	6087/1/220
Goodness-of-fit on F^2	1.063
Final *R* indexes [I≥2σ(I)]	R_1 = 0.0250, wR_2 = 0.0714
Final *R* indexes [all data]	R_1 = 0.0257, wR_2 = 0.0723
Largest peak/hole [eÅ^{-3}]	0.34/-0.18
Flack X parameter	0.003(7)

Table S1: Selected bond lengths [Å] and angles [°] for **3h**

S1–C1	1.7712(11)	C8–C18	1.3950(14)
S1–C7	1.7942(15)	C9–C10	1.4281(15)
O1–C14	1.2413(13)	C9–C15	1.4085(15)
N1–C2	1.4244(13)	C10–H10	0.9500
N1–C8	1.3785(13)	C10–C11	1.3812(14)
N1–C11	1.4001(13)	C11–C12	1.4448(15)
N2–C14	1.3544(13)	C12–H12	0.9500
N2–C19	1.4567(14)	C12–C13	1.3385(14)
N2–C20	1.4578(15)	C13–H13	0.9500
C1–C2	1.4024(15)	C13–C14	1.4870(15)
C1–C6	1.4022(14)	C15–H15	0.9500
C2–C3	1.3973(15)	C15–C16	1.3861(16)
C3–H3	0.9500	C16–H16	0.9500
C3–C4	1.3921(15)	C16–C17	1.4098(17)
C4–H4	0.9500	C17–H17	0.9500
C4–C5	1.391(2)	C17–C18	1.3843(16)
C5–H5	0.9500	C18–H18	0.9500
C5–C6	1.3871(18)	C19–H19A	0.9800

C6–H6	0.9500	C19–H19B	0.9800
C7–H7A	0.9800	C19–H19C	0.9800
C7–H7B	0.9800	C20–H20A	0.9800
C7–H7C	0.9800	C20–H20B	0.9800
C8–C9	1.4168(14)	C20–H20C	0.9800

5k (10 mg, 96% ee) was recrystallized from *n*-hexane/EtOAc/CH_2Cl_2 at RT by slow evaporation to obtain suitable crystals for X-Ray crystallographic analysis.

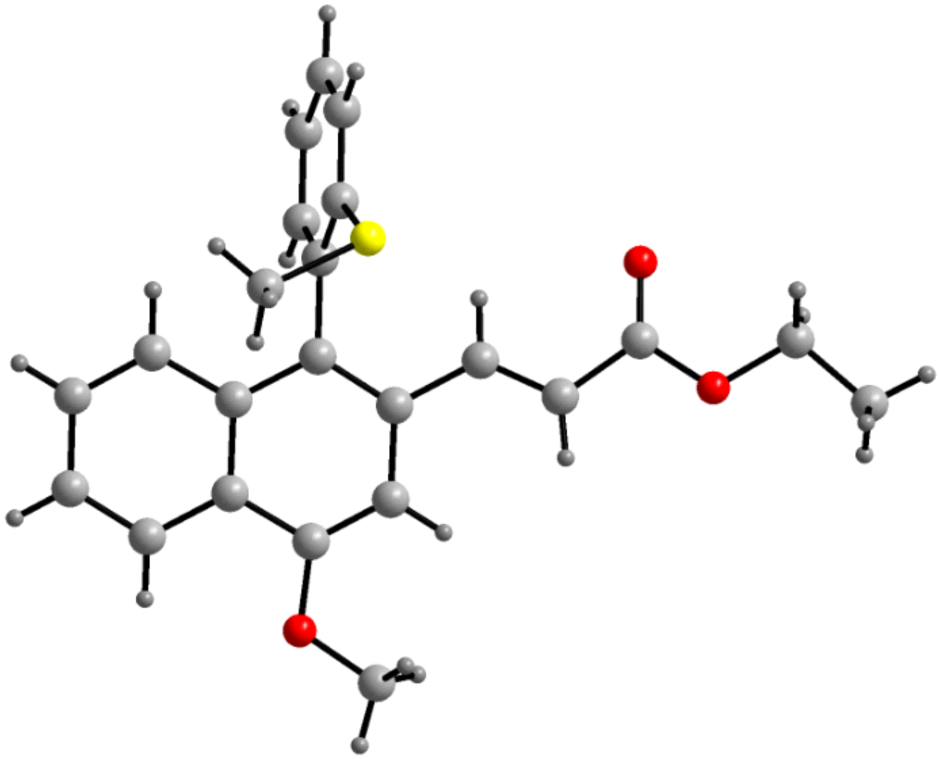

Fig. S2 X-Ray crystallographic data of **5k.**

compound	**5k**
CCDC number	CCDC 2130699
Empirical formula	$C_{23}H_{22}O_3S$
Formula weight	378.46
Temperature [K]	100.00
Crystal system	triclinic
Space group (number)	*P*1 (1)
a [Å]	7.2748(7)
b [Å]	12.2905(9)
c [Å]	12.5063(12)

α [°]	63.599(2)
β [°]	79.446(2)
γ [°]	75.302(2)
Volume [Å^3]	965.60(15)
Z	2
ρ_{calc} [gcm^{-3}]	1.302
μ [mm^{-1}]	0.188
F(000)	400
Crystal size [mm^3]	0.181×0.084×0.071
Crystal colour	colourless
Crystal shape	block
Radiation	MoK_α (λ=0.71073 Å)
2Θ range [°]	5.81 to 57.46 (0.74 Å)
Index ranges	$-9 \le h \le 9$, $-16 \le k \le 16$, $-16 \le l \le 16$
Reflections collected	73464
Independent reflections	9983, R_{int} = 0.0258, R_{sigma} = 0.0155
Data / Restraints / Parameters	9983/3/493
Goodness-of-fit on F^2	1.039
Final R indexes [$I \geq 2\sigma(I)$]	R_1 = 0.0259, wR_2 = 0.0651
Final R indexes [all data]	R_1 = 0.0272, wR_2 = 0.0662
Largest peak/hole [eÅ^{-3}]	0.28/-0.20
Flack X parameter	-0.008(10)

Table S2: Selected bond lengths [Å] and angles [°] for **5k**

S1–C1	1.7696(17)	S2–C24	1.7772(17)
S1–C20	1.8007(18)	S2–C43	1.806(2)
O1–C14	1.365(2)	O4–C37	1.3676(19)
O1–C21	1.424(2)	O4–C44	1.426(2)
O2–C19	1.344(2)	O5–C42	1.344(2)
O2–C22	1.452(2)	O5–C45	1.449(2)
O3–C19	1.215(2)	O6–C42	1.210(2)
C1–C2	1.399(2)	C24–C25	1.404(2)
C1–C6	1.409(2)	C24–C29	1.397(2)
C2–H2	0.9500	C25–C26	1.399(2)
C2–C3	1.390(3)	C25–C30	1.498(2)
C3–H3	0.9500	C26–H26	0.9500
C3–C4	1.387(3)	C26–C27	1.392(2)

C4–H4	0.9500	C27–H27	0.9500
C4–C5	1.392(2)	C27–C28	1.385(3)
C5–H5	0.9500	C28–H28	0.9500
C5–C6	1.394(2)	C28–C29	1.382(3)
C6–C7	1.498(2)	C29–H29	0.9500
C7–C8	1.429(2)	C30–C31	1.433(2)
C7–C16	1.390(2)	C30–C39	1.386(2)
C8–C9	1.425(2)	C31–C32	1.426(2)
C8–C13	1.418(2)	C31–C36	1.424(2)
C9–C10	1.416(2)	C32–H32	0.9500
C9–C14	1.431(2)	C32–C33	1.370(3)
C10–H10	0.9500	C33–H33	0.9500
C10–C11	1.371(3)	C33–C34	1.409(3)
C11–H11	0.9500	C34–H34	0.9500
C11–C12	1.410(3)	C34–C35	1.375(3)
C12–H12	0.9500	C35–H35	0.9500
C12–C13	1.373(2)	C35–C36	1.417(2)
C13–H13	0.9500	C36–C37	1.428(2)
C14–C15	1.366(2)	C37–C38	1.365(2)
C15–H15	0.9500	C38–H38	0.9500
C15–C16	1.428(2)	C38–C39	1.429(2)
C16–C17	1.464(2)	C39–C40	1.468(2)
C17–H17	0.9500	C40–H40	0.9500
C17–C18	1.338(2)	C40–C41	1.334(2)
C18–H18	0.9500	C41–H41	0.9500
C18–C19	1.473(2)	C41–C42	1.479(2)
C20–H20A	0.9800	C43–H43A	0.9800
C20–H20B	0.9800	C43–H43B	0.9800
C20–H20C	0.9800	C43–H43C	0.9800
C21–H21A	0.9800	C44–H44A	0.9800
C21–H21B	0.9800	C44–H44B	0.9800
C21–H21C	0.9800	C44–H44C	0.9800
C22–H22A	0.9900	C45–H45A	0.9900
C22–H22B	0.9900	C45–H45B	0.9900
C22–C23	1.506(3)	C45–C46	1.508(3)
C23–H23A	0.9800	C46–H46A	0.9800
C23–H23B	0.9800	C46–H46B	0.9800
C23–H23C	0.9800	C46–H46C	0.9800

7. Key Mechanistic Findings

7.1 KIE Studies

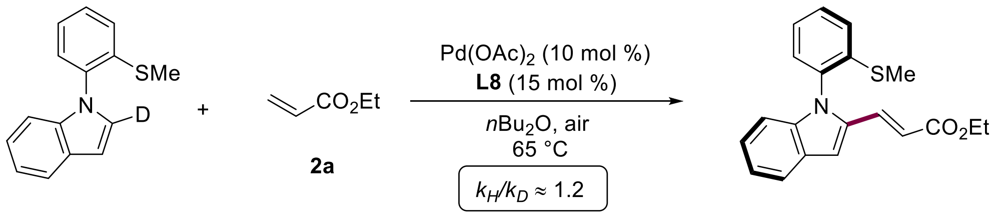

1a-D or **1a** **3a**-D or **3a**

Two parallel reactions of **1a** and **1a**-D with **2a** were performed to determine the KIE by comparison of the initial reaction rates through ^{1}H-NMR-analysis with triphenylmethane as the the internal standard. A suspension of **1a** (23.9 mg, 0.10 mmol, 1.0 equiv) or **1a**-D (23.9 mg, 0.10 mmol, 1.0 equiv), **2a** (32 µL, 0.30 mmol, 3.0 equiv), $Pd(OAc)_2$ (2.3 mg, 10 mol %), **L8** (11.3 mg, 10 mol %) and triphenylmethane (24.4 mg, 0.10 mmol) in $n$$Bu_2O$ (2.0 mL) was stirred at 65 °C. Aliquots (40 µL) were periodically removed to provide the following conversions as determined by ^{1}H-NMR.

Table S3: Conversion-time table.

t/min	20	40	60	80	100	120
**1a** / %	5.5	6.7	8.1	9.8	11.6	12
**1a-D** / %	5.3	7.1	8.5	9.5	10.4	11.3

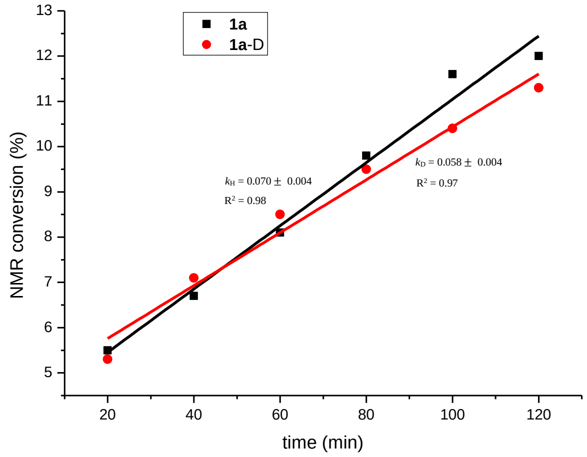

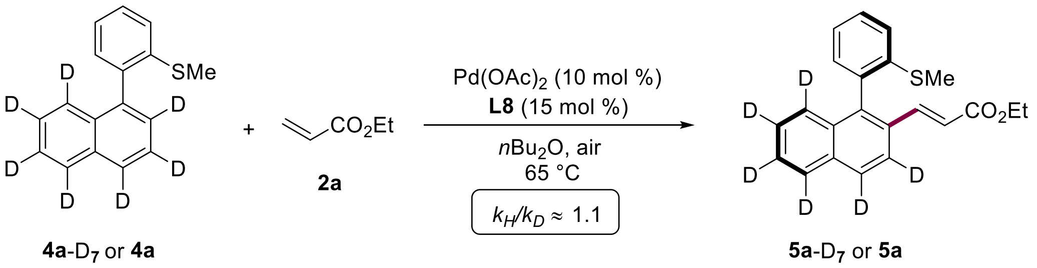

Two parallel reactions of **4a** and **4a**-D_7 with **2a** were performed to determine the KIE by comparison of the initial reaction rates through ^{1}H-NMR-analysis with triphenylmethane as the the internal standard. A suspension of **4a** (25.1 mg, 0.10 mmol, 1.0 equiv) or **4a**-D_7 (25.7 mg, 0.10 mmol, 1.0 equiv), **2a** (32 µL, 0.30 mmol, 3.0 equiv), $Pd(OAc)_2$ (2.4 mg, 10 mol %), **L8** (11.3 mg, 10 mol %) and triphenylmethane (24.4 mg, 0.10 mmol) in $n$$Bu_2O$ (2.0 mL) was stirred at 65 °C. Aliquots (40 µL) were periodically removed to provide the following conversions as determined by ^{1}H-NMR.

Table S4: Conversion-time table.

t/min	20	40	60	80	100	120
**5a** / %	3.1	5.7	8.8	13.8	15.6	18.1
**5a-D$_7$** / %	3	4.8	7.9	11.6	14.3	17.0

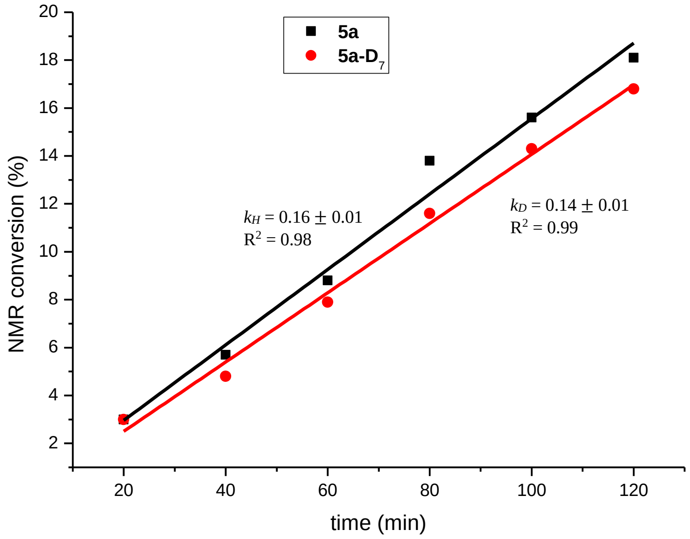

7.2 Reaction under N_2 atmosphere

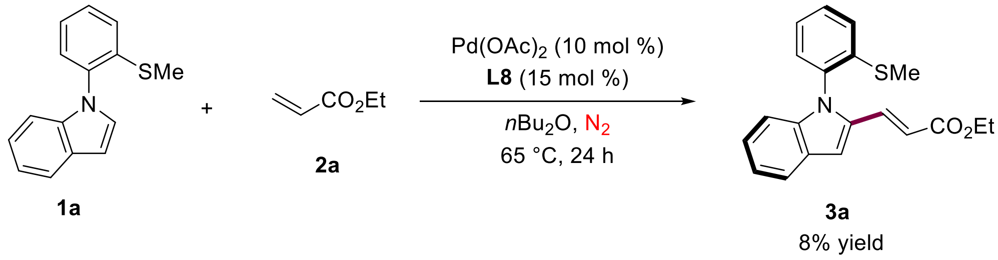

To an oven-dried 25 mL Schlenk tube was added substrate 1-(2-(methylthio)phenyl)-1*H*-indole (23.9 mg, 0.10 mmol), ethyl acrylate (32 µL, 0.30 mmol), $Pd(OAc)_2$ (2.3 mg, 0.010 mmol), **L8** (11.3 mg, 0.0150 mmol), *n*-Bu_2O (2.0 mL). The mixture was stirred for 24 h at 65 °C under N_2 atmosphere. The resulting mixture was purified by column chromatography on silica gel (*n*-hexane/EtOAc: 15/1) yielded **3a** (2.7 mg, 8%) as a yellow oil.

7.3 Reaction under $^{18}O_2$ atmosphere

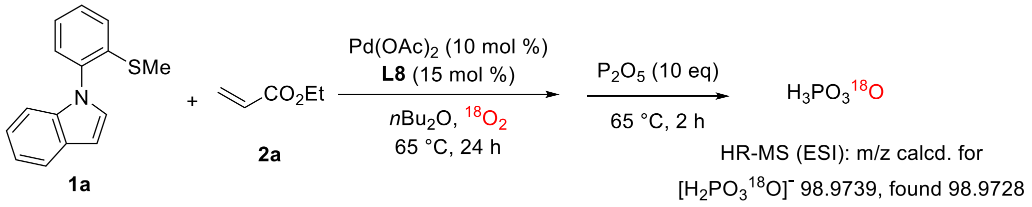

To an oven-dried 25 mL Schlenk tube was added substrate 1-(2-(methylthio)phenyl)-1*H*-indole (23.9 mg, 0.10 mmol), ethyl acrylate (32 µL, 0.30 mmol), $Pd(OAc)_2$ (2.3 mg, 0.010 mmol), **L8** (11.3 mg, 0.0150 mmol), *n*-Bu_2O (2.0 mL). The mixture was stirred for 24 h at 65 °C under $^{18}O_2$ atmosphere ($^{18}O_2$ balloon). Then P_2O_5 (142 mg, 1 mmol) was added. After being stirred at 65 °C for 2 h, the reaction mixture was analysed by HR-MS. The existence of ^{18}O-containing phosphoric acid was established by HR-MS (ESI): m/z calcd. for $[H_2PO_3{}^{18}O]^-$ 98.9739, found 98.9728.

HR-MS analysis of ^{18}O-containing phosphoric acid:

Display Report

Analysis Info

Analysis Name	Z:\Data\2022\2203\sam030322\neg\yli00002_14_01_115797.d	Acquisition Date	04.03.2022 01:44:40
Method	hystar_nl.m	Operator	BDAL@DE
Sample Name	yli00002	Instrument / Ser#	micrOTOF 10237
Comment			

Acquisition Parameter

Source Type	ESI	Ion Polarity	Negative	Set Nebulizer	1.6 Bar
Focus	Not active			Set Dry Heater	180 °C
Scan Begin	50 m/z	Set Capillary	3800 V	Set Dry Gas	8.0 l/min
Scan End	1600 m/z	Set End Plate Offset	-500 V	Set Divert Valve	Source

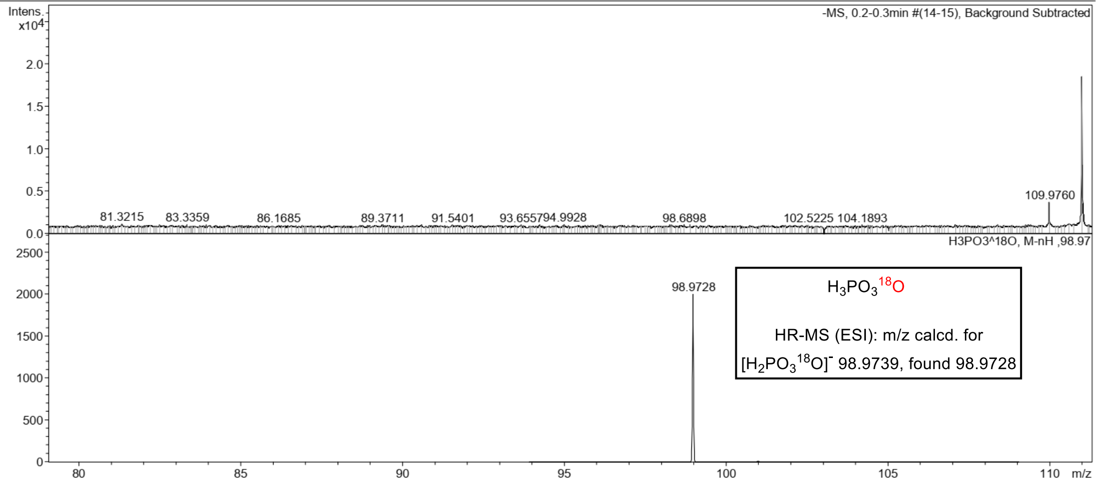

8. Computational Methods

All DFT calculations were carried out with Gaussian 16 program.[2] The geometry optimizations were conducted using B3LYP functional[3] including Grimme's dispersion corrections[4] with Becke-Johnson damping, LANL2DZ basis set[5] for palladium and 6-31G(d) basis set for other atoms. To confirm whether each optimized stationary point is an energy minimum or a transition state as well as evaluate the zero-point vibrational energy and thermal corrections at 298 K, the vibrational frequencies were computed at the same level of theory as for the geometry optimizations. On the basis of the gas-phase optimized structures, the single-point energies and solvent effects were evaluated with the ω-B97XD[6] functional SDD basis set[7] for palladium and 6-311+G(d, p) for other atoms. The solvation energies were calculated using the self-consistent reaction field with the SMD implicit solvent model.[8] The 3D diagrams of molecules were generated using CYLView.[9]

The half-life calculations are based on **equation 1** and **equation 2**.

$$k = \frac{\kappa k_b T}{h} e^{-\frac{\Delta G^{\ddagger}}{RT}} \qquad (1)$$

In the above Eyring Equation, $\Delta G\ddagger$ is the Gibbs energy of activation, κ is the transmission coefficient, k_b is Boltzmann's constant, and h is Planck's constant. The transmission coefficient is often assumed to be equal to 1 as it reflects what fraction of the flux through the transition state proceeds to the product without recrossing the transition state.

$$t_{1/2} = ln2/2k \qquad (2)$$

The epimerization of atropoisomer is a first order reaction, which makes the half-life $t_{1/2}$ only relates to the reaction rate constant k.

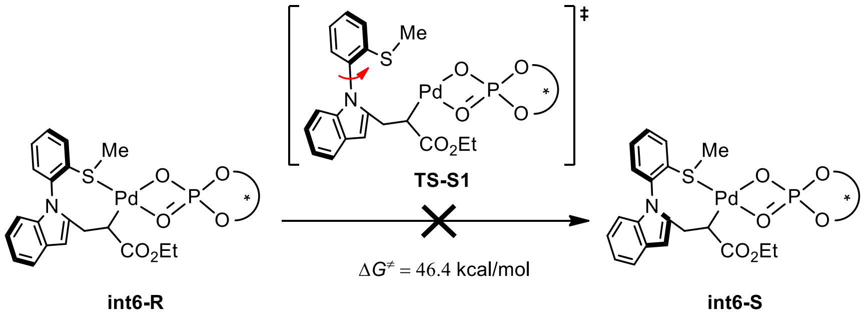

Fig. S3 Racemization of axial chirality of the alkylpalladium species **int6-R**.

Table of Energies

Zero-point correction (*ZPE*) thermal correction to enthalpy (*TCH*) thermal correction to Gibbs free energy (*TCG*) energies (*E*) enthalpies (*H*) and Gibbs free energy (*G*) (in Hartree) of the

structures calculated at the ω-B97XD /6-311+G(d,p)-SDD-SMD(Dibutylether)//B3LYP-D3(BJ)/6-31G(d)-LANL2DZ level of theory.

Table S5. Energies in Fig.1, Fig. 2 and Fig. S3.

Structures	*ZPE*	*TCH*	*TCG*	*E*	*H*	*G*	Imaginary Frequency
**$[Pd(OAc)_2]_3$**	0.316177	0.351925	0.241778	−1754.838162	−1754.486237	−1754.596384	
**int1-R**	1.242826	1.319038	1.131474	−3970.181890	−3968.862852	−3969.050416	
**TS2-R**	1.237017	1.313235	1.122824	−3970.163920	−3968.850685	−3969.041096	982.54*i*
**int3-R**	1.244049	1.320021	1.132991	−3970.204296	−3968.884275	−3969.071305	
**int4-R**	1.306712	1.386316	1.189956	−4086.882412	−4085.496096	−4085.692456	
**TS5-R**	1.305505	1.384338	1.189673	−4086.863756	−4085.479418	−4085.674083	305.17*i*
**int6-R**	1.308238	1.387134	1.192859	−4086.904002	−4085.516868	−4085.711143	
**TS7-R**	1.301928	1.381279	1.184825	−4086.867237	−4085.485958	−4085.682412	616.78*i*
**int8-R**	1.304635	1.383849	1.189451	−4086.888951	−4085.505102	−4085.6995	
**TS9-R**	1.301672	1.381114	1.184645	−4086.862892	−4085.481778	−4085.678247	185.27*i*
**int10-R**	1.306218	1.385955	1.189965	−4086.894407	−4085.508452	−4085.704442	
**int1-S**	1.241848	1.318724	1.124625	−3970.171517	−3968.887403	−3969.075849	
**TS2-S**	1.237875	1.313554	1.126176	−3970.164624	−3968.85107	−3969.038448	756.16*i*
**int3-S**	1.243398	1.319481	1.131035	−3970.206884	−3968.887403	−3969.075849	
**int4-S**	1.305539	1.385454	1.187702	−4086.884825	−4085.499371	−4085.697123	
**TS5-S**	1.305672	1.384390	1.192499	−4086.861985	−4085.477595	−4085.669486	299.91*i*
**int6-S**	1.307418	1.386408	1.191037	−4086.898917	−4085.512509	−4085.70788	

**TS7-S**	1.301969	1.381289	1.184732	−4086.865980	−4085.484691	−4085.681248	598.16*i*
**int8-S**	1.304797	1.383978	1.189493	−4086.885481	−4085.501503	−4085.695988	
**TS9-S**	1.301556	1.380809	1.184805	−4086.851064	−4085.470255	−4085.666259	86.02*i*
**int10-S**	1.306176	1.385809	1.187428	−4086.895048	−4085.509239	−4085.7076	
**TS-S1**	1.307504	1.385611	1.193184	-4086.830379	−4085.444768	−4085.637195	35.53*i*
**L8**	0.956620	1.011968	0.866503	−2581.983124	−2580.971156	−2581.116621	
**1a**	0.239968	0.255039	0.197554	−1032.324898	−1032.069859	−1032.127344	
**2a**	0.124415	0.133267	0.091636	−345.776868	−345.643601	−345.685232	
**HOAc**	0.061763	0.067264	0.034875	−229.087313	−229.020049	−229.052438	

Cartesian coordinates of the structures

[Pd(OAc)$_2$]$_3$

Pd	1.58430000	0.91139100	0.00864600
C	-0.04506000	2.57847100	-1.87910200
O	1.02408200	2.45682300	-1.21241800
O	-1.13969600	1.96701100	-1.69595400
C	-2.28370600	-1.23902100	1.85882200
O	-1.62818200	-2.10734900	1.21308000
O	-2.28813300	0.01546600	1.67573100
C	-3.19115000	-1.76102800	2.95176600
H	-3.41808800	-0.96939700	3.66770000
H	-4.12834200	-2.09672700	2.49309100
H	-2.72801200	-2.61585700	3.44867400
C	-0.00061100	3.56568200	-3.02537800
H	0.72199500	3.21432800	-3.76886400
H	-0.98489600	3.67387900	-3.48202200
H	0.35185800	4.53255500	-2.65423400
Pd	-0.00241900	-1.82610700	-0.00017900

C	-2.19887700	-1.33749800	-1.88864300
O	-2.63330900	-0.34710300	-1.23039000
O	-1.12402500	-1.97904900	-1.69293700
C	0.05215200	2.57836700	1.87910900
O	1.14474800	1.96313600	1.69638300
O	-1.01730500	2.45997600	1.21233600
C	0.01085500	3.56629300	3.02488600
H	-0.71343900	3.21809600	3.76821100
H	0.99532800	3.67103800	3.48192800
H	-0.33776600	4.53429700	2.65304300
C	-3.06524500	-1.79688700	-3.04132600
H	-3.09524000	-1.00680500	-3.79862700
H	-2.67435500	-2.71681200	-3.47720000
H	-4.08791200	-1.95123300	-2.68509500
Pd	-1.58182500	0.91593700	-0.00852700
C	2.19488800	-1.34367200	1.88886800
O	2.63218100	-0.35450200	1.23066600
O	1.11866100	-1.98272800	1.69253900
C	3.05924000	-1.80479100	3.04236700
H	3.08929200	-1.01506000	3.80004400
H	2.66653200	-2.72433900	3.47740200
H	4.08215200	-1.96033000	2.68738700
C	2.28053800	-1.24503900	-1.85900900
O	2.28835700	0.00942100	-1.67574400
O	1.62258000	-2.11166700	-1.21347500
C	3.18671200	-1.76932500	-2.95191100
H	4.12315900	-2.10708500	-2.49322400
H	2.72158700	-2.62315700	-3.44867100
H	3.41545100	-0.97832600	-3.66797300

int1-R

C	0.74260500	1.53267500	-5.35789400
C	0.41005700	1.94266600	-4.08455800
C	1.30336200	1.75247200	-2.99542000
C	2.55526400	1.10382800	-3.25006000
C	2.87944200	0.72031200	-4.57940100
C	1.99397800	0.92391200	-5.61376100
H	0.03970000	1.68211200	-6.17249400

H	-0.54952200	2.41075100	-3.89851500
C	0.97885700	2.12890000	-1.65043100
C	3.43194700	0.82671400	-2.16974600
H	3.84281000	0.24897200	-4.76046900
H	2.25097700	0.61783900	-6.62370500
C	3.08349700	1.08917500	-0.86233800
C	1.83129300	1.72822100	-0.63291400
H	4.39164600	0.36467500	-2.38110200
C	-0.26134000	2.87915000	-1.31280600
C	-0.55547300	4.16317400	-1.88313200
C	-1.14730100	2.35387300	-0.38696600
C	0.34754000	4.85115700	-2.73720700
C	-1.79588100	4.79934500	-1.55229600
C	-2.38912100	2.96644800	-0.04984300
C	0.02560100	6.08145600	-3.26604500
H	1.30670900	4.40024800	-2.96206400
C	-2.10117400	6.06224500	-2.12371000
C	-2.69387600	4.16463800	-0.65385400
C	-1.21495600	6.69141700	-2.96757800
H	0.73459800	6.59146700	-3.91196300
H	-3.04964200	6.52868700	-1.86967100
H	-3.64136700	4.64828000	-0.43692000
H	-1.45841900	7.66027100	-3.39388000
O	1.47961300	1.99022800	0.67150100
O	-0.85690000	1.13124000	0.19460700
P	0.31517000	1.05758200	1.38322400
O	0.78402800	-0.40312100	1.27435400
O	-0.05599200	1.67934200	2.66925000
C	3.96367600	0.72813800	0.28782500
C	4.23536300	-0.62864700	0.58223100
C	4.51694300	1.74688600	1.09354300
C	5.05293900	-0.93209600	1.67246800
C	5.32706300	1.38832700	2.17449800
C	5.60356800	0.05872400	2.48694500
H	5.25263200	-1.97485600	1.90040600
H	5.75466100	2.17087200	2.79615600
C	-3.29599600	2.21046600	0.86692200
C	-3.24531400	2.40852100	2.26059000

C	-4.08304800	1.17586300	0.31631100
C	-3.91859100	1.49824700	3.08258800
C	-4.73940000	0.29645400	1.17730900
C	-4.62986500	0.41661700	2.56548000
H	-5.32123600	-0.51984500	0.76166700
H	-3.85147400	1.60776600	4.15994300
C	-0.18964800	-2.88124100	3.37314000
O	0.58309500	-3.70178400	2.88059400
O	-0.98471300	-2.08893600	2.69005700
C	-0.31961300	-2.66628600	4.87094800
H	-1.37163800	-2.61074000	5.16509000
H	0.18543500	-3.47154900	5.40743700
H	0.14350900	-1.70656800	5.12492900
Pd	-0.52185500	-1.92947900	0.73799000
H	0.15915800	-4.39579100	0.72330000
C	-2.88482100	-3.80747300	-0.52388000
C	-2.09591900	-4.11777900	-1.65484700
C	-2.63316600	-4.20367100	-2.93868500
C	-4.01058200	-4.02517000	-3.06140300
C	-4.82115600	-3.76761500	-1.94057200
C	-4.26833300	-3.64919000	-0.66948300
C	-1.99103600	-3.76853400	0.61281200
C	-0.70901700	-4.10725300	0.14324800
H	-2.00687300	-4.41640100	-3.79882100
H	-4.46814600	-4.09663800	-4.04350100
H	-5.89087900	-3.64373100	-2.07510300
H	-4.88942500	-3.42663200	0.19220700
H	-2.28689700	-3.75439600	1.65053800
C	0.33041700	-4.12320100	-2.11068900
C	0.88430300	-5.20980800	-2.78274600
C	0.82053700	-2.82425200	-2.34144400
C	1.92431000	-5.01216600	-3.69347500
H	0.49043600	-6.20103300	-2.58365500
C	1.86130200	-2.63418900	-3.25341900
C	2.40527900	-3.72570800	-3.93278100
H	2.35528100	-5.86317100	-4.21137900
H	2.24245200	-1.63333600	-3.42328000
H	3.21368100	-3.56612300	-4.63941300

N	-0.77486800	-4.31871900	-1.23126300
S	0.23671600	-1.36946000	-1.46492200
C	-1.16997200	-0.84491800	-2.50241300
H	-1.90260500	-1.64135000	-2.61439200
H	-1.60495300	0.00639000	-1.98017700
H	-0.77393000	-0.52385800	-3.46819700
C	-2.51835400	3.60819400	2.85443500
C	-2.02391500	3.39726800	4.29058900
C	-3.43131200	4.84903400	2.79071800
H	-1.63626200	3.79818100	2.23531500
H	-1.40651800	2.50056700	4.35569900
H	-1.41395200	4.25546600	4.59483300
H	-2.85667000	3.32498600	5.00143800
H	-3.74221500	5.07542100	1.76665600
H	-4.33763700	4.68812200	3.38736100
H	-2.91275500	5.72841500	3.19109600
C	-5.22479200	-0.63333600	3.48682600
C	-4.51967000	-1.98754300	3.28186800
C	-6.74643000	-0.76764200	3.31759500
H	-5.02925500	-0.31097700	4.51799000
H	-3.43360300	-1.88576700	3.37441600
H	-4.87496600	-2.72807500	4.00921800
H	-4.72903200	-2.37846600	2.27774800
H	-7.24864200	0.19028800	3.49043600
H	-7.00358700	-1.10071000	2.30482100
H	-7.15364400	-1.50229000	4.02251800
C	-4.24695200	1.05041100	-1.19351200
C	-5.37063800	1.98147600	-1.68623000
C	-4.50020000	-0.38370300	-1.66538100
H	-3.31783000	1.39416200	-1.66251700
H	-5.16163800	3.02427700	-1.43157800
H	-5.48727200	1.91123400	-2.77480300
H	-6.32393000	1.70507000	-1.22048600
H	-3.77883100	-1.07885200	-1.23167500
H	-5.50076600	-0.73257300	-1.38690800
H	-4.43024900	-0.44900900	-2.75745300
C	3.67930000	-1.77805900	-0.24781000
C	3.01093100	-2.86209000	0.61068100

C	4.77680000	-2.39682200	-1.13169400
H	2.91245400	-1.36695600	-0.90606900
H	2.26172700	-2.43788700	1.28042700
H	2.52424700	-3.60200800	-0.03763100
H	3.73827100	-3.40604300	1.22331700
H	5.22710600	-1.65376400	-1.79932500
H	5.58123000	-2.81297000	-0.51412200
H	4.36774000	-3.20750800	-1.74610000
C	4.30450600	3.22795800	0.80600500
C	5.62523800	3.89075000	0.37472600
C	3.67259000	3.96117800	2.00068100
H	3.61402100	3.32360300	-0.03542900
H	6.04898000	3.38963700	-0.50299100
H	5.46223600	4.94561900	0.12336200
H	6.37163700	3.84794700	1.17636200
H	2.73209700	3.48763100	2.29290100
H	4.34311500	3.96383800	2.86802100
H	3.46797300	5.00588900	1.73757500
C	6.46217900	-0.29875600	3.68716200
C	7.71583500	-1.08915000	3.27678100
C	5.64629200	-1.06387000	4.74369200
H	6.79634000	0.64375600	4.14136100
H	8.30865700	-0.53414100	2.54161400
H	8.34924800	-1.29229400	4.14839700
H	7.44548000	-2.05285800	2.82957400
H	4.76840800	-0.48742800	5.05333000
H	5.29117700	-2.02194400	4.34680300
H	6.25609300	-1.27168400	5.63115900

TS2-R

C	-2.49471100	2.11568000	5.19244900
C	-2.17187000	2.38393600	3.88030300
C	-2.70649600	1.60295200	2.81992000
C	-3.56045000	0.50235000	3.15384000
C	-3.89026300	0.26626300	4.51451800
C	-3.37257500	1.05487800	5.51647600
H	-2.06925900	2.72388700	5.98561600
H	-1.49361600	3.19517200	3.64540600

C	-2.36535300	1.82443800	1.44215500
C	-4.03663000	-0.35096700	2.12448900
H	-4.55217900	-0.56369900	4.74920300
H	-3.62702500	0.85988800	6.55429000
C	-3.64539600	-0.20008400	0.81200300
C	-2.78931700	0.89616600	0.50326000
H	-4.71805000	-1.15484000	2.38598500
C	-1.49330500	2.95614500	1.02035800
C	-1.82766500	4.32254800	1.29905300
C	-0.32895500	2.69509200	0.31255300
C	-3.05981000	4.70863000	1.89222700
C	-0.89564700	5.34962200	0.93955500
C	0.59337100	3.70063300	-0.08146500
C	-3.33727900	6.03203800	2.15224800
H	-3.79012000	3.94491500	2.13212400
C	-1.20798300	6.70339600	1.23282700
C	0.30581800	5.00198500	0.27027900
C	-2.39949800	7.04165300	1.83135300
H	-4.28827200	6.30412500	2.60126400
H	-0.48771000	7.47064100	0.96033900
H	1.00079800	5.79133600	-0.00066300
H	-2.62919800	8.08144100	2.04537600
O	-2.37767300	1.03653200	-0.80650200
O	-0.00130200	1.38690400	0.02007600
P	-0.83738400	0.55904300	-1.16446900
O	-0.71817500	-0.88506500	-0.64923800
O	-0.49719600	0.97132900	-2.54037700
C	-4.13884500	-1.11415900	-0.26417800
C	-3.68698600	-2.45209500	-0.34715300
C	-5.08442600	-0.63225500	-1.19285300
C	-4.21749900	-3.28192600	-1.33643800
C	-5.57366400	-1.50057200	-2.17396800
C	-5.15866400	-2.82731100	-2.26271700
H	-3.87026100	-4.30885200	-1.39826300
H	-6.29961800	-1.12997800	-2.89351900
C	1.79974200	3.33565900	-0.88600900
C	1.80400600	3.59219300	-2.27122300
C	2.91179000	2.71496700	-0.27022900

C	2.94455900	3.25652700	-3.01080700
C	4.01838500	2.38759600	-1.05392900
C	4.05526700	2.65574400	-2.42664000
H	4.87387800	1.91015800	-0.58828200
H	2.95912700	3.45474800	-4.07868500
C	1.45469400	-3.48684700	-2.67586600
O	2.19569800	-4.20983600	-1.93640900
O	1.05904000	-2.31547800	-2.38828500
C	0.97291300	-4.05543500	-3.99065400
H	0.92967400	-3.26552400	-4.74338800
H	1.61554400	-4.87455700	-4.31653900
H	-0.04544400	-4.43381200	-3.84495000
Pd	1.20161200	-1.65961300	-0.43294300
H	2.63153000	-3.41683100	-0.98600200
C	5.27339900	-1.83655700	0.19543400
C	4.79810900	-2.54204200	1.34340300
C	5.59667600	-2.73311200	2.48069600
C	6.87770300	-2.20538900	2.45106800
C	7.37337200	-1.50862200	1.32097400
C	6.58898600	-1.32368200	0.19801300
C	4.20348400	-1.79939100	-0.73500400
C	3.10330000	-2.47380800	-0.19578500
H	5.21823100	-3.26057200	3.34982400
H	7.51953900	-2.32505100	3.31902500
H	8.38493500	-1.11539100	1.34526400
H	6.96768100	-0.78861200	-0.66734500
H	4.21212900	-1.34711200	-1.71675500
C	2.61825200	-3.53423100	2.02470100
C	2.83782200	-4.82964000	2.48776500
C	1.48596800	-2.81572000	2.44992100
C	1.92570200	-5.41620200	3.36657700
H	3.70932300	-5.37167400	2.13547800
C	0.56308800	-3.41101000	3.30971900
C	0.78948100	-4.71180700	3.76557900
H	2.09342200	-6.42804800	3.72121000
H	-0.32256500	-2.87482500	3.62845500
H	0.06950500	-5.17266700	4.43467700
N	3.49699200	-2.91815600	1.10209500

S	1.37340400	-1.13208600	1.84845700
C	-0.20689000	-0.55352900	2.51540500
H	-0.13265900	-0.49132100	3.60290700
H	-0.33883300	0.43647900	2.09003300
H	-1.03454900	-1.18575100	2.20277000
C	0.61933600	4.23893800	-2.97940100
C	0.25969900	3.54313300	-4.30156400
C	0.88291900	5.73881900	-3.20667200
H	-0.25252200	4.14515000	-2.32600700
H	0.08044300	2.48006800	-4.13155300
H	-0.65337000	3.98948100	-4.71354200
H	1.04726900	3.66364500	-5.05522200
H	1.07481500	6.26409400	-2.26511400
H	1.75744100	5.88408000	-3.85266800
H	0.02138000	6.21266800	-3.69204900
C	5.27095400	2.29311000	-3.26192200
C	5.51344400	0.77472300	-3.26975300
C	6.52748700	3.04588100	-2.79321300
H	5.06164700	2.60134100	-4.29469000
H	4.62398200	0.23860800	-3.61931900
H	6.35515100	0.51260700	-3.92188700
H	5.74730700	0.41851100	-2.26000900
H	6.36574800	4.12878800	-2.81210100
H	6.79250400	2.76670600	-1.76646200
H	7.38509700	2.81366200	-3.43606000
C	2.92833400	2.45030800	1.23171100
C	3.25110500	3.74122500	2.00842700
C	3.89586700	1.33434700	1.64965500
H	1.91996600	2.13258800	1.52140800
H	2.52517900	4.52995400	1.79347700
H	3.24353500	3.55515600	3.08952200
H	4.24655400	4.10969900	1.73342300
H	3.75700400	0.43296200	1.04821400
H	4.94230900	1.64558900	1.55105500
H	3.73400400	1.07046300	2.70046400
C	-2.62576600	-3.01329900	0.59065300
C	-1.56454900	-3.84768600	-0.14720700
C	-3.24563700	-3.83581000	1.73304400

H	-2.10678300	-2.15749600	1.02206900
H	-1.19660100	-3.30972300	-1.02276000
H	-0.71674300	-4.04712500	0.51977600
H	-1.95963500	-4.81899000	-0.46802500
H	-3.93584200	-3.23875000	2.33704800
H	-3.80532300	-4.69034000	1.33467700
H	-2.46179300	-4.22726400	2.39466000
C	-5.60428600	0.79926200	-1.16031800
C	-7.11858100	0.83455400	-0.89093500
C	-5.24226600	1.55678000	-2.44896300
H	-5.12263400	1.32312200	-0.33076300
H	-7.36352100	0.32290900	0.04667300
H	-7.47144100	1.87023000	-0.81961500
H	-7.67975400	0.34505500	-1.69524900
H	-4.16119000	1.54637100	-2.61038700
H	-5.72633100	1.10727100	-3.32400300
H	-5.57396900	2.59997100	-2.38323000
C	-5.69558900	-3.74315400	-3.34859000
C	-6.43507700	-4.95453700	-2.75646800
C	-4.57561100	-4.19163600	-4.30345900
H	-6.42092700	-3.16354700	-3.93518600
H	-7.24961900	-4.63489900	-2.09763400
H	-6.85937100	-5.57847000	-3.55206700
H	-5.75533400	-5.58123500	-2.16722200
H	-4.06536500	-3.32768500	-4.74191600
H	-3.82450600	-4.78866200	-3.77274200
H	-4.97963600	-4.80621800	-5.11684200

int3-R

C	-3.11623800	1.21357300	5.21229100
C	-2.86471200	1.61656800	3.91913800
C	-3.13598400	0.75931000	2.81830800
C	-3.64767200	-0.55131700	3.09082300
C	-3.91435400	-0.92763300	4.43359800
C	-3.65703300	-0.06665500	5.47601600
H	-2.89456500	1.88478800	6.03712000
H	-2.44347900	2.59652900	3.73030500
C	-2.84271500	1.12378800	1.46022000

C	-3.85362000	-1.45943500	2.01930000
H	-4.31580500	-1.92036200	4.62184700
H	-3.85896100	-0.36925300	6.49947600
C	-3.50269700	-1.14765800	0.72417900
C	-2.96796500	0.15029000	0.47942500
H	-4.28959000	-2.43089600	2.23311300
C	-2.31155100	2.47002700	1.10325100
C	-3.01262800	3.68433400	1.40285500
C	-1.10299500	2.56229800	0.43166300
C	-4.31479700	3.70171000	1.97036000
C	-2.38940700	4.93713200	1.09028900
C	-0.47616500	3.79144200	0.09129500
C	-4.95072200	4.89093100	2.24937200
H	-4.81080400	2.76023900	2.17564800
C	-3.06903200	6.14426300	1.40120200
C	-1.12044200	4.95390500	0.45311300
C	-4.32022600	6.12680200	1.97330500
H	-5.94849000	4.87989100	2.67879700
H	-2.58193000	7.08698000	1.16497000
H	-0.66473600	5.91253200	0.22329800
H	-4.83098800	7.05783400	2.20143500
O	-2.57951100	0.44083800	-0.81034100
O	-0.42641600	1.40434800	0.12024800
P	-0.95745500	0.44186100	-1.13791900
O	-0.39013000	-0.91233300	-0.69926900
O	-0.74674600	1.02247700	-2.48086700
C	-3.73094200	-2.09881200	-0.40790400
C	-2.93710400	-3.25832700	-0.56325500
C	-4.77286000	-1.83248700	-1.32103200
C	-3.22481700	-4.13763100	-1.60908200
C	-5.01203200	-2.73872000	-2.35868300
C	-4.25551700	-3.89741200	-2.52004700
H	-2.61569300	-5.02924500	-1.72400900
H	-5.81328800	-2.53452200	-3.06468300
C	0.81274300	3.74222600	-0.66596400
C	0.80901200	3.97825800	-2.05678700
C	2.00473300	3.35881300	-0.01163600
C	2.00530000	3.82512600	-2.76234800

C	3.17043300	3.19885100	-0.76649700
C	3.18752900	3.41643200	-2.14628400
H	4.08153900	2.89670200	-0.26585800
H	2.01161000	4.00244400	-3.83428600
C	2.82782300	-1.49015300	-3.17131900
O	4.04591900	-1.84355300	-2.79839000
O	1.94099300	-1.11648200	-2.40156500
C	2.59447300	-1.55793400	-4.65140500
H	1.53739000	-1.39775900	-4.86166400
H	3.19000400	-0.77713100	-5.13675700
H	2.92946500	-2.52374600	-5.04071500
Pd	1.72321200	-0.97296000	-0.25038300
H	4.16494300	-1.66669400	-1.82783500
C	5.89747600	-0.89493400	0.52640600
C	5.45942200	-1.92059000	1.40508700
C	6.33060500	-2.54886900	2.29870800
C	7.66539700	-2.15210700	2.28216300
C	8.12238200	-1.15379500	1.40189800
C	7.24873300	-0.51969200	0.52621000
C	4.73845100	-0.43716400	-0.19754100
C	3.64626900	-1.16174500	0.22742400
H	5.98390300	-3.30517700	2.99363500
H	8.36488400	-2.62147800	2.96755400
H	9.17073800	-0.87070400	1.41552900
H	7.59909900	0.26304300	-0.14076900
H	4.70150100	0.38310900	-0.90052300
C	3.24463100	-2.97268400	1.91362200
C	3.62637000	-4.30073500	2.11851400
C	1.99164800	-2.54041500	2.39491300
C	2.79185800	-5.17677900	2.81226600
H	4.56963600	-4.64365700	1.70895500
C	1.15208500	-3.42155600	3.07409500
C	1.55759200	-4.73957900	3.29068700
H	3.10200000	-6.20633100	2.96025600
H	0.18434300	-3.09139200	3.43184700
H	0.90041900	-5.42053100	3.82193600
N	4.07910300	-2.07633700	1.21087100
S	1.56071500	-0.83000700	2.06920400

C	-0.18152900	-0.70402200	2.54863900
H	-0.25879400	-0.75368200	3.63655500
H	-0.49200300	0.27469400	2.19177500
H	-0.78682300	-1.46350000	2.06140300
C	-0.44899100	4.41704800	-2.79644200
C	-0.62487900	3.73095900	-4.15956700
C	-0.46399800	5.94902600	-2.95426500
H	-1.31009900	4.13198700	-2.18564000
H	-0.59097600	2.64665300	-4.04071700
H	-1.59689200	4.00721900	-4.58542200
H	0.14200500	4.04447800	-4.87855100
H	-0.40168900	6.45782100	-1.98663100
H	0.38844300	6.28191300	-3.55913800
H	-1.38312600	6.27721100	-3.45426200
C	4.42028900	3.17057800	-3.00437100
C	4.30474500	1.81016700	-3.72091500
C	5.74853200	3.26802400	-2.24402800
H	4.42812500	3.94545000	-3.78378100
H	3.36306300	1.74021600	-4.27510600
H	5.13666600	1.66113100	-4.42029800
H	4.32600700	0.99031700	-2.99434300
H	5.83454900	4.21919400	-1.70810200
H	5.85475500	2.46028300	-1.50960600
H	6.59159100	3.18774200	-2.93927000
C	2.03397800	3.16265700	1.50123700
C	1.99919000	4.52320100	2.22471600
C	3.23368400	2.34784500	2.00350600
H	1.12563200	2.61508300	1.78041900
H	1.10876100	5.09829100	1.95899100
H	2.00251300	4.38230500	3.31252000
H	2.88150600	5.11619300	1.95609400
H	3.35575700	1.41207800	1.45309600
H	4.16840600	2.91422800	1.91432700
H	3.10209900	2.10583900	3.06395600
C	-1.77803500	-3.58283200	0.36958400
C	-0.50789600	-4.01498600	-0.38236300
C	-2.17583400	-4.64461300	1.40915900
H	-1.53036300	-2.66115600	0.89601300

H	-0.26201600	-3.28847300	-1.15908500
H	0.33484300	-4.07512900	0.31695400
H	-0.62191200	-5.00461200	-0.84036100
H	-3.02127800	-4.31479600	2.02178600
H	-2.46854300	-5.57852600	0.91496100
H	-1.33337700	-4.86767600	2.07568800
C	-5.65859200	-0.59761000	-1.21256200
C	-7.12946200	-0.98526200	-0.98238100
C	-5.50070300	0.31425000	-2.44117300
H	-5.34276000	-0.02130000	-0.33933000
H	-7.23699200	-1.60704600	-0.08646100
H	-7.74655100	-0.08807500	-0.85407600
H	-7.53307700	-1.54933200	-1.83111100
H	-4.45533300	0.60613100	-2.57133500
H	-5.83512200	-0.18980600	-3.35566900
H	-6.10317100	1.22269200	-2.32110700
C	-4.53433900	-4.85907600	-3.66192200
C	-4.92361900	-6.25464400	-3.14619900
C	-3.34046100	-4.94029900	-4.62866300
H	-5.39039800	-4.46021400	-4.22254800
H	-5.79013600	-6.19975500	-2.47850100
H	-5.17187300	-6.92244000	-3.97972000
H	-4.09906000	-6.71140500	-2.58633100
H	-3.08045300	-3.94884300	-5.01400600
H	-2.45483500	-5.34470400	-4.12462100
H	-3.57110800	-5.59360000	-5.47873200

int4-R

C	7.02312400	-3.10360900	-1.19446300
C	6.09650000	-2.20663800	-0.71254500
C	4.72353700	-2.55802400	-0.61001500
C	4.31981100	-3.85735100	-1.05784100
C	5.30293100	-4.76355800	-1.53590800
C	6.62805600	-4.40028300	-1.60025700
H	8.06650700	-2.81103800	-1.26964600
H	6.40748900	-1.21301700	-0.41271400
C	3.72812500	-1.65326000	-0.11248200
C	2.94668700	-4.21197600	-1.02883400

H	4.98238100	-5.74950500	-1.86285900
H	7.37021500	-5.10007300	-1.97332100
C	1.97288500	-3.31785700	-0.63952400
C	2.39598600	-2.03141700	-0.20794300
H	2.65814600	-5.21146900	-1.34034600
C	4.07200900	-0.33943200	0.49941800
C	4.95848100	-0.22891000	1.62388800
C	3.45575100	0.81194800	0.03944800
C	5.53567600	-1.35470700	2.27110000
C	5.25359600	1.07402300	2.14264200
C	3.64946600	2.09950900	0.60896900
C	6.39912900	-1.19759000	3.33232200
H	5.28523000	-2.34904400	1.92300100
C	6.16144700	1.20165700	3.22709600
C	4.59198800	2.20925300	1.60819000
C	6.73033000	0.09249700	3.80849000
H	6.82549100	-2.07402300	3.81199400
H	6.38319500	2.19810100	3.60077900
H	4.81074400	3.18590900	2.02815400
H	7.41814900	0.20236100	4.64186200
O	1.41082800	-1.13833000	0.18153400
O	2.61130800	0.71710600	-1.05592300
P	1.09598400	0.15729800	-0.80528500
O	0.48966400	-0.22048200	-2.11049600
O	0.38361100	1.13224000	0.12762900
C	0.51870000	-3.67514600	-0.62698900
C	-0.22788100	-3.65675100	-1.82284700
C	-0.10704400	-4.01058700	0.59757300
C	-1.58697800	-3.99584200	-1.77084600
C	-1.46481600	-4.33914000	0.59534300
C	-2.22307500	-4.34143400	-0.58078100
H	-2.17031700	-3.98174300	-2.68673700
H	-1.94803700	-4.58758900	1.53356900
C	2.82250200	3.26498500	0.16469500
C	1.78277100	3.74306200	1.00062700
C	3.05320200	3.86747100	-1.08635400
C	1.03787400	4.84612600	0.57860900
C	2.27292900	4.96666900	-1.46706400

C	1.26833300	5.47805200	-0.64754400
H	2.45611600	5.43884400	-2.42933700
H	0.24555000	5.21717900	1.22097000
Pd	-1.72202100	1.02403800	0.33705400
C	-4.76199800	-0.48680700	-2.53294400
C	-4.12410200	0.53976400	-1.82913000
C	-3.03062300	1.20257600	-2.42730800
C	-2.59974900	0.82974000	-3.70014300
C	-3.25631100	-0.18618000	-4.39469800
C	-4.33817000	-0.84262300	-3.81166400
H	-5.57460300	-1.01883500	-2.05211800
H	-1.74663400	1.31789400	-4.15321400
H	-2.90536400	-0.46996400	-5.38130000
H	-4.84148500	-1.64688900	-4.33910300
C	-5.86559200	1.02225500	-0.08789100
C	-3.68242400	1.09998600	0.56505700
C	-7.07088900	0.94315500	-0.78833600
C	-5.81667300	1.36113800	1.29261200
C	-4.42733900	1.40632500	1.67150500
C	-8.24723300	1.16764800	-0.07588800
H	-7.09683500	0.72606500	-1.85033000
C	-7.01785500	1.57689900	1.98464000
H	-4.03169700	1.61762500	2.65477500
C	-8.22247200	1.47217700	1.29726000
H	-9.19967600	1.10864200	-0.59451400
H	-7.00186900	1.83464800	3.04015200
H	-9.15822500	1.63865000	1.82321500
S	-2.22367600	2.51475700	-1.49053600
C	-0.64502200	2.75470300	-2.37120000
H	0.00106100	3.29383700	-1.67701600
H	-0.20345800	1.78642100	-2.61102600
H	-0.82475300	3.36144700	-3.26160700
C	-1.36487100	-0.63808500	1.80583100
C	-1.76398600	-1.20356200	0.60969800
H	-0.31611100	-0.45079500	2.01015900
H	-2.78400400	-1.53558900	0.48514100
H	-1.05300900	-1.49933700	-0.15082700
C	-2.27128100	-0.53143600	2.98265400

O	-2.05012000	0.17653600	3.94615700
O	-3.34764600	-1.33102300	2.85385100
C	-4.33727900	-1.27443100	3.90627900
H	-5.27897300	-1.50506600	3.40434200
H	-4.37671600	-0.25651600	4.29801700
C	-4.00478300	-2.27782800	4.99793900
H	-3.91647900	-3.28704500	4.58256000
H	-3.06167500	-2.01285500	5.48438900
H	-4.79573200	-2.28332400	5.75623500
N	-4.54179500	0.87037600	-0.52380300
C	4.13021900	3.36507100	-2.03747500
C	5.22083400	4.42831000	-2.25305800
C	3.52642100	2.89926200	-3.37322500
H	4.61218900	2.49785300	-1.57816400
H	5.67026500	4.72815300	-1.29992400
H	6.01557100	4.03880500	-2.90013900
H	4.81309900	5.32826200	-2.72809400
H	2.78159000	2.11624800	-3.20686600
H	3.04708800	3.72990500	-3.90518400
H	4.30931500	2.49491800	-4.02566300
C	0.45042300	6.68703900	-1.07011300
C	0.69340600	7.87710700	-0.12569300
C	-1.05051300	6.36355000	-1.16723600
H	0.79376700	6.98044700	-2.07137300
H	1.75868300	8.12578800	-0.07430300
H	0.14723100	8.76405300	-0.46784500
H	0.35499300	7.64553300	0.89086900
H	-1.23928600	5.56396600	-1.89093600
H	-1.45018000	6.03797800	-0.19998600
H	-1.61869700	7.24629800	-1.48267200
C	1.43843800	3.08396400	2.33298800
C	-0.07396900	3.05866600	2.62004700
C	2.15708300	3.77580600	3.50694700
H	1.77921200	2.04649900	2.28251700
H	-0.64238100	2.77225700	1.72934800
H	-0.30394300	2.33653700	3.40968000
H	-0.44417400	4.03729100	2.94859200
H	3.24417500	3.72914900	3.40663700

H	1.86900000	4.83259800	3.56215500
H	1.88372700	3.29990700	4.45610100
C	0.41306600	-3.31830100	-3.16292200
C	-0.48455100	-2.45399900	-4.06000500
C	0.82521000	-4.60755900	-3.89768100
H	1.31452100	-2.73406500	-2.96068900
H	-0.79998500	-1.55493700	-3.53045600
H	0.07628200	-2.14732000	-4.95079700
H	-1.37076300	-2.99994100	-4.40578300
H	1.52477600	-5.20570100	-3.30589200
H	-0.05320100	-5.23041800	-4.10676300
H	1.30679900	-4.36813100	-4.85317200
C	0.68927200	-4.04368500	1.89803000
C	1.46353600	-5.37000500	2.02424300
C	-0.16299500	-3.81939600	3.15576000
H	1.42413000	-3.23249600	1.85672900
H	2.14935600	-5.51441400	1.18537200
H	2.04953500	-5.38740500	2.95088600
H	0.76639100	-6.21614600	2.04525900
H	-0.80966700	-2.94287600	3.05986100
H	-0.80177800	-4.68379100	3.37236300
H	0.48774200	-3.67134200	4.02413400
C	-3.70700100	-4.67398000	-0.56540400
C	-4.53402900	-3.51390800	0.01909400
C	-4.00475300	-5.98647400	0.17642700
H	-4.01781300	-4.80555100	-1.61061700
H	-4.39875200	-2.60198700	-0.57233800
H	-5.60307600	-3.75860200	0.02180500
H	-4.23470700	-3.29379400	1.05033300
H	-3.41503800	-6.81387300	-0.23209900
H	-3.77136300	-5.90364100	1.24410700
H	-5.06670500	-6.24400900	0.09064800

TS5-R

C	6.85013200	-3.19326800	-1.16514400
C	5.93416700	-2.25172100	-0.75302500
C	4.56491000	-2.58981700	-0.57758800
C	4.15346200	-3.92730700	-0.88323500

C	5.12598700	-4.87830200	-1.29100800
C	6.44839200	-4.52479900	-1.42534800
H	7.89025100	-2.90944700	-1.29783900
H	6.25118300	-1.23293900	-0.56571900
C	3.58096600	-1.63888700	-0.14588000
C	2.78104600	-4.27355800	-0.79635600
H	4.79856600	-5.89183600	-1.50884600
H	7.18235400	-5.25939800	-1.74389300
C	1.81609900	-3.34273100	-0.47879900
C	2.24679900	-2.02317300	-0.17342500
H	2.48629800	-5.29614500	-1.01201400
C	3.94362200	-0.26934400	0.31226900
C	4.87774400	-0.03838900	1.37817900
C	3.31075200	0.82696500	-0.24731600
C	5.47493100	-1.08715900	2.12783000
C	5.20323700	1.31265900	1.72753600
C	3.53672400	2.17020900	0.15865500
C	6.38505300	-0.81748200	3.12548500
H	5.20092100	-2.11206700	1.91068600
C	6.15874000	1.55547200	2.74949700
C	4.52543300	2.38548000	1.09368600
C	6.74556600	0.51541100	3.43206100
H	6.82518700	-1.63679600	3.68683500
H	6.40262900	2.58623900	2.99426600
H	4.76677900	3.40105600	1.39053900
H	7.46994300	0.71406700	4.21672700
O	1.26653900	-1.10663000	0.15852700
O	2.41702400	0.61706300	-1.28394000
P	0.90810300	0.11343500	-0.90486100
O	0.23352700	-0.35280500	-2.14682700
O	0.27228000	1.18846900	-0.02305700
C	0.35626100	-3.67541500	-0.44448200
C	-0.38252400	-3.70362400	-1.64546800
C	-0.27921200	-3.93666500	0.79203300
C	-1.75230800	-3.98517800	-1.58263200
C	-1.64454400	-4.23694400	0.79800800
C	-2.40118300	-4.25897100	-0.37920100
H	-2.33314800	-3.99667000	-2.49980100

H	-2.12982200	-4.45979700	1.74237300
C	2.67501600	3.27292400	-0.36903100
C	1.71814700	3.87540200	0.48513300
C	2.77101400	3.67931300	-1.71405000
C	0.91773700	4.90094300	-0.02210700
C	1.93610600	4.70344500	-2.17725900
C	1.00865500	5.33264800	-1.34855500
H	2.01564000	5.02389800	-3.21334900
H	0.19227000	5.37046800	0.63460400
Pd	-1.75097900	0.87424300	0.44465300
C	-4.85054600	-1.24567800	-1.98230600
C	-4.24668900	-0.10612200	-1.44938400
C	-3.30467500	0.61204200	-2.21469200
C	-2.97885000	0.16360000	-3.49500000
C	-3.59384700	-0.97346700	-4.01935400
C	-4.53239700	-1.67838700	-3.26861700
H	-5.56223300	-1.78926800	-1.37059100
H	-2.23931700	0.68927600	-4.08520300
H	-3.32234200	-1.31128300	-5.01400600
H	-5.00566900	-2.56772300	-3.67256400
C	-5.86605300	0.78526500	0.21349400
C	-3.70557100	0.59670000	0.91086300
C	-7.05984500	0.78812800	-0.51388500
C	-5.77326600	1.34424000	1.52084600
C	-4.41372100	1.20611900	1.93471700
C	-8.18068600	1.33225000	0.10274300
H	-7.10712000	0.38339200	-1.51903600
C	-6.92965500	1.87950500	2.11965000
H	-3.98926400	1.45460200	2.89676000
C	-8.11968500	1.86649700	1.40823100
H	-9.12514500	1.34687100	-0.43330100
H	-6.88179600	2.30461700	3.11810500
H	-9.01942400	2.27917300	1.85472800
S	-2.58595400	2.09401400	-1.49305200
C	-1.15056000	2.43812700	-2.56308200
H	-0.50406700	3.09915100	-1.98329800
H	-0.61923400	1.50792300	-2.77034800
H	-1.48677400	2.94102300	-3.47254300

C	-1.32048500	-0.63366300	1.88412600
C	-2.67099500	-1.03818800	1.70430700
H	-0.54595700	-1.14685500	1.32305500
H	-3.34071600	-1.06162100	2.55152500
H	-2.86737000	-1.75872800	0.91982300
C	-0.83874900	-0.04609500	3.15523200
O	0.32851800	0.00139400	3.48508900
O	-1.85899900	0.42683300	3.92310800
C	-1.48629100	0.94949900	5.21744500
H	-2.30035000	1.62845100	5.48173300
H	-0.55904100	1.51697800	5.11394500
C	-1.33389200	-0.17170700	6.23313300
H	-2.25391500	-0.76184500	6.30040400
H	-0.50918900	-0.83096300	5.94932600
H	-1.11771000	0.24568000	7.22303300
N	-4.60260500	0.32102600	-0.14203500
C	3.76986600	3.05566400	-2.67891600
C	4.83391300	4.08195200	-3.10586300
C	3.06988700	2.43262100	-3.89832100
H	4.29174100	2.25016000	-2.15605200
H	5.35497500	4.49251900	-2.23385200
H	5.57790600	3.61553700	-3.76255500
H	4.38324800	4.91972000	-3.65092000
H	2.33607000	1.68614600	-3.58322800
H	2.55779900	3.19492700	-4.49785300
H	3.80375900	1.93985200	-4.54706200
C	0.14298800	6.47016100	-1.86488300
C	0.53879900	7.80181200	-1.20257000
C	-1.35915300	6.19536100	-1.68078000
H	0.33517300	6.56388100	-2.94235600
H	1.60129800	8.01632300	-1.35874000
H	-0.04633200	8.63294900	-1.61404900
H	0.36092900	7.76656200	-0.12138500
H	-1.66333800	5.27845700	-2.19589800
H	-1.61613500	6.08249300	-0.62141100
H	-1.95589200	7.02323100	-2.08117800
C	1.52786900	3.44022600	1.93477700
C	0.05180300	3.38967800	2.36116100

C	2.29942700	4.36421700	2.89593800
H	1.92083200	2.42579200	2.03729900
H	-0.54426100	2.81109100	1.65083600
H	-0.02659300	2.91024000	3.34202500
H	-0.38960700	4.38971000	2.44812300
H	3.37016300	4.38164500	2.67658500
H	1.92733700	5.39344200	2.82038300
H	2.17214700	4.03173300	3.93314300
C	0.29017400	-3.50676700	-2.99874700
C	-0.56285600	-2.72058000	-4.00188100
C	0.68383700	-4.87304200	-3.59252200
H	1.20230100	-2.92687600	-2.83575300
H	-0.85870500	-1.76404300	-3.57303200
H	0.02355300	-2.52885800	-4.90830800
H	-1.45785600	-3.27718500	-4.30592800
H	1.35006600	-5.42991500	-2.92733600
H	-0.20790200	-5.48863400	-3.76387600
H	1.19540100	-4.74124000	-4.55340400
C	0.52285900	-3.94461400	2.09167100
C	1.29286100	-5.27134600	2.24450300
C	-0.31614900	-3.70169800	3.35465000
H	1.25791800	-3.13393400	2.03217200
H	1.97835700	-5.43780400	1.41050300
H	1.87869200	-5.27052800	3.17137800
H	0.59180200	-6.11375400	2.28460900
H	-0.95082900	-2.81709400	3.26304500
H	-0.96013000	-4.55920400	3.58400000
H	0.34678400	-3.55159200	4.21311100
C	-3.86876400	-4.66066300	-0.38065300
C	-4.71443100	-3.93439600	0.67649100
C	-3.99792100	-6.18674200	-0.21892800
H	-4.27306800	-4.39408800	-1.36661000
H	-4.69600300	-2.84982300	0.53210600
H	-5.75914200	-4.26202200	0.62137800
H	-4.35964300	-4.14481000	1.69186700
H	-3.42945800	-6.71094000	-0.99415700
H	-3.60658100	-6.50552600	0.75440800
H	-5.04619800	-6.50260500	-0.28372600

int6-R

C	-6.67326300	-0.84078700	-2.94449400
C	-5.70695100	-1.00053900	-1.97647200
C	-4.89357100	0.09073700	-1.56939100
C	-5.07531300	1.35291300	-2.22111600
C	-6.09016700	1.48740500	-3.20475600
C	-6.87848400	0.41695300	-3.55826700
H	-7.27976000	-1.69077400	-3.24367500
H	-5.55369100	-1.97101400	-1.52075500
C	-3.87355000	-0.02935100	-0.56569400
C	-4.22795700	2.44112000	-1.88886700
H	-6.22232200	2.45465800	-3.68287300
H	-7.64824300	0.53029100	-4.31608000
C	-3.19488500	2.30960500	-0.98765600
C	-3.03754000	1.04904200	-0.34997400
H	-4.39069300	3.39732300	-2.37679000
C	-3.66607500	-1.28628200	0.19844500
C	-4.72279300	-1.88547900	0.96280400
C	-2.42803300	-1.90817100	0.18876900
C	-5.97260400	-1.24448600	1.17738800
C	-4.50325700	-3.16491000	1.56625400
C	-2.20245500	-3.19677600	0.74137000
C	-6.96060900	-1.85085500	1.92040400
H	-6.13877000	-0.26027400	0.75603900
C	-5.54764800	-3.76885100	2.31495100
C	-3.24902000	-3.80405800	1.40149900
C	-6.75382800	-3.13048200	2.48748100
H	-7.90575700	-1.33912300	2.07770100
H	-5.36821000	-4.74456100	2.75914200
H	-3.10579600	-4.79834700	1.81493100
H	-7.54366300	-3.59939400	3.06710500
O	-2.00063600	0.91506700	0.57144500
O	-1.35893900	-1.30942300	-0.46768500
P	-0.68611500	0.07277000	0.08645600
O	0.04972700	0.70922400	-1.07728900
O	0.19971200	-0.11102100	1.31835500
C	-2.27249700	3.42763800	-0.63000800

C	-1.23348900	3.80818600	-1.50017000
C	-2.43572800	4.06996300	0.62024700
C	-0.34720700	4.80803500	-1.07807300
C	-1.50938700	5.03865000	1.00529900
C	-0.44257900	5.40527800	0.17732800
H	0.44328600	5.13101000	-1.75012800
H	-1.61611000	5.51887700	1.97217400
C	-0.90865200	-3.91321100	0.53368600
C	-0.00721100	-4.08872000	1.60392200
C	-0.63458800	-4.47901700	-0.72857800
C	1.14209500	-4.85831300	1.39593100
C	0.52809400	-5.24031300	-0.88541600
C	1.42592600	-5.45003000	0.16214400
H	0.73422000	-5.69967000	-1.84945400
H	1.83110900	-5.00166900	2.22353200
Pd	1.89795400	0.86219200	0.26185200
C	6.89229600	0.17560200	-0.25702000
C	5.55568200	0.40434300	-0.58094800
C	5.09338400	1.72416700	-0.74861600
C	5.98143500	2.79117400	-0.59053400
C	7.31692800	2.54765900	-0.26440000
C	7.77614700	1.24272400	-0.09585400
H	7.21886700	-0.85141600	-0.13086300
H	5.64477200	3.81302100	-0.71498400
H	7.99515600	3.38662200	-0.14186300
H	8.81412400	1.05469800	0.15951500
S	3.37098100	1.96545900	-1.19992200
C	3.09292700	3.69916300	-0.69828300
H	2.01489400	3.84778000	-0.76637200
H	3.41534300	3.85109500	0.33305400
H	3.60168800	4.38358700	-1.38112600
C	3.87074400	-1.23013100	0.30890600
C	3.90809000	-0.66758400	1.69685200
H	3.30173600	-1.30569000	2.34172800
H	4.92991900	-0.67030900	2.10362300
C	3.33383600	0.75296400	1.73114800
H	4.07171000	1.52327500	1.50687600
C	2.61111900	1.10394700	2.98804400

O	2.30633600	0.33121200	3.87391600
O	2.31752300	2.43201300	3.01219800
C	1.39816200	2.84674300	4.04784700
H	1.65479300	2.32733100	4.97388900
H	1.58265400	3.91674900	4.16609700
C	-0.03212500	2.55978000	3.62287000
H	-0.17343800	1.49196500	3.43803500
H	-0.72112900	2.87581500	4.41446800
H	-0.28075700	3.10034800	2.70477000
N	4.66612300	-0.69075200	-0.71833900
C	4.29538400	-1.27146700	-1.92980400
C	3.02101500	-2.15973300	-0.23392400
H	2.27606500	-2.71728400	0.31443600
C	3.26872400	-2.21267800	-1.64970600
C	4.77819000	-1.04074400	-3.21824100
H	5.56282800	-0.31337700	-3.40438600
C	4.20924700	-1.78030400	-4.25215400
H	4.55374100	-1.62374500	-5.27032900
C	3.19650200	-2.72681500	-3.99988000
H	2.77468400	-3.28669200	-4.82982500
C	2.72557400	-2.95210600	-2.71146400
H	1.94474500	-3.67716900	-2.51740800
C	-1.58382900	-4.31773400	-1.90941600
C	-2.14624100	-5.67605000	-2.36152100
C	-0.92130700	-3.56626500	-3.07566300
H	-2.43490000	-3.71523400	-1.58281600
H	-2.65529000	-6.18448700	-1.53517600
H	-2.86597600	-5.54101300	-3.17756200
H	-1.35116800	-6.33833900	-2.72313200
H	-0.52039200	-2.60445900	-2.74341900
H	-0.10032500	-4.14812500	-3.51079200
H	-1.65227300	-3.38037400	-3.87150200
C	-0.26275800	-3.48436500	2.97809000
C	0.90535700	-2.60424100	3.44481000
C	-0.58711300	-4.58278100	4.00541800
H	-1.13916300	-2.83436800	2.90154900
H	1.06770300	-1.78391600	2.74491800
H	0.68641300	-2.16064400	4.42200000

H	1.83396400	-3.17956800	3.54502500
H	-1.45399300	-5.17720900	3.69479300
H	0.25923500	-5.26910000	4.12866700
H	-0.80759300	-4.14095400	4.98413400
C	-3.65795400	3.77022700	1.48235100
C	-4.85911300	4.58966000	0.96994200
C	-3.45822600	4.01070700	2.98278600
H	-3.90631600	2.71137900	1.36022200
H	-5.07396300	4.36923500	-0.07964500
H	-5.75788800	4.36569400	1.55678600
H	-4.65297900	5.66322100	1.05626400
H	-2.59982500	3.45444500	3.36528400
H	-3.31218700	5.07267800	3.21315400
H	-4.34874200	3.68178000	3.52966000
C	0.58691100	6.42391200	0.63399100
C	1.30698900	5.95347200	1.91030800
C	-0.03645000	7.81539700	0.83351300
H	1.33868500	6.50747700	-0.16347700
H	1.73590700	4.95430300	1.78300400
H	2.11115700	6.64767100	2.18224700
H	0.60992700	5.90516200	2.75485800
H	-0.52059300	8.16443300	-0.08470900
H	-0.79542800	7.79378400	1.62406400
H	0.72693600	8.54782500	1.12169700
C	-1.09363000	3.21509400	-2.89674400
C	0.35722300	2.91892900	-3.30294100
C	-1.75615900	4.14867200	-3.92815400
H	-1.62619400	2.26053800	-2.90815400
H	0.81315200	2.22583700	-2.59545100
H	0.37523900	2.44777200	-4.29222200
H	0.96376800	3.83060100	-3.36987100
H	-2.80813600	4.33402000	-3.68925200
H	-1.24666900	5.11977300	-3.95362700
H	-1.70543500	3.71231000	-4.93265500
C	2.65519400	-6.32216600	-0.03251500
C	2.56350300	-7.59565800	0.82645100
C	3.96260500	-5.56255000	0.24522000
H	2.66945700	-6.63117000	-1.08639900

H	1.64654800	-8.15365700	0.60805300
H	3.42105700	-8.25305200	0.64013500
H	2.55668500	-7.34703500	1.89420800
H	4.05765800	-4.68760000	-0.40335300
H	4.00430100	-5.21348400	1.28365700
H	4.82693500	-6.21626900	0.07766500

TS7-R

C	5.63967700	-4.29738300	2.39977200
C	4.62833300	-3.90032500	1.55373900
C	4.53211400	-2.55356900	1.11192900
C	5.49121000	-1.60742300	1.60001200
C	6.52971900	-2.05368900	2.45950800
C	6.60899500	-3.37044500	2.84963300
H	5.69041300	-5.33130100	2.72894500
H	3.88728600	-4.61761500	1.22232700
C	3.49814100	-2.09592700	0.22746000
C	5.37680700	-0.24093100	1.23621200
H	7.25576100	-1.32642500	2.81382400
H	7.40498600	-3.69837000	3.51185400
C	4.33326000	0.22019000	0.46307500
C	3.40389100	-0.73889100	-0.01543500
H	6.12117300	0.46264000	1.59732400
C	2.51945300	-3.02983300	-0.38984500
C	2.94280500	-4.14207500	-1.19220400
C	1.16068200	-2.83484800	-0.20477900
C	4.29559500	-4.33621700	-1.58071400
C	1.96154500	-5.07534500	-1.65643800
C	0.17554500	-3.77930300	-0.60138200
C	4.66034800	-5.41194800	-2.35881400
H	5.04208000	-3.61675600	-1.26657900
C	2.37309600	-6.18186500	-2.44516100
C	0.60073600	-4.88405100	-1.30790400
C	3.69421900	-6.35216500	-2.78819700
H	5.69907800	-5.53659200	-2.65086900
H	1.61752100	-6.88714200	-2.78158500
H	-0.12596600	-5.63918400	-1.59316500
H	3.99696000	-7.19894000	-3.39726600

O	2.38452000	-0.27264700	-0.83118100
O	0.72381200	-1.69061000	0.46430100
P	0.84470000	-0.24793200	-0.30810200
O	0.52650300	0.84142500	0.70017400
O	-0.00990300	-0.16126800	-1.56646600
C	4.17554700	1.66409300	0.11701100
C	3.77380700	2.58495700	1.10539100
C	4.43641900	2.10394100	-1.19914500
C	3.70271300	3.94200700	0.76892800
C	4.32720200	3.46425600	-1.49052800
C	3.97254800	4.40397600	-0.51964200
H	3.42425100	4.66073600	1.53557900
H	4.53135900	3.80004600	-2.50304800
C	-1.23872800	-3.67405800	-0.12213400
C	-2.24887500	-3.09032300	-0.91391800
C	-1.55218600	-4.22523400	1.14190500
C	-3.56054300	-3.07017000	-0.42426500
C	-2.87693900	-4.17893200	1.58345500
C	-3.89767800	-3.61075100	0.81754300
H	-3.12872900	-4.60192200	2.55276100
H	-4.33444900	-2.61760400	-1.03215600
Pd	-1.01240800	1.65346300	-0.85598300
C	-4.99662700	3.41455400	1.64733800
C	-4.45685500	2.26721700	1.07154400
C	-3.62075500	1.40358600	1.80563300
C	-3.31044700	1.74889700	3.12827400
C	-3.83804000	2.90918300	3.69566100
C	-4.68757700	3.74354100	2.96701900
H	-5.64024600	4.04687300	1.04357500
H	-2.65016600	1.12088300	3.71346100
H	-3.57954200	3.16068500	4.72023800
H	-5.09743300	4.64229000	3.41618000
S	-3.02317800	-0.02337800	0.95574500
C	-1.90949200	-0.77197400	2.17595900
H	-1.50849400	-1.65402300	1.68134900
H	-1.08490200	-0.09610100	2.40865000
H	-2.45552500	-1.07987600	3.07084400
C	-4.06169000	2.40617100	-1.43560800

C	-2.77078700	3.08729900	-1.44131400
H	-1.83521600	2.00338500	-2.15231900
H	-2.58234600	3.68618900	-2.33188400
C	-1.98699300	3.44555200	-0.30134500
H	-2.35740900	3.29567200	0.70260600
C	-0.97472300	4.52237700	-0.45736800
O	-0.59720500	4.98748600	-1.51836100
O	-0.53292400	4.90926400	0.75183800
C	0.53375500	5.88416100	0.76253200
H	1.44711000	5.37321500	0.45217500
H	0.30925100	6.66401700	0.03021200
C	0.64091400	6.43023900	2.17174800
H	0.83113900	5.62463000	2.88668900
H	1.46795300	7.14662400	2.22785200
H	-0.28087800	6.94326500	2.46453200
N	-4.73830900	1.95533800	-0.29344500
C	-5.84302800	1.22307400	-0.69754000
C	-4.74096500	1.96497900	-2.55545200
H	-4.41817700	2.12317100	-3.57587700
C	-5.86478300	1.20177100	-2.11968400
C	-6.80899000	0.57756000	0.07940700
H	-6.77119300	0.61273000	1.16284000
C	-7.79997800	-0.12299000	-0.59634200
H	-8.55915200	-0.65079100	-0.02709000
C	-7.83539300	-0.17322700	-2.00841700
H	-8.62405200	-0.73685500	-2.49769200
C	-6.88359100	0.48299400	-2.77397500
H	-6.91715100	0.44291100	-3.85883700
C	-0.47860700	-4.83426400	2.03879200
C	-0.91225900	-6.16867400	2.66496700
C	-0.03881600	-3.83360700	3.12401300
H	0.39730900	-5.04462500	1.41895000
H	-1.25103400	-6.87438500	1.89875200
H	-0.07159500	-6.62243400	3.20227100
H	-1.72722300	-6.03845500	3.38586000
H	0.34863800	-2.91203200	2.68020700
H	-0.88339800	-3.56999200	3.77248000
H	0.74808800	-4.26761400	3.75238600

C	-1.95031200	-2.55626900	-2.30871900
C	-2.76883400	-1.30917200	-2.67677500
C	-2.16741300	-3.66509800	-3.35552500
H	-0.89872400	-2.26186200	-2.33514200
H	-2.68456600	-0.53979800	-1.90725900
H	-2.39082100	-0.88795400	-3.61484600
H	-3.83209200	-1.53554300	-2.82069000
H	-1.52494800	-4.52996400	-3.16273800
H	-3.20890400	-4.00922700	-3.34434000
H	-1.94170800	-3.29285900	-4.36192800
C	4.84404500	1.14588000	-2.31055900
C	6.25972100	1.46073600	-2.82392700
C	3.81638400	1.14679200	-3.45550800
H	4.87249300	0.13329800	-1.89933300
H	6.99093600	1.42307900	-2.00871300
H	6.56092300	0.73611700	-3.58964500
H	6.30916200	2.46016500	-3.27141900
H	2.81991400	0.89646900	-3.08139500
H	3.76496400	2.12756500	-3.94272400
H	4.09504500	0.40960000	-4.21794700
C	3.90519400	5.88489400	-0.85359900
C	2.97299700	6.17297000	-2.04320500
C	5.31304200	6.45250900	-1.10771500
H	3.49803300	6.40063000	0.02754500
H	1.96847000	5.76958000	-1.88245500
H	2.88978600	7.25369800	-2.20988500
H	3.36546200	5.72851900	-2.96493200
H	5.96823500	6.28243000	-0.24652500
H	5.77367200	5.97083200	-1.97811900
H	5.26997800	7.53076500	-1.30260000
C	3.40949700	2.14612400	2.51803200
C	2.05339100	2.71416000	2.96860600
C	4.51948300	2.51726600	3.51665300
H	3.31309000	1.05679800	2.51521900
H	1.27404700	2.47007000	2.24317000
H	1.77370700	2.28623500	3.93904200
H	2.09609000	3.80218700	3.09338600
H	5.47613900	2.06150900	3.23993900

H	4.66645000	3.60363600	3.55002300
H	4.26061700	2.18002800	4.52746800
C	-5.31665300	-3.58497400	1.36920200
C	-6.39966600	-3.52820700	0.28439600
C	-5.49457200	-2.42485200	2.36735300
H	-5.45336900	-4.52187200	1.92801500
H	-6.26963500	-4.32701000	-0.45392300
H	-7.39165800	-3.63970500	0.73715400
H	-6.39060800	-2.56918200	-0.24382300
H	-4.75324300	-2.47859600	3.17249900
H	-5.36905000	-1.46427200	1.85920400
H	-6.49329400	-2.45254600	2.82016600

int8-R

C	-6.38458600	4.10161400	1.72813600
C	-5.18963300	3.75697700	1.13712600
C	-4.92937200	2.41782200	0.74032800
C	-5.92654100	1.42430300	1.00697400
C	-7.15403400	1.81634900	1.60396300
C	-7.38443700	3.12647000	1.95433000
H	-6.55898600	5.13087400	2.02861700
H	-4.42773000	4.51020900	0.97586700
C	-3.70155600	2.01575100	0.11623800
C	-5.66456100	0.06660200	0.69163000
H	-7.90474100	1.05269900	1.79117000
H	-8.32508800	3.41271200	2.41595000
C	-4.44815300	-0.34172700	0.18739200
C	-3.48126000	0.66264000	-0.07943100
H	-6.43616100	-0.67376800	0.88157000
C	-2.66950900	2.99511300	-0.31806900
C	-2.98825500	4.06707800	-1.21921500
C	-1.35461000	2.85495500	0.10044400
C	-4.26040800	4.20284700	-1.83870800
C	-1.97633700	5.02785500	-1.53912100
C	-0.32578400	3.77488200	-0.24257400
C	-4.52857100	5.25521400	-2.68509700
H	-5.02306500	3.45901100	-1.64481600
C	-2.29008700	6.11129700	-2.40177900

C	-0.67044900	4.86332300	-1.01450000
C	-3.54102300	6.23004800	-2.96078700
H	-5.50687100	5.33389900	-3.15063400
H	-1.51220100	6.83756800	-2.62374600
H	0.08393500	5.60893300	-1.24619900
H	-3.76849700	7.05891400	-3.62489700
O	-2.27506200	0.25413500	-0.62033300
O	-1.01956400	1.80678100	0.94367700
P	-0.94471300	0.27224600	0.33863300
O	-0.95775800	-0.68956500	1.47163900
O	0.21034300	0.25169300	-0.67682700
C	-4.14895000	-1.78051300	-0.07803800
C	-4.01641600	-2.68059600	1.00089600
C	-4.02020000	-2.24541200	-1.40487800
C	-3.82417800	-4.03817500	0.72279300
C	-3.81450700	-3.60871500	-1.62946200
C	-3.72965900	-4.52716600	-0.58158700
H	-3.74371600	-4.73971400	1.55004400
H	-3.72718000	-3.96066600	-2.65249000
C	1.07666400	3.60272600	0.24999300
C	2.08725400	3.13158900	-0.62007900
C	1.39820800	3.97154500	1.57266000
C	3.40330400	3.06573800	-0.14971700
C	2.73059700	3.89405300	1.99156000
C	3.75072400	3.45511200	1.14639000
H	2.98456300	4.19416400	3.00595200
H	4.17646300	2.70367300	-0.81773300
Pd	1.31133500	-1.51161700	-0.61556900
C	5.65506700	-3.15420700	1.28515400
C	4.83047800	-2.15083400	0.78275400
C	3.94766200	-1.45516600	1.63021400
C	3.88311900	-1.80626900	2.98101200
C	4.70804800	-2.81781400	3.47720300
C	5.59794100	-3.48962200	2.63861500
H	6.32714400	-3.66674200	0.60432800
H	3.19087900	-1.30173600	3.64412100
H	4.64858000	-3.08150400	4.52885600
H	6.23654800	-4.27430300	3.03096500

S	2.97045000	-0.19021300	0.85872800
C	1.96176100	0.47075400	2.21591100
H	1.43434600	1.31550600	1.77544300
H	1.23559000	-0.26274700	2.56670700
H	2.61234400	0.83997900	3.00988500
C	4.01878400	-2.19163700	-1.63287700
C	2.80739800	-2.96096800	-1.47863100
H	0.25709200	-2.18338500	-1.50990200
H	2.36356200	-3.29109900	-2.41397200
C	2.23082700	-3.46379900	-0.30838100
H	2.71696700	-3.39149800	0.65473400
C	1.13178200	-4.46971700	-0.38509900
O	0.67536800	-4.94406500	-1.40707100
O	0.71932000	-4.76924900	0.85637800
C	-0.46262200	-5.59880500	0.95366500
H	-1.29664300	-5.03904300	0.52486100
H	-0.30900600	-6.50408300	0.35950500
C	-0.68423200	-5.89886900	2.42140700
H	-0.81674500	-4.97226100	2.98660600
H	-1.58832700	-6.50629800	2.53925000
H	0.16099900	-6.45248600	2.84337800
N	4.88743800	-1.79803200	-0.59903800
C	5.83083300	-0.92754700	-1.12412400
C	4.43540900	-1.58787200	-2.80816200
H	3.93290400	-1.68930800	-3.76029600
C	5.56692100	-0.77395600	-2.51432700
C	6.87403400	-0.25653900	-0.48035700
H	7.05911500	-0.39130300	0.57967600
C	7.64787900	0.60105600	-1.25288100
H	8.45909100	1.14985700	-0.78419900
C	7.39901500	0.78027900	-2.63244300
H	8.02490700	1.46336500	-3.19840100
C	6.37332800	0.09918400	-3.26965500
H	6.18431300	0.24010100	-4.32966600
C	0.33843700	4.45868900	2.55247800
C	0.64987500	5.87463600	3.06485200
C	0.15606900	3.46678300	3.71437600
H	-0.61523700	4.51262600	2.02078900

H	0.75418500	6.57886200	2.23214500
H	-0.15576200	6.23116000	3.71720400
H	1.58118700	5.89923200	3.64245500
H	-0.14961500	2.48564700	3.33994700
H	1.08550500	3.34898500	4.28504400
H	-0.61713000	3.82356200	4.40495500
C	1.78796100	2.75570400	-2.06782200
C	2.55126800	1.51317000	-2.55049600
C	2.09201400	3.94170800	-3.00316200
H	0.72173200	2.52457400	-2.13713000
H	2.37641100	0.66113600	-1.89338900
H	2.20632900	1.23750500	-3.55386700
H	3.63196000	1.68366100	-2.61220000
H	1.50860700	4.82786600	-2.73906500
H	3.15411300	4.21042000	-2.94912900
H	1.85977300	3.68067300	-4.04253300
C	-4.11584300	-1.31726000	-2.60862500
C	-5.32283800	-1.67476500	-3.49324700
C	-2.80643800	-1.31530700	-3.41716200
H	-4.27688400	-0.29840100	-2.24799500
H	-6.25488900	-1.64090900	-2.91808100
H	-5.40896800	-0.96985800	-4.32870300
H	-5.22627700	-2.68218600	-3.91441700
H	-1.96271500	-1.03138000	-2.78249300
H	-2.60123200	-2.30414300	-3.84417500
H	-2.87174400	-0.59993800	-4.24588400
C	-3.60083000	-6.02051600	-0.83641500
C	-2.61599100	-6.36759700	-1.96343000
C	-4.98830300	-6.62843300	-1.11565600
H	-3.22545100	-6.47658400	0.09102200
H	-1.63509200	-5.90933900	-1.80658800
H	-2.48541800	-7.45441800	-2.02809700
H	-2.99268700	-6.03046400	-2.93600900
H	-5.68095500	-6.42578700	-0.29164100
H	-5.41792000	-6.19644100	-2.02725900
H	-4.92051800	-7.71450800	-1.25291600
C	-4.06822000	-2.22195100	2.45308100
C	-2.84546300	-2.70229600	3.25198500

C	-5.37710300	-2.66737000	3.12794300
H	-4.03983500	-1.12960300	2.46147500
H	-1.92537900	-2.36845000	2.76638100
H	-2.87537700	-2.28441600	4.26572700
H	-2.83077400	-3.79425200	3.34893100
H	-6.25435800	-2.28086400	2.59787800
H	-5.45613900	-3.76120500	3.14533900
H	-5.41965300	-2.30973000	4.16368500
C	5.18332100	3.39436600	1.65785600
C	6.22632200	3.73495100	0.58352800
C	5.49273400	2.02389000	2.28784000
H	5.26675200	4.14767500	2.45343200
H	6.00086900	4.69075400	0.09876300
H	7.22371900	3.80485200	1.03314500
H	6.27223600	2.96238100	-0.19151400
H	4.78984800	1.78947500	3.09502900
H	5.42012900	1.23379000	1.53511800
H	6.50663700	2.00711000	2.70628600

TS9-R

C	6.96360000	0.92823000	-2.72243900
C	5.87441000	1.22624200	-1.93414300
C	5.10624900	0.19563100	-1.33073300
C	5.46798800	-1.16644000	-1.58906800
C	6.60610600	-1.43775600	-2.39334400
C	7.34276400	-0.41591200	-2.94704600
H	7.53377600	1.73199000	-3.17946600
H	5.58675500	2.25851600	-1.77195300
C	3.96602100	0.46174800	-0.50166700
C	4.68485400	-2.21930600	-1.04558300
H	6.87614800	-2.47515100	-2.57354300
H	8.20747600	-0.63781700	-3.56575600
C	3.53229200	-1.97228800	-0.33360300
C	3.19422800	-0.61308300	-0.10181700
H	5.00286100	-3.24426100	-1.20824700
C	3.59891600	1.82589500	-0.03636300
C	4.53327100	2.63921900	0.69220100
C	2.30960900	2.30547000	-0.21414100

C	5.82512500	2.18142000	1.07044700
C	4.14394500	3.95832400	1.09119600
C	1.88668600	3.58606000	0.23662200
C	6.69653800	2.99995300	1.75316500
H	6.12033400	1.16979700	0.82313200
C	5.07209400	4.78356600	1.77975400
C	2.82253500	4.40050200	0.83564900
C	6.32669100	4.32070700	2.09980900
H	7.67603600	2.62424200	2.03468100
H	4.76130100	5.78574000	2.06394700
H	2.53341200	5.39949400	1.14918100
H	7.02691500	4.95743000	2.63262700
O	2.02551900	-0.36561600	0.61243800
O	1.38114900	1.54403300	-0.91260800
P	0.76427600	0.14585200	-0.28709300
O	0.39923200	-0.74267100	-1.44577100
O	-0.36422500	0.43549200	0.69670400
C	2.67340300	-3.03872600	0.26449500
C	1.78088600	-3.78771900	-0.53243400
C	2.72182100	-3.24216600	1.66039600
C	0.95858500	-4.73147400	0.09334800
C	1.89016000	-4.20538500	2.23256100
C	0.99649900	-4.95867100	1.47003600
H	0.25575100	-5.30325500	-0.50247900
H	1.93678800	-4.36647600	3.30596200
C	0.46740800	4.02505000	0.06027800
C	-0.48348000	3.74364200	1.07032200
C	0.08453100	4.71265100	-1.10947000
C	-1.81098600	4.12983100	0.85993000
C	-1.25631800	5.08470000	-1.26891100
C	-2.21977000	4.79143800	-0.30237200
H	-1.55197100	5.60972800	-2.17303300
H	-2.55798500	3.90227900	1.61387600
Pd	-1.44119900	-1.69160200	-0.09254000
C	-5.84038200	-1.07045200	-1.22942900
C	-4.79353200	-0.31244200	-0.70970600
C	-3.92043500	0.35482600	-1.58002800
C	-4.07355000	0.24683200	-2.96078000

C	-5.11176200	-0.53605900	-3.47063400
C	-5.99830700	-1.18505100	-2.61094400
H	-6.50102400	-1.58358500	-0.53888500
H	-3.39397600	0.74815600	-3.63884800
H	-5.22465600	-0.63340800	-4.54568000
H	-6.80641300	-1.78661500	-3.01429700
S	-2.71549700	1.43385000	-0.80534600
C	-1.55617200	1.79717600	-2.15014900
H	-0.74589800	2.34914900	-1.67811700
H	-1.17653500	0.86774600	-2.57641400
H	-2.05108700	2.43897500	-2.87796600
C	-3.83177200	-1.11275600	1.48636900
C	-3.13597700	-2.30144400	1.00839400
H	-1.84345800	0.51134200	-0.10113000
H	-2.78348800	-2.93185300	1.82273800
C	-3.17964300	-2.91843200	-0.26783000
H	-3.77342100	-2.54027300	-1.08746500
C	-2.81012400	-4.34048100	-0.39143900
O	-2.46164400	-5.08437500	0.50960000
O	-2.92975600	-4.75285500	-1.68028600
C	-2.58379900	-6.12574400	-1.93103200
H	-1.56796300	-6.31167000	-1.56917600
H	-3.25850900	-6.77458900	-1.36183200
C	-2.69789600	-6.35371200	-3.42600000
H	-2.00990400	-5.69768300	-3.96859500
H	-2.45020000	-7.39318100	-3.66720800
H	-3.71587500	-6.15066100	-3.77447600
N	-4.57353300	-0.21770700	0.68652600
C	-4.95216100	0.88116800	1.45793500
C	-3.77989500	-0.59086200	2.76416400
H	-3.25427900	-1.05056200	3.58948300
C	-4.46128900	0.66753700	2.77412700
C	-5.64724500	2.03156200	1.08556800
H	-6.01203900	2.16735000	0.07193800
C	-5.84313300	3.00313800	2.06511600
H	-6.37774800	3.91405900	1.81229100
C	-5.36509800	2.81885200	3.37841200
H	-5.53512500	3.59570900	4.11809100

C	-4.68494000	1.66212700	3.74219200
H	-4.31710000	1.53112400	4.75556400
C	0.11722200	-6.01597500	2.11361500
C	-0.74068400	-5.43588300	3.24835800
C	0.96549100	-7.20078800	2.60854100
H	-0.57189400	-6.37978500	1.34544800
H	-1.36122000	-4.61893500	2.87185000
H	-1.40421200	-6.20648400	3.65827600
H	-0.12074400	-5.05650100	4.06959000
H	1.55127800	-7.63583300	1.79091300
H	1.66836700	-6.88334200	3.38845700
H	0.32742800	-7.98669300	3.03075000
C	3.65516000	-2.45044600	2.56846100
C	4.67551500	-3.37290400	3.25726800
C	2.86731500	-1.61856100	3.59531000
H	4.22590100	-1.74993900	1.95270600
H	5.25836000	-3.93404000	2.51834900
H	5.37062200	-2.78822100	3.87178500
H	4.17943700	-4.09880900	3.91172900
H	2.15919000	-0.95507900	3.09130100
H	2.30350200	-2.26377500	4.27913000
H	3.55010200	-1.00757000	4.19829800
C	1.72475600	-3.61550600	-2.04544500
C	0.30781600	-3.73860400	-2.62378800
C	2.65360800	-4.63288300	-2.73556200
H	2.07953100	-2.60819800	-2.27806100
H	-0.39828000	-3.09546500	-2.09239000
H	0.31198200	-3.44203900	-3.67932600
H	-0.06107600	-4.76906200	-2.57668500
H	3.68975500	-4.53281800	-2.39938000
H	2.33048700	-5.65750500	-2.51410900
H	2.63499000	-4.49817900	-3.82406500
C	-0.06912900	3.10814400	2.39327200
C	-1.17194900	2.28018800	3.06526700
C	0.41986000	4.20154100	3.36448600
H	0.76795100	2.43287300	2.19145900
H	-1.56172200	1.51353900	2.39597400
H	-0.75750500	1.77288200	3.94384800

H	-2.00290400	2.90361000	3.41290500
H	1.26535400	4.76171700	2.95705500
H	-0.38805300	4.91228700	3.57673300
H	0.73625600	3.75290200	4.31329600
C	1.09107800	5.02060000	-2.21209000
C	1.01840000	6.48271100	-2.67928100
C	0.93083200	4.05110300	-3.39732700
H	2.09171400	4.86257300	-1.79980000
H	1.11993800	7.17304300	-1.83496100
H	1.82347500	6.69202100	-3.39278100
H	0.06998000	6.70512400	-3.18168900
H	1.07393900	3.01391200	-3.07923100
H	-0.06738100	4.14239800	-3.84426300
H	1.66782200	4.27199500	-4.17820400
C	-3.67321200	5.20394500	-0.47307000
C	-4.29404200	4.66900200	-1.77387000
C	-3.81877600	6.73347000	-0.38945200
H	-4.23259700	4.76451200	0.36192400
H	-4.27232700	3.57416700	-1.80536500
H	-5.34102400	4.98260700	-1.85813900
H	-3.76448900	5.04551200	-2.65686900
H	-3.40730300	7.11372800	0.55133300
H	-3.28216400	7.22349300	-1.21055700
H	-4.87270200	7.03004700	-0.45016500

int10-R

C	-5.90244600	-4.81606700	0.76830700
C	-5.21733900	-3.73498700	0.25921100
C	-3.81018800	-3.78265900	0.06971000
C	-3.11309600	-4.96188500	0.49306100
C	-3.85163500	-6.06489700	0.99731200
C	-5.21961400	-6.00188100	1.12394600
H	-6.97823000	-4.75303400	0.90386000
H	-5.75740400	-2.83308400	0.00292000
C	-3.04769300	-2.69538700	-0.48694400
C	-1.69465600	-4.96920600	0.51223200
H	-3.30690500	-6.95455200	1.30220800
H	-5.77361400	-6.84966400	1.51608800

C	-0.95802400	-3.87924800	0.10453100
C	-1.67293100	-2.80687900	-0.48747100
H	-1.18143400	-5.81972100	0.95095200
C	-3.68188400	-1.42236300	-0.91514300
C	-4.77976200	-1.39654900	-1.83912700
C	-3.25405100	-0.21428400	-0.37785600
C	-5.15539100	-2.53150300	-2.60688100
C	-5.50998200	-0.18039800	-2.02022100
C	-3.96522700	1.00425400	-0.54130600
C	-6.22049800	-2.47391100	-3.47686800
H	-4.58300100	-3.44621200	-2.50789900
C	-6.61727700	-0.15949800	-2.90931100
C	-5.10281600	0.98140400	-1.32001100
C	-6.96981600	-1.28194100	-3.62144200
H	-6.48470900	-3.34954600	-4.06242700
H	-7.16945500	0.76907100	-3.02773800
H	-5.69066500	1.88809000	-1.42029600
H	-7.81222600	-1.25327700	-4.30621900
O	-0.92860600	-1.78010300	-1.08382400
O	-2.10690100	-0.16676300	0.43489500
P	-0.64641300	-0.45583300	-0.22126100
O	0.39686200	-0.53905000	0.83418800
O	-0.39585000	0.59930100	-1.38334100
C	0.46731300	-3.68220400	0.49876800
C	0.72223700	-3.39303600	1.86379200
C	1.50897300	-3.67454500	-0.44180700
C	2.03843700	-3.17617500	2.25749000
C	2.81760700	-3.38271500	-0.00735500
C	3.10595000	-3.17322900	1.35141300
H	2.25499200	-2.99684200	3.30725800
H	3.62245500	-3.41373400	-0.73270000
C	-3.52264600	2.25643400	0.14447800
C	-2.82370700	3.24580700	-0.58310900
C	-3.82265400	2.45193100	1.50702000
C	-2.41301900	4.40283000	0.08285300
C	-3.40954000	3.64033100	2.12286400
C	-2.69450400	4.62047700	1.43505700
H	-3.63424500	3.79790500	3.17495800

H	-1.85533800	5.15434700	-0.46577900
Pd	2.57686000	-0.66554100	-0.13461400
C	4.74177800	2.41018000	1.15731800
C	3.42303100	2.54535000	0.73175600
C	2.35409000	2.34341500	1.62668600
C	2.64469300	1.93713600	2.93508100
C	3.96781900	1.76521000	3.34206600
C	5.02250600	2.01257500	2.46400600
H	5.53764400	2.60978800	0.44676700
H	1.84444500	1.75769700	3.64222800
H	4.17163800	1.44520000	4.35988300
H	6.05101000	1.89360200	2.78894200
S	0.71978200	2.70768900	1.03560700
C	-0.31857800	2.06928700	2.37907700
H	-1.33926100	2.10743200	2.00447400
H	-0.05427700	1.03573200	2.60026700
H	-0.24601500	2.69914600	3.26959600
C	2.81499700	2.06032900	-1.67289000
C	3.14464500	0.64319000	-1.74463000
H	0.27274900	1.26489800	-1.10381200
H	2.76151900	0.18069000	-2.65237900
C	4.29378100	0.02648000	-1.18172900
H	4.93254200	0.58296100	-0.50769300
C	4.99938200	-1.02765200	-1.93004700
O	4.58560100	-1.68083100	-2.87381900
O	6.26395100	-1.18561400	-1.44454200
C	7.07675100	-2.16037600	-2.12538300
H	6.58210500	-3.13597400	-2.07722700
H	7.15298900	-1.88215600	-3.18137100
C	8.42901900	-2.17765000	-1.43898600
H	8.32645100	-2.46158900	-0.38689500
H	9.08920300	-2.90066200	-1.93032600
H	8.90076000	-1.19081700	-1.48529800
N	3.13310000	2.92841600	-0.61099500
C	2.71629100	4.21492300	-0.94735800
C	2.22724700	2.80511300	-2.67905400
H	1.92654700	2.40790600	-3.63904100
C	2.16434900	4.17137000	-2.25492400

C	2.75754700	5.38486300	-0.18794800
H	3.16185700	5.38307900	0.81906700
C	2.24936400	6.54276500	-0.76937900
H	2.26504900	7.47189400	-0.20707000
C	1.72185600	6.53198000	-2.07737300
H	1.34320000	7.45618600	-2.50474100
C	1.67863700	5.36116400	-2.82442100
H	1.26186800	5.35819200	-3.82754800
C	-4.56986000	1.40475400	2.32387300
C	-5.84210400	1.98616900	2.96238500
C	-3.65774900	0.76024800	3.38328300
H	-4.88789400	0.60974800	1.64293100
H	-6.49677600	2.42738500	2.20293800
H	-6.40096000	1.20034200	3.48362500
H	-5.60587300	2.76661400	3.69452100
H	-2.79554100	0.27645200	2.91582200
H	-3.28684400	1.50892200	4.09347100
H	-4.20856400	0.00206900	3.95242200
C	-2.55959900	3.09537500	-2.07580700
C	-1.19481700	3.64650700	-2.50025300
C	-3.67127500	3.78184200	-2.89252800
H	-2.57336100	2.02771000	-2.31376100
H	-0.39059600	3.28587300	-1.85881500
H	-0.96899300	3.34279000	-3.52835400
H	-1.16430900	4.74034300	-2.47013200
H	-4.65742800	3.36162900	-2.67700200
H	-3.70573400	4.85353900	-2.66319300
H	-3.48189300	3.66951000	-3.96657700
C	1.25133200	-4.04039300	-1.89693900
C	2.04916300	-5.29793900	-2.28835900
C	1.54092000	-2.88789300	-2.87153200
H	0.18878800	-4.29386800	-1.98338800
H	1.83854400	-6.13135200	-1.60848900
H	1.79175400	-5.61017300	-3.30701700
H	3.12743700	-5.10419300	-2.26410000
H	0.93933700	-2.00961300	-2.62726400
H	2.59318300	-2.59612200	-2.85400500
H	1.29247900	-3.19789000	-3.89452600

C	4.51502900	-3.00824000	1.90849100
C	4.77974000	-1.55206900	2.32741900
C	5.61311100	-3.51454100	0.97025800
H	4.54926100	-3.62204400	2.82039300
H	4.02658700	-1.19655100	3.03709000
H	5.76764900	-1.45216900	2.79386000
H	4.74094600	-0.89099900	1.45524700
H	5.42551600	-4.54455100	0.64722800
H	5.69104200	-2.87847900	0.08698200
H	6.58338500	-3.48754900	1.47913800
C	-0.38195100	-3.29411400	2.91244200
C	-0.27749200	-2.00565600	3.74635300
C	-0.39544400	-4.53560600	3.82069600
H	-1.34409300	-3.25388700	2.39638600
H	-0.22803900	-1.13465600	3.08954700
H	-1.15288600	-1.90944600	4.39949800
H	0.61286200	-2.00230000	4.38560700
H	-0.53918800	-5.45310400	3.24046900
H	0.55249200	-4.63071600	4.36348000
H	-1.20353300	-4.46788700	4.55869900
C	-2.24337000	5.89528600	2.12777900
C	-2.94159100	7.12737500	1.52572800
C	-0.71416400	6.05994700	2.09389000
H	-2.55010500	5.82000400	3.17997400
H	-4.03131400	7.02525600	1.56992400
H	-2.65751900	8.03707200	2.06754500
H	-2.65965500	7.26169300	0.47500800
H	-0.21098900	5.20569000	2.55652500
H	-0.33783400	6.13525000	1.06798300
H	-0.41679700	6.96929200	2.62979800

int1-S

C	-6.77638400	1.45131000	2.65235700
C	-5.70432700	1.60526600	1.80220000
C	-5.00442900	0.47670100	1.29614300
C	-5.41618200	-0.82505900	1.72851300
C	-6.53564100	-0.94963700	2.59319500
C	-7.20793500	0.16229500	3.04372600

H	-7.29168200	2.32929300	3.03095200
H	-5.37886200	2.59871100	1.51982700
C	-3.88107600	0.59416500	0.40698800
C	-4.69231700	-1.96554500	1.29860300
H	-6.84157400	-1.94575200	2.90222000
H	-8.05918800	0.05543400	3.70967800
C	-3.56110700	-1.85737400	0.51929200
C	-3.16989100	-0.55711100	0.11215700
H	-5.03438200	-2.95158900	1.59872300
C	-3.45951900	1.91336200	-0.13691300
C	-4.34676700	2.77042700	-0.86978500
C	-2.16195800	2.34532800	0.05553900
C	-5.65163700	2.37280800	-1.26445500
C	-3.88446000	4.07245000	-1.25079100
C	-1.66970700	3.62236200	-0.31253300
C	-6.46995400	3.23010900	-1.96546600
H	-5.99701600	1.37692200	-1.01379300
C	-4.76040200	4.93633600	-1.96039700
C	-2.56121600	4.47341200	-0.93183900
C	-6.02757700	4.52949600	-2.30810800
H	-7.46217400	2.90360000	-2.26314800
H	-4.40098000	5.92448100	-2.23555200
H	-2.23611000	5.47202400	-1.20719200
H	-6.68542100	5.19744000	-2.85642500
O	-2.02313100	-0.48653200	-0.68619900
O	-1.26774800	1.48824200	0.69506800
P	-0.68581700	0.24274600	-0.15471700
O	0.17275300	-0.54912000	0.84689400
O	0.15810100	0.61045200	-1.37271500
C	-2.82758000	-3.07394100	0.05232400
C	-1.89910300	-3.71674600	0.89404100
C	-3.12140900	-3.60232000	-1.22240200
C	-1.34544700	-4.93098200	0.47358200
C	-2.51993800	-4.80145500	-1.60914300
C	-1.65032900	-5.49634700	-0.76501800
H	-0.65185300	-5.44688300	1.12939500
H	-2.74965200	-5.21090900	-2.58903800
C	-0.25978600	4.01883000	-0.01760300

C	0.64847600	4.25139500	-1.07347900
C	0.16045100	4.19268600	1.32087200
C	1.92814900	4.73215500	-0.77379900
C	1.45476100	4.65344100	1.56448900
C	2.34886000	4.95613000	0.53520500
H	1.77657600	4.80254600	2.59305900
H	2.61659800	4.91672700	-1.59027200
C	4.04993400	1.51099800	-1.35677100
O	3.33696500	0.43692700	-1.58014600
O	4.00244500	2.19441800	-0.33528500
C	5.01304600	1.81430600	-2.49125400
H	4.53609100	1.64919000	-3.46047800
H	5.36706700	2.84400400	-2.41292200
H	5.87032000	1.13584500	-2.41378600
Pd	1.91901800	-0.05251300	-0.25169200
C	2.72892200	-4.39161000	-0.16495900
C	3.88018700	-3.75805500	0.37501800
C	4.50103600	-4.18975900	1.54889600
C	3.95279700	-5.29535800	2.19034600
C	2.82441400	-5.95684900	1.66443400
C	2.21396300	-5.51864500	0.49602800
C	2.36616900	-3.66312700	-1.35235000
H	5.37723800	-3.68164300	1.94011300
H	4.40657100	-5.65904000	3.10765400
H	2.42839300	-6.82530100	2.18319800
H	1.35046600	-6.03589000	0.09614800
H	1.52163800	-3.85685300	-1.99817300
N	4.20860500	-2.70059300	-0.46891600
C	3.27579700	-2.65210700	-1.50936900
H	3.35141400	-1.86718400	-2.24626700
C	5.23787000	-1.75387600	-0.22493600
C	6.45957900	-1.84755900	-0.88701400
C	5.01751000	-0.70907700	0.68986900
C	7.46270200	-0.90850000	-0.64141700
H	6.60705700	-2.66164200	-1.58896500
C	6.01849100	0.22923900	0.93774200
C	7.24066400	0.12216600	0.27200200
H	8.41330200	-0.98397700	-1.16007300

H	5.84394800	1.05952500	1.60906500
H	8.01454400	0.85936500	0.46212400
S	3.39663200	-0.66654900	1.44562900
C	3.41391000	0.87792600	2.41974600
H	3.63040000	1.71545200	1.75582300
H	2.40197100	0.96892000	2.82015100
H	4.12958700	0.78258700	3.24025200
C	-4.05738700	-2.89273900	-2.19205300
C	-5.22194100	-3.79390400	-2.63348400
C	-3.27501500	-2.34878200	-3.40058600
H	-4.49661600	-2.03406500	-1.67633900
H	-5.78608000	-4.15923600	-1.76820800
H	-5.90963300	-3.23968500	-3.28292600
H	-4.86746700	-4.66641300	-3.19398700
H	-2.47786400	-1.67429800	-3.07360100
H	-2.81725300	-3.16463800	-3.97253100
H	-3.94105800	-1.79702200	-4.07472500
C	-1.50534200	-3.13076900	2.24382800
C	0.00341000	-3.24366000	2.51836700
C	-2.31579400	-3.77742600	3.38132400
H	-1.74941100	-2.06357100	2.22451300
H	0.58497900	-2.85484100	1.68043400
H	0.25974500	-2.66219000	3.41163900
H	0.31384900	-4.27824400	2.69962400
H	-3.39136300	-3.62632200	3.24355000
H	-2.12974300	-4.85750200	3.42171500
H	-2.03297300	-3.34800300	4.34985000
C	0.29047500	3.99283200	-2.53267800
C	1.32859400	3.09871300	-3.23287300
C	0.10738300	5.31476100	-3.29923300
H	-0.66082500	3.45441600	-2.55879300
H	1.44052300	2.14912900	-2.70714400
H	1.00488000	2.88715200	-4.25916400
H	2.30908200	3.58576500	-3.29183800
H	-0.66591700	5.94226200	-2.84284000
H	1.03919800	5.89279200	-3.30752300
H	-0.17971700	5.12236600	-4.33984300
C	3.69730200	5.56827400	0.88384700

C	3.51579700	7.06502400	1.20476600
C	4.77952700	5.36960300	-0.18301600
H	4.04392700	5.07326100	1.80271600
H	2.78248900	7.21641500	2.00420200
H	4.46540800	7.51655600	1.51679600
H	3.15800300	7.60234300	0.31820100
H	4.89069900	4.30849200	-0.41873700
H	4.53652300	5.91376800	-1.10408000
H	5.73785300	5.76069300	0.17877400
C	-0.74839800	3.92325400	2.51385400
C	-1.01483300	5.20921700	3.31539300
C	-0.17562000	2.81225200	3.41184700
H	-1.71648000	3.57559800	2.14526000
H	-1.45289100	5.98403000	2.67665900
H	-1.70845400	5.01187200	4.14144500
H	-0.09015200	5.61304900	3.74325800
H	-0.01945200	1.89522400	2.83644200
H	0.78132300	3.11551800	3.85365100
H	-0.86557600	2.58837500	4.23417500
C	-1.06923300	-6.83857900	-1.17837500
C	-0.18703800	-6.72372000	-2.43270200
C	-2.17781200	-7.88605600	-1.38149700
H	-0.43601700	-7.19001600	-0.35260500
H	0.64015700	-6.02419500	-2.27606500
H	0.23783900	-7.69862000	-2.69905200
H	-0.76875300	-6.36632200	-3.29012500
H	-2.79207600	-7.98734800	-0.48050800
H	-2.84045400	-7.60158900	-2.20700700
H	-1.74768400	-8.86619900	-1.61896300

TS2-S

C	-0.62531900	-1.53623900	4.94110900
C	-0.53931200	-0.56428600	3.96726000
C	-1.54743500	-0.43288600	2.97584000
C	-2.65560500	-1.33910000	3.00006300
C	-2.73039200	-2.30515600	4.03722500
C	-1.73711400	-2.41058700	4.98533700
H	0.16117900	-1.62700600	5.68526500

H	0.30981300	0.10975300	3.94005600
C	-1.47327700	0.54538800	1.93109400
C	-3.64964400	-1.25714100	1.98957700
H	-3.58595200	-2.97556700	4.05849200
H	-1.80350600	-3.16220700	5.76669400
C	-3.52092400	-0.40163200	0.91814600
C	-2.38477400	0.45227800	0.89535800
H	-4.52039500	-1.90432100	2.05262200
C	-0.49658400	1.66766100	1.95648300
C	-0.47184100	2.58898800	3.06005700
C	0.34864600	1.90552700	0.88453200
C	-1.39414600	2.52699000	4.14010900
C	0.49244000	3.64768100	3.05975900
C	1.33391300	2.93301600	0.88069000
C	-1.33895900	3.43401500	5.17507700
H	-2.15855100	1.76026200	4.13934900
C	0.52971800	4.55986500	4.14652900
C	1.38582300	3.77717400	1.96616000
C	-0.36149600	4.45576600	5.18916800
H	-2.05991900	3.36785200	5.98496700
H	1.27475800	5.35144300	4.13119900
H	2.11821400	4.57841400	1.98426000
H	-0.32658000	5.16195300	6.01364700
O	-2.22890200	1.27500200	-0.20716100
O	0.32005300	1.08393300	-0.21705200
P	-0.99784100	0.90831100	-1.23246400
O	-0.95079600	-0.60295000	-1.51111100
O	-0.98670100	1.87800800	-2.35064700
C	-4.55126800	-0.27768300	-0.15737700
C	-4.71430900	-1.28713900	-1.13024100
C	-5.35647100	0.88155500	-0.20060100
C	-5.68593500	-1.11867700	-2.12024400
C	-6.31273100	1.00194700	-1.21243600
C	-6.49319400	0.01794300	-2.18360200
H	-5.80691200	-1.89710700	-2.86850500
H	-6.93490000	1.89294400	-1.24929500
C	2.28072800	3.03676000	-0.27359100
C	1.92027900	3.78494700	-1.41403400

C	3.51437900	2.34704500	-0.22750000
C	2.80459800	3.81891700	-2.49864400
C	4.35852300	2.40940900	-1.33936400
C	4.02083900	3.13662700	-2.48398400
H	5.30282000	1.87630100	-1.31447100
H	2.53754500	4.38872000	-3.38303800
C	-0.92968700	-3.96543300	0.86575400
O	-1.03852800	-2.72375900	0.60958600
O	0.10964600	-4.66136900	0.65415300
C	-2.10882000	-4.64277300	1.52473400
H	-2.21220800	-5.66210600	1.14626000
H	-1.90866300	-4.69187900	2.60052100
H	-3.02306400	-4.07480300	1.36568200
Pd	0.47107100	-1.73458100	-0.41618200
H	1.09466700	-3.74788800	0.52811600
C	3.48401300	-2.51833600	2.31444900
C	4.15005400	-2.87648200	1.10176600
C	5.54411800	-3.02491000	1.03873900
C	6.25869600	-2.80836400	2.20531500
C	5.61923000	-2.45513900	3.42047100
C	4.24626100	-2.31083900	3.48526500
C	2.10117000	-2.44133600	2.01922400
C	1.90636700	-2.76144900	0.66892300
H	6.04029000	-3.28048200	0.10900700
H	7.34030300	-2.90464400	2.18859200
H	6.22350600	-2.29541700	4.30804200
H	3.75436700	-2.03639100	4.41365500
H	1.29711900	-2.22157100	2.71043600
C	3.44969500	-3.22936600	-1.25825500
C	4.09824700	-4.38899700	-1.68043800
C	3.02288100	-2.27367700	-2.20122300
C	4.32468300	-4.60560700	-3.03946800
H	4.39904100	-5.12029700	-0.93745100
C	3.23051600	-2.50721100	-3.56140900
C	3.88361100	-3.66976200	-3.97489900
H	4.82570900	-5.51146900	-3.36531100
H	2.89368200	-1.79102300	-4.30052000
H	4.04345200	-3.84087800	-5.03491200

N	3.20082400	-2.99442100	0.11419100
S	2.30487300	-0.77210000	-1.53233800
C	1.73990800	0.11499400	-3.01302800
H	0.95575800	-0.44184100	-3.52531400
H	1.34595100	1.06951300	-2.66521600
H	2.60748300	0.30020400	-3.64707000
C	-5.23405600	2.00163900	0.82557700
C	-6.53298600	2.15713900	1.63520300
C	-4.81954900	3.32850000	0.16702600
H	-4.44845700	1.73441300	1.53710000
H	-6.80198800	1.21762400	2.13088600
H	-6.41603400	2.93044200	2.40384300
H	-7.37216500	2.44819100	0.99296800
H	-3.88506800	3.20787900	-0.38712500
H	-5.58836200	3.68378900	-0.52917700
H	-4.67542300	4.10343600	0.92960800
C	-7.53041300	0.18653400	-3.28014500
C	-8.59681300	-0.92036400	-3.22684700
C	-6.87101700	0.25272200	-4.66829100
H	-8.03668000	1.14550800	-3.10550500
H	-9.08474500	-0.94992900	-2.24661700
H	-9.36627700	-0.75669800	-3.99077200
H	-8.15071600	-1.90535000	-3.40748800
H	-6.12926500	1.05693800	-4.71215300
H	-6.35733800	-0.68666000	-4.90356500
H	-7.62168800	0.42973300	-5.44792800
C	-3.87877400	-2.55838000	-1.13029700
C	-3.15621700	-2.78696800	-2.46775100
C	-4.75255500	-3.77203400	-0.76546200
H	-3.10163000	-2.45086300	-0.37371300
H	-2.52438200	-1.92951300	-2.70854400
H	-2.51263900	-3.67312100	-2.39402200
H	-3.86072700	-2.95638100	-3.29096300
H	-5.21741300	-3.65010100	0.22045800
H	-5.56129200	-3.90760800	-1.49317500
H	-4.15719600	-4.69281300	-0.75351600
C	4.95575200	3.18736500	-3.68155300
C	5.07732500	1.81233900	-4.36152400

C	6.34162400	3.73441100	-3.30067200
H	4.51441300	3.87908200	-4.41111100
H	4.10309400	1.47244500	-4.73029100
H	5.76396900	1.85517000	-5.21526200
H	5.45730100	1.05911700	-3.66082500
H	6.25751100	4.71798800	-2.82688200
H	6.85178600	3.06717600	-2.59634300
H	6.97782200	3.83183500	-4.18822100
C	0.63110300	4.59794600	-1.44312100
C	0.03685900	4.77796600	-2.84570900
C	0.86442400	5.97272300	-0.78645600
H	-0.11394900	4.06076300	-0.84827400
H	-0.11177400	3.81254500	-3.33144800
H	-0.94147500	5.26410000	-2.76441100
H	0.66549100	5.41842900	-3.47669400
H	1.20543100	5.87625500	0.24816100
H	1.62281500	6.53838000	-1.34147400
H	-0.06287200	6.55755600	-0.78518300
C	3.94922600	1.58460500	1.02201800
C	4.57541400	2.54078700	2.05686500
C	4.94208300	0.44690500	0.75110200
H	3.05229600	1.14093100	1.47209100
H	3.87665600	3.31670600	2.37364000
H	4.89169000	1.98422100	2.94735500
H	5.45867200	3.03123900	1.63047100
H	4.58686200	-0.22913900	-0.02901600
H	5.92287800	0.83180500	0.44752900
H	5.09310300	-0.13506300	1.66407100

int3-S

C	-7.68635600	0.49462700	0.59849300
C	-6.38221600	0.70742500	0.21105100
C	-5.42310400	-0.33958200	0.26263600
C	-5.83630500	-1.61329000	0.77349500
C	-7.19222800	-1.80293900	1.14991500
C	-8.10279100	-0.77573700	1.06049700
H	-8.40036500	1.31206400	0.55460800
H	-6.07385000	1.68750300	-0.13203200

C	-4.05651900	-0.16431800	-0.14249400
C	-4.88220100	-2.65263600	0.92838300
H	-7.49224600	-2.77757700	1.52626900
H	-9.13574900	-0.93198200	1.35757100
C	-3.55021500	-2.46040500	0.63823500
C	-3.17214900	-1.20251700	0.09499100
H	-5.21228600	-3.61386900	1.31040000
C	-3.56634000	1.09904800	-0.75904100
C	-4.15792800	1.65237800	-1.94380900
C	-2.48066900	1.75696900	-0.20337400
C	-5.16476100	0.98071700	-2.68788400
C	-3.69192200	2.91937400	-2.42231700
C	-1.98220200	3.00150400	-0.67562900
C	-5.71064500	1.55101000	-3.81601500
H	-5.49665800	0.00302300	-2.36049800
C	-4.28790800	3.48722400	-3.57951200
C	-2.62831700	3.57368100	-1.75097300
C	-5.28011200	2.82278100	-4.26205200
H	-6.47579900	1.01675900	-4.37186500
H	-3.93116800	4.45477600	-3.92337900
H	-2.30238400	4.54414100	-2.11182900
H	-5.72433000	3.26395600	-5.14957100
O	-1.83796900	-1.04467100	-0.25820800
O	-1.85311800	1.20142100	0.90214800
P	-0.86685400	-0.08213900	0.64493200
O	-0.42632400	-0.62143700	1.95273900
O	0.19659100	0.35876100	-0.38826100
C	-2.48334800	-3.48293000	0.86058200
C	-1.88912600	-3.61283600	2.13408700
C	-1.99377400	-4.22252200	-0.23426700
C	-0.79094400	-4.46656100	2.27069500
C	-0.88527700	-5.05137800	-0.05004700
C	-0.25762700	-5.17189500	1.19138700
H	-0.31173800	-4.56334800	3.23990300
H	-0.49302300	-5.60221800	-0.90066500
C	-0.82348200	3.67714500	-0.01419000
C	0.39690200	3.84616500	-0.70864300
C	-0.96147300	4.19082300	1.29550400

C	1.42602100	4.57260600	-0.09729700
C	0.10258300	4.89438700	1.86658100
C	1.29976700	5.11277900	1.18277800
H	-0.01980700	5.29896100	2.86693700
H	2.35997900	4.71557300	-0.63319600
C	0.85455300	-1.12746700	-3.11603700
O	1.67610000	-1.54982100	-2.28476700
C	0.81388000	-1.68980700	-4.50834700
H	-0.13810700	-2.20838300	-4.65747700
H	0.86240800	-0.87157000	-5.23342100
H	1.64298000	-2.38091600	-4.65836200
Pd	2.15712800	-0.60954800	-0.40300500
O	-0.03094900	-0.19117500	-2.88824000
H	-0.02307000	0.08430700	-1.90720900
C	5.71399800	-2.82269600	-0.72965300
C	6.22883000	-1.55156600	-0.35777500
C	7.59076400	-1.34489700	-0.12636100
C	8.44880700	-2.42853800	-0.30355400
C	7.96118000	-3.68833300	-0.69517100
C	6.60168100	-3.89469400	-0.90372000
C	4.28282300	-2.69380500	-0.82274600
H	7.97221200	-0.38310400	0.19804500
H	9.51257200	-2.29561200	-0.12905500
H	8.65690200	-4.51244700	-0.82518200
H	6.22580400	-4.87403300	-1.18683500
H	3.58081500	-3.46884000	-1.09101900
C	3.95425800	-1.40369400	-0.51330000
N	5.13670700	-0.68160300	-0.22221600
C	5.20968100	0.71169600	-0.04699900
C	4.25063200	1.38125600	0.73986600
C	6.19659100	1.46727100	-0.69044500
C	4.28918900	2.76813700	0.87299400
C	6.24613600	2.85126200	-0.53602100
H	6.90472000	0.95981500	-1.33448100
C	5.29308300	3.50431700	0.24413700
H	3.53442000	3.28230500	1.45011400
H	7.01948200	3.41796800	-1.04546800
H	5.31884700	4.58321000	0.36169000

S	2.99758100	0.36923700	1.53652600
C	1.77707000	1.59550000	2.10604100
H	0.95661000	0.99913100	2.50692400
H	2.23140700	2.19911900	2.89382900
H	1.42755900	2.21381600	1.28062200
C	-2.63580700	-4.13200600	-1.61272300
C	-3.08303300	-5.51393900	-2.11786900
C	-1.70283500	-3.44396800	-2.62299400
H	-3.53470900	-3.51367800	-1.52734000
H	-3.75930200	-5.99342800	-1.40192900
H	-3.60655900	-5.42142900	-3.07691800
H	-2.22837600	-6.18333500	-2.26842600
H	-1.46976300	-2.42891600	-2.29386400
H	-0.76212200	-3.99773200	-2.72872100
H	-2.17873500	-3.38994300	-3.61057700
C	1.00136500	-6.00453400	1.35904200
C	2.18247800	-5.34294500	0.62637800
C	0.80884500	-7.45932100	0.90256600
H	1.24184000	-6.01997000	2.43043900
H	2.33558000	-4.31533500	0.97098900
H	3.11172800	-5.90259700	0.78829900
H	1.99394000	-5.30586700	-0.45371300
H	-0.02325500	-7.93318000	1.43444800
H	0.59374400	-7.51410400	-0.17100700
H	1.71591200	-8.04630200	1.08874600
C	-2.44132800	-2.87281200	3.34662000
C	-1.36708600	-2.45271500	4.35980200
C	-3.51957100	-3.72657600	4.04144100
H	-2.91745600	-1.95553200	2.98543100
H	-0.57723800	-1.88599200	3.86474200
H	-1.82030300	-1.81886400	5.13114600
H	-0.92788800	-3.31738500	4.87175500
H	-4.33792800	-3.98074600	3.36123900
H	-3.08604100	-4.66528300	4.40726800
H	-3.94335500	-3.19065300	4.89928200
C	-2.24504500	4.04329700	2.10271700
C	-2.88518600	5.41585100	2.37551100
C	-2.00363600	3.26520100	3.40722900

H	-2.96253100	3.47094900	1.51110900
H	-3.08297100	5.94995300	1.43955000
H	-3.83494400	5.29549200	2.90945600
H	-2.23380700	6.04783000	2.99030200
H	-1.58873200	2.27602000	3.19667300
H	-1.31243700	3.80005700	4.06963600
H	-2.94677400	3.13192200	3.94975400
C	2.41491700	5.95462000	1.78130200
C	2.78349100	5.54011000	3.21454200
C	2.04481800	7.44827200	1.72785900
H	3.30379900	5.81270800	1.15096200
H	3.06569900	4.48323600	3.27260900
H	3.62812400	6.13452300	3.58057900
H	1.94637800	5.69741100	3.90335200
H	1.82097400	7.75951900	0.70221900
H	1.15647500	7.64682200	2.33870200
H	2.86452300	8.06948400	2.10786900
C	0.63859700	3.27177600	-2.09973100
C	1.98087600	2.52729100	-2.20175400
C	0.56900300	4.37134100	-3.17505500
H	-0.15284700	2.54771900	-2.30738700
H	2.08335300	1.78126000	-1.40932200
H	2.04779000	2.01012100	-3.16559400
H	2.83811000	3.20561500	-2.13175200
H	-0.39814700	4.88293900	-3.17006600
H	1.34601500	5.12681400	-3.00781200
H	0.72200200	3.94353800	-4.17279800

int4-S

C	-2.31158900	3.47280800	5.00115100
C	-2.17444600	2.54350300	3.99392200
C	-1.22207600	2.72665900	2.95522600
C	-0.42950700	3.91840500	2.96654500
C	-0.58830900	4.85130500	4.02423700
C	-1.50578200	4.63479900	5.02623700
H	-3.05154500	3.31451500	5.78048900
H	-2.80847900	1.66539800	3.98222700
C	-1.05028000	1.79219100	1.87841300

C	0.50037600	4.14538400	1.92031700
H	0.02746000	5.74710300	4.02064400
H	-1.62188600	5.35729800	5.82875800
C	0.63725400	3.27730300	0.86001500
C	-0.20027000	2.12643900	0.83901700
H	1.11866600	5.03697600	1.95743800
C	-1.81852600	0.52030400	1.85673100
C	-1.72444900	-0.43497100	2.92196900
C	-2.67867300	0.24539600	0.80779600
C	-0.71160800	-0.36456800	3.91567600
C	-2.63987800	-1.53546800	2.95168000
C	-3.65792800	-0.78592900	0.85786500
C	-0.60890500	-1.33541800	4.88807900
H	-0.00618600	0.45818100	3.88633200
C	-2.52158100	-2.50436000	3.98246900
C	-3.63180400	-1.64106700	1.93986100
C	-1.52585400	-2.41277300	4.92871400
H	0.18216800	-1.27383000	5.62986200
H	-3.22500200	-3.33285000	3.99830000
H	-4.38237900	-2.42241000	2.01662700
H	-1.43747800	-3.16817500	5.70388300
O	-0.07372800	1.27181600	-0.23424500
O	-2.63921200	1.04179000	-0.32141800
P	-1.32003000	0.90580700	-1.30387100
O	-1.43765300	1.89603400	-2.39722800
O	-1.00137300	-0.56781900	-1.58612800
C	1.67269900	3.47999400	-0.20090100
C	1.33707900	4.11952400	-1.41329300
C	2.98660700	3.00947200	0.02357600
C	2.32446000	4.24717300	-2.39670200
C	3.93994900	3.18265200	-0.98248500
C	3.62519400	3.77910900	-2.20462900
H	2.07570900	4.72187500	-3.34072600
H	4.94710500	2.81442500	-0.82037300
C	-4.75622600	-0.82501400	-0.15798600
C	-4.70383200	-1.69205400	-1.26894400
C	-5.85901200	0.03488900	0.02371400
C	-5.77624300	-1.70518900	-2.16252100

C	-6.90015000	-0.00293400	-0.90939900
C	-6.88258200	-0.86669200	-2.00325700
H	-7.74932400	0.66393500	-0.78458300
H	-5.74177400	-2.37755400	-3.01397100
Pd	0.75411000	-1.24544400	-0.49841800
C	-0.00229600	-3.34025500	-0.43443300
C	-0.49628400	-2.69102200	0.67959600
H	-0.55682800	-3.33869700	-1.36822700
H	-0.03542300	-2.83301400	1.64942600
H	-1.46411000	-2.21266700	0.64654800
C	1.14802300	-4.29811900	-0.41543700
O	1.67491900	-4.72004200	-1.42522400
O	1.50579800	-4.63798900	0.83151700
C	2.69561600	-5.45046700	0.95414300
H	3.50957100	-4.94570300	0.42850300
H	2.51431000	-6.41027400	0.46025900
C	2.99116200	-5.60162200	2.43197200
H	3.18602300	-4.62595000	2.88466100
H	2.15196500	-6.07524100	2.95162400
H	3.87981900	-6.22750000	2.56680000
C	3.81763000	-1.97553600	2.27172600
C	4.50738800	-2.22322900	1.05239300
C	5.87404200	-2.50844600	1.02392400
C	6.54364800	-2.59058400	2.24319200
C	5.87184300	-2.38305200	3.46148200
C	4.51705400	-2.06911700	3.48492200
C	2.46027500	-1.64138200	1.93341000
C	2.34829200	-1.68769800	0.56950500
H	6.40534700	-2.64662300	0.08941800
H	7.60624100	-2.81461800	2.24875300
H	6.42349300	-2.45691300	4.39419000
H	4.00552600	-1.88419200	4.42553200
H	1.65948500	-1.42089900	2.62568400
N	3.58627200	-2.04406700	0.00773300
C	3.86825100	-2.18231100	-1.37070900
C	3.35519600	-1.26578300	-2.31189800
C	4.64040200	-3.25634200	-1.82153800
C	3.63376100	-1.43539100	-3.66986700

C	4.92830600	-3.41021100	-3.17472700
H	4.98847900	-3.98839500	-1.10343600
C	4.42465400	-2.49947900	-4.10103100
H	3.24106000	-0.73584200	-4.39709900
H	5.52526200	-4.25512600	-3.50288100
H	4.63542400	-2.61541100	-5.15946200
S	2.38863100	0.11284500	-1.69705000
C	1.55951400	0.74425100	-3.18734000
H	0.99681700	-0.04803900	-3.68165300
H	0.87606800	1.52136300	-2.84651900
H	2.30518300	1.19269700	-3.84528100
C	-5.92298400	1.02960900	1.17686900
C	-7.25773200	0.96691200	1.93597200
C	-5.63015300	2.45612400	0.67621300
H	-5.13891600	0.76967900	1.89388600
H	-7.46493500	-0.04926000	2.28945400
H	-7.22967600	1.63408600	2.80532700
H	-8.09901400	1.28175900	1.30854100
H	-4.66293800	2.50148200	0.16784500
H	-6.39982300	2.78392000	-0.03272300
H	-5.61680500	3.16310200	1.51453900
C	-3.51425000	-2.61479300	-1.48862700
C	-3.08613800	-2.70865700	-2.95992600
C	-3.78371900	-4.00975800	-0.89934700
H	-2.67062600	-2.17267700	-0.95894800
H	-2.89392600	-1.71454700	-3.37155400
H	-2.16052000	-3.29071300	-3.04491700
H	-3.83910900	-3.20903300	-3.57948900
H	-4.00331800	-3.95387200	0.17197200
H	-4.64230200	-4.48052700	-1.39272100
H	-2.91472900	-4.66584100	-1.03413300
C	-8.02821100	-0.88285500	-3.00047500
C	-8.71994400	-2.25580400	-3.04502200
C	-7.55872400	-0.45451600	-4.40126400
H	-8.76881800	-0.14779100	-2.65774100
H	-9.07751100	-2.54915500	-2.05212800
H	-9.57676700	-2.23716800	-3.72885600
H	-8.03022800	-3.03279400	-3.39488800

H	-7.08850700	0.53392200	-4.37281600
H	-6.82330900	-1.16139800	-4.80257300
H	-8.40341000	-0.41661800	-5.09938300
C	3.38295100	2.33395100	1.33430900
C	3.82887100	3.37807900	2.37615200
C	4.48725300	1.27840000	1.17793900
H	2.49681700	1.82165200	1.72727400
H	3.03646100	4.09564700	2.60031100
H	4.11723400	2.88557300	3.31249500
H	4.69654400	3.93651400	2.00489400
H	4.25475000	0.56104400	0.38873700
H	5.45600100	1.73642300	0.94674900
H	4.60624000	0.72111100	2.11110900
C	4.67388500	3.91524300	-3.29592400
C	5.20814800	2.54392400	-3.74536800
C	5.82731400	4.83213700	-2.85393800
H	4.18749100	4.38428800	-4.16165000
H	4.39948000	1.90434200	-4.11613200
H	5.94542200	2.65643000	-4.54886900
H	5.69229700	2.01559600	-2.91637700
H	5.45261000	5.81760900	-2.55803700
H	6.36020800	4.40607300	-1.99596200
H	6.55167100	4.96733400	-3.66584000
C	-0.04623300	4.72477000	-1.62026700
C	-0.49492900	4.77732800	-3.08581200
C	-0.09497700	6.13572500	-1.00165000
H	-0.76878700	4.09700100	-1.09096100
H	-0.44537000	3.79030100	-3.54884600
H	-1.53723300	5.10961400	-3.13401200
H	0.10194500	5.48775900	-3.67091200
H	0.13409900	6.11704200	0.06767400
H	0.63180800	6.79475700	-1.49202500
H	-1.09193900	6.57408300	-1.12757700

TS5-S

C	-7.37149200	0.30637700	-1.74060900
C	-6.23165700	-0.13319600	-1.10581800
C	-5.20142000	0.77557700	-0.74224200

C	-5.36078400	2.15534600	-1.08793800
C	-6.55500800	2.57896800	-1.72867100
C	-7.54416100	1.67733400	-2.04594000
H	-8.14323300	-0.40702400	-2.01524900
H	-6.10512500	-1.18615200	-0.88449200
C	-4.00177200	0.36109100	-0.07483800
C	-4.31662100	3.06860200	-0.79656600
H	-6.66481900	3.63202800	-1.97549600
H	-8.45111300	2.01115700	-2.54174500
C	-3.12011800	2.65826500	-0.24775100
C	-2.98112800	1.28461700	0.09137600
H	-4.45895500	4.11917100	-1.03231600
C	-3.82192100	-1.02021800	0.44174900
C	-4.74727600	-1.59465300	1.37555100
C	-2.70393000	-1.75215500	0.06985600
C	-5.83451700	-0.87095400	1.93733000
C	-4.55343600	-2.95031300	1.79033400
C	-2.46528700	-3.08066600	0.51621200
C	-6.70553700	-1.47112900	2.81872300
H	-5.97046000	0.16959800	1.66978000
C	-5.47888300	-3.54548600	2.68813100
C	-3.41986400	-3.66090800	1.32612800
C	-6.53834500	-2.82636200	3.18985000
H	-7.52657000	-0.89623800	3.23774600
H	-5.32175000	-4.58022300	2.98229100
H	-3.28643600	-4.69150200	1.64032500
H	-7.23735500	-3.28905000	3.88060900
O	-1.78936300	0.87293300	0.66433400
O	-1.80736500	-1.18789400	-0.82146600
P	-0.76927200	-0.03244900	-0.28615800
O	-0.23122000	0.74998500	-1.43136800
O	0.18001200	-0.65828900	0.73607700
C	-2.03204300	3.64591700	0.02818800
C	-1.23414000	4.13781400	-1.02365500
C	-1.85297300	4.12873500	1.34454600
C	-0.28886900	5.13237700	-0.73809700
C	-0.89840000	5.12084400	1.57776300
C	-0.10505900	5.63824400	0.54811200

H	0.31976000	5.53001300	-1.54616300
H	-0.76881400	5.50152900	2.58623600
C	-1.24654600	-3.86149900	0.13671200
C	-0.22860600	-4.07758400	1.09287400
C	-1.16697700	-4.46156700	-1.13660700
C	0.82512600	-4.93783700	0.76757500
C	-0.09054900	-5.30889500	-1.41509700
C	0.90452100	-5.57431200	-0.47447100
H	-0.02546300	-5.78238800	-2.38984300
H	1.59984200	-5.12017800	1.50661000
Pd	2.26280800	-0.69344600	0.38449400
C	5.07941300	2.03408000	-1.74324500
C	4.46430300	1.51797900	-0.60325300
C	3.32783800	2.15770800	-0.06844800
C	2.81059000	3.28714600	-0.70501000
C	3.44109400	3.79934600	-1.83962800
C	4.57964100	3.18299900	-2.35608900
H	5.94404300	1.51580300	-2.14510500
H	1.91758200	3.77158200	-0.33251600
H	3.02565900	4.67852000	-2.32191400
H	5.06396000	3.57648400	-3.24405000
C	6.22853300	0.38620000	0.71550300
C	4.30069700	-0.78969700	0.40027300
C	7.23795800	1.35217200	0.72751900
C	6.29969000	-0.79289500	1.50841000
C	5.08450700	-1.51038500	1.28695600
C	8.35159400	1.10117300	1.52199200
H	7.15467400	2.26039200	0.14055300
C	7.44441200	-1.02324900	2.29370100
H	4.82640800	-2.48205900	1.68604000
C	8.45845500	-0.07674500	2.29152400
H	9.15713800	1.82905200	1.54979100
H	7.52160900	-1.92143000	2.89967700
H	9.34613400	-0.23678300	2.89616400
S	2.61891600	1.48844400	1.44031200
C	0.98826700	2.29094500	1.50786900
H	0.38199000	1.67676800	2.17303800
H	1.08514800	3.30187000	1.90018000

H	0.52642100	2.29815000	0.52230800
C	3.69937800	-2.10194300	-1.07344400
C	2.28394000	-2.21590400	-1.12296500
H	4.25601300	-2.92451300	-0.64674100
H	4.20070100	-1.54328700	-1.85615900
H	1.80710200	-3.08389300	-0.67894100
C	1.53851400	-1.65485700	-2.28662500
O	0.48542800	-2.11238800	-2.67719500
O	2.19031300	-0.63740300	-2.87918500
C	1.48342200	-0.01088800	-3.97838000
H	0.53671400	0.36164400	-3.58459400
H	1.27814300	-0.77309400	-4.73668400
C	2.37183800	1.09597000	-4.50556900
H	3.33057800	0.69999600	-4.85872200
H	2.56809500	1.83603700	-3.72651300
H	1.87654900	1.59922200	-5.34310800
N	5.01405800	0.36908500	0.02850500
C	-2.27776900	-4.26534000	-2.16064100
C	-3.38326400	-5.31654300	-1.94435600
C	-1.79071100	-4.28333200	-3.61579800
H	-2.71514200	-3.27818900	-1.98786800
H	-3.79603400	-5.26096000	-0.93226400
H	-4.20368900	-5.16575500	-2.65635000
H	-2.98524800	-6.32771400	-2.09355300
H	-0.97616100	-3.57052300	-3.75829600
H	-1.44847800	-5.27993700	-3.92087600
H	-2.61754400	-4.01071400	-4.28191600
C	-0.26668000	-3.43109100	2.47403800
C	1.10601600	-2.91583600	2.93514200
C	-0.82531800	-4.40671200	3.52698400
H	-0.93088000	-2.56490700	2.41632500
H	0.99414600	-2.32898900	3.85404700
H	1.80055300	-3.73590900	3.15468800
H	1.56229500	-2.27072500	2.17853800
H	-1.84263300	-4.72531700	3.28593800
H	-0.19806800	-5.30432000	3.59061700
H	-0.84435300	-3.93526600	4.51694500
C	2.04307300	-6.52808100	-0.79746000

C	2.09999000	-7.70097700	0.19528300
C	3.39556900	-5.79806400	-0.86326800
H	1.84583300	-6.94436300	-1.79418300
H	1.14329800	-8.23269500	0.22791000
H	2.88233000	-8.41458300	-0.08931500
H	2.32094000	-7.35011900	1.21002900
H	3.37614000	-5.01298600	-1.62624700
H	3.63364300	-5.33102600	0.10088900
H	4.20670300	-6.49416000	-1.10756900
C	-1.39729500	3.64164500	-2.45431900
C	-0.05286500	3.37675900	-3.14626000
C	-2.24337500	4.62905600	-3.27819400
H	-1.92289400	2.68460400	-2.41359200
H	0.54897500	2.68712300	-2.55370700
H	-0.22833200	2.91938700	-4.12665900
H	0.51413500	4.30014700	-3.31742400
H	-3.23647700	4.77205200	-2.84169400
H	-1.75556000	5.61035800	-3.32959600
H	-2.37314200	4.26116600	-4.30285000
C	0.92961100	6.71814500	0.81973300
C	2.02754700	6.22991900	1.78102200
C	0.27271300	8.00569700	1.34519200
H	1.41021000	6.95641500	-0.13895700
H	2.54836500	5.35398100	1.37936400
H	2.77249600	7.01587200	1.95112800
H	1.60520600	5.95546500	2.75480100
H	-0.49293800	8.36718700	0.65073300
H	-0.20985200	7.83360600	2.31422200
H	1.01961600	8.79694700	1.47902600
C	-2.73125100	3.62786700	2.48469400
C	-3.96396600	4.53766600	2.63834400
C	-1.98563800	3.48649000	3.81898700
H	-3.09168200	2.63111200	2.21711700
H	-4.54160300	4.57372200	1.70922000
H	-4.62084000	4.17050600	3.43581400
H	-3.66004600	5.56110300	2.88917000
H	-1.09043100	2.86554600	3.71064800
H	-1.67973800	4.45658400	4.22740400

H	-2.63766200	3.01326800	4.56131800

int6-S

C	7.65045400	-1.22918900	0.84582200
C	6.36072000	-1.39485800	0.39307700
C	5.50707900	-0.27735600	0.18865400
C	6.00807900	1.02603300	0.51131800
C	7.34816200	1.16320300	0.96038700
C	8.15764300	0.06259100	1.12052000
H	8.28244200	-2.09930600	0.99906400
H	5.97992600	-2.38926500	0.19404400
C	4.16071700	-0.40789400	-0.29157100
C	5.15178600	2.15269800	0.40700800
H	7.71681000	2.15976400	1.19029700
H	9.17899500	0.18007400	1.47103600
C	3.82866800	2.02232000	0.04828200
C	3.36163800	0.72573700	-0.30021800
H	5.54961900	3.13621900	0.63852900
C	3.59204200	-1.70783000	-0.74424500
C	4.19787300	-2.49847100	-1.77800100
C	2.39644200	-2.14979800	-0.20242000
C	5.34110600	-2.07511100	-2.50780700
C	3.60538500	-3.75827100	-2.11834500
C	1.74773200	-3.35725100	-0.58036500
C	5.89854400	-2.87472100	-3.48067600
H	5.77233800	-1.10474300	-2.29430900
C	4.21501100	-4.56513500	-3.11521800
C	2.39678500	-4.16003200	-1.49264700
C	5.34153500	-4.13986300	-3.78044100
H	6.77096200	-2.52748800	-4.02700200
H	3.75960800	-5.52298300	-3.35387000
H	1.95839700	-5.11307500	-1.77110600
H	5.79460900	-4.76381900	-4.54546500
O	2.04295400	0.62621600	-0.70847300
O	1.79639700	-1.38930500	0.78729900
P	0.94942900	-0.06614500	0.31778900
O	0.64736200	0.74363100	1.53452000
O	-0.18145900	-0.46166700	-0.63284400

C	2.88035900	3.17766800	-0.00865500
C	2.29146800	3.67323500	1.17331700
C	2.53420200	3.73207600	-1.25927800
C	1.37431300	4.72743100	1.07916100
C	1.60210700	4.77120200	-1.30270000
C	1.00793900	5.28432800	-0.14623500
H	0.92608400	5.10537400	1.99016600
H	1.32555500	5.19401100	-2.26653800
C	0.40421500	-3.69146600	-0.01373600
C	-0.75207800	-3.57529300	-0.82090800
C	0.28333000	-4.06982800	1.33850400
C	-1.99573600	-3.85647500	-0.24899500
C	-0.98199900	-4.35556100	1.85800500
C	-2.13545700	-4.24676200	1.08392100
H	-1.06309400	-4.65042700	2.89958600
H	-2.88963900	-3.75425900	-0.85706600
Pd	-1.99782100	-0.03758600	0.46708000
C	-6.02201700	2.79027700	0.15300300
C	-4.98215100	2.04213100	-0.39352500
C	-3.70269100	2.61553600	-0.52980200
C	-3.48379200	3.93101400	-0.12110000
C	-4.53422500	4.67075200	0.42751900
C	-5.80066900	4.10516300	0.56893600
H	-6.99923800	2.32727100	0.24470300
H	-2.50354900	4.38084500	-0.21268500
H	-4.35062300	5.69156300	0.74876800
H	-6.61408000	4.68216200	0.99712900
C	-5.38172200	0.31392100	-2.13730000
C	-5.52373000	1.10022500	-3.28066400
C	-5.39298300	-1.10464100	-2.17490600
C	-5.68086800	0.43645100	-4.49401200
H	-5.50829400	2.18432100	-3.22337900
C	-5.56006100	-1.74535600	-3.41297800
C	-5.69938600	-0.97086400	-4.55879800
H	-5.79006400	1.01462400	-5.40686600
H	-5.57194600	-2.83008000	-3.47224600
H	-5.82240400	-1.45611800	-5.52261900
S	-2.42550200	1.55540600	-1.19449600

C	-0.93518200	2.60394900	-1.12869900
H	-0.13862500	1.97913700	-1.52790300
H	-1.07365300	3.47583900	-1.76948400
H	-0.68304300	2.88981700	-0.10779000
C	-5.06369200	-0.45636600	-0.01993500
C	-4.76608800	-0.40285200	1.44813900
H	-5.62194300	0.00696400	2.00652300
H	-4.62971400	-1.43549000	1.77969200
C	-3.51803000	0.41170100	1.79795500
H	-3.68383000	1.48008200	1.92222200
C	-2.55223400	-0.15979600	2.75018200
O	-1.97031000	-1.21365300	2.38530100
O	-2.19966100	0.53835800	3.81227600
C	-0.93142700	0.15806400	4.44965700
H	-0.20172500	-0.00227200	3.65357600
H	-0.66152600	1.05296100	5.01217500
N	-5.19743900	0.69887000	-0.80878500
C	-5.19294700	-1.55674200	-0.82591400
H	-5.13120600	-2.58136400	-0.48748500
C	-1.10115500	-1.05010200	5.35197100
H	-1.35256000	-1.93821600	4.76802100
H	-0.15812600	-1.24253600	5.87566000
H	-1.88153500	-0.87562900	6.10043000
C	-3.51162200	-4.58258800	1.63336600
C	-3.72455500	-4.11644300	3.08053100
C	-3.78809900	-6.09129100	1.49861400
H	-4.24423400	-4.05646800	1.00508200
H	-3.46502000	-3.06018800	3.19313100
H	-4.76930800	-4.26142100	3.37993900
H	-3.10432200	-4.68838100	3.78031700
H	-3.68973800	-6.41687200	0.45756800
H	-3.07004900	-6.66487200	2.09678300
H	-4.79818600	-6.34101300	1.84612700
C	1.49270300	-4.18593800	2.25618700
C	1.71695800	-5.64354600	2.69389800
C	1.37189800	-3.24756800	3.46870700
H	2.37940500	-3.87962100	1.69502500
H	1.84532800	-6.29841200	1.82487900

H	2.61242700	-5.72595700	3.32162200
H	0.86539700	-6.02016500	3.27277300
H	1.20711900	-2.21846400	3.13957900
H	0.53781600	-3.54050600	4.11676300
H	2.28844700	-3.28008900	4.07028100
C	-0.68517300	-3.16626800	-2.28801700
C	-1.82895400	-2.23166900	-2.71304900
C	-0.67683000	-4.40988800	-3.19791100
H	0.25129400	-2.62291200	-2.43910900
H	-1.92606600	-1.39045400	-2.02615400
H	-1.63288600	-1.83537700	-3.71604800
H	-2.79318400	-2.74899100	-2.75654700
H	0.16232300	-5.07505900	-2.97559800
H	-1.60128100	-4.98551600	-3.06640900
H	-0.60770700	-4.11621700	-4.25234900
C	0.01733800	6.43438100	-0.26550900
C	0.76018800	7.75150700	-0.56063100
C	-0.89730300	6.60395100	0.95454800
H	-0.62269400	6.21912600	-1.13509600
H	1.37684100	7.66498900	-1.46087200
H	0.05410900	8.57822200	-0.70418600
H	1.42265000	8.00746400	0.27453700
H	-1.39058300	5.66595500	1.23219900
H	-0.33499300	6.95041800	1.82904600
H	-1.67024400	7.35261600	0.74683300
C	3.15220900	3.23184800	-2.55924300
C	3.89401100	4.36193600	-3.29325000
C	2.10717800	2.56558600	-3.47034900
H	3.89522300	2.46997200	-2.30763200
H	4.65239100	4.81815700	-2.64763000
H	4.39312400	3.97389000	-4.18879200
H	3.20635300	5.15390500	-3.61226500
H	1.65508900	1.70335100	-2.97269700
H	1.31115100	3.26898600	-3.74402900
H	2.57553000	2.21620000	-4.39795900
C	2.64904400	3.10920200	2.54298100
C	1.44841500	3.02988600	3.49840300
C	3.78482800	3.92932600	3.18281900

H	3.00525400	2.08629100	2.39389200
H	0.62325700	2.49763600	3.02319300
H	1.73815300	2.48255600	4.40339900
H	1.10951800	4.02306500	3.81787100
H	4.68185100	3.93679600	2.55685200
H	3.47165000	4.97010600	3.33158200
H	4.05655700	3.51371800	4.16051300

TS7-S

C	7.36703300	-0.86786000	2.15614400
C	6.24252400	-1.11122200	1.40004900
C	5.38805700	-0.04946500	0.99755900
C	5.70260300	1.27728400	1.43595400
C	6.87863500	1.49618100	2.20117600
C	7.69896300	0.44938200	2.55174000
H	8.00189500	-1.69614300	2.45730700
H	5.99292000	-2.12508900	1.11164800
C	4.20936100	-0.26335900	0.20651900
C	4.82737800	2.34507700	1.11384300
H	7.10890700	2.51053100	2.51676800
H	8.59227300	0.62786300	3.14315000
C	3.64378800	2.13435700	0.44042800
C	3.35347000	0.81219700	0.01465500
H	5.09268800	3.35291500	1.41888000
C	3.87712700	-1.59112400	-0.37864000
C	4.76855300	-2.30716600	-1.24633300
C	2.62702300	-2.13143700	-0.14341700
C	6.02218900	-1.79044600	-1.66952500
C	4.36075700	-3.58988900	-1.73990900
C	2.15840600	-3.35053800	-0.70289100
C	6.85033600	-2.52354300	-2.49010000
H	6.32318100	-0.80304400	-1.34170100
C	5.24596600	-4.32546400	-2.57182600
C	3.06170300	-4.07999800	-1.44545600
C	6.46842300	-3.81044200	-2.93589900
H	7.80348900	-2.10664900	-2.80253300
H	4.92728100	-5.30096700	-2.93016600
H	2.75922200	-5.03510700	-1.86329600

H	7.13381700	-4.38063900	-3.57760700
O	2.15808800	0.64030100	-0.68033300
O	1.76477800	-1.43273500	0.69289900
P	0.94239800	-0.20487200	0.00698200
O	0.20931800	0.51817700	1.12657200
O	0.02022400	-0.67016100	-1.10554900
C	2.72647900	3.26677700	0.10179000
C	1.86045600	3.80493700	1.07913100
C	2.76896900	3.82078600	-1.19334100
C	1.10000100	4.93332500	0.75280900
C	1.97631800	4.93833700	-1.47503600
C	1.15421300	5.52551900	-0.51331800
H	0.46226500	5.37117100	1.51531600
H	2.01586600	5.37903100	-2.46821800
C	0.72164300	-3.73861500	-0.55528900
C	-0.13731900	-3.65784300	-1.67914600
C	0.20172000	-4.10595900	0.70000300
C	-1.48309900	-3.98090900	-1.51228400
C	-1.15869100	-4.42261500	0.81486400
C	-2.01804500	-4.37398000	-0.28139300
H	-1.54342800	-4.71204300	1.78639000
H	-2.14792800	-3.91656400	-2.36920500
Pd	-1.68822900	-0.50320800	0.37453600
C	-4.94603700	2.81370400	1.28170000
C	-4.32129800	2.20423000	0.19431600
C	-3.03615700	2.60082100	-0.22269700
C	-2.36742400	3.57471800	0.53141600
C	-2.98473900	4.15765300	1.63685800
C	-4.28069700	3.79849200	2.01011900
H	-5.94066600	2.48027900	1.56147700
H	-1.36008300	3.87089700	0.27294300
H	-2.43923700	4.89926400	2.21244000
H	-4.75865700	4.26159300	2.86707400
C	-5.94689800	1.30898400	-1.45883300
C	-6.39436500	2.49075500	-2.05661000
C	-6.46868800	0.03147300	-1.80596000
C	-7.37995800	2.37491100	-3.02771300
H	-5.97951600	3.45213400	-1.77262400

C	-7.46832200	-0.05080100	-2.79420200
C	-7.91179700	1.11751900	-3.39374000
H	-7.74907000	3.27012600	-3.51944100
H	-7.88083300	-1.01446700	-3.07902000
H	-8.68065100	1.07111800	-4.15923300
S	-2.35333900	1.79590600	-1.64314100
C	-0.65945100	2.46187900	-1.69106000
H	-0.13570900	1.86269400	-2.43606700
H	-0.64630700	3.51270100	-1.98505500
H	-0.16080200	2.33577900	-0.73005300
C	-4.89526100	-0.23550300	-0.18682900
N	-4.98351200	1.13212100	-0.47885900
C	-5.78771000	-0.92037700	-0.99046800
H	-5.91310800	-1.99525800	-0.99709300
C	-3.48797700	-4.76987500	-0.21964300
C	-4.05695100	-4.88684100	1.19810900
C	-3.71416700	-6.08576500	-0.98913600
H	-4.05347400	-3.98360800	-0.74464100
H	-3.90039400	-3.97266700	1.77580800
H	-5.13246000	-5.09333900	1.15713000
H	-3.58769000	-5.71483200	1.74337000
H	-3.36460600	-6.01067300	-2.02341500
H	-3.16311300	-6.90312100	-0.50903200
H	-4.77776800	-6.35257900	-1.00376700
C	1.08565100	-4.20213400	1.93547600
C	1.21659600	-5.66276200	2.40183900
C	0.58438600	-3.29787900	3.07241800
H	2.08830000	-3.86189100	1.66432400
H	1.61766500	-6.29593800	1.60273000
H	1.88698600	-5.73416700	3.26664600
H	0.24328800	-6.07320200	2.69616900
H	0.45876200	-2.26761900	2.72952000
H	-0.37520700	-3.65265600	3.46032100
H	1.29830300	-3.30054800	3.90458000
C	0.35095400	-3.23879500	-3.06128200
C	-0.59035000	-2.24199000	-3.75840700
C	0.56715400	-4.47563800	-3.95351200
H	1.31361000	-2.73577100	-2.93954300

H	-0.78213800	-1.38369500	-3.11261100
H	-0.12760000	-1.88841300	-4.68759400
H	-1.54717300	-2.70281500	-4.03104000
H	1.27462800	-5.18201100	-3.50768000
H	-0.37894100	-5.00878000	-4.10735300
H	0.95329500	-4.18086700	-4.93658100
C	0.39770600	6.80638900	-0.82882500
C	0.97424100	7.98503400	-0.02392000
C	-1.11693900	6.68434900	-0.60533900
H	0.55847700	7.02152100	-1.89373500
H	2.04779400	8.09832200	-0.20787200
H	0.47567200	8.92334300	-0.29447300
H	0.83482400	7.82691700	1.05198400
H	-1.54764800	5.87163800	-1.19887700
H	-1.35083200	6.48673600	0.44659500
H	-1.62225500	7.61567400	-0.88607600
C	3.65855600	3.24677300	-2.28863000
C	4.63145600	4.30080800	-2.84286300
C	2.81732900	2.61300100	-3.41011500
H	4.26645100	2.45064100	-1.85047000
H	5.23873700	4.73492900	-2.04101000
H	5.30654300	3.84729000	-3.57781300
H	4.10016000	5.11955600	-3.34134900
H	2.19308600	1.80765000	-3.01329200
H	2.16437300	3.35586600	-3.88369600
H	3.46707200	2.19258600	-4.18675800
C	1.77893800	3.21307800	2.48149500
C	0.33980900	3.10363400	3.00678400
C	2.65094800	4.02192700	3.45926200
H	2.17710300	2.19526600	2.43470000
H	-0.28451600	2.53471400	2.31678600
H	0.33555700	2.57964700	3.96878400
H	-0.11169000	4.08978300	3.17123900
H	3.69795500	4.04599800	3.14116200
H	2.29751100	5.05821600	3.52606400
H	2.61004400	3.58402800	4.46340700
C	-3.98061900	-0.85487800	0.76191400
C	-3.21266900	-0.19777700	1.77681300

H	-4.22297700	-1.89566800	0.95802300
H	-3.27190500	0.87740200	1.88860700
C	-2.87443100	-0.86428200	3.06273700
O	-2.48705000	-0.25315900	4.03742200
O	-3.07430500	-2.20261200	3.03899000
C	-2.87285600	-2.90067700	4.28930000
H	-2.66161600	-3.92949300	3.99172900
H	-1.99884500	-2.47814900	4.78763800
C	-4.11321100	-2.81340800	5.16313700
H	-3.96934100	-3.40465900	6.07441600
H	-4.30428100	-1.77655700	5.45297000
H	-4.98893600	-3.20331200	4.63374400
H	-2.82459800	-1.39818700	-0.25358600

int8-S

C	7.51980100	-0.51286200	2.07463300
C	6.38618700	-0.82554500	1.35862900
C	5.48098600	0.18796300	0.94309100
C	5.75399300	1.54026400	1.32704900
C	6.94027600	1.83103400	2.05148800
C	7.81010100	0.82949700	2.41516100
H	8.19445700	-1.30498900	2.38662100
H	6.16838800	-1.85755800	1.11118500
C	4.29268000	-0.09499600	0.19137200
C	4.82575000	2.55999400	0.99785000
H	7.13854700	2.86398200	2.32620000
H	8.71093200	1.06298500	2.97531400
C	3.63235600	2.28132600	0.36717400
C	3.38906600	0.93650000	-0.02194300
H	5.05543900	3.58524300	1.27196700
C	3.98931300	-1.44743000	-0.34858000
C	4.88877200	-2.14619100	-1.22268100
C	2.75860100	-2.02183300	-0.07757500
C	6.11403500	-1.59059200	-1.68074700
C	4.52328700	-3.45150600	-1.68815300
C	2.33365000	-3.26506900	-0.61895500
C	6.95477600	-2.30651600	-2.50350900
H	6.38309500	-0.58630000	-1.37815500

C	5.42029600	-4.16894100	-2.52335400
C	3.24694100	-3.97796500	-1.36541700
C	6.61557400	-3.61544100	-2.91916800
H	7.88494600	-1.85864600	-2.84155100
H	5.13173000	-5.16207200	-2.85838200
H	2.96428800	-4.94609600	-1.76828000
H	7.29092400	-4.17166000	-3.56285300
O	2.19389800	0.66802100	-0.67597400
O	1.89303200	-1.36730600	0.78158600
P	1.03015800	-0.08950600	0.22054700
O	0.54155900	0.71446300	1.37247400
O	0.03580800	-0.57179100	-0.84769200
C	2.64383800	3.35996400	0.05796100
C	1.86095300	3.93059700	1.08819800
C	2.52576000	3.83239900	-1.26518500
C	1.00375400	4.98968500	0.77055700
C	1.64429700	4.88600800	-1.53237200
C	0.88406100	5.48797300	-0.53026300
H	0.42509400	5.44864800	1.56714200
H	1.55876400	5.25982600	-2.55000600
C	0.91549200	-3.70602300	-0.46786100
C	0.06662700	-3.68784900	-1.59646300
C	0.40539100	-4.08642700	0.79061300
C	-1.26447100	-4.09392800	-1.44935800
C	-0.93545700	-4.46428500	0.88943100
C	-1.78983600	-4.48240700	-0.21864000
H	-1.32789600	-4.75684200	1.85867500
H	-1.90661200	-4.07983700	-2.32218900
Pd	-1.87247100	-0.68631400	0.07368100
C	-5.10744200	2.69193500	1.56292000
C	-4.43358100	2.09334500	0.49979000
C	-3.09942100	2.43376900	0.21685800
C	-2.43380800	3.34240600	1.04294300
C	-3.11115800	3.92799100	2.11312500
C	-4.44692400	3.61749200	2.37143900
H	-6.13815300	2.40991300	1.75283200
H	-1.39578700	3.59126600	0.86697000
H	-2.57960400	4.62554100	2.75298500

H	-4.96681900	4.07742100	3.20555300
C	-5.99851200	1.45166200	-1.31362800
C	-6.47831400	2.70541300	-1.70145400
C	-6.40718100	0.24708000	-1.95170400
C	-7.39117500	2.73808700	-2.74827500
H	-6.14669700	3.61084600	-1.20429400
C	-7.33612500	0.31510000	-3.00761900
C	-7.81761100	1.55603300	-3.39429200
H	-7.78525600	3.69457900	-3.07877000
H	-7.66265700	-0.59090800	-3.50981000
H	-8.53271700	1.62640900	-4.20834500
S	-2.34438500	1.58364500	-1.15189100
C	-0.72057000	2.38856900	-1.28864600
H	-0.18415400	1.82632500	-2.05134600
H	-0.84335700	3.42448500	-1.60273500
H	-0.17002300	2.32713300	-0.35164000
C	-4.91203600	-0.26741400	-0.31263700
N	-5.09269000	1.12575000	-0.31506800
C	-5.71075200	-0.81427000	-1.30406400
H	-5.77991700	-1.87064000	-1.52312700
C	-3.23510000	-4.91982500	-0.03864100
C	-3.31379300	-6.44004200	0.19691900
C	-4.15461500	-4.51116600	-1.19593100
H	-3.60239000	-4.42393400	0.86931300
H	-2.70073800	-6.74163000	1.05269800
H	-4.34703900	-6.75525300	0.38653600
H	-2.94673100	-6.98238700	-0.68241000
H	-4.05857300	-3.44507100	-1.42845600
H	-3.92226700	-5.07001200	-2.10993100
H	-5.20027700	-4.71987500	-0.94206700
C	1.27580200	-4.11778900	2.03812900
C	1.47341400	-5.56354600	2.52706800
C	0.71207500	-3.22306000	3.15367900
H	2.26238400	-3.72936200	1.77433400
H	1.91697100	-6.18732300	1.74305200
H	2.13459100	-5.58816000	3.40135800
H	0.51814700	-6.01851800	2.81549300
H	0.54710400	-2.20312300	2.79806900

H	-0.23799400	-3.61540000	3.53194300
H	1.40989500	-3.18351500	3.99809200
C	0.53545300	-3.23103200	-2.97371800
C	-0.40924300	-2.19532600	-3.60785800
C	0.72514400	-4.43699300	-3.91133400
H	1.50524200	-2.74214000	-2.85595300
H	-0.53421500	-1.34066900	-2.94019400
H	0.01243100	-1.84201000	-4.55657100
H	-1.39636500	-2.61857500	-3.82814400
H	1.43956000	-5.15802000	-3.50030800
H	-0.22468300	-4.96322500	-4.06592700
H	1.09431700	-4.11128200	-4.89104400
C	-0.00943200	6.67705200	-0.84507500
C	0.52763900	7.95536600	-0.17737000
C	-1.47689800	6.43577000	-0.45719200
H	0.02544600	6.82984500	-1.93216400
H	1.56227800	8.14994400	-0.47860200
H	-0.08188900	8.82480200	-0.45094100
H	0.51022500	7.86146900	0.91477800
H	-1.88398300	5.54752500	-0.95160400
H	-1.58494200	6.28989800	0.62331300
H	-2.09622900	7.29525900	-0.73894700
C	3.35198900	3.25424800	-2.40755000
C	4.23723400	4.33172400	-3.05715400
C	2.46538500	2.55299900	-3.45044100
H	4.02291500	2.49753500	-1.99292100
H	4.88132900	4.81177300	-2.31227200
H	4.87660700	3.88650900	-3.82830600
H	3.63612900	5.11443900	-3.53411200
H	1.92175200	1.72207900	-2.99313400
H	1.73968300	3.24818300	-3.88957100
H	3.07884000	2.15110800	-4.26543800
C	1.96319500	3.46021500	2.53480300
C	0.59355300	3.27181600	3.20532800
C	2.83408700	4.43202000	3.35251300
H	2.44835800	2.48121300	2.53250300
H	-0.00543000	2.54835500	2.65080700
H	0.73146800	2.88461800	4.22124000

H	0.04448200	4.21823600	3.29205300
H	3.83650100	4.53571600	2.92524600
H	2.38025000	5.43038700	3.38105700
H	2.93916800	4.07822300	4.38480500
H	-1.50995900	-2.03404800	0.70353200
C	-4.06052900	-1.01712900	0.57992100
C	-3.32188400	-0.55596000	1.67842100
H	-4.20164000	-2.08953200	0.50290600
H	-3.29922100	0.49200700	1.94894500
C	-2.99273400	-1.42447900	2.84515200
O	-2.53482100	-0.98474200	3.87830200
O	-3.33105700	-2.72092900	2.65723300
C	-3.16830000	-3.59257100	3.79916300
H	-3.05945800	-4.58902800	3.36578200
H	-2.25001000	-3.31862600	4.32077900
C	-4.37516200	-3.51069600	4.71923600
H	-4.26835200	-4.22993800	5.53899700
H	-4.45906600	-2.50910500	5.14968800
H	-5.29578600	-3.74283900	4.17370700

TS9-S

C	6.68116500	-2.93034900	2.09638800
C	5.54993900	-2.79606800	1.32315400
C	5.07196300	-1.51293300	0.94271400
C	5.77141300	-0.35885700	1.42394800
C	6.94345800	-0.53294000	2.20663400
C	7.39549900	-1.79008200	2.53369300
H	7.02501800	-3.92138900	2.37835300
H	5.00624900	-3.67620500	1.00216700
C	3.90265600	-1.32970500	0.13130500
C	5.27036400	0.93551000	1.13330900
H	7.46901600	0.35268500	2.55465000
H	8.28978200	-1.91056400	3.13819100
C	4.09594100	1.12059100	0.43627600
C	3.43563400	-0.03670000	-0.05411100
H	5.82337800	1.80230600	1.48254400
C	3.15842500	-2.47604500	-0.46220600
C	3.76922900	-3.43933200	-1.33116700

C	1.80211300	-2.59285300	-0.21284800
C	5.11840200	-3.34405700	-1.76563500
C	2.97421500	-4.53022700	-1.81386500
C	0.97534300	-3.62222800	-0.73402100
C	5.66549400	-4.30020400	-2.59179000
H	5.71704200	-2.50117300	-1.44195800
C	3.57468600	-5.50654000	-2.65248200
C	1.59512400	-4.59738900	-1.48668100
C	4.89291300	-5.40102200	-3.03099900
H	6.69850900	-4.20523600	-2.91399000
H	2.96264500	-6.33373500	-3.00291800
H	1.00804400	-5.42518300	-1.87212500
H	5.33860600	-6.15116000	-3.67779400
O	2.27756800	0.17135100	-0.79580000
O	1.20680900	-1.64318300	0.60349700
P	0.84364500	-0.21849100	-0.11202400
O	0.54684500	0.72905600	1.05503500
O	-0.17740800	-0.35259500	-1.20479400
C	3.56153800	2.48924600	0.14884500
C	2.87099600	3.21702600	1.14499800
C	3.77956500	3.06037400	-1.12073300
C	2.46312200	4.52364000	0.86007200
C	3.33770300	4.36630900	-1.36125100
C	2.69547600	5.12225400	-0.38173300
H	1.95495800	5.09186400	1.63312900
H	3.51187400	4.81312800	-2.33714500
C	-0.50295900	-3.62096900	-0.49938800
C	-1.38383300	-3.36958800	-1.57865500
C	-1.02481700	-3.87657500	0.78501300
C	-2.75801300	-3.47099800	-1.35509100
C	-2.41315800	-3.94591700	0.96052600
C	-3.29955000	-3.77652600	-0.10324900
H	-2.80111500	-4.15974200	1.95050100
H	-3.43634600	-3.31693000	-2.19061100
Pd	-1.55275500	0.71443900	1.36430500
C	-4.51368200	3.73574600	0.77820400
C	-3.78743100	3.02269200	-0.17180300
C	-2.42496800	3.30743900	-0.41010700

C	-1.79582200	4.26411300	0.40156200
C	-2.52032400	4.94502200	1.37948200
C	-3.88405000	4.70540700	1.56014100
H	-5.56300000	3.49379300	0.91792000
H	-0.74086000	4.47157300	0.27742200
H	-2.01092000	5.67797800	1.99857500
H	-4.44662100	5.25163500	2.31007800
C	-4.83871200	2.02880200	-2.19230700
C	-5.08960400	3.16640400	-2.96431500
C	-5.00244800	0.71025900	-2.70455600
C	-5.52761700	2.96435000	-4.26610400
H	-4.93927900	4.16110000	-2.55812200
C	-5.45466600	0.53969300	-4.02764900
C	-5.71149500	1.66500300	-4.79323300
H	-5.73215800	3.82472000	-4.89652100
H	-5.58795000	-0.45741600	-4.43699400
H	-6.05509100	1.55362500	-5.81721500
S	-1.63464500	2.41679000	-1.71299800
C	0.11000900	2.88897000	-1.53079700
H	0.64892800	2.25955400	-2.23935700
H	0.27488800	3.93754300	-1.78296700
H	0.47981900	2.67517400	-0.52753900
C	-4.24556000	0.57440800	-0.56406500
N	-4.39861600	1.93920900	-0.88011400
C	-4.61528000	-0.18153400	-1.66589200
H	-4.59799100	-1.26123300	-1.69832700
C	-4.80184300	-3.99745300	0.01525800
C	-5.36936600	-3.79750100	1.42481000
C	-5.15791300	-5.40092700	-0.51311300
H	-5.29276200	-3.26655500	-0.64440800
H	-5.11673700	-2.81358200	1.82727900
H	-6.46052400	-3.89633600	1.40704300
H	-4.98685200	-4.55598400	2.11862800
H	-4.80626000	-5.53879700	-1.54074100
H	-4.68405600	-6.17048600	0.10781000
H	-6.24218200	-5.56395500	-0.49313100
C	-0.12352300	-4.11077200	1.98999900
C	-0.31309200	-5.52510900	2.56494800

C	-0.33655400	-3.03581600	3.06896100
H	0.91529000	-4.03861700	1.65905900
H	-0.12803300	-6.28803000	1.80077000
H	0.37932500	-5.69752400	3.39737100
H	-1.33200200	-5.67113700	2.94258600
H	-0.19771300	-2.03505700	2.65195500
H	-1.34445600	-3.09631800	3.49057100
H	0.37805300	-3.17110700	3.88965800
C	-0.89509800	-2.99629300	-2.97366900
C	-1.66526900	-1.80133000	-3.56310200
C	-0.97286000	-4.20218500	-3.92767800
H	0.15116300	-2.69169500	-2.89001900
H	-1.65421100	-0.95715300	-2.87264500
H	-1.19514200	-1.48700900	-4.50232700
H	-2.70606600	-2.05884200	-3.79475000
H	-0.37980800	-5.04788400	-3.56586600
H	-2.00933100	-4.54512100	-4.03455200
H	-0.60347300	-3.92971200	-4.92344300
C	2.30944600	6.56845300	-0.64763300
C	3.17570900	7.52292000	0.19373600
C	0.81527700	6.84085300	-0.41350700
H	2.52122400	6.77231500	-1.70581900
H	4.24105000	7.35446300	0.00459000
H	2.94393600	8.56907100	-0.03891000
H	2.99842900	7.36759300	1.26443800
H	0.18816700	6.20790500	-1.04960100
H	0.53494700	6.65163300	0.62949200
H	0.57387200	7.88683900	-0.63514900
C	4.48922600	2.30254300	-2.23548600
C	5.74187600	3.05137400	-2.72064700
C	3.53069500	1.99706900	-3.39923000
H	4.82649700	1.34349100	-1.83326400
H	6.42835400	3.24770800	-1.88969900
H	6.27418400	2.45772400	-3.47292400
H	5.48556300	4.01404300	-3.17763500
H	2.68977500	1.38883400	-3.05450500
H	3.13459100	2.91969300	-3.84004500
H	4.05099300	1.44390800	-4.19009200

C	2.58320800	2.62347100	2.51891900
C	1.19609600	3.01236800	3.05837700
C	3.67127500	3.02518800	3.53159900
H	2.59268200	1.53550700	2.41616700
H	0.42156900	2.85006800	2.30515700
H	0.95419300	2.40070500	3.93487300
H	1.15854800	4.06196600	3.37471800
H	4.66005000	2.67597200	3.22089300
H	3.71713700	4.11604800	3.63678400
H	3.45563100	2.59751900	4.51796600
C	-3.82940300	0.02984300	0.69620600
C	-3.66954300	0.68418400	1.91776000
H	-3.73925000	-1.05340300	0.69427600
H	-3.90473400	1.73663500	2.02481600
C	-3.59533100	-0.02354700	3.22197500
O	-3.40075400	0.56138600	4.27303200
O	-3.77855900	-1.35694500	3.12298800
C	-3.76488000	-2.10219700	4.36280300
H	-3.53495100	-3.12356600	4.05567800
H	-2.95769000	-1.72057100	4.99166400
C	-5.10985200	-2.02114100	5.06565000
H	-5.09674900	-2.64888800	5.96375700
H	-5.32354700	-0.99259300	5.36755100
H	-5.91096600	-2.37566600	4.40901300
H	-1.50453600	0.14103500	-0.08438200

int10-S

C	5.66360500	4.09361900	-2.25411800
C	4.62675100	3.74005500	-1.42032500
C	4.46985700	2.39823700	-0.97993900
C	5.38917800	1.41279100	-1.46472900
C	6.45774600	1.81426400	-2.30969400
C	6.59869800	3.12712000	-2.69366700
H	5.76073200	5.12417700	-2.58274900
H	3.91287300	4.48827200	-1.09942900
C	3.41075000	1.98473600	-0.10265000
C	5.20026100	0.05032500	-1.12282900
H	7.15349600	1.05624500	-2.65978800

H	7.41538400	3.42220600	-3.34583300
C	4.12665700	-0.37170300	-0.36760900
C	3.25848200	0.63013000	0.13589600
H	5.91188500	-0.68463200	-1.48597800
C	2.45845900	2.96206900	0.49065500
C	2.89735800	4.08187400	1.27330900
C	1.09772500	2.80158800	0.30203200
C	4.24942000	4.26179700	1.66946600
C	1.92672700	5.04389400	1.70265400
C	0.10991100	3.72726600	0.72327900
C	4.62742500	5.35492000	2.41642000
H	4.98581400	3.52045600	1.38321900
C	2.35352200	6.16787700	2.45895700
C	0.55828600	4.85162300	1.38551600
C	3.67490300	6.32580700	2.80629900
H	5.66564800	5.47011000	2.71387200
H	1.60805300	6.89511300	2.76968700
H	-0.16152600	5.60158100	1.69809000
H	3.98810300	7.18628000	3.39019300
O	2.19360400	0.21043200	0.94831100
O	0.64420500	1.68081900	-0.40442200
P	0.72496900	0.22843600	0.26892700
O	0.52822600	-0.84883900	-0.74490200
O	-0.22371100	0.21934200	1.53121100
C	3.91453300	-1.82196200	-0.06644700
C	3.35877100	-2.67850500	-1.04556300
C	4.31672300	-2.33634000	1.18189300
C	3.26053700	-4.04376200	-0.76089200
C	4.18338600	-3.70870400	1.42218300
C	3.67489300	-4.58202200	0.46118000
H	2.85540500	-4.70589200	-1.51959100
H	4.49918300	-4.11211000	2.38121000
C	-1.33762100	3.49374100	0.44257400
C	-2.24460400	3.28917600	1.50645000
C	-1.80439800	3.50056100	-0.89135000
C	-3.60737800	3.14430600	1.21522200
C	-3.16798400	3.33387000	-1.12752400
C	-4.09108500	3.16245700	-0.09396100

H	-3.53040500	3.31498500	-2.14857500
H	-4.30232800	3.01007300	2.03842300
Pd	-1.58495600	-0.92605700	-1.45629400
C	-3.44930300	-4.20661400	-0.86460700
C	-2.89469400	-3.45579700	0.16813400
C	-1.53006900	-3.60194400	0.49415900
C	-0.72916300	-4.43943000	-0.28772700
C	-1.28691000	-5.14659300	-1.35368700
C	-2.64870900	-5.05093800	-1.63465600
H	-4.50963700	-4.09776600	-1.06842300
H	0.32935800	-4.53301800	-0.08532400
H	-0.64730200	-5.78378100	-1.95757300
H	-3.08518500	-5.61976400	-2.44938400
C	-4.05245900	-2.75034300	2.23253400
C	-4.11380900	-3.94940900	2.94259900
C	-4.35625100	-1.49611400	2.82561700
C	-4.50254500	-3.88234100	4.27730700
H	-3.86178300	-4.89309900	2.46916000
C	-4.75307900	-1.45972000	4.17314900
C	-4.82111500	-2.65088400	4.88528300
H	-4.56270800	-4.79660000	4.86066300
H	-4.99562800	-0.51294700	4.64775300
H	-5.12330200	-2.63683800	5.92862000
S	-0.93224600	-2.75476300	1.94325300
C	0.86546400	-3.02420400	1.85478700
H	1.29537900	-2.41397200	2.65065900
H	1.11562600	-4.06928600	2.04013900
H	1.27244800	-2.70253800	0.89634800
C	-3.75697100	-1.13675000	0.65223800
N	-3.69532100	-2.52814000	0.90122200
C	-4.15509100	-0.50525800	1.81329800
H	-4.30776800	0.55843200	1.90313700
C	-5.56771400	3.06822300	-0.44649300
C	-6.17160000	4.48404700	-0.51702800
C	-6.39120000	2.17605000	0.48983200
H	-5.61703900	2.62739700	-1.44981800
H	-5.61710800	5.11437500	-1.22046500
H	-7.21932200	4.44705100	-0.83894400

H	-6.13418800	4.96774400	0.46693600
H	-6.00206300	1.15442300	0.51965800
H	-6.40865500	2.56472600	1.51541200
H	-7.43027400	2.13057300	0.14449700
C	-0.89064700	3.72202400	-2.08988400
C	-1.21108100	5.06186900	-2.77644000
C	-0.97350300	2.55197600	-3.08480300
H	0.14260200	3.78689500	-1.73996000
H	-1.11721200	5.89700400	-2.07300400
H	-0.52583700	5.23889300	-3.61409500
H	-2.23348100	5.06858000	-3.17064900
H	-0.75435200	1.60101300	-2.58843500
H	-1.97508700	2.46816000	-3.51632300
H	-0.25491300	2.69618400	-3.90125400
C	-1.79425300	3.22277200	2.96233300
C	-2.30898000	1.96385200	3.68015100
C	-2.21651200	4.48797100	3.73052400
H	-0.70209200	3.17206900	2.97686000
H	-2.04268200	1.05959300	3.12895100
H	-1.87332600	1.89896200	4.68407000
H	-3.39865700	1.97923600	3.79777400
H	-1.81586200	5.39492500	3.26532500
H	-3.30850700	4.58392100	3.75151600
H	-1.86081000	4.44973400	4.76709900
C	3.62630600	-6.07894100	0.72243000
C	4.66687800	-6.80980700	-0.14540200
C	2.22719300	-6.67972500	0.51438100
H	3.90138200	-6.23475300	1.77418500
H	5.67124900	-6.40795200	0.02444700
H	4.68026300	-7.88229200	0.08148000
H	4.43587000	-6.69242700	-1.21066700
H	1.48454200	-6.20658000	1.16497700
H	1.89018000	-6.55876700	-0.52171000
H	2.23306100	-7.75305500	0.73540400
C	4.90477200	-1.44461100	2.26820900
C	6.30762600	-1.91427600	2.68843800
C	3.96254500	-1.34136400	3.47978800
H	5.01518800	-0.43734500	1.85677000

H	6.97837700	-1.96975800	1.82414900
H	6.74109000	-1.21813500	3.41567100
H	6.27910100	-2.90584400	3.15418700
H	2.99909200	-0.91589100	3.18415000
H	3.78463500	-2.32575100	3.92896300
H	4.39727400	-0.69499500	4.25111000
C	2.90300900	-2.16016400	-2.40512800
C	1.60646800	-2.82319600	-2.89971900
C	4.01517100	-2.33500600	-3.45615600
H	2.69779400	-1.09126200	-2.30053200
H	0.82890600	-2.79968000	-2.13450000
H	1.23235100	-2.28881000	-3.78032400
H	1.76977400	-3.86558300	-3.19963300
H	4.92576000	-1.79693300	-3.17767300
H	4.27300900	-3.39476000	-3.57123700
H	3.68367000	-1.95847800	-4.43090600
C	-3.50755600	-0.48316800	-0.62289700
C	-3.64288000	-1.04220600	-1.91512900
H	-3.57277600	0.60009700	-0.55656000
H	-3.89764700	-2.08369600	-2.06565400
C	-3.89742400	-0.16683100	-3.07326800
O	-4.06672800	1.04261400	-3.05922600
O	-3.93752100	-0.90489900	-4.21785300
C	-4.06702400	-0.16862800	-5.44748300
H	-4.56400400	-0.85778600	-6.13530400
H	-4.70705200	0.70038700	-5.27721800
C	-2.69992900	0.24896300	-5.96976600
H	-2.80303400	0.76544700	-6.93129400
H	-2.21220900	0.92663000	-5.26387200
H	-2.05785600	-0.62655700	-6.11300800
H	-0.74765100	-0.63063600	1.58533000

TS-S1

C	5.59365300	-1.89268300	-4.25942100
C	5.11846700	-1.38592900	-3.07052300
C	3.98254200	-1.95527000	-2.43432000
C	3.31232400	-3.03972600	-3.08857000
C	3.83793200	-3.54880800	-4.30536900

C	4.95832000	-2.99420800	-4.87940900
H	6.46185400	-1.43856500	-4.72852900
H	5.60870700	-0.53729400	-2.60940700
C	3.46195700	-1.47098700	-1.18836000
C	2.11478800	-3.56067000	-2.53366200
H	3.32451000	-4.37947700	-4.78329800
H	5.34753700	-3.38840100	-5.81372900
C	1.55571900	-3.02820400	-1.39331400
C	2.27110100	-1.99988400	-0.72249200
H	1.62198800	-4.38428400	-3.04084400
C	4.11120100	-0.37850000	-0.41761500
C	5.47395400	-0.45779200	0.02256900
C	3.37269200	0.74880100	-0.08577700
C	6.26283600	-1.63046800	-0.12745200
C	6.06130400	0.67523200	0.67213300
C	3.94070800	1.88298100	0.55709800
C	7.57003500	-1.66581400	0.30359800
H	5.81825900	-2.50818200	-0.58114300
C	7.41614200	0.61097100	1.09193300
C	5.27540500	1.83427200	0.89472600
C	8.16031600	-0.53147900	0.90919500
H	8.15185500	-2.57525500	0.18288300
H	7.84983400	1.48338400	1.57428100
H	5.74044100	2.70413700	1.34930200
H	9.19421700	-0.57073400	1.23992200
O	1.75228200	-1.52488500	0.46600900
O	2.03851700	0.82336600	-0.44749300
P	0.93193100	-0.08417800	0.38366500
O	-0.22145100	-0.24243900	-0.60053300
O	0.69366400	0.36857900	1.78204800
C	0.25469700	-3.49655200	-0.82586000
C	-0.97409300	-3.04440600	-1.37536200
C	0.25576600	-4.36178700	0.28135000
C	-2.17000100	-3.47195500	-0.77144400
C	-0.96315300	-4.81820700	0.80348100
C	-2.18221300	-4.40483200	0.28473700
H	-3.11795500	-3.18487100	-1.21858400
H	-0.94572900	-5.51529100	1.63397000

C	3.11310800	3.10935200	0.76862200
C	2.48807200	3.35184500	2.00959000
C	2.95681600	4.01598900	-0.30077400
C	1.70565100	4.50384300	2.14623700
C	2.16507000	5.15210700	-0.11349300
C	1.52370000	5.40990100	1.10035800
H	2.03901500	5.84758200	-0.93924100
H	1.21384300	4.70114000	3.09402000
Pd	-1.92413100	-1.03038700	0.53965300
C	-6.63374700	0.95162200	0.41635200
C	-5.70308900	1.42281600	-0.53497100
C	-6.26874900	2.01492700	-1.70758400
C	-7.60964000	1.70976400	-2.00896700
C	-8.46011000	1.05297800	-1.12941400
C	-7.97890500	0.75008900	0.13847000
H	-6.30069000	0.77253800	1.42437600
H	-8.00915900	2.04840000	-2.95641100
H	-9.48872100	0.85209100	-1.41139000
H	-8.63183100	0.36654600	0.91592200
S	-5.54223600	3.33621600	-2.68928200
C	-7.01619500	4.31691100	-3.14195500
H	-6.61508800	5.28262400	-3.46258300
H	-7.58115800	3.88295200	-3.97018100
H	-7.66639100	4.46359800	-2.27644400
C	-3.89152400	0.57726400	0.92790400
C	-4.55825700	-0.59811000	1.61733700
H	-5.01740700	-0.31062900	2.57128100
H	-5.32762500	-1.02646400	0.97442600
C	-3.43573200	-1.61091300	1.80635800
H	-3.69362900	-2.61686500	1.49410600
C	-2.79169200	-1.56944700	3.14993500
O	-3.03205700	-0.73925300	4.01133300
O	-1.87097900	-2.54858700	3.28355200
C	-1.05877200	-2.52456200	4.48602000
H	-1.72144800	-2.41423600	5.34902000
H	-0.60306700	-3.51630500	4.50293200
C	-0.01040400	-1.42536800	4.44045900
H	-0.47353800	-0.43959400	4.51223700

H	0.67864900	-1.54822200	5.28451900
H	0.56019000	-1.45355000	3.50977100
N	-4.31690200	1.29372200	-0.18631300
C	-3.16416300	2.00997100	-0.68127800
C	-2.57205500	0.96993300	1.28066200
H	-2.16629400	0.84990200	2.27743900
C	-2.15806700	1.96226400	0.30937900
C	-2.84764300	2.46758600	-1.95647100
H	-3.48159500	2.29092800	-2.81090300
C	-1.62222900	3.11365100	-2.13654200
H	-1.37127700	3.48884500	-3.12414100
C	-0.70781700	3.25406400	-1.08722300
H	0.22847100	3.77238100	-1.24182200
C	-0.94846700	2.63676000	0.13601800
H	-0.18883200	2.60952800	0.90734200
C	0.64565400	6.63651000	1.28169800
C	-0.58501200	6.58945300	0.35997200
C	1.43952600	7.93769000	1.07796700
H	0.28418100	6.62576300	2.31869300
H	-1.16334800	5.67435900	0.51919500
H	-1.23781500	7.45201000	0.54030700
H	-0.28659900	6.61017100	-0.69493900
H	2.30133000	7.98310100	1.75244200
H	1.81529700	8.01324000	0.05084100
H	0.80648400	8.81284600	1.26693300
C	3.61879900	3.77842200	-1.65348300
C	4.48174700	4.97515600	-2.08523100
C	2.58272700	3.41739000	-2.73213200
H	4.28915100	2.92067600	-1.55272600
H	5.23174100	5.21467300	-1.32341500
H	5.00378500	4.75091400	-3.02291200
H	3.87537400	5.87313400	-2.25084600
H	1.99598100	2.54476900	-2.43145600
H	1.89302800	4.25222600	-2.91034500
H	3.08155600	3.19028100	-3.68200300
C	2.69481600	2.41861500	3.19643800
C	1.45667400	2.27744800	4.09303800
C	3.90450400	2.88194800	4.03035000

H	2.91128000	1.42354200	2.79856200
H	0.59006500	1.97215400	3.50475800
H	1.63914500	1.50310300	4.84691600
H	1.22806200	3.20584800	4.63034500
H	4.82087500	2.90902100	3.43337700
H	3.73392600	3.88911900	4.43019000
H	4.07052200	2.20412500	4.87631500
C	-1.02445100	-2.26122600	-2.68327400
C	-2.16197000	-1.23506800	-2.79148000
C	-1.13349000	-3.26118000	-3.85424500
H	-0.08470400	-1.71300400	-2.77593700
H	-2.08467200	-0.47274500	-2.01483400
H	-2.09728200	-0.72685500	-3.76036000
H	-3.15220300	-1.70385200	-2.73964600
H	-0.32688100	-3.99838600	-3.84084500
H	-2.08431700	-3.80619800	-3.80643700
H	-1.09376300	-2.73083000	-4.81258000
C	-3.50119100	-5.01724500	0.72635200
C	-3.54315500	-5.41558600	2.20770200
C	-3.82626000	-6.22743500	-0.17226200
H	-4.28886200	-4.27036100	0.54976100
H	-3.22964700	-4.59165500	2.85426300
H	-4.55733300	-5.72060800	2.48820200
H	-2.88267400	-6.26751200	2.40588300
H	-3.85193900	-5.94114300	-1.22903000
H	-3.06211200	-7.00425800	-0.05364600
H	-4.79846500	-6.65920900	0.09317100
C	1.54889900	-4.83919300	0.92538500
C	1.69152500	-6.36760800	0.82977600
C	1.65430700	-4.34986000	2.37931800
H	2.38463100	-4.40205800	0.37348100
H	1.64568000	-6.70249600	-0.21242100
H	2.65138900	-6.68795800	1.25092000
H	0.89712700	-6.88220400	1.38286000
H	1.58537300	-3.26010100	2.42057000
H	0.85419200	-4.77245700	2.99876500
H	2.61258100	-4.65420100	2.81616300

L8

C	0.96218400	5.14034100	-2.46895400
C	0.41984200	4.17032400	-1.65638300
C	1.23476000	3.16007700	-1.07976400
C	2.62878400	3.14347900	-1.41140800
C	3.15895300	4.16894900	-2.23797900
C	2.34755900	5.15277700	-2.75211700
H	0.31779600	5.90014600	-2.90147400
H	-0.64403200	4.16853300	-1.45489600
C	0.71679200	2.13987700	-0.21026800
C	3.44601300	2.07368200	-0.96471000
H	4.22054000	4.14851900	-2.47051500
H	2.76316900	5.92848100	-3.38851600
C	2.93120800	1.02845400	-0.23093000
C	1.56971500	1.11931900	0.16352500
H	4.49125800	2.05823600	-1.25723900
C	-0.70277900	2.11667300	0.23391200
C	-1.31423500	3.23736600	0.89115600
C	-1.47424600	0.98222500	0.03603900
C	-0.57959900	4.38084400	1.30451600
C	-2.71599000	3.19101100	1.17962100
C	-2.85883100	0.90306000	0.33285500
C	-1.20509700	5.43761700	1.92632100
H	0.48967600	4.41067000	1.13391400
C	-3.33334700	4.30520900	1.80706900
C	-3.45734700	2.02573800	0.86219900
C	-2.59826100	5.40926400	2.16913300
H	-0.62170700	6.29859200	2.23952600
H	-4.40016700	4.25860900	2.00951200
H	-4.52192400	2.00628800	1.07471000
H	-3.08003300	6.25275500	2.65486500
O	1.09346700	0.11303600	1.00326800
O	-0.88826500	-0.15257200	-0.53718000
P	0.10738200	-1.02539000	0.39605500
O	-0.45513000	-1.85992800	1.46835600
C	3.68940700	-0.22132100	0.07741800
C	3.98388400	-1.13305500	-0.97061500
C	4.01834500	-0.54636100	1.40899300

C	4.57855000	-2.35638100	-0.64503300
C	4.62746000	-1.77675900	1.67570200
C	4.90380800	-2.70102000	0.67080300
H	4.80103400	-3.05797900	-1.44278700
H	4.88289000	-2.02832800	2.70159600
C	-3.62077200	-0.35521200	0.07756600
C	-4.03208000	-1.15840100	1.16139000
C	-3.92483000	-0.73493800	-1.24720400
C	-4.77228300	-2.31425900	0.89174100
C	-4.65791800	-1.90228100	-1.46242000
C	-5.09518200	-2.70496500	-0.40631300
H	-4.89429500	-2.19213600	-2.48268700
H	-5.09871000	-2.93768700	1.71968000
C	3.71782500	-0.80604200	-2.43847000
C	3.11558500	-1.97145400	-3.24025300
C	5.01934500	-0.32530600	-3.10821000
H	2.99695800	0.01445200	-2.47733900
H	2.17911500	-2.32326400	-2.79987200
H	2.89875300	-1.63867800	-4.26132400
H	3.80454600	-2.81997200	-3.31721200
H	5.45526700	0.52803900	-2.57953600
H	5.76777900	-1.12652900	-3.11437000
H	4.83215700	-0.02652100	-4.14604400
C	5.53068100	-4.04502600	0.99626600
C	6.88998900	-4.22205400	0.29952900
C	4.57640300	-5.20154100	0.65261800
H	5.70567500	-4.06775800	2.07993700
H	7.57632800	-3.40986600	0.56138200
H	7.35253000	-5.17185900	0.59127500
H	6.77791400	-4.22724000	-0.79089900
H	3.61900800	-5.09048900	1.17262600
H	4.37141300	-5.23606800	-0.42379800
H	5.01444400	-6.16408100	0.94045500
C	3.76336000	0.41654100	2.55990500
C	5.09505500	0.97722000	3.09040200
C	2.93728200	-0.22471000	3.68642400
H	3.18937900	1.26334300	2.17432600
H	5.65815100	1.47550800	2.29358700

H	4.91425200	1.70450800	3.89030100
H	5.72614000	0.17867200	3.49756800
H	1.99447100	-0.62378400	3.30338400
H	3.48463000	-1.04006200	4.17332400
H	2.70793300	0.52169400	4.45553000
C	-3.49670100	0.09897300	-2.44779600
C	-4.71833400	0.65464000	-3.19942000
C	-2.57262100	-0.69393100	-3.38699800
H	-2.92849700	0.95965300	-2.08375700
H	-5.35176700	1.24988900	-2.53257100
H	-4.40135600	1.29252800	-4.03299100
H	-5.33409200	-0.15333500	-3.61090600
H	-1.69411100	-1.05935200	-2.84816900
H	-3.09137000	-1.55639300	-3.82138800
H	-2.23234400	-0.05896600	-4.21374200
C	-5.88923200	-3.97405600	-0.66216800
C	-5.06294700	-5.00190000	-1.45341300
C	-7.22268900	-3.67556400	-1.36739200
H	-6.12128800	-4.41566400	0.31619300
H	-4.12540100	-5.23260000	-0.93701300
H	-5.62468600	-5.93429400	-1.58499800
H	-4.80989200	-4.61972100	-2.44922900
H	-7.82223400	-2.96326900	-0.79059500
H	-7.05498600	-3.24315100	-2.36067400
H	-7.80784400	-4.59360600	-1.49721300
C	-3.70918800	-0.80199300	2.60761900
C	-3.12790100	-1.98869600	3.39281400
C	-4.95124400	-0.23310600	3.31664700
H	-2.94301900	-0.02145300	2.59922400
H	-2.24459600	-2.38543100	2.88843500
H	-2.83570700	-1.65961300	4.39717600
H	-3.86087500	-2.79470200	3.51476300
H	-5.34149100	0.65080600	2.80069900
H	-5.75470900	-0.97859600	3.35006400
H	-4.71113900	0.05177000	4.34783800
O	0.91678700	-1.75192100	-0.78433500
H	1.79863500	-2.05727200	-0.49560400

1a

C	-2.63464000	-0.46633000	0.59719600
C	-1.46430400	-0.22776500	-0.17318600
C	-1.48956300	0.46855700	-1.38336500
C	-2.71993500	0.94689300	-1.81809500
C	-3.89411700	0.73324400	-1.06753100
C	-3.86251500	0.03247700	0.13099800
C	-2.23123700	-1.23195500	1.74585800
H	-0.58158500	0.63135500	-1.95461900
H	-2.77701200	1.49626600	-2.75341900
H	-4.83838100	1.12313100	-1.43705000
H	-4.77318900	-0.13321400	0.70035400
H	-2.86724300	-1.58196000	2.54704900
C	0.94651200	-0.89084100	0.03336000
C	1.76342300	0.25910400	0.04330200
C	1.44909800	-2.11301000	-0.41146000
C	3.07806200	0.14546600	-0.42858100
C	2.76539000	-2.21867500	-0.85734100
H	0.78672400	-2.97313400	-0.40749800
C	3.57199900	-1.08208100	-0.86974300
H	3.72981500	1.01052100	-0.44413400
H	3.15017300	-3.17361100	-1.20107000
H	4.59699300	-1.14419000	-1.22417200
C	-0.88187600	-1.43101200	1.64651000
H	-0.19252500	-1.94180000	2.30339100
N	-0.39535600	-0.81751500	0.49544400
S	1.05783100	1.75996200	0.68413900
C	2.49106100	2.88400300	0.69762400
H	2.85007300	3.09975500	-0.31227800
H	2.12788800	3.81205500	1.14564900
H	3.30631100	2.48958100	1.31019600

2a

C	2.18054300	-1.39315200	-0.00008600
C	2.00919700	-0.06934700	-0.00004100
H	1.33353300	-2.07139800	-0.00064000
H	3.17364200	-1.83299000	0.00038600
H	2.84827200	0.61988600	0.00051500

C	0.68928800	0.60618800	-0.00077300
O	0.55547300	1.81457900	0.00021100
O	-0.34724200	-0.26125800	-0.00025000
C	-1.66038600	0.33710000	0.00026900
H	-1.75632600	0.97705000	0.88321100
H	-1.75683100	0.97744700	-0.88232200
C	-2.67127900	-0.79244800	0.00032200
H	-2.55232000	-1.42195000	0.88791000
H	-2.55286700	-1.42153300	-0.88763400
H	-3.68712700	-0.38313200	0.00073200

HOAc

C	0.13414600	-0.13853300	-0.00014600
O	0.70214100	-1.19885200	0.00003600
O	0.85282200	1.02163900	0.00003000
H	0.25291900	1.78531800	-0.00000100
C	-1.37450000	0.03656100	0.00000800
H	-1.70041000	0.59384600	0.88673800
H	-1.70063400	0.59487000	-0.88598600
H	-1.84946100	-0.94449800	-0.00046300

9. References

1. G. Liao, T. Zhang, L. Jin, B.-J. Wang, C.-K. Xu, Y. Lan, Y. Zhao and B.-F. Shi, *Angew. Chem., Int. Ed.*, 2022, DOI: 10.1002/anie.202115221.
2. Gaussian 16, Revision A.03, Gaussian Inc.: Wallingford CT, 2016. M. J. Frisch, G. W. Trucks, H. B. Schlegel, G. E. Scuseria, M. A. Robb, J. R. Cheeseman, G. Scalmani, V. Barone, G. A. Petersson, H. Nakatsuji, X. Li, M. Caricato, A. V. Marenich, J. Bloino, B. G. Janesko, R. Gomperts, B. Mennucci, H. P. Hratchian, J. V. Ortiz, A. F. Izmaylov, J. L. Sonnenberg, D. Williams-Young, F. Ding, F. Lipparini, F. Egidi, J. Goings, B. Peng, A. Petrone, T. Henderson, D. Ranasinghe, V. G. Zakrzewski, J. Gao, N. Rega, G. Zheng, W. Liang, M. Hada, M. Ehara, K. Toyota, R. Fukuda, J. Hasegawa, M. Ishida, T. Nakajima, Y. Honda, O. Kitao, H. Nakai, T. Vreven, K. Throssell, J. A. Montgomery, Jr., J. E. Peralta, F. Ogliaro, M. J. Bearpark, J. J. Heyd, E. N. Brothers, K. N. Kudin, V. N. Staroverov, T. A. Keith, R. Kobayashi, J. Normand, K. Raghavachari, A. P. Rendell, J. C. Burant, S. S. Iyengar, J. Tomasi, M. Cossi, J. M. Millam, M. Klene, C. Adamo, R. Cammi, J. W. Ochterski, R. L. Martin, K. Morokuma, O. Farkas, J. B. Foresman, D. J. Fox.
3. (*a*) A. D. Becke, *J. Chem. Phys*., 1993, **98**, 5648; (*b*) C. Lee, W. Yang and R. G. Parr, *Phys. Rev. B: Condens. Matter Mater. Phys*., 1988, **37**, 785.
4. S. Grimme, S. Ehrlich and L. Goerigk, *J. Comp. Chem*., 2011, **32**, 1456.
5. (*a*) P. J. Hay and W. R. Wadt, *J. Chem. Phys*., 1985, **82**, 270; (*b*) W. R, Wadt and P. J. Hay, *J. Chem. Phys*., 1985, **82**, 284; (*c*) P. J. Hay and W. R. Wadt, *J. Chem. Phys.*, 1985, **82**, 299.
6. J.-D. Chai and M. Head-Gordon, *Phys. Chem. Chem. Phys.*, 2008, **10**, 6615.
7. (*a*) H. Stoll, P. Fuentealba, P. Schwerdtfeger, J. Flad, L. V. Szentpály and H. Preuss, *J. Chem. Phys.*, 1984, **81**, 2732; (*b*) P. Schwerdtfeger, M. Dolg, W. H. E. Schwarz, G. A. Bowmaker and P. D. W. Boyd, *J. Chem. Phys.* 1989, **91**, 1762; (*c*) D. Andrae, U. Huermann, M. Dolg, H. Stoll and H. Preu, *Theoretica Chimica Acta*, 1990, **77**, 123; (*d*) A. Nicklass, M. Dolg, H. Stoll and H. Preuss, *J. Chem. Phys.*, 1995, **102**, 8942; (*e*) G. Igel-Mann, H. Stoll and H. Preuss, *Molecular Physics*, 2006, **65**, 1321; (*f*) A. Bergner, M. Dolg, W. Küchle, H. Stoll and H. Preuß, *Molecular Physics*, 2006, **80**, 1431.
8. A. V. Marenich, C. J. Cramer and D. G. Truhlar, *J. Phys. Chem. B.*, 2009, **113**, 6378.
9. CYLview, 1.0b; C. Y. Legault, Universitéde Sherbrooke, 2009 (http://www.cylview.org).

10. NMR-Spectra

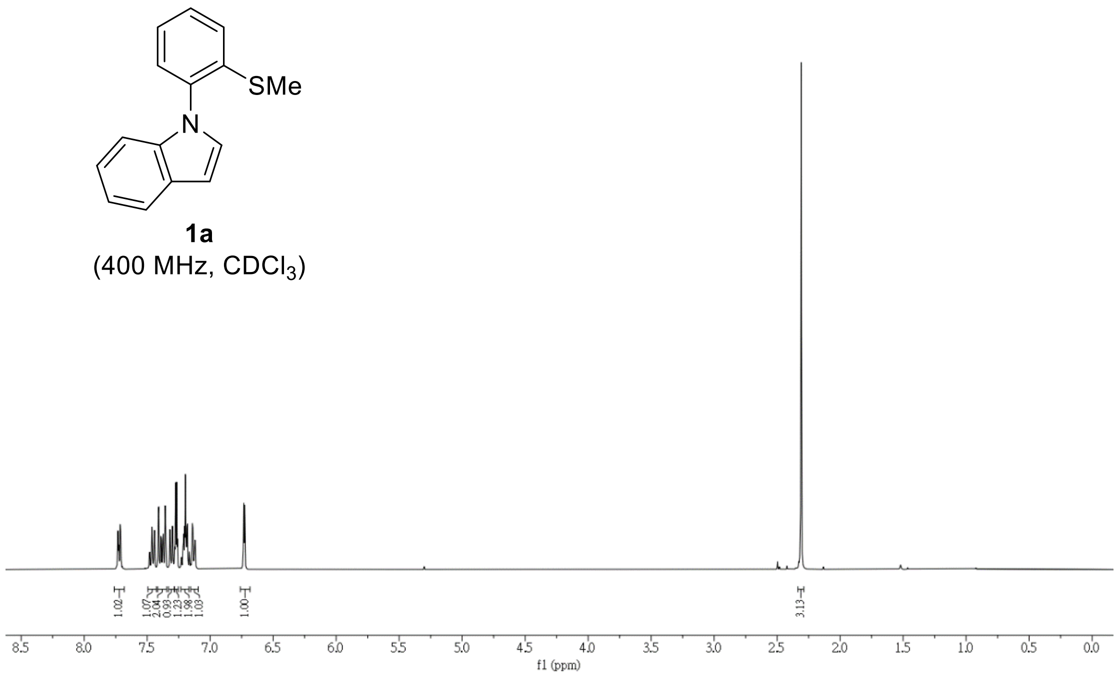

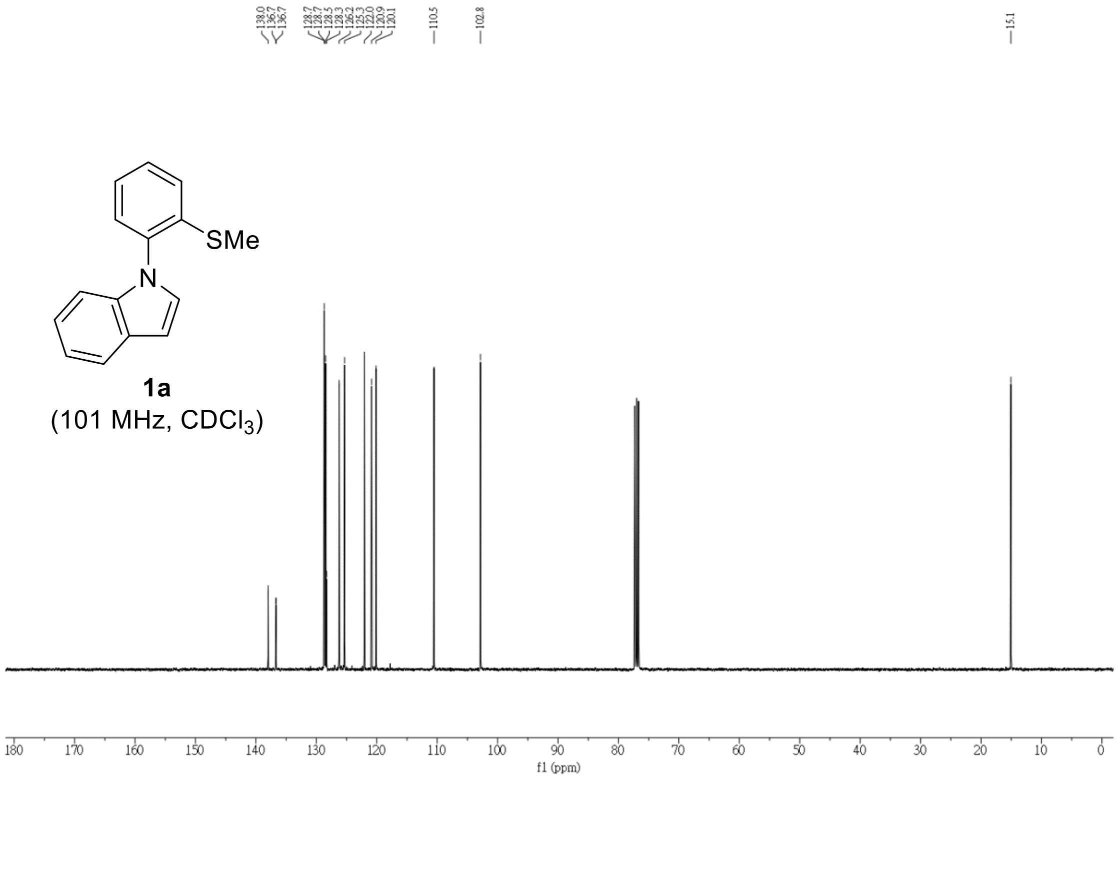

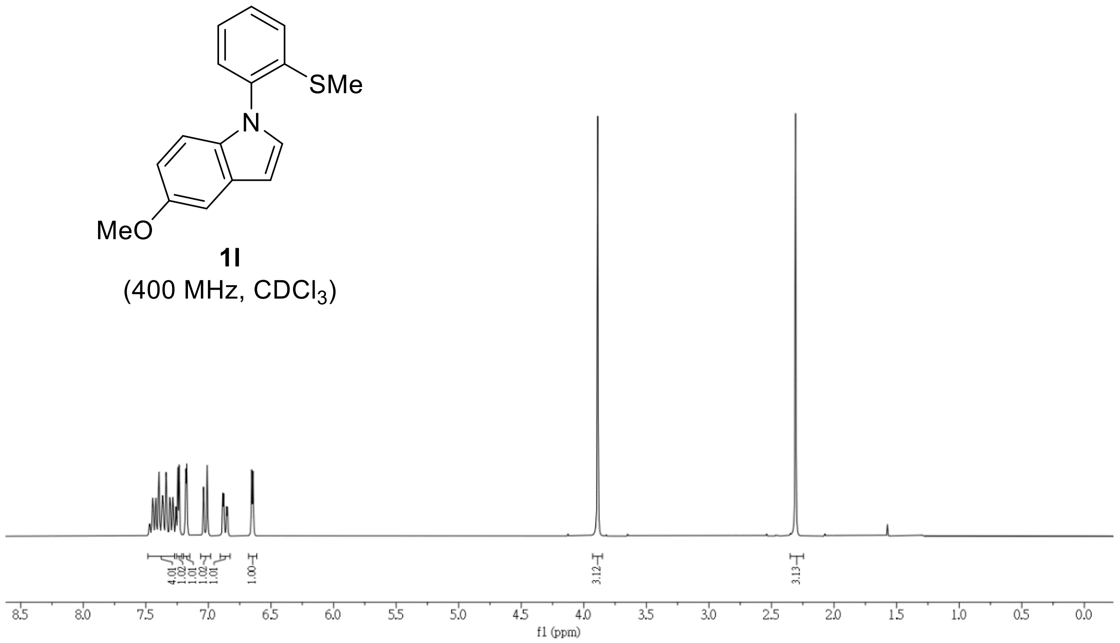
SMe
N
MeO
1l
(400 MHz, CDCl3)
4.01 1.02 1.01 1.02 1.01 1.00 3.12 3.13
8.5 8.0 7.5 7.0 6.5 6.0 5.5 5.0 4.5 4.0 3.5 3.0 2.5 2.0 1.5 1.0 0.5 0.0
f1 (ppm)

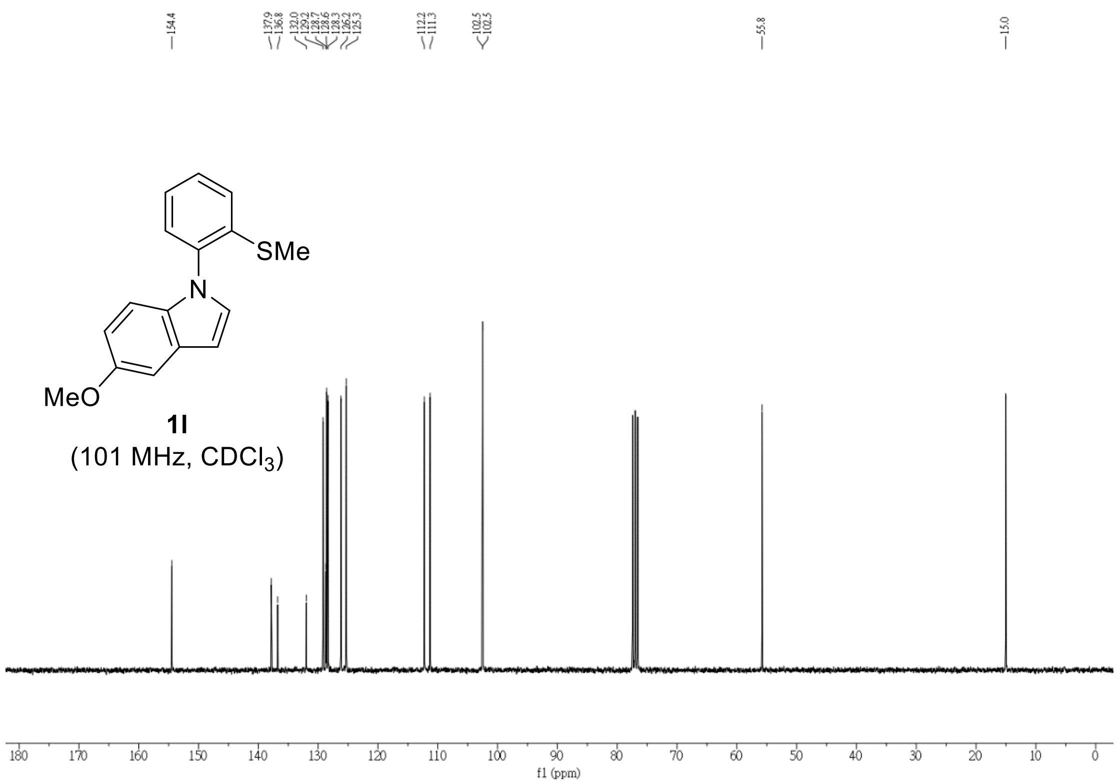
154.4
137.9 136.8 132.0 129.2 128.7 128.6 128.3 126.2 125.3
112.2 111.3
102.5 102.5
55.8
15.0
SMe
N
MeO
1l
(101 MHz, CDCl3)
180 170 160 150 140 130 120 110 100 90 80 70 60 50 40 30 20 10 0
f1 (ppm)

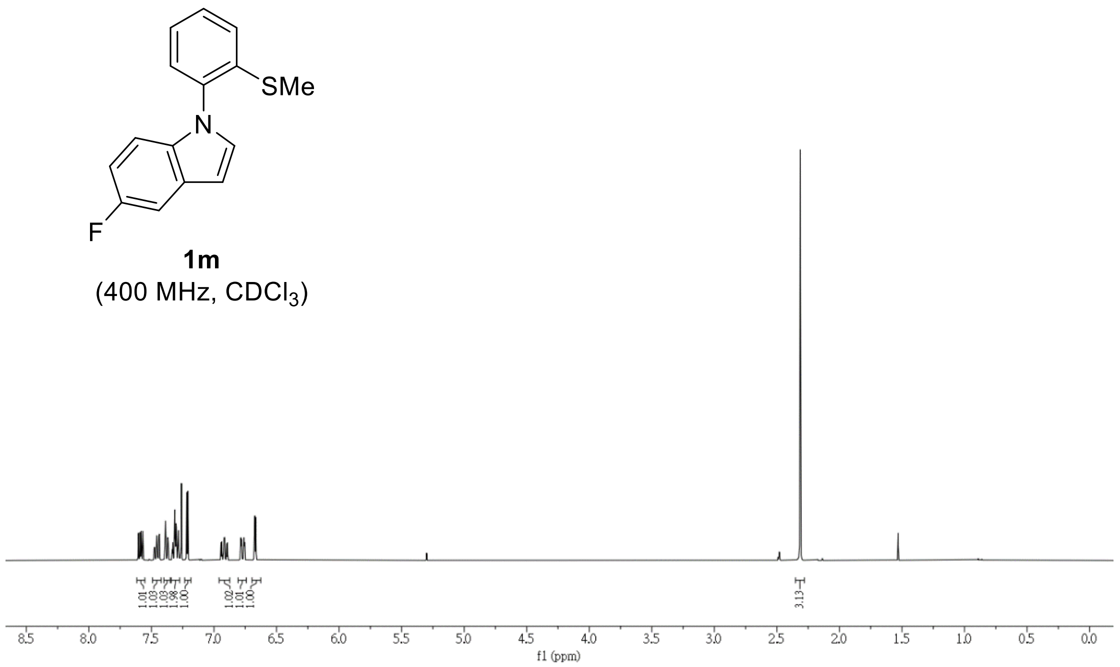
SMe
N
F
1m
(400 MHz, $CDCl_3$)
1.01 1.03 1.03 1.98 1.00 1.02 1.01 1.00 3.13
f1 (ppm)

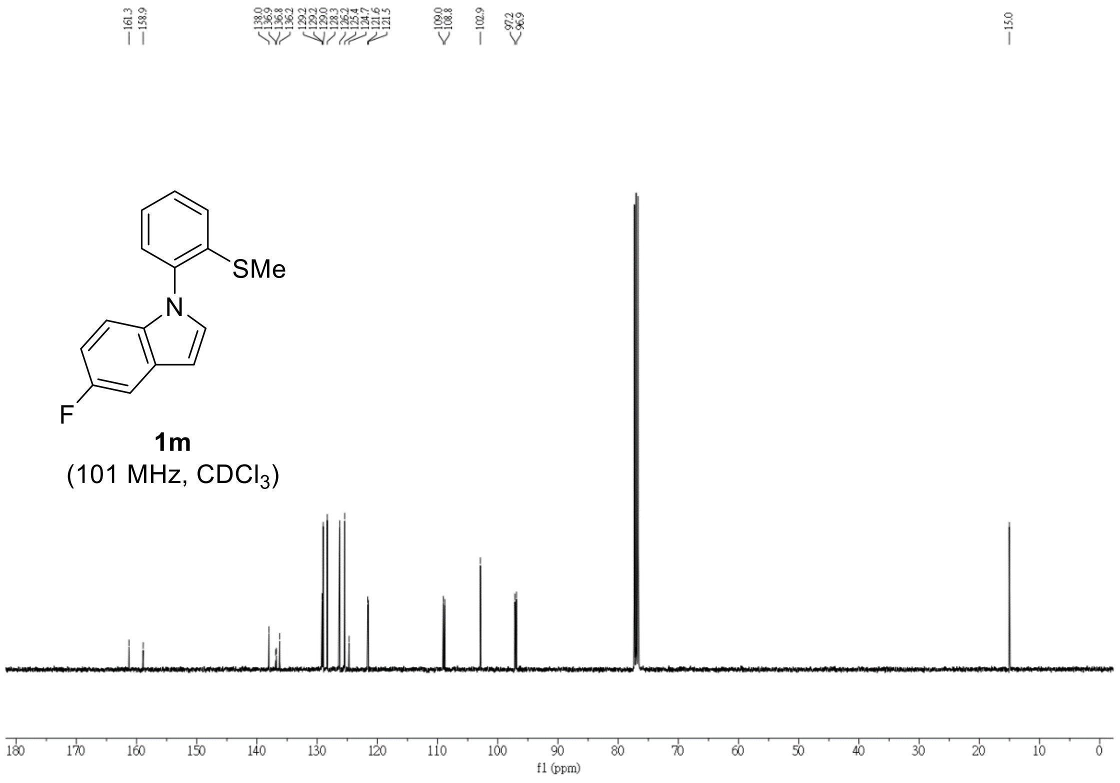
161.3 158.9 138.0 136.9 136.8 136.2 129.2 129.2 129.0 128.3 126.2 125.4 124.7 121.6 121.5 109.0 108.8 102.9 97.2 96.9 15.0
SMe
N
F
1m
(101 MHz, $CDCl_3$)
f1 (ppm)

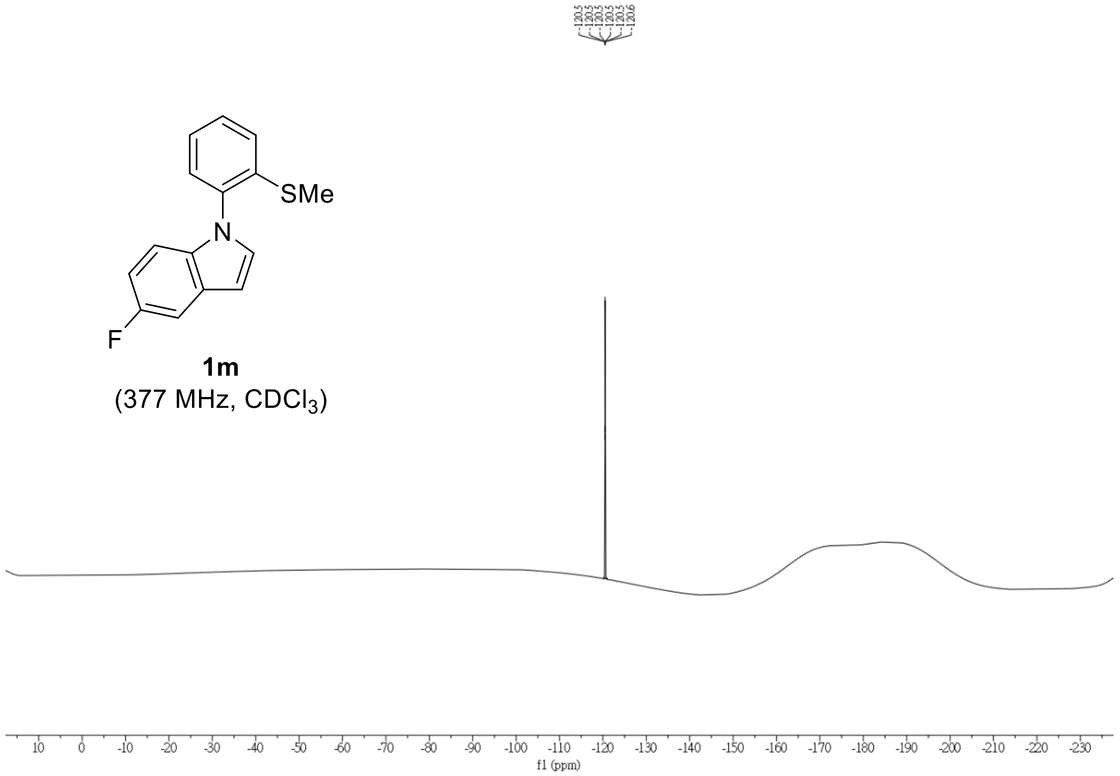
-120.5
-120.5
-120.5
-120.5
-120.5
-120.6
SMe
N
F
1m
(377 MHz, CDCl3)
10 0 -10 -20 -30 -40 -50 -60 -70 -80 -90 -100 -110 -120 -130 -140 -150 -160 -170 -180 -190 -200 -210 -220 -230
f1 (ppm)

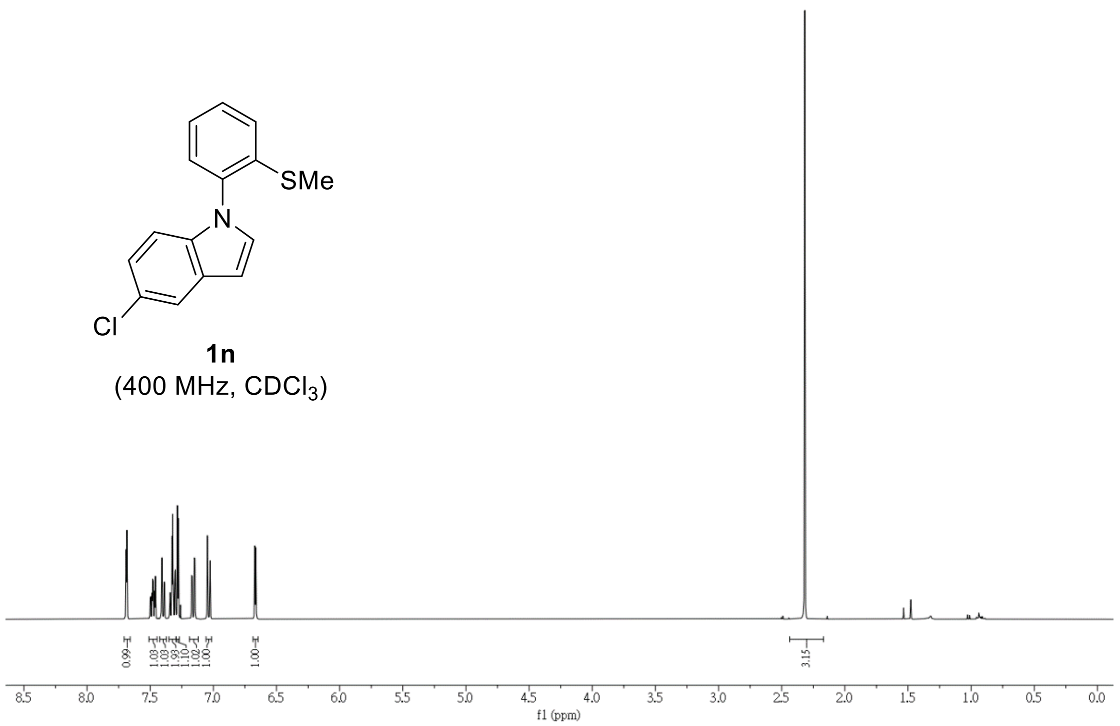
SMe
N
Cl
1n
(400 MHz, CDCl3)
0.99
1.03
1.03
1.93
1.10
1.02
1.00
1.00
3.15
f1 (ppm)

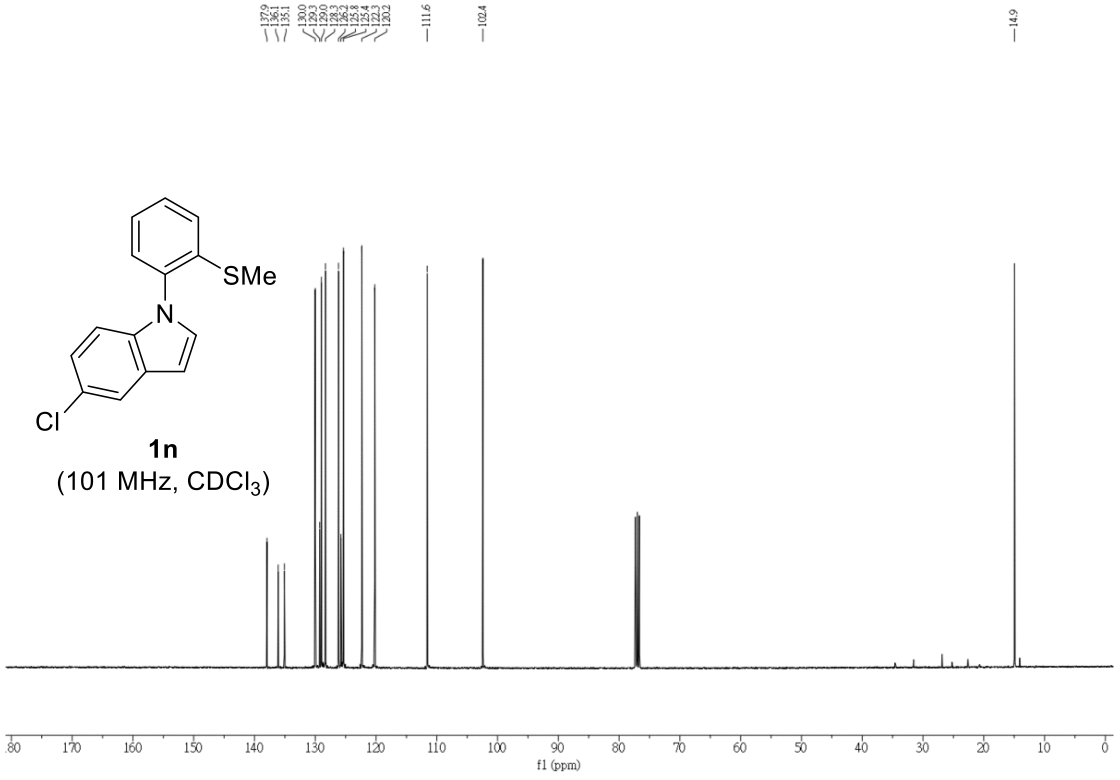
137.9
136.1
135.1
130.0
129.3
129.0
128.3
126.2
125.8
125.4
122.3
120.2
111.6
102.4
14.9
SMe
N
Cl
1n
(101 MHz, CDCl3)
f1 (ppm)

1a-D
(101 MHz, $CDCl_3$)

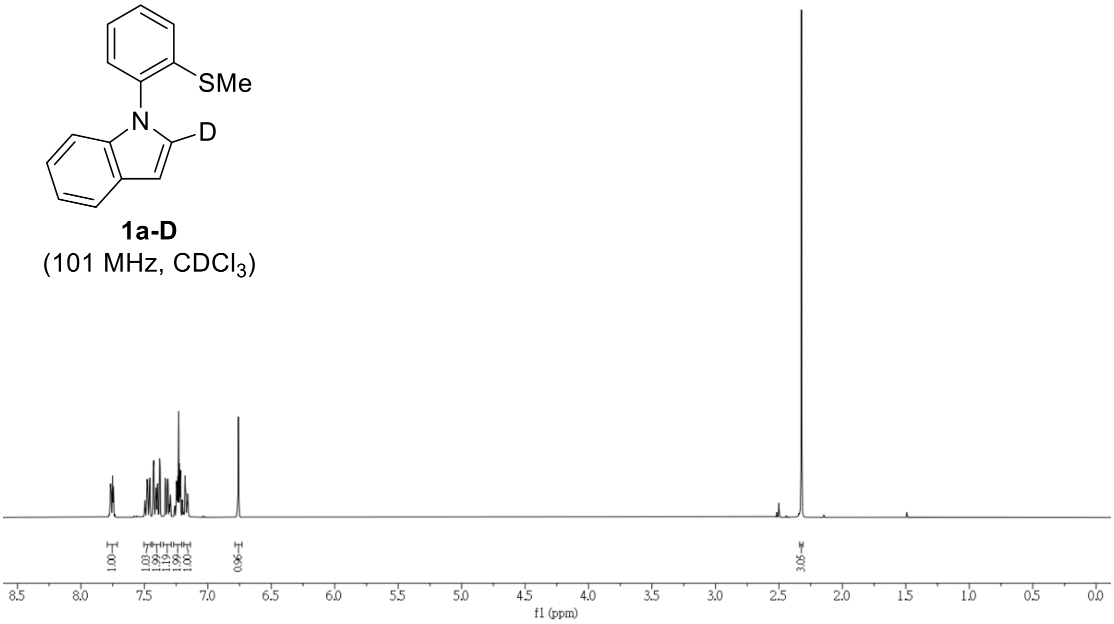

1a-D
(400 MHz, $CDCl_3$)

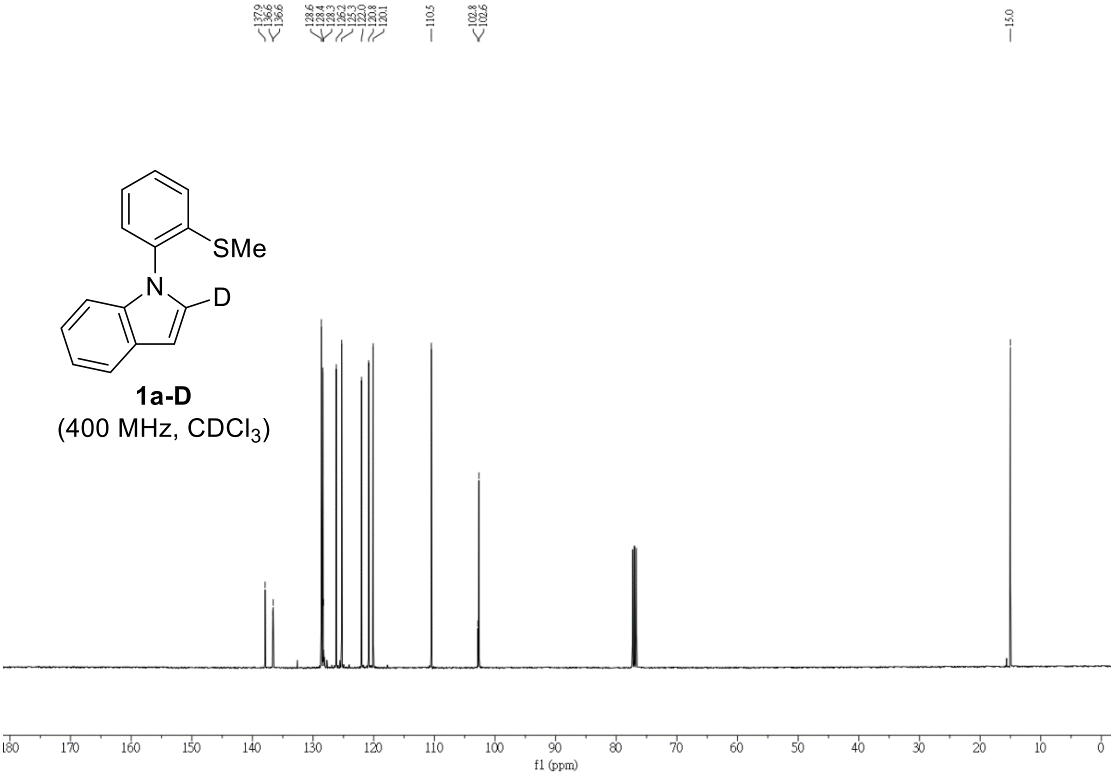

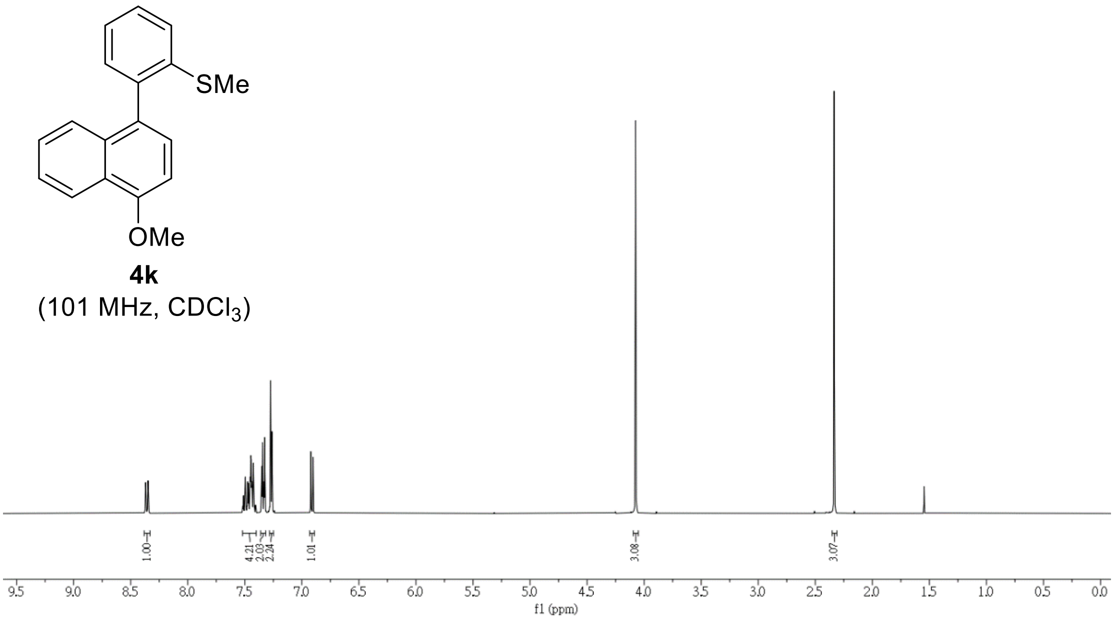

4k

(101 MHz, $CDCl_3$)

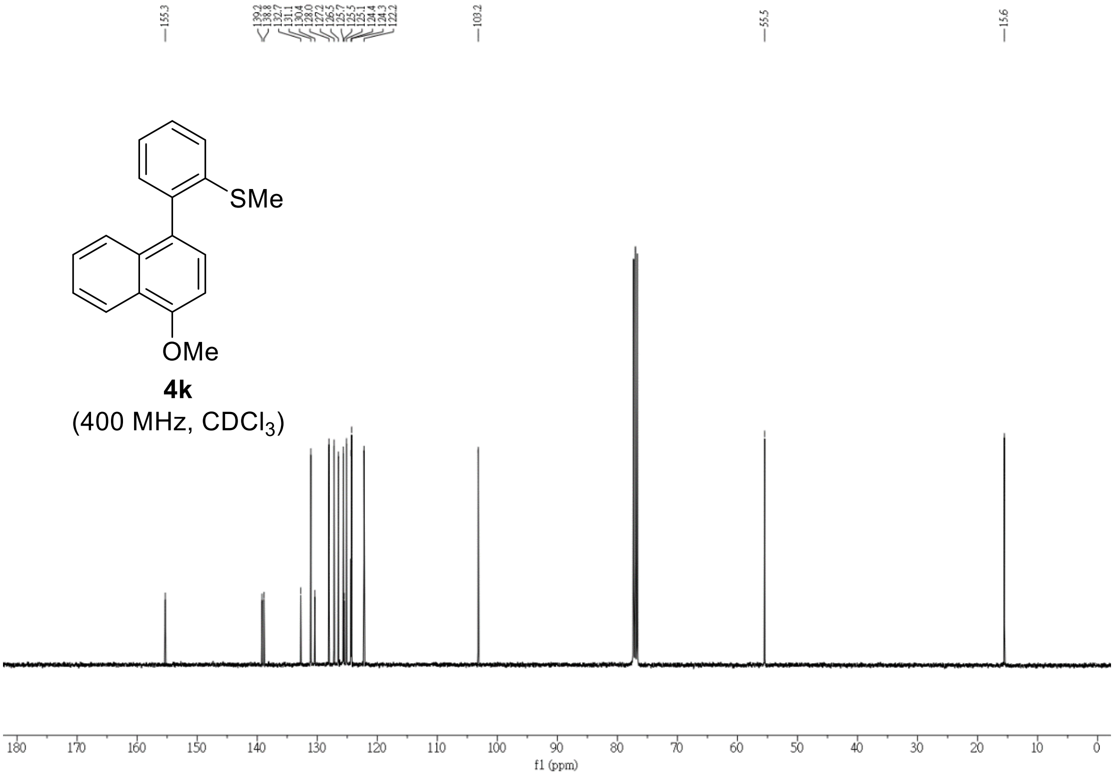

4k

(400 MHz, $CDCl_3$)

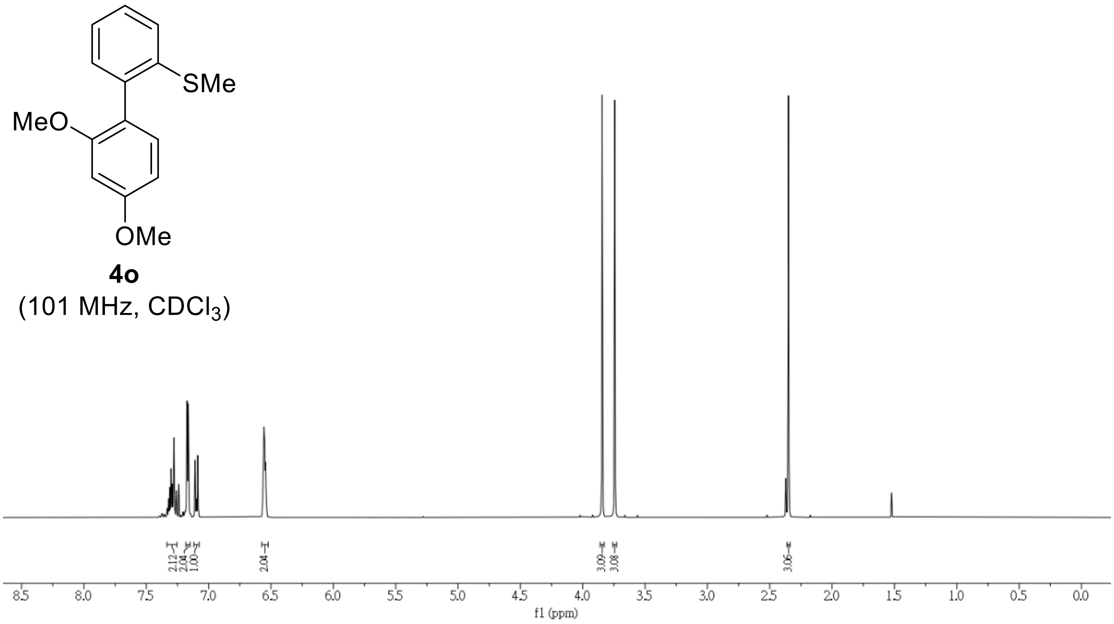
SMe
MeO
OMe
4o
(101 MHz, CDCl$_3$)
2.12
2.04
1.00
2.04
3.09
3.08
3.06
8.5 8.0 7.5 7.0 6.5 6.0 5.5 5.0 4.5 4.0 3.5 3.0 2.5 2.0 1.5 1.0 0.5 0.0
f1 (ppm)

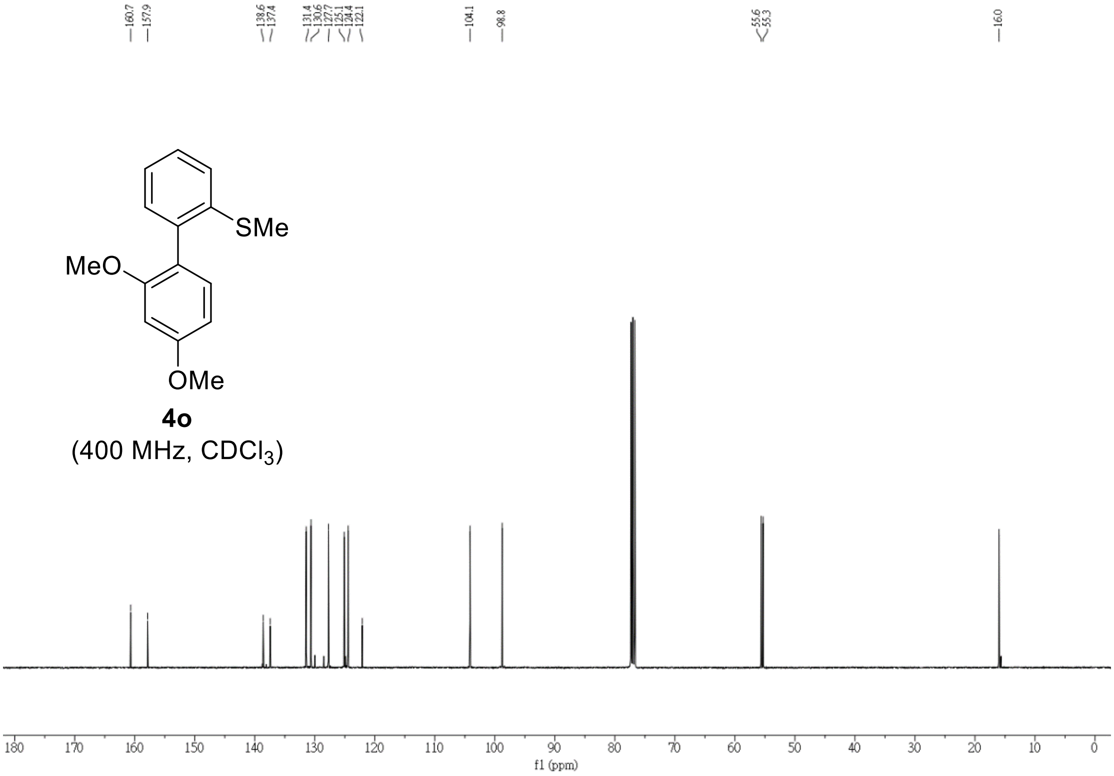
160.7
157.9
138.6
137.4
131.4
130.6
127.7
125.1
124.4
122.1
104.1
98.8
55.6
55.3
16.0
SMe
MeO
OMe
4o
(400 MHz, CDCl$_3$)
180 170 160 150 140 130 120 110 100 90 80 70 60 50 40 30 20 10 0
f1 (ppm)

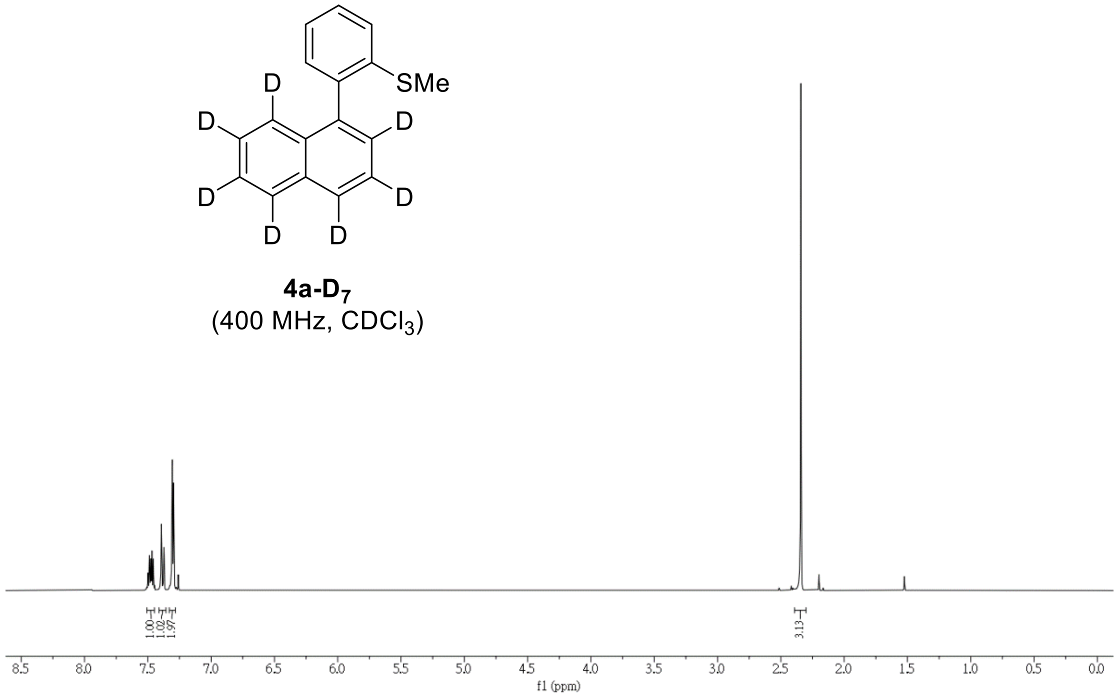

4a-D$_7$
(400 MHz, $CDCl_3$)

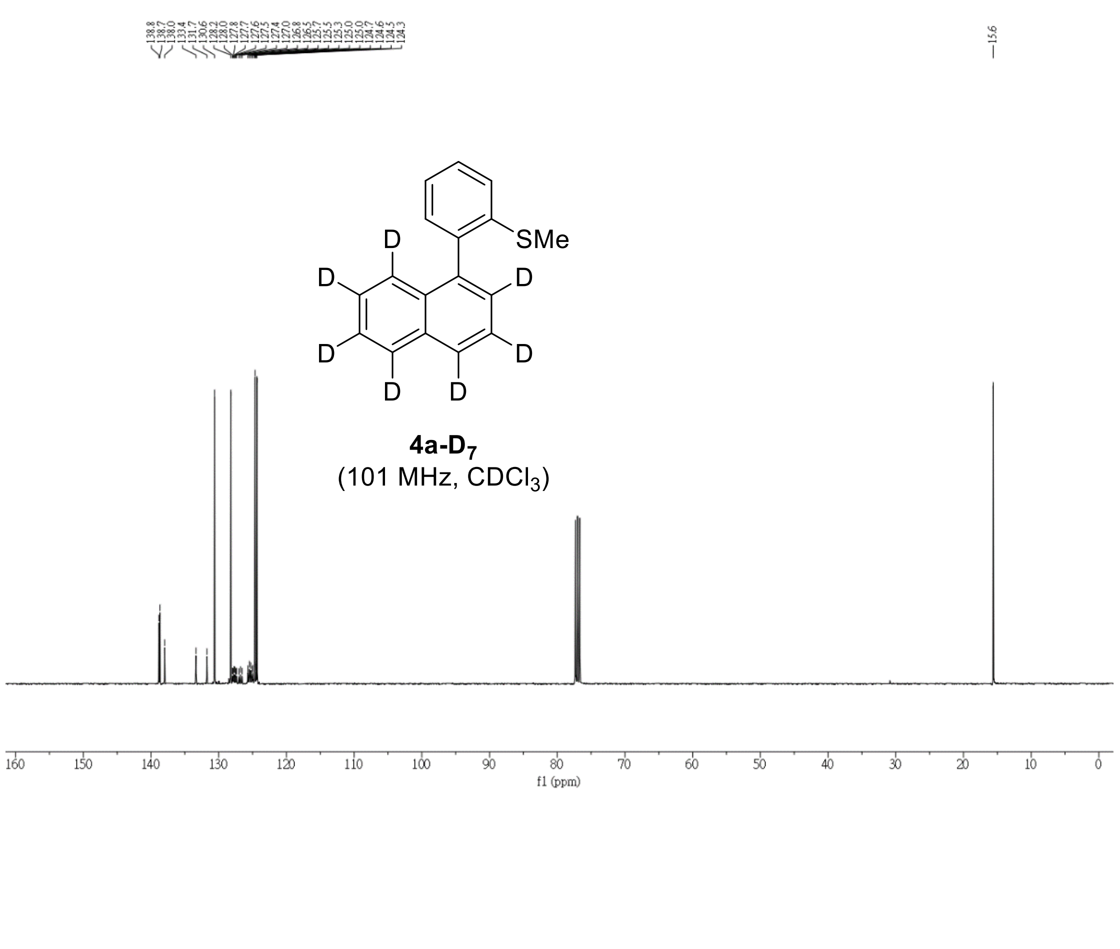

4a-D$_7$
(101 MHz, $CDCl_3$)

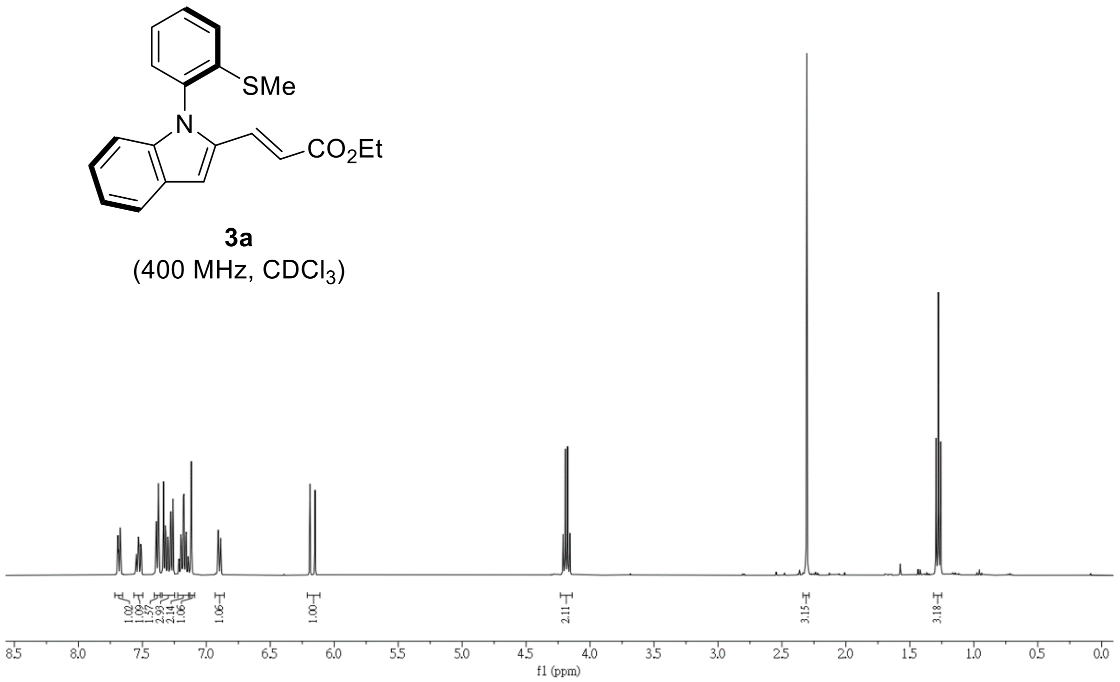

SMe
N
CO2Et
3a
(400 MHz, CDCl3)
1.06
1.00
2.11
3.15
3.18
f1 (ppm)

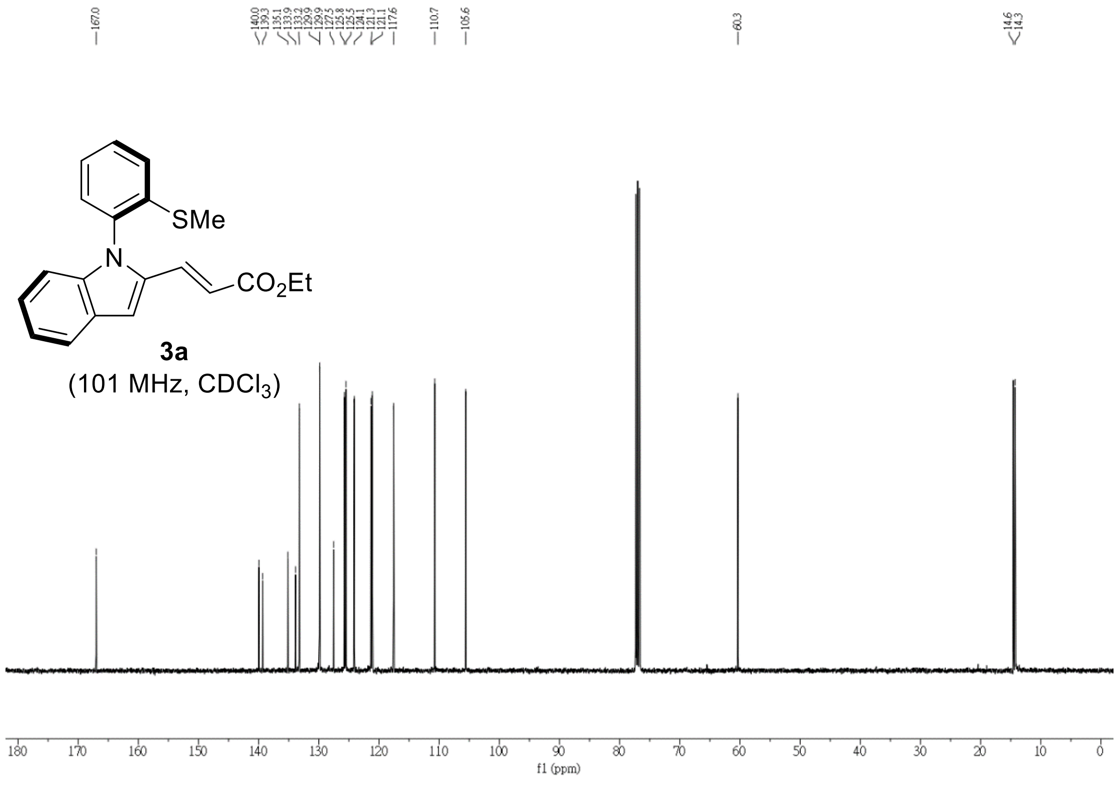

167.0
140.0
139.3
135.1
133.9
133.2
129.9
129.9
127.5
125.8
125.5
124.1
121.3
121.1
117.6
110.7
105.6
60.3
14.6
14.3
SMe
N
CO2Et
3a
(101 MHz, CDCl3)
f1 (ppm)

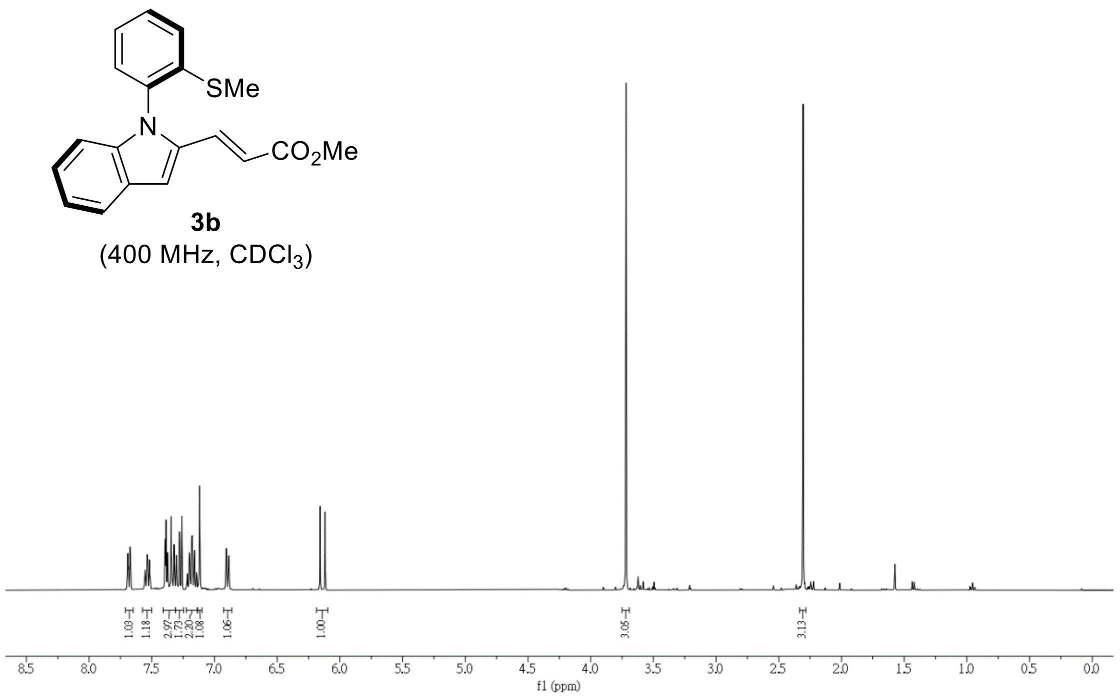
SMe
N
CO2Me
3b
(400 MHz, CDCl3)
1.03
1.18
2.97
1.73
2.20
1.08
1.06
1.00
3.05
3.13
8.5 8.0 7.5 7.0 6.5 6.0 5.5 5.0 4.5 4.0 3.5 3.0 2.5 2.0 1.5 1.0 0.5 0.0
f1 (ppm)

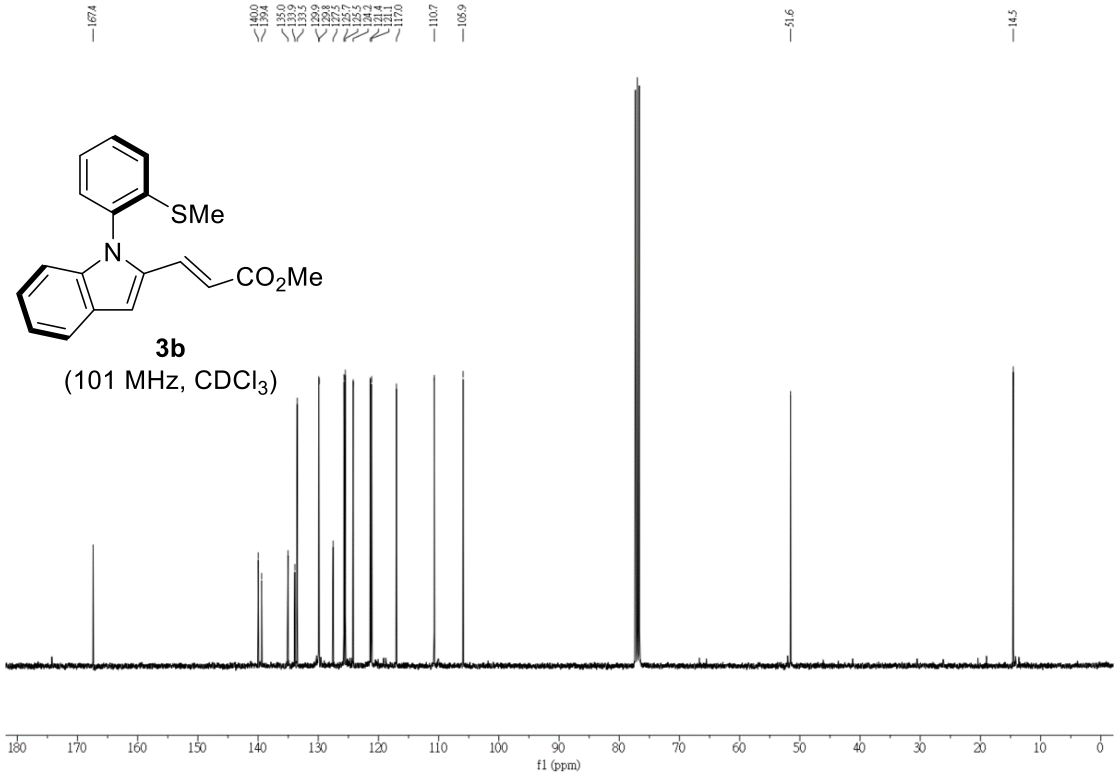
167.4
140.0
139.4
135.0
133.9
133.5
129.9
129.8
127.5
125.7
125.5
124.2
121.4
121.1
117.0
110.7
105.9
51.6
14.5
SMe
N
CO2Me
3b
(101 MHz, CDCl3)
180 170 160 150 140 130 120 110 100 90 80 70 60 50 40 30 20 10 0
f1 (ppm)

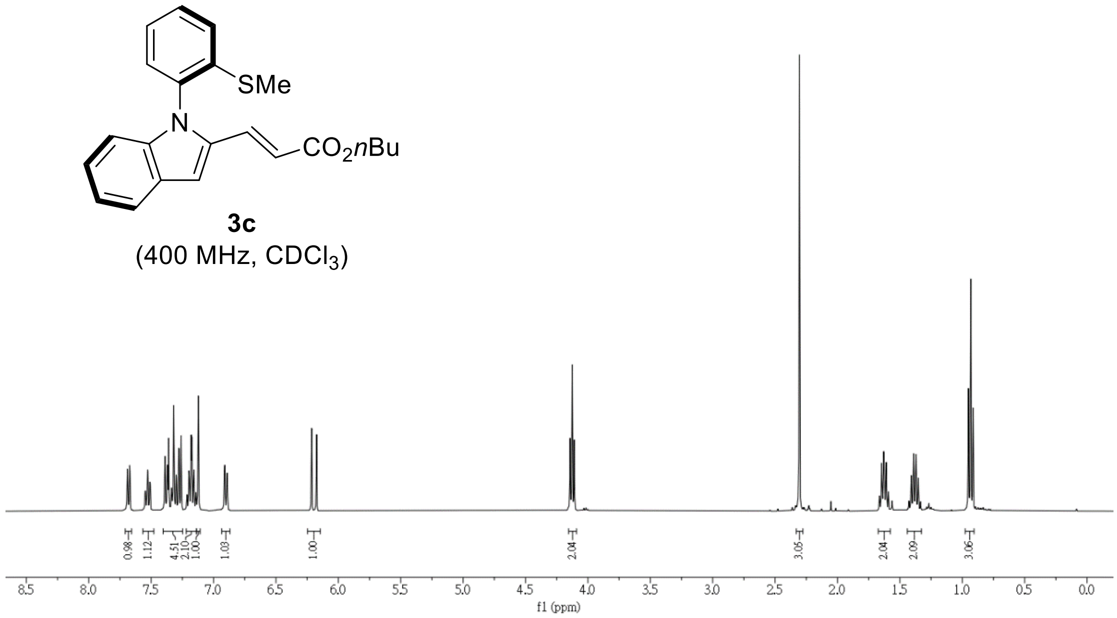
SMe
N
CO2nBu
3c
(400 MHz, CDCl3)
0.98
1.12
4.51
2.10
1.00
1.03
1.00
2.04
3.05
2.04
2.09
3.06
f1 (ppm)

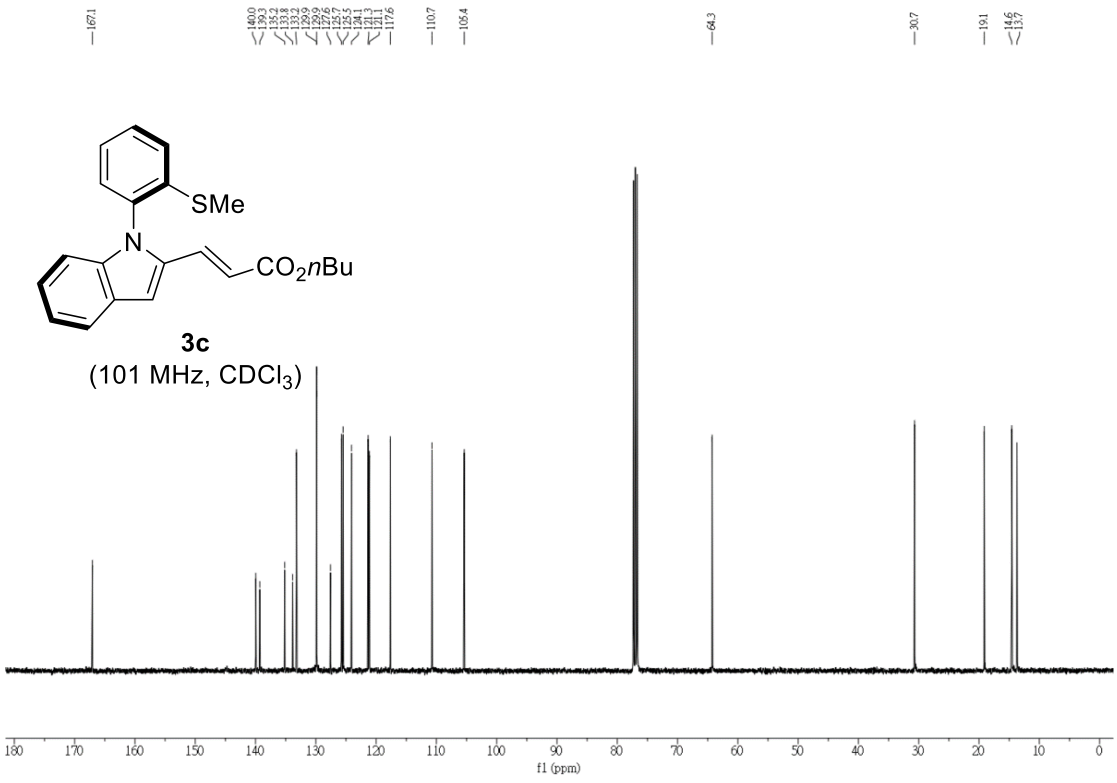
167.1
140.0
139.3
135.2
133.8
133.2
129.9
129.9
127.6
125.7
125.5
124.1
121.3
121.1
117.6
110.7
105.4
64.3
30.7
19.1
14.6
13.7
SMe
N
CO2nBu
3c
(101 MHz, CDCl3)
f1 (ppm)

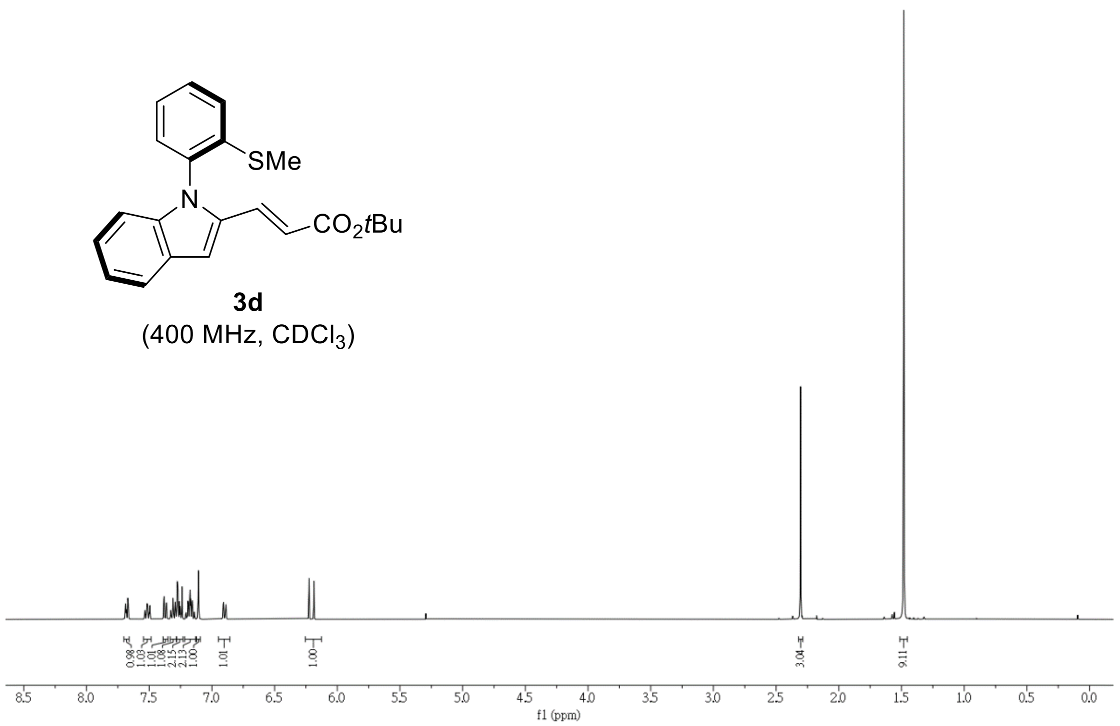

3d
(400 MHz, $CDCl_3$)

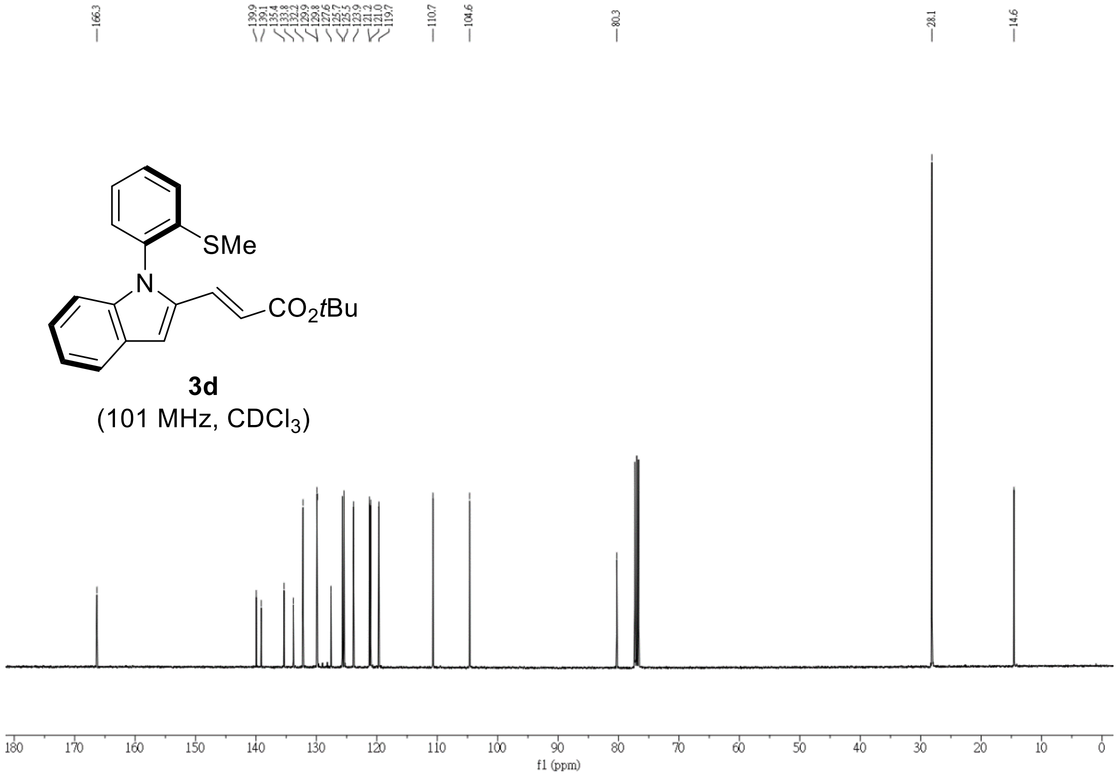

3d
(101 MHz, $CDCl_3$)

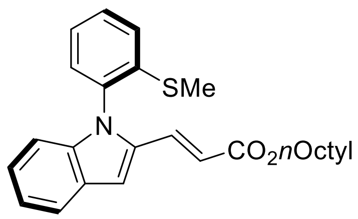

3e
(400 MHz, $CDCl_3$)

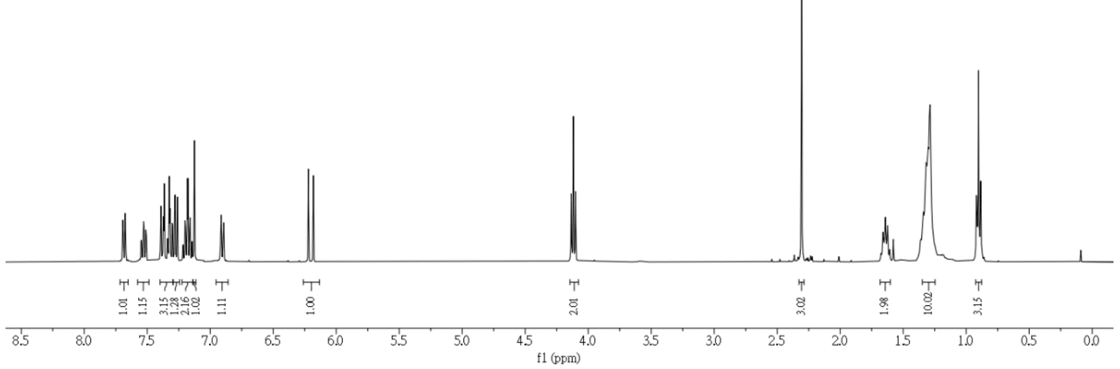

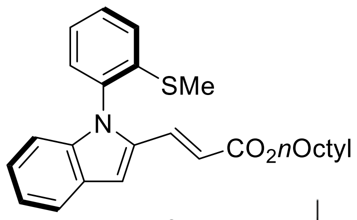

3e
(101 MHz, $CDCl_3$)

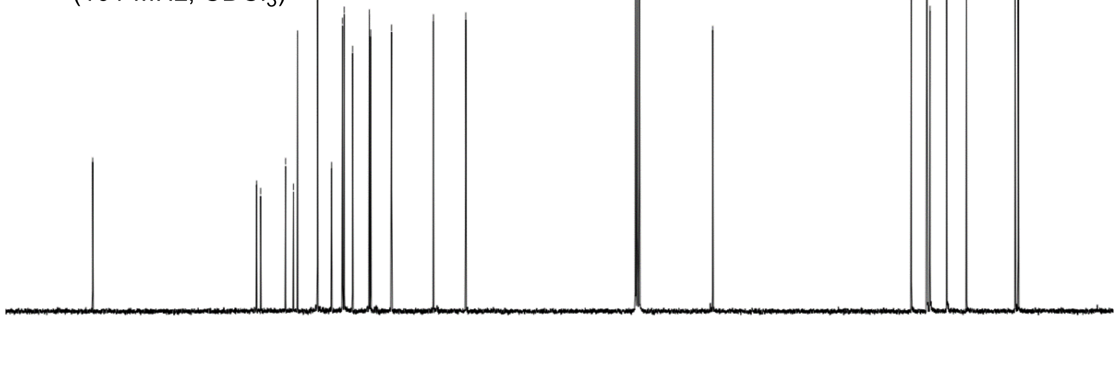

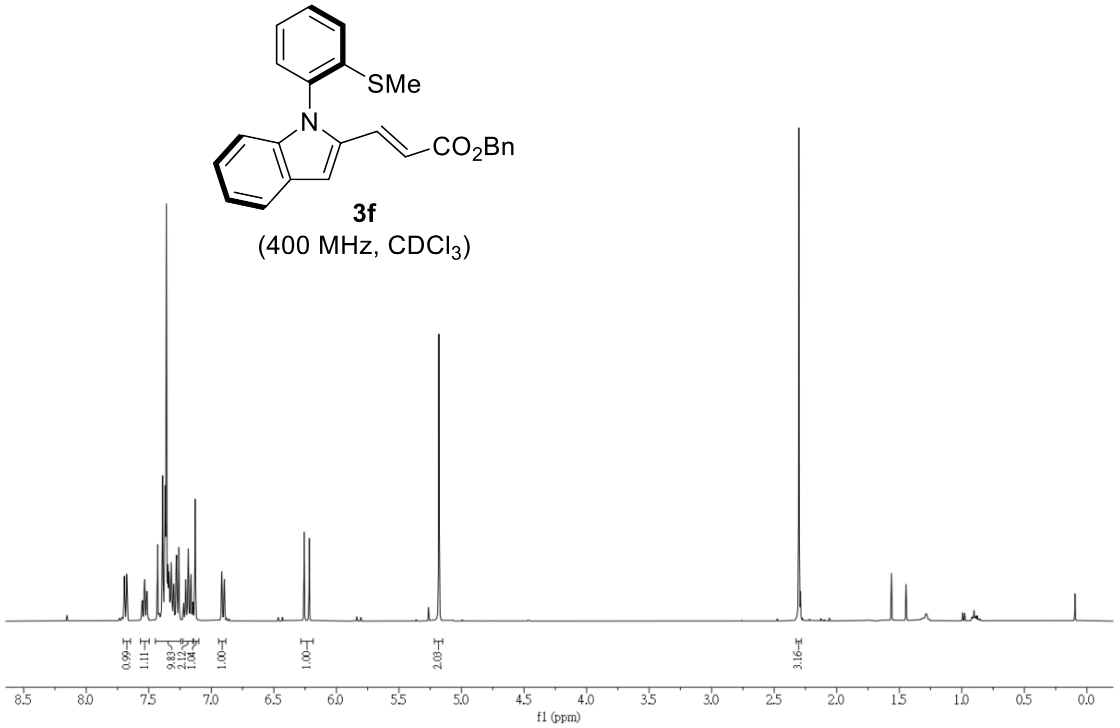
SMe
N
CO_2Bn
3f
(400 MHz, $CDCl_3$)
0.99
1.11
9.83
2.12
1.04
1.00
1.00
2.03
3.16
f1 (ppm)

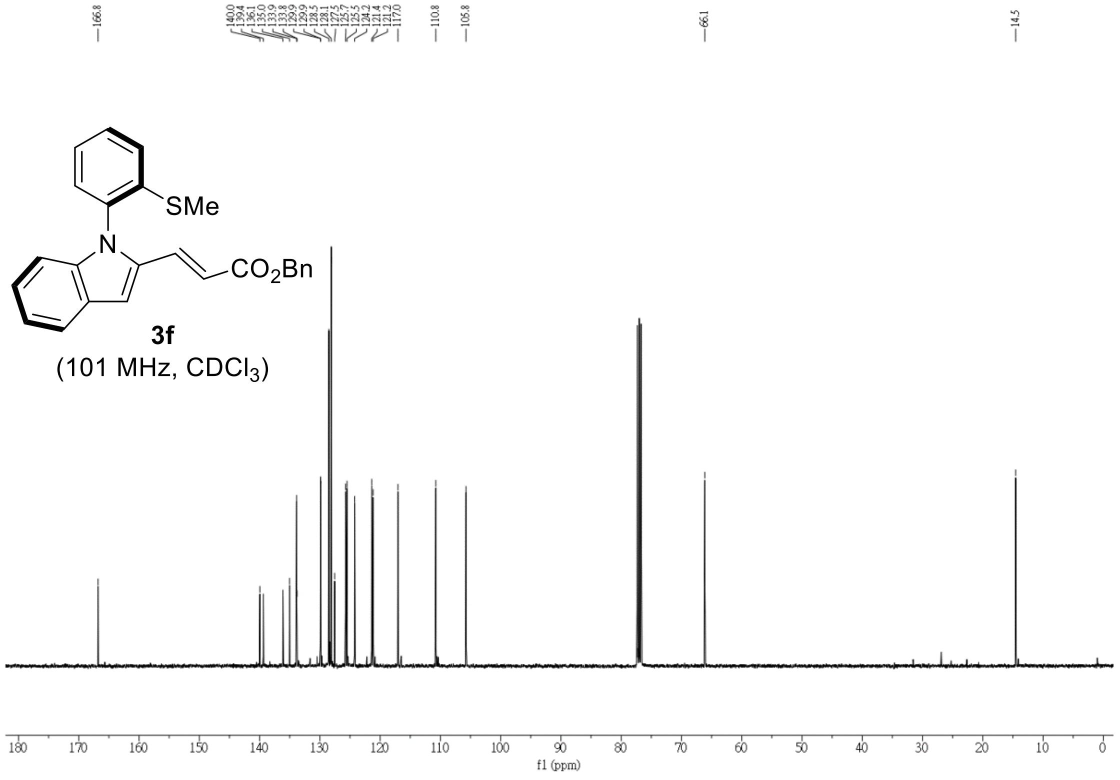
166.8
140.0
139.4
136.1
135.0
133.9
133.8
129.9
129.9
128.5
128.1
127.5
125.7
125.5
124.2
121.4
121.2
117.0
110.8
105.8
66.1
14.5
SMe
N
CO_2Bn
3f
(101 MHz, $CDCl_3$)
f1 (ppm)

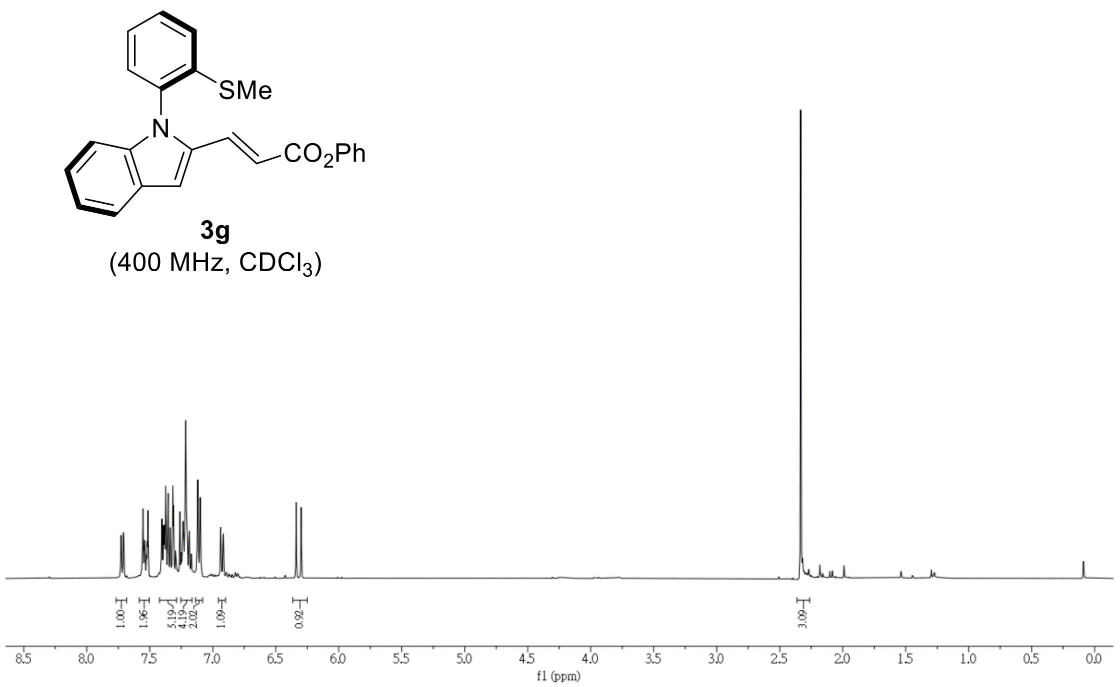
SMe
N
CO_2Ph
3g
(400 MHz, $CDCl_3$)
1.00
1.96
5.19
4.19
2.02
1.09
0.92
3.09
8.5 8.0 7.5 7.0 6.5 6.0 5.5 5.0 4.5 4.0 3.5 3.0 2.5 2.0 1.5 1.0 0.5 0.0
f1 (ppm)

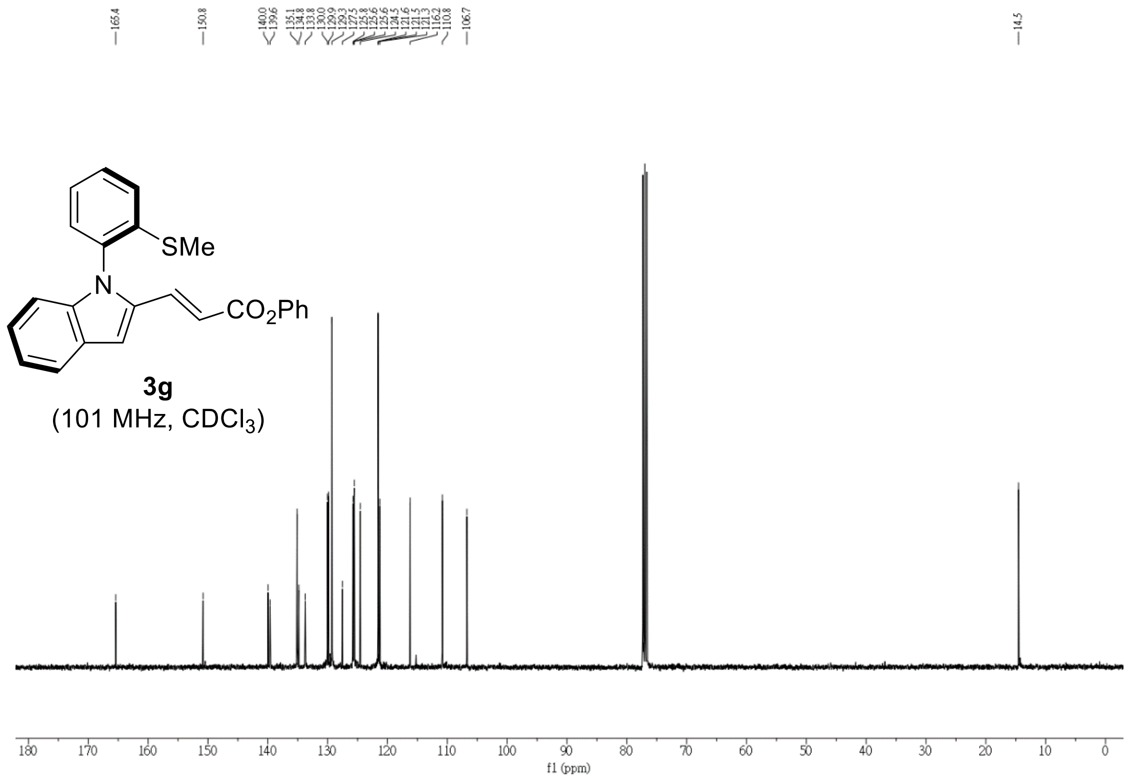
165.4
150.8
140.0
139.6
135.1
134.8
133.8
130.0
129.9
129.3
127.5
125.8
125.6
125.6
124.5
121.6
121.5
121.3
116.2
110.8
106.7
14.5
SMe
N
CO_2Ph
3g
(101 MHz, $CDCl_3$)
180 170 160 150 140 130 120 110 100 90 80 70 60 50 40 30 20 10 0
f1 (ppm)

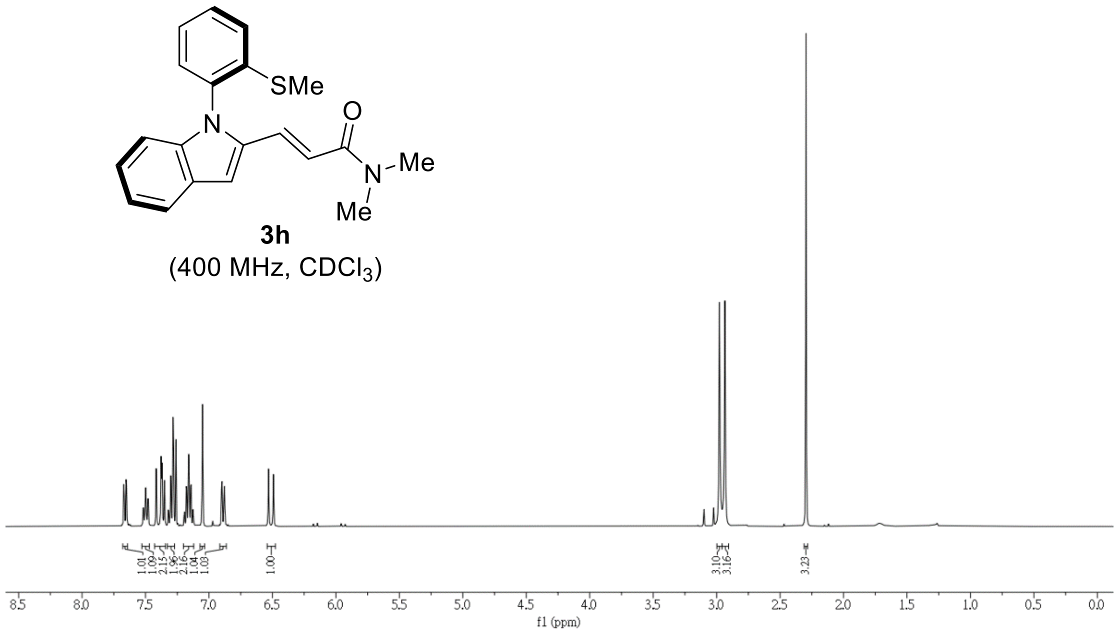
SMe
O
N
Me
Me
3h
(400 MHz, CDCl3)
f1 (ppm)

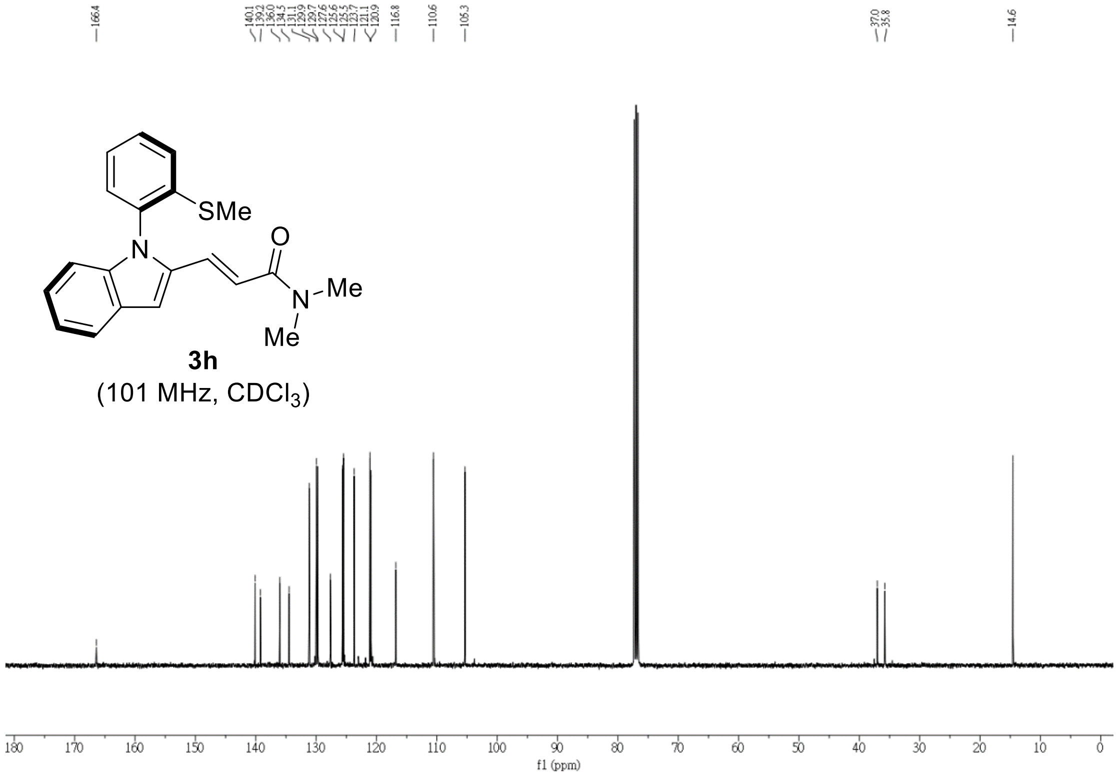
SMe
O
N
Me
Me
3h
(101 MHz, CDCl3)
f1 (ppm)

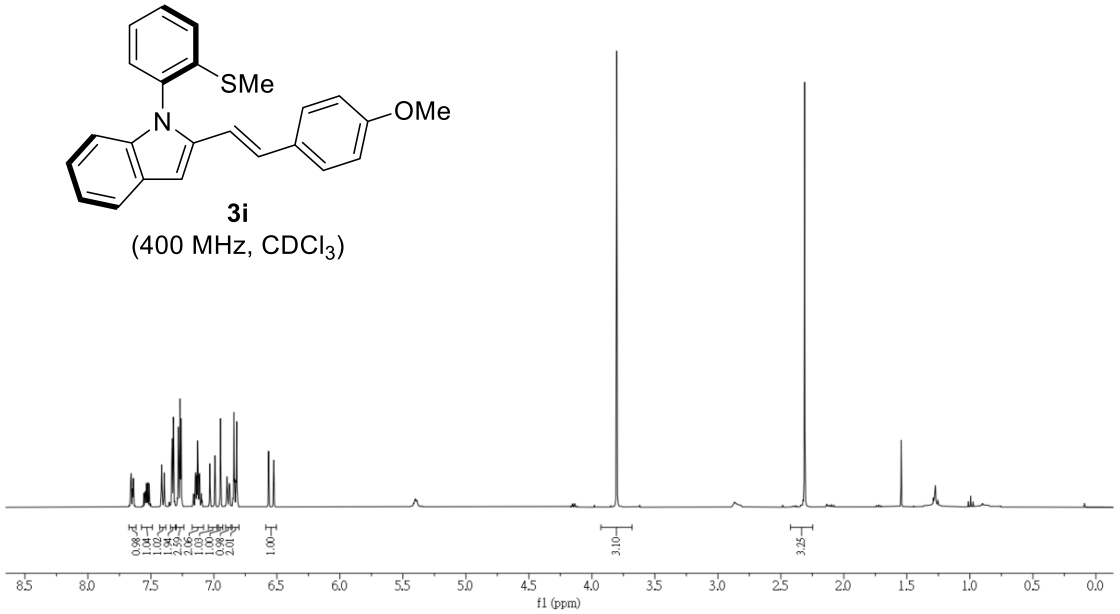

3i
(400 MHz, $CDCl_3$)

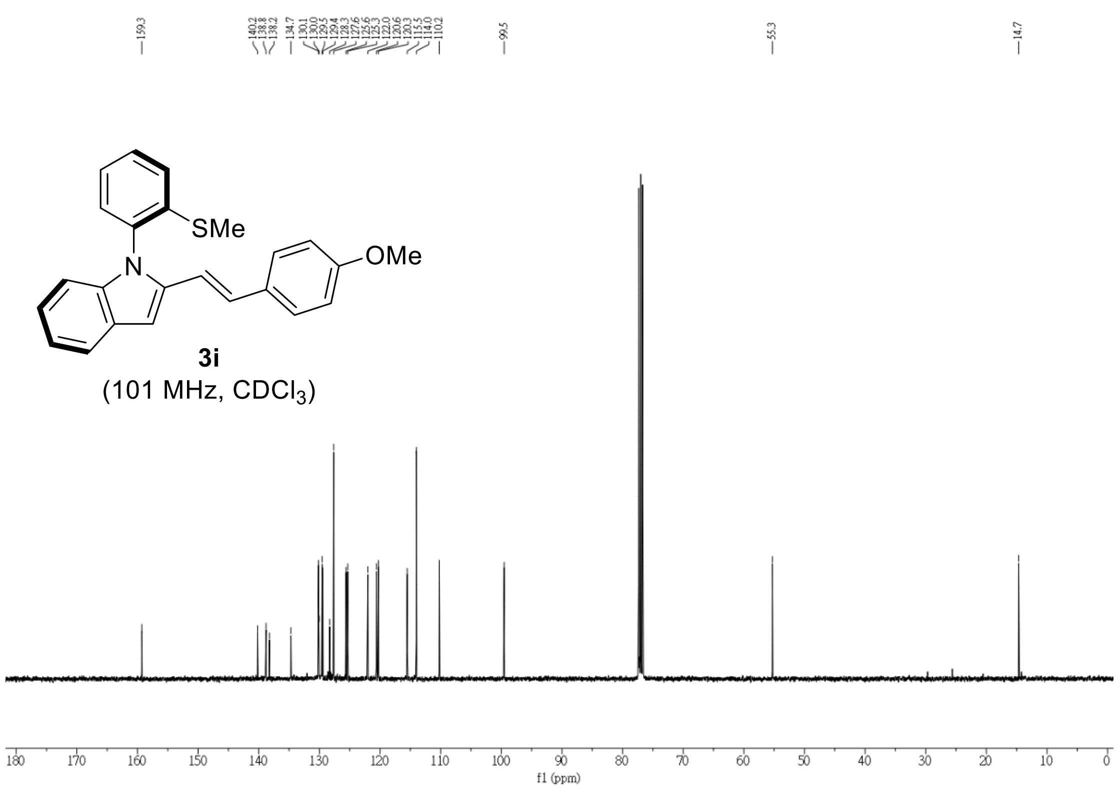

3i
(101 MHz, $CDCl_3$)

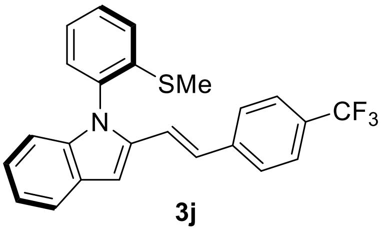

3j
(400 MHz, $CDCl_3$)

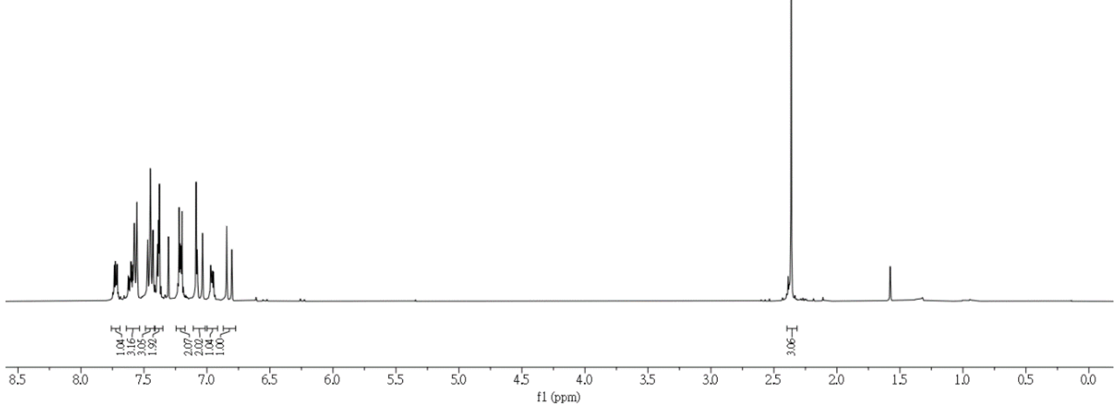

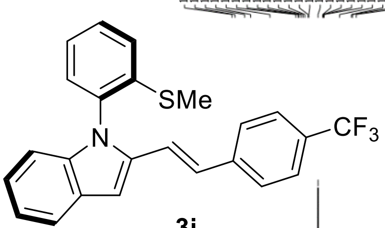

3j
(101MHz, $CDCl_3$)

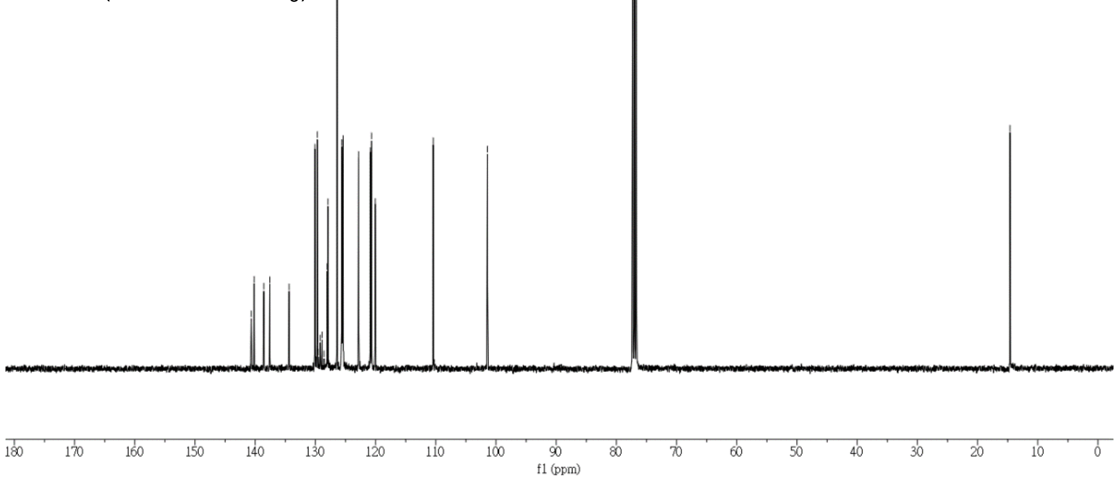

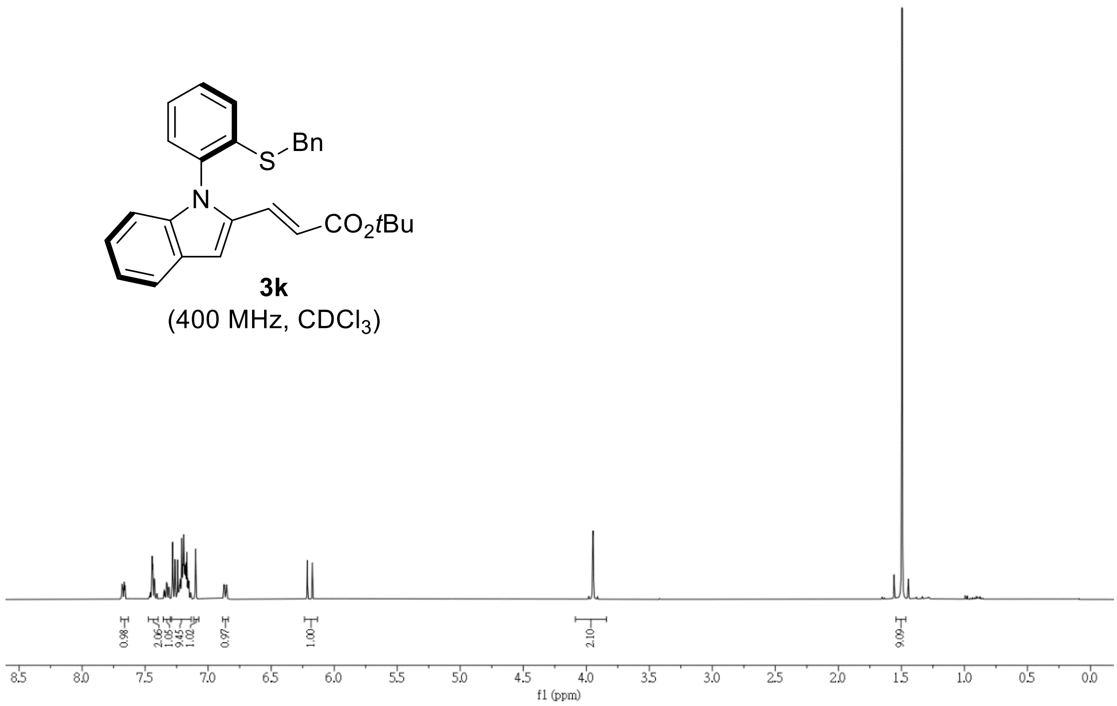
S
Bn
N
CO2tBu
3k
(400 MHz, CDCl3)
f1 (ppm)

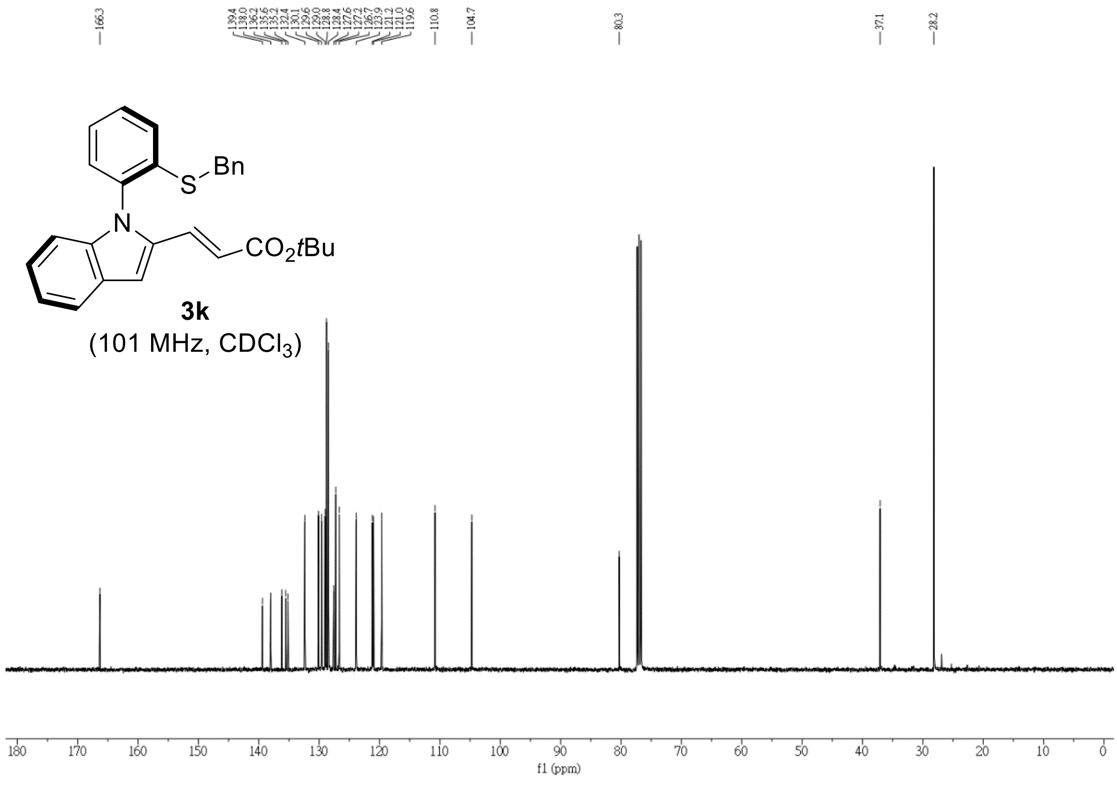
166.3
139.4
138.0
136.2
135.6
135.2
132.4
130.1
129.6
129.0
128.8
128.4
127.6
127.2
126.7
123.9
121.2
121.0
119.6
110.8
104.7
80.3
37.1
28.2
S
Bn
N
CO2tBu
3k
(101 MHz, CDCl3)
f1 (ppm)

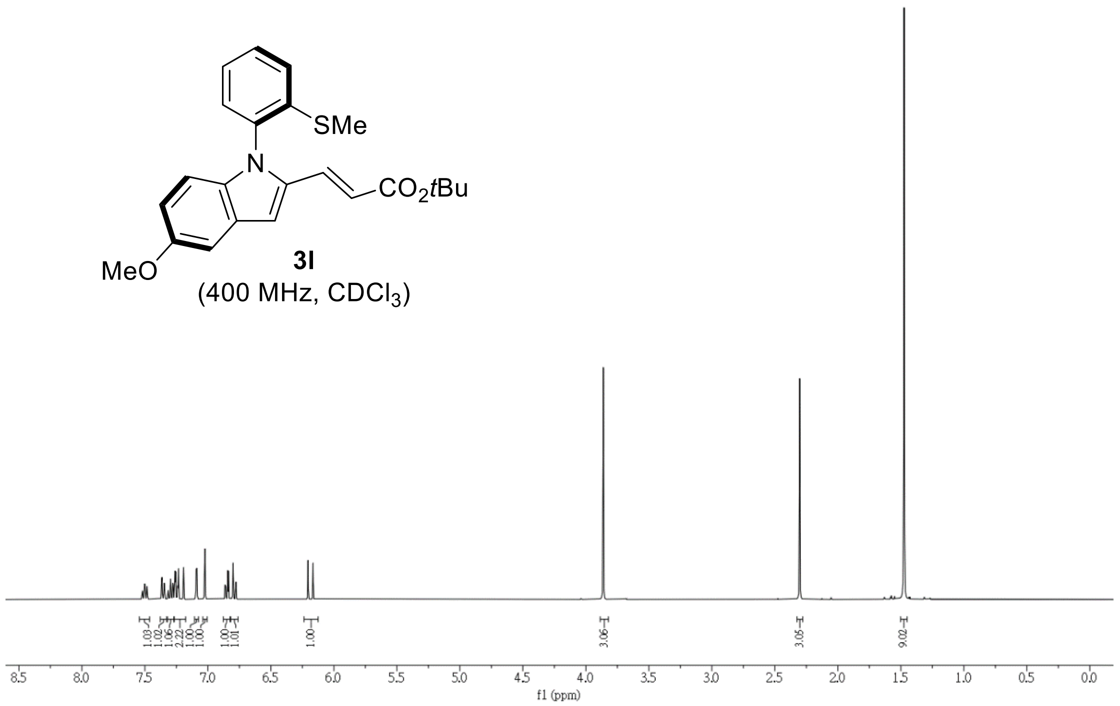
SMe
N
CO2tBu
MeO
3l
(400 MHz, CDCl3)
f1 (ppm)

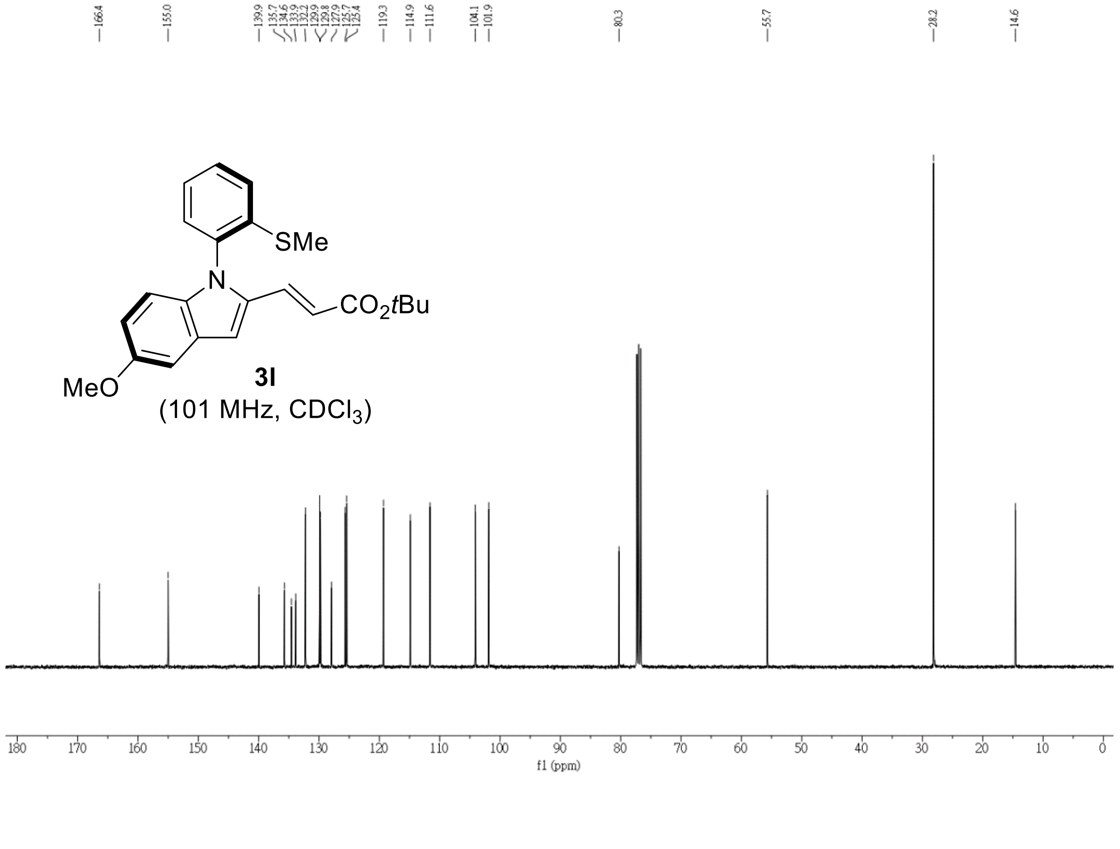
SMe
N
CO2tBu
MeO
3l
(101 MHz, CDCl3)
f1 (ppm)

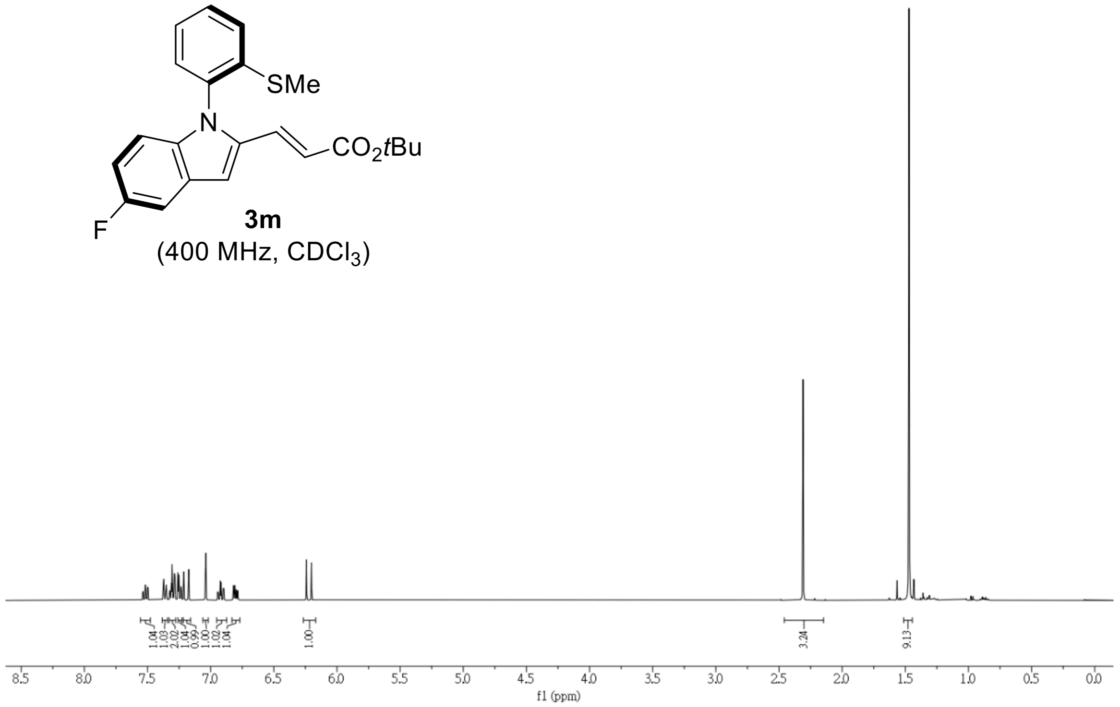
SMe
N
CO_2tBu
F
3m
(400 MHz, $CDCl_3$)
f1 (ppm)

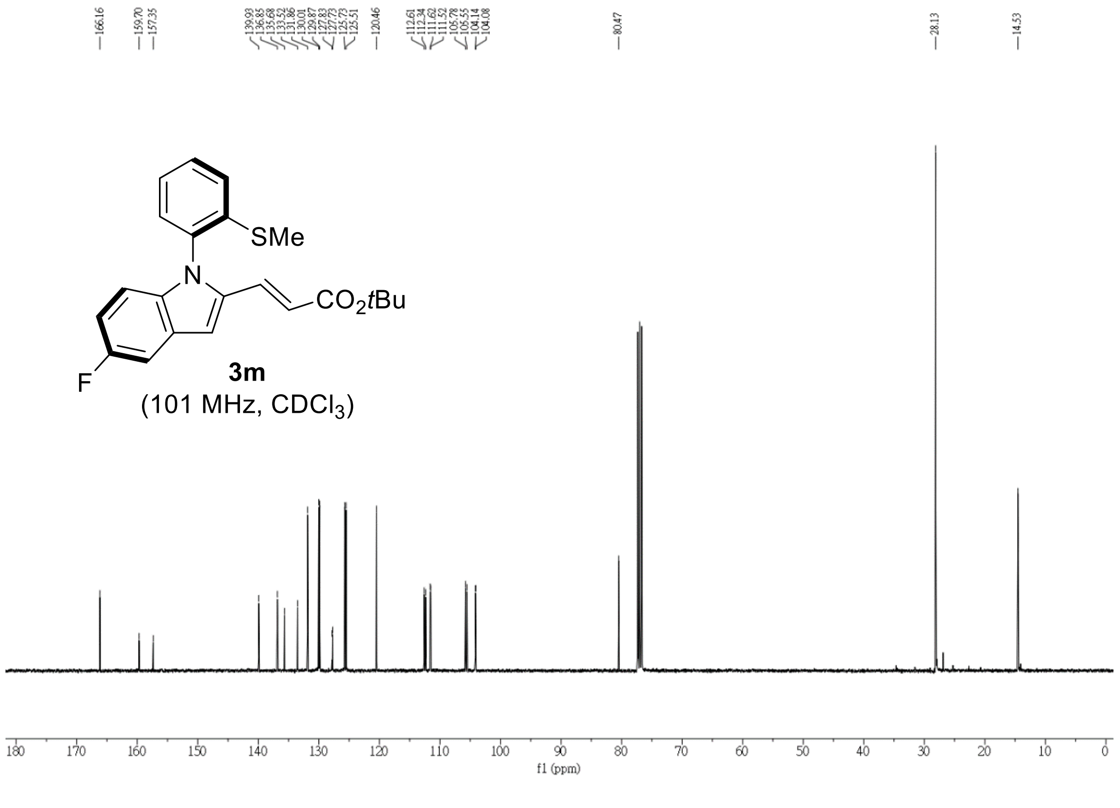
SMe
N
CO_2tBu
F
3m
(101 MHz, $CDCl_3$)
f1 (ppm)

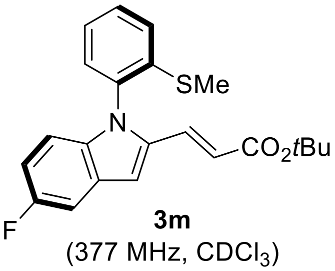

3m
(377 MHz, $CDCl_3$)

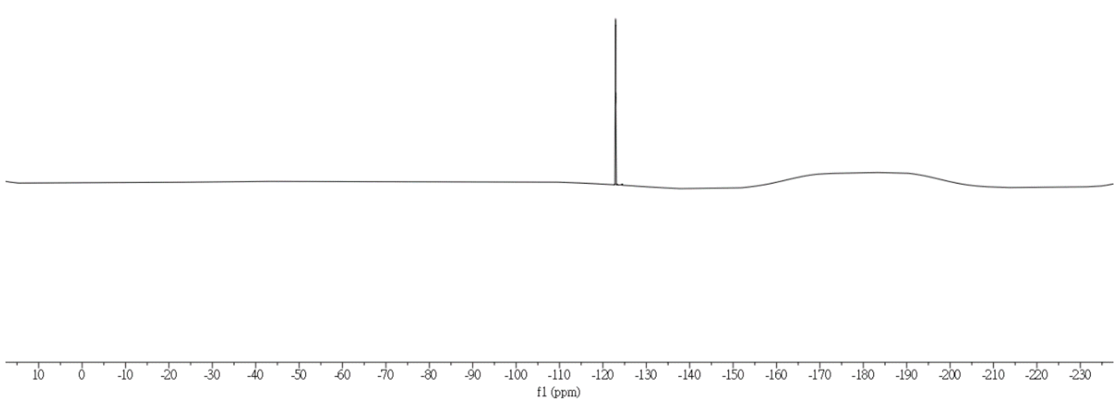

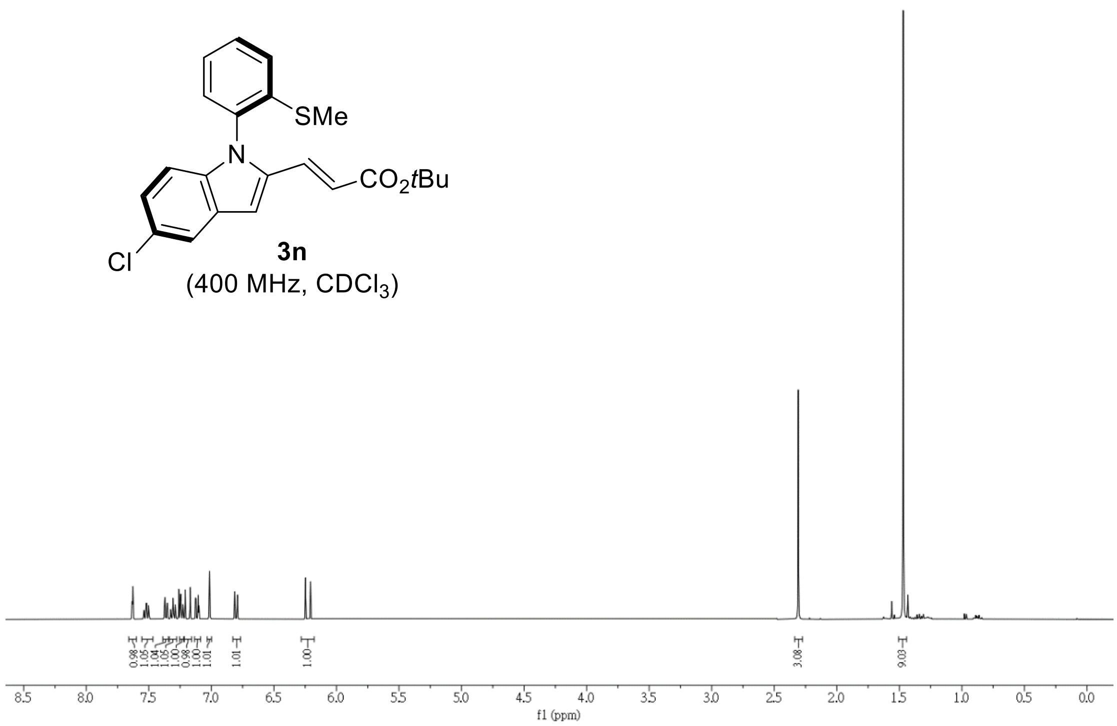

3n
(400 MHz, $CDCl_3$)

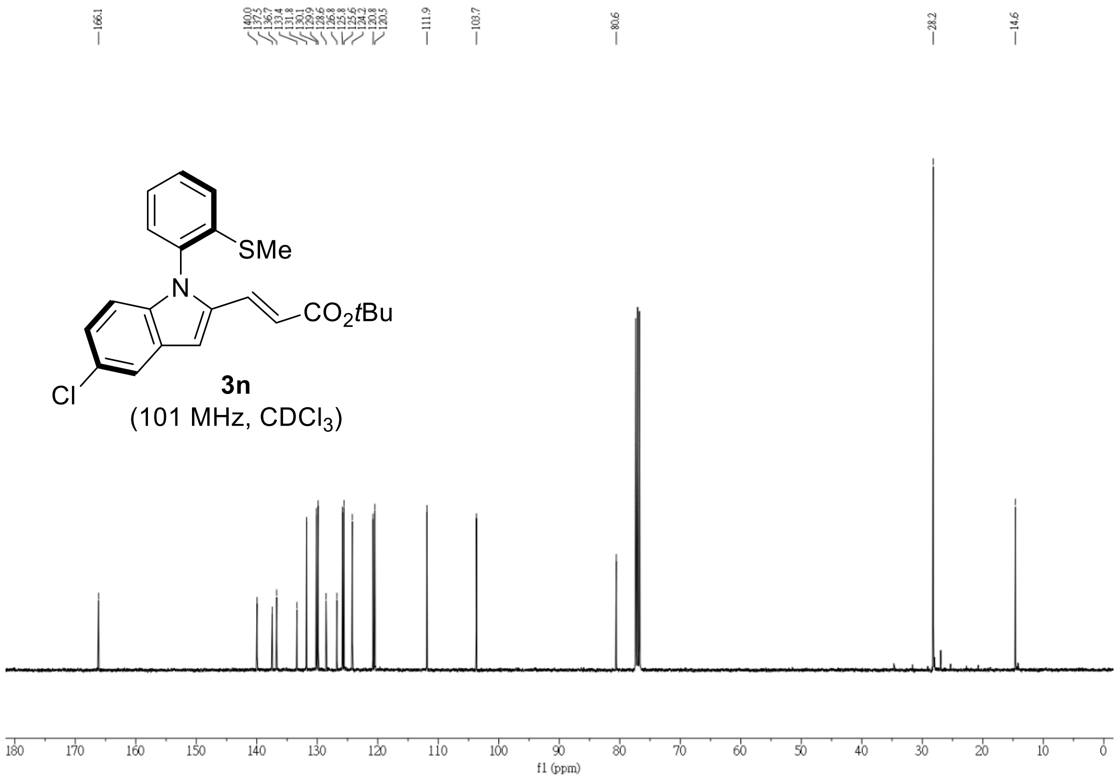

3n
(101 MHz, $CDCl_3$)

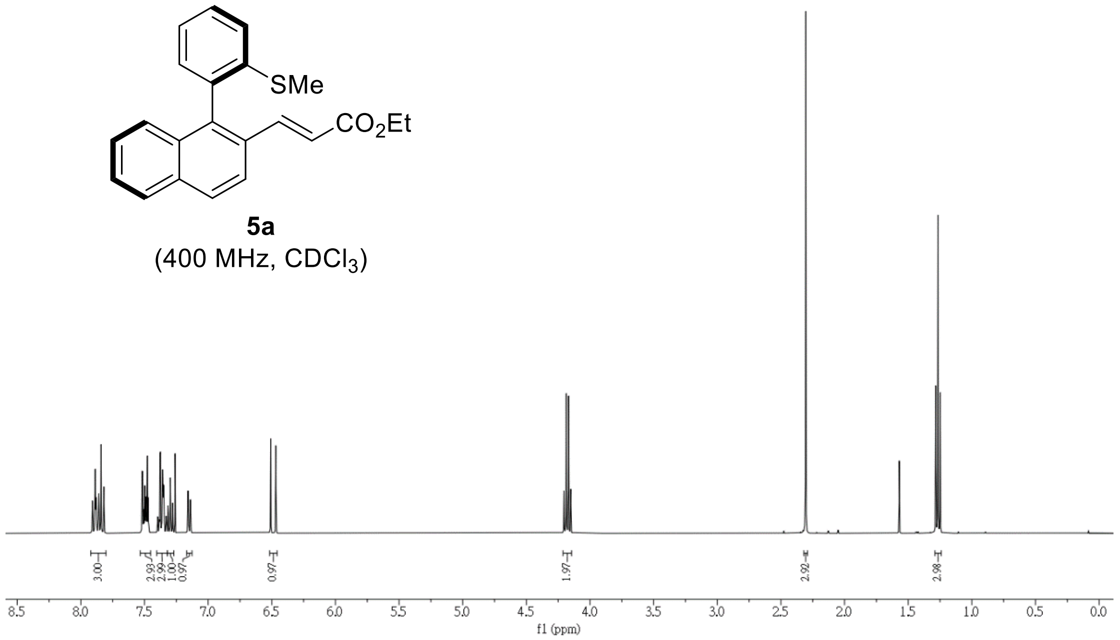

5a
(400 MHz, $CDCl_3$)

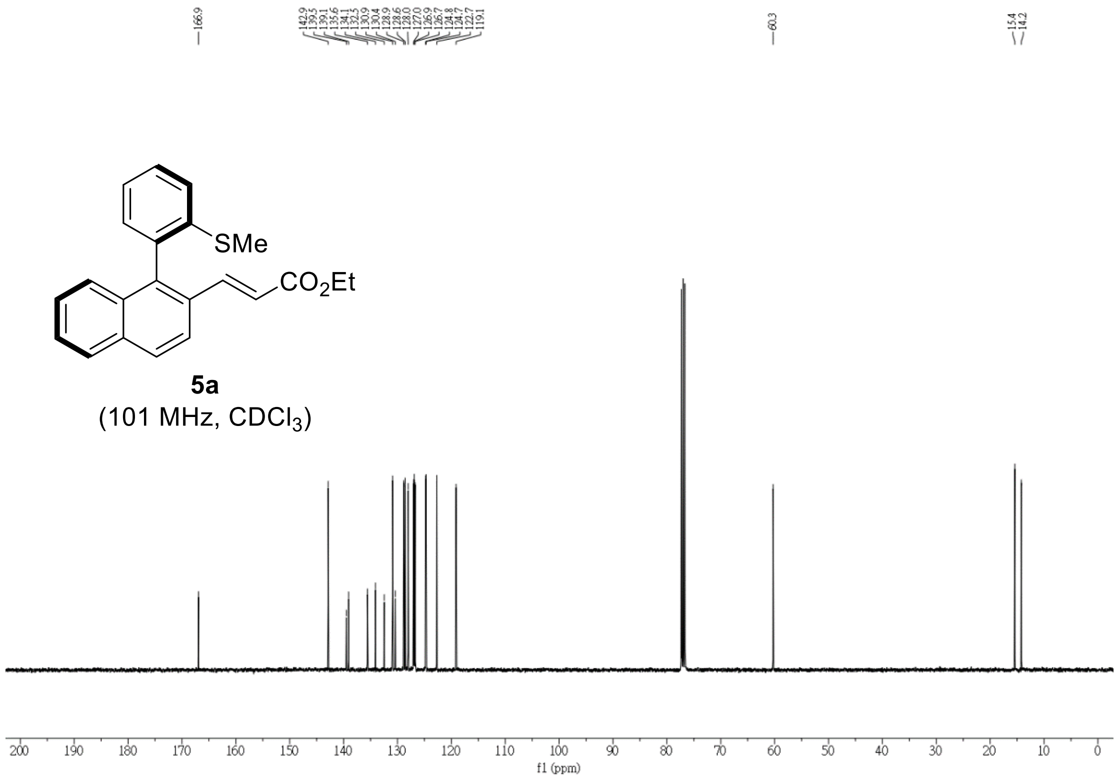

5a
(101 MHz, $CDCl_3$)

5b
(400 MHz, $CDCl_3$)

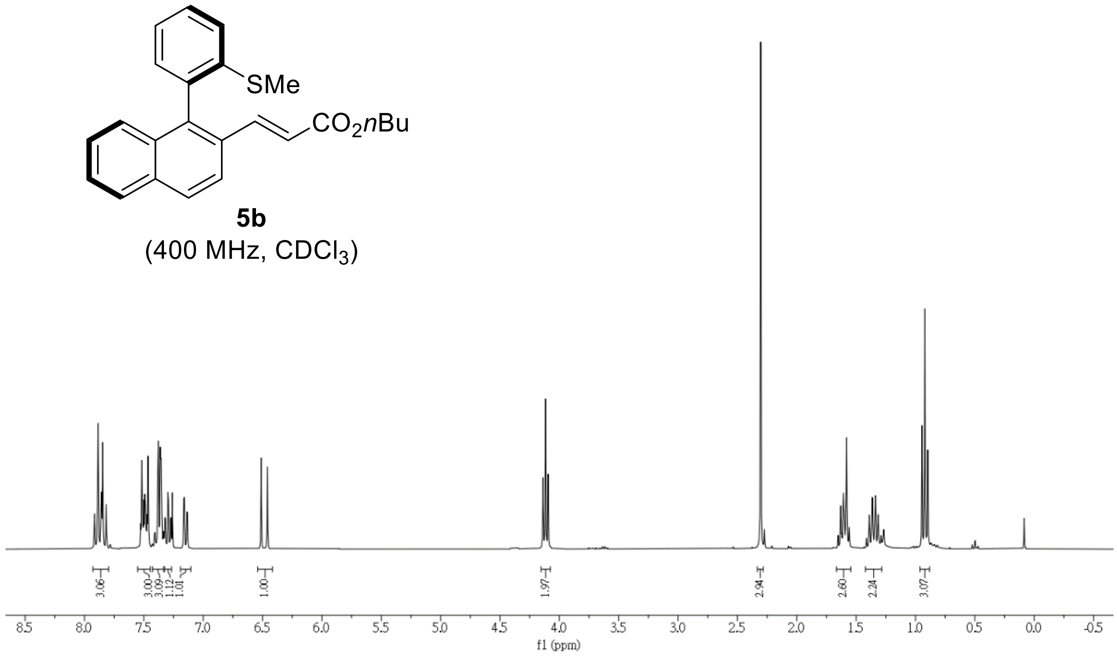

5b
(101 MHz, $CDCl_3$)

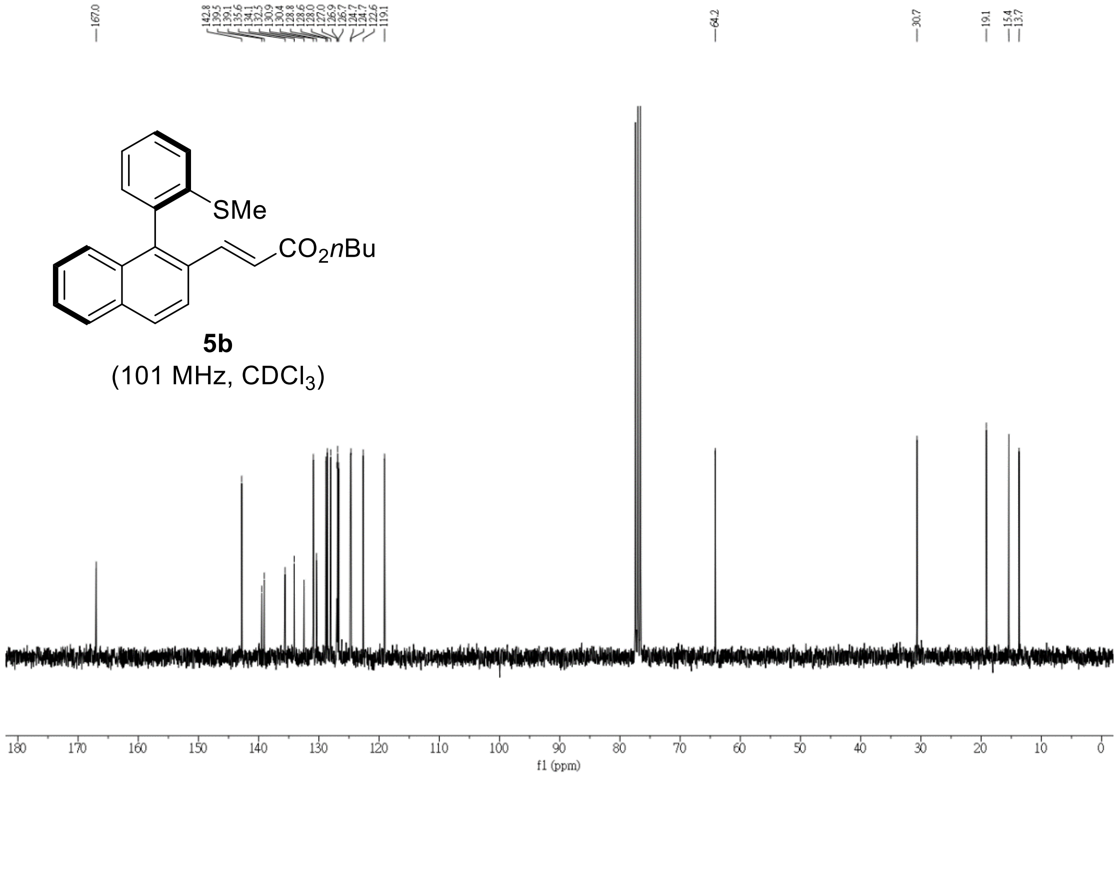

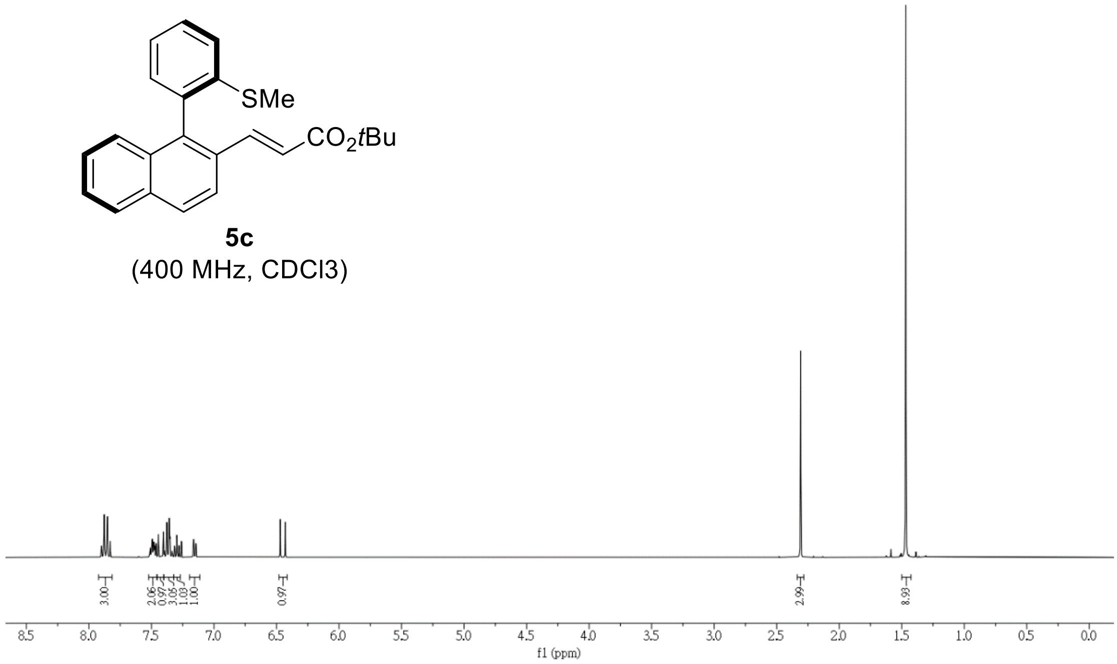
SMe
CO2tBu
5c
(400 MHz, CDCl3)
3.00
2.06
0.97
3.05
1.03
1.00
0.97
2.99
8.93
8.5 8.0 7.5 7.0 6.5 6.0 5.5 5.0 4.5 4.0 3.5 3.0 2.5 2.0 1.5 1.0 0.5 0.0
f1 (ppm)

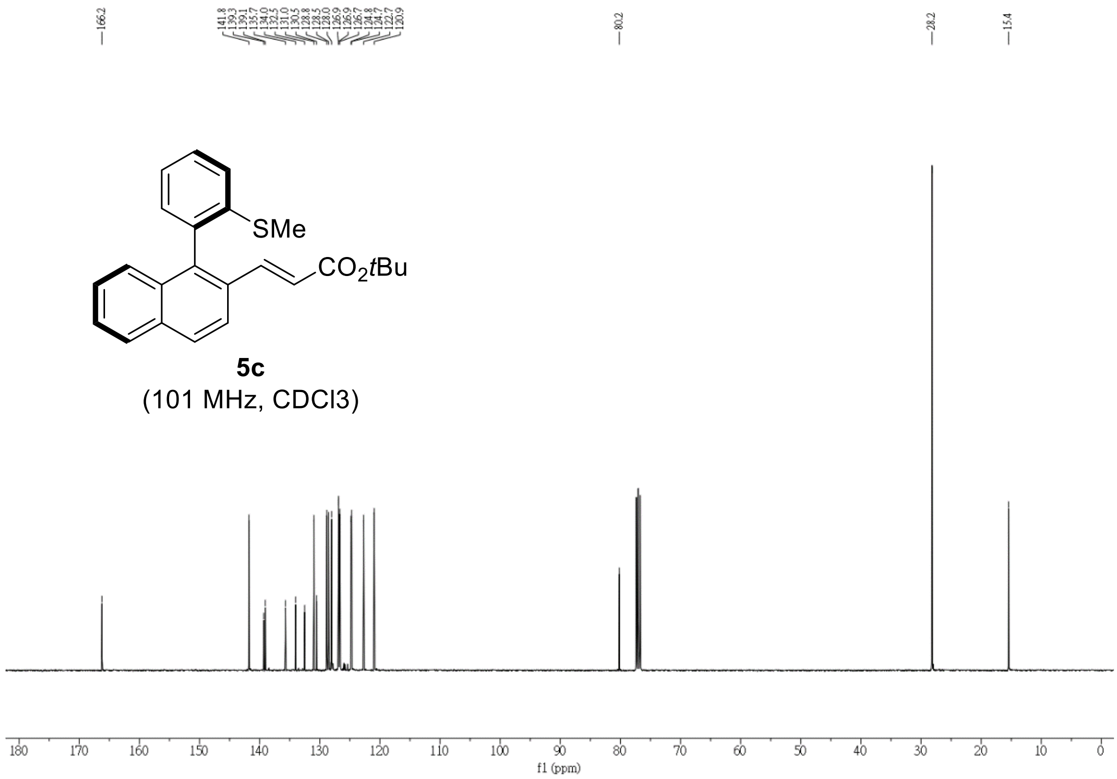
166.2
141.8
139.3
139.1
135.7
134.0
132.5
131.0
130.5
128.8
128.5
128.0
126.9
126.9
126.7
124.8
124.7
122.7
120.9
80.2
28.2
15.4
SMe
CO2tBu
5c
(101 MHz, CDCl3)
180 170 160 150 140 130 120 110 100 90 80 70 60 50 40 30 20 10 0
f1 (ppm)

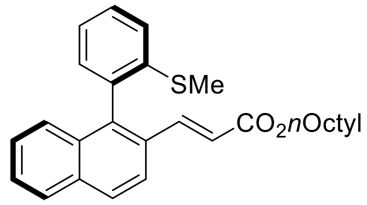

5d
(400 MHz, $CDCl_3$)

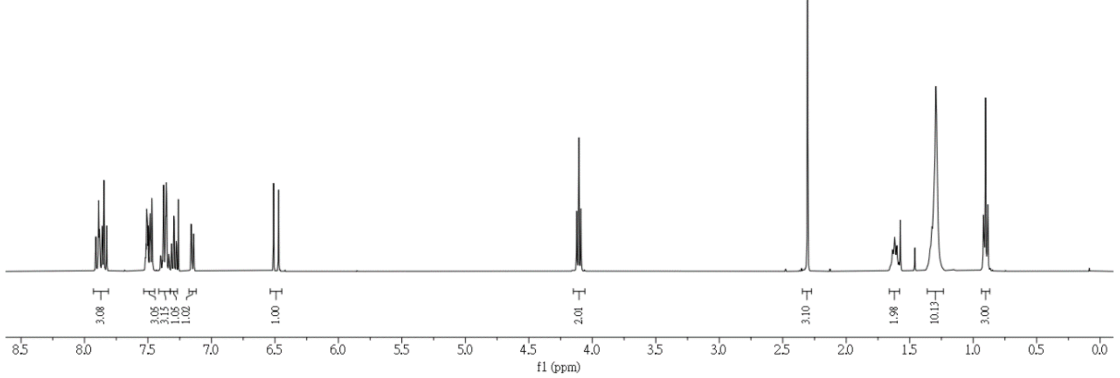

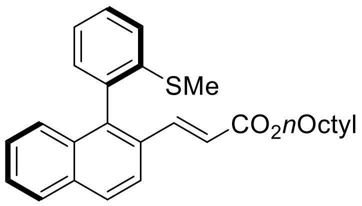

5d
(400 MHz, $CDCl_3$)

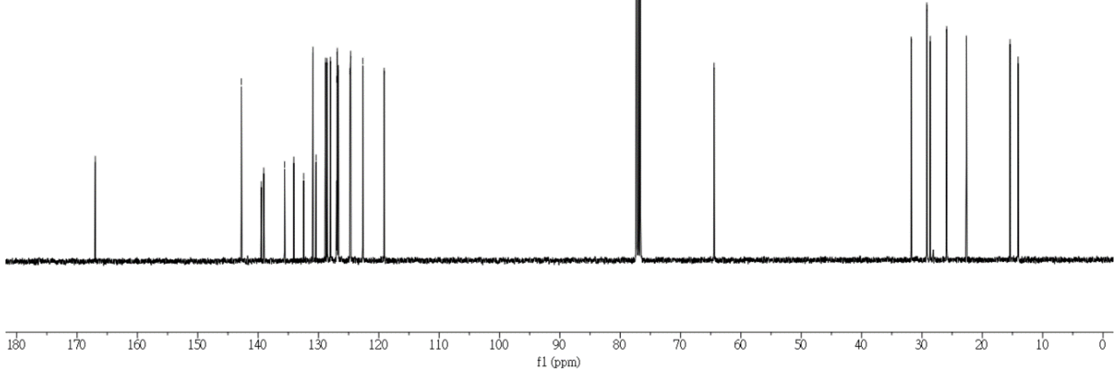

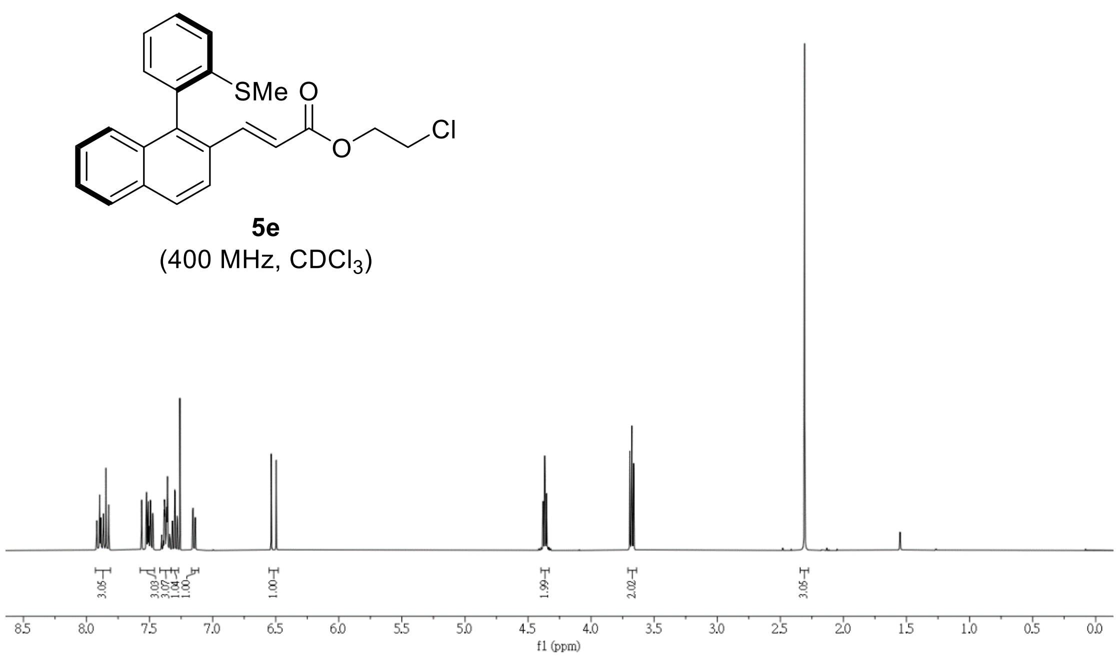
SMe
O
O
Cl
5e
(400 MHz, CDCl$_3$)
3.05
3.03
3.07
1.04
1.00
1.00
1.99
2.02
3.05
8.5 8.0 7.5 7.0 6.5 6.0 5.5 5.0 4.5 4.0 3.5 3.0 2.5 2.0 1.5 1.0 0.5 0.0
f1 (ppm)

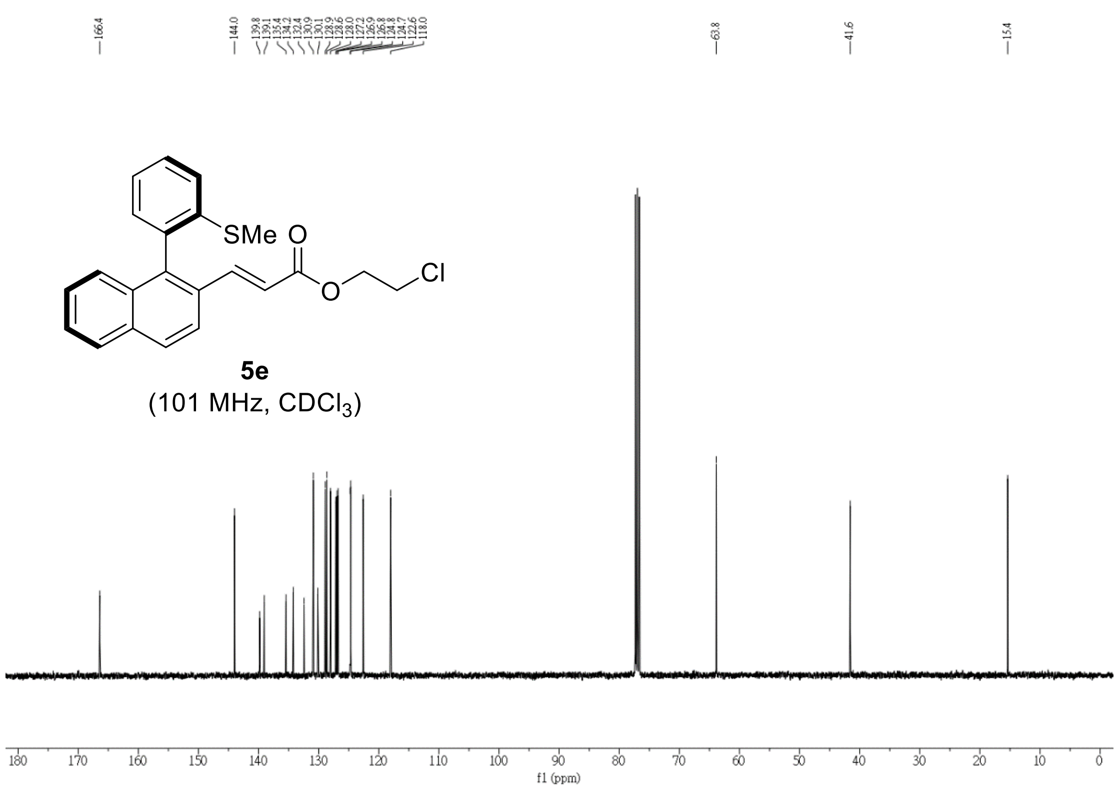
166.4
144.0
139.8
139.1
135.4
134.2
132.4
130.9
130.1
128.9
128.6
128.0
127.2
126.9
126.8
124.8
124.7
122.6
118.0
63.8
41.6
15.4
SMe
O
O
Cl
5e
(101 MHz, CDCl$_3$)
180 170 160 150 140 130 120 110 100 90 80 70 60 50 40 30 20 10 0
f1 (ppm)

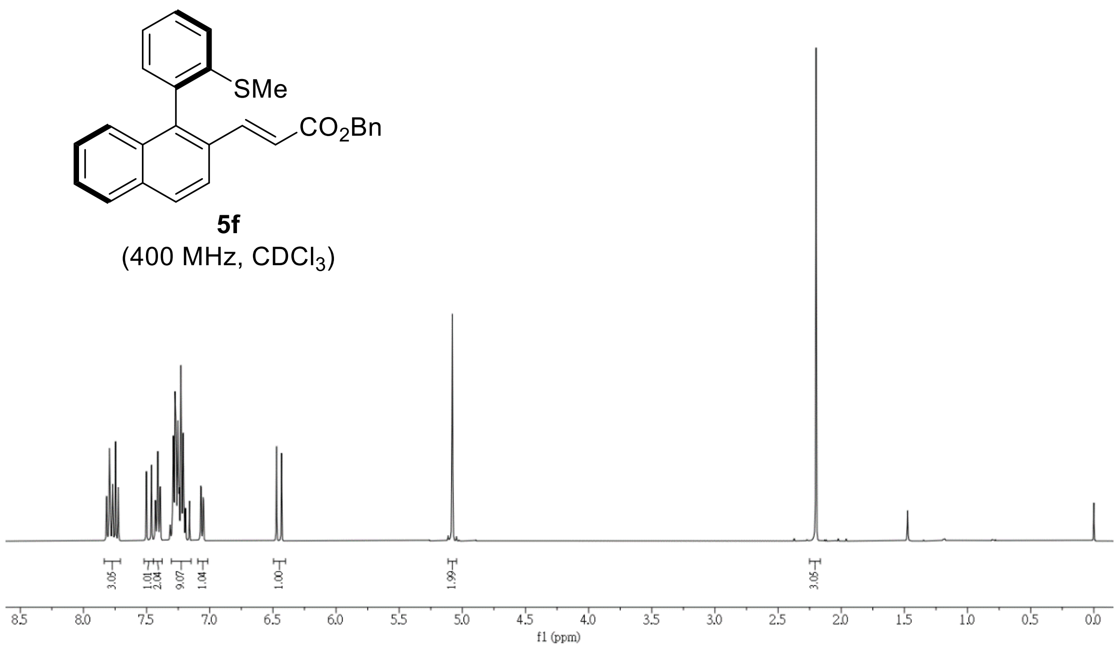
SMe
CO2Bn
5f
(400 MHz, CDCl3)
3.05
1.01
2.04
9.07
1.04
1.00
1.99
3.05
8.5 8.0 7.5 7.0 6.5 6.0 5.5 5.0 4.5 4.0 3.5 3.0 2.5 2.0 1.5 1.0 0.5 0.0
f1 (ppm)

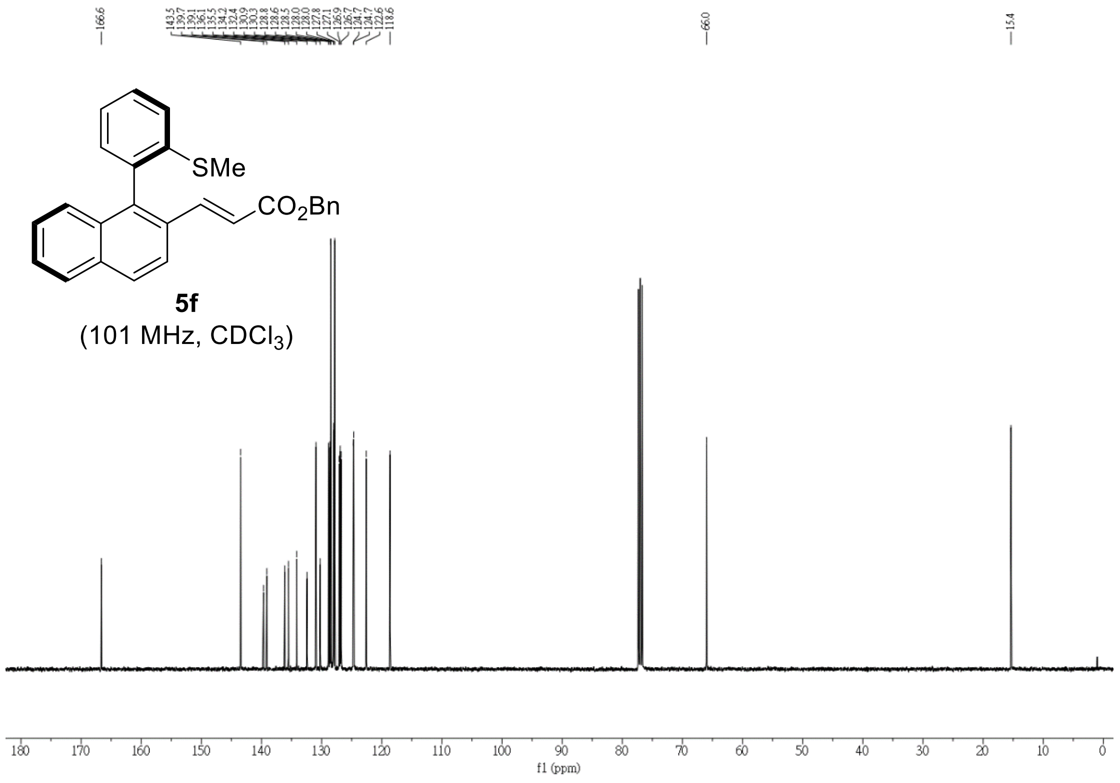
166.6
143.5
139.7
139.1
136.1
135.5
134.2
132.4
130.9
130.3
128.8
128.6
128.5
128.0
128.0
127.8
127.1
126.9
126.7
124.7
124.7
122.6
118.6
66.0
15.4
SMe
CO2Bn
5f
(101 MHz, CDCl3)
180 170 160 150 140 130 120 110 100 90 80 70 60 50 40 30 20 10 0
f1 (ppm)

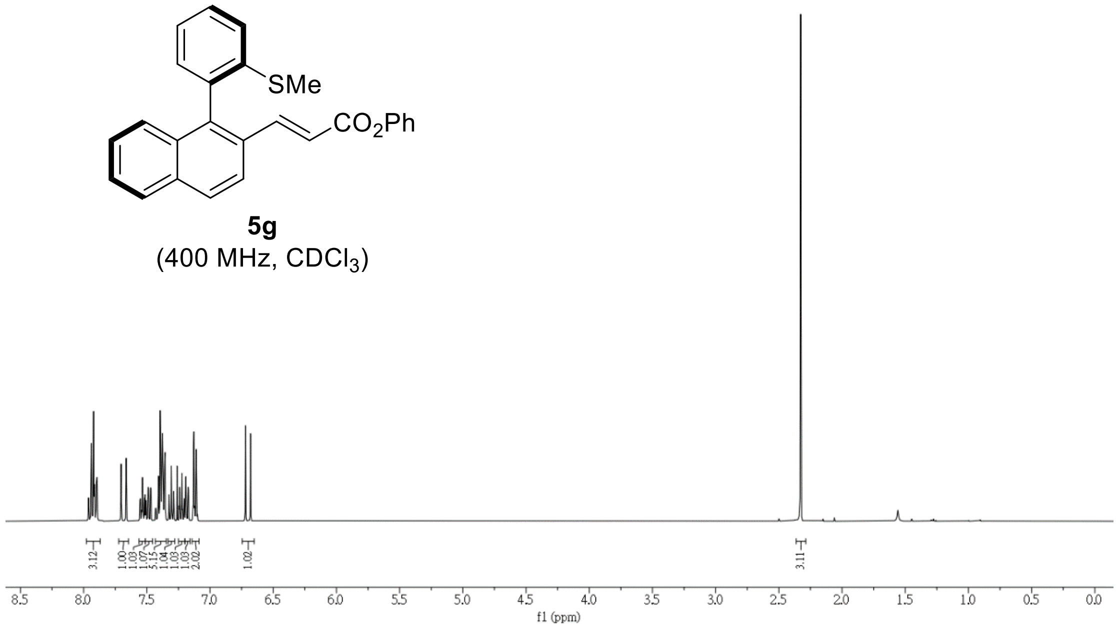
SMe
CO_2Ph
5g
(400 MHz, $CDCl_3$)
f1 (ppm)

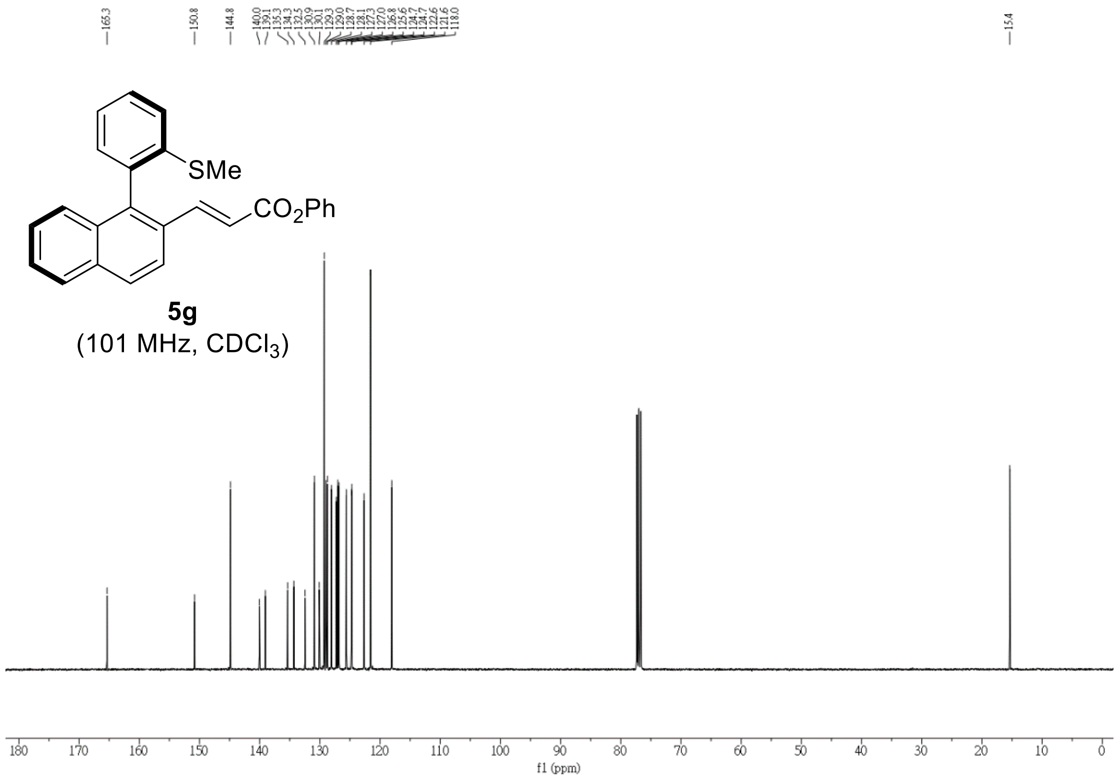
SMe
CO_2Ph
5g
(101 MHz, $CDCl_3$)
f1 (ppm)

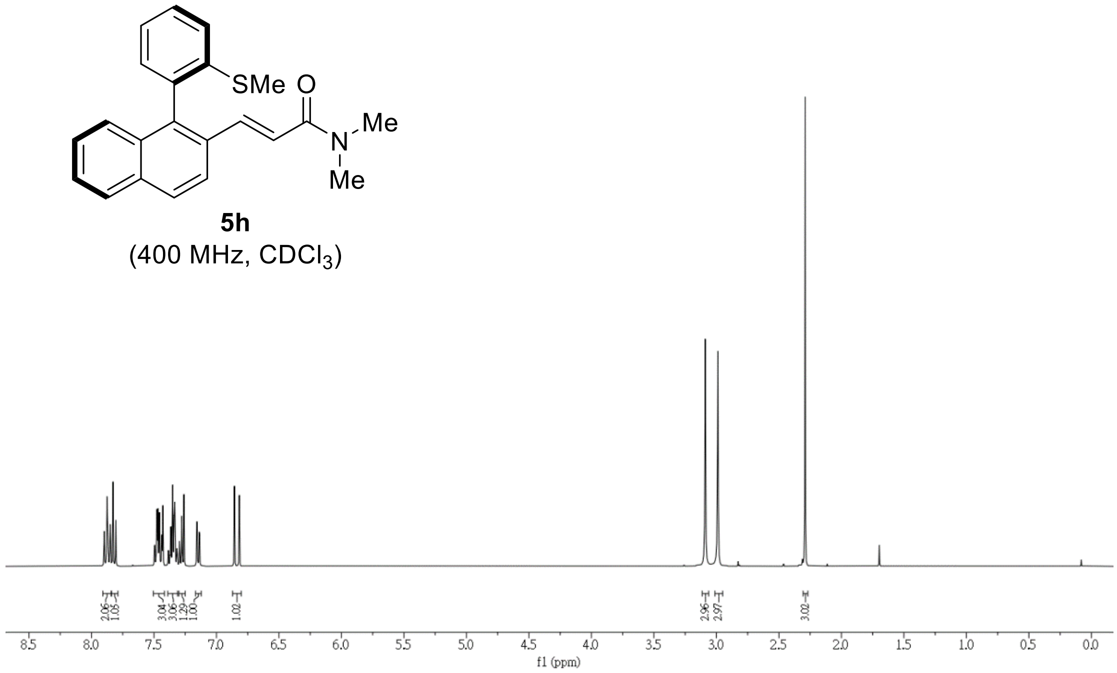

5h
(400 MHz, $CDCl_3$)

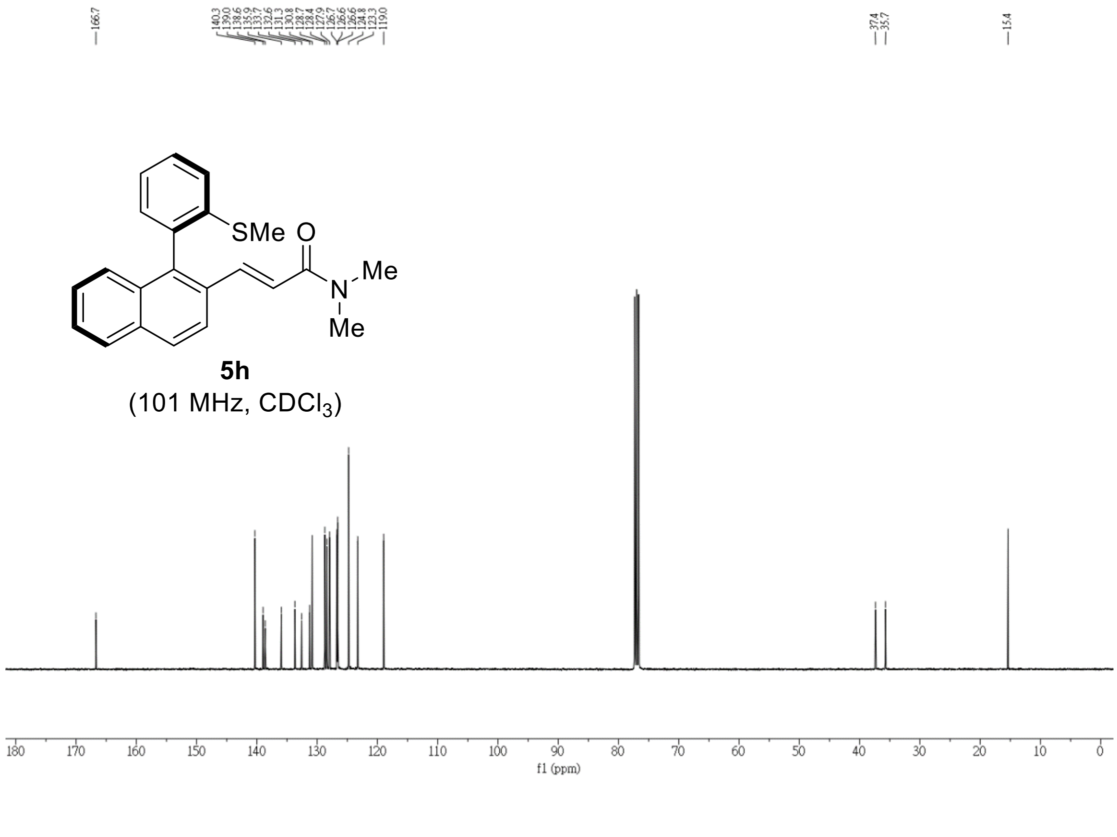

5h
(101 MHz, $CDCl_3$)

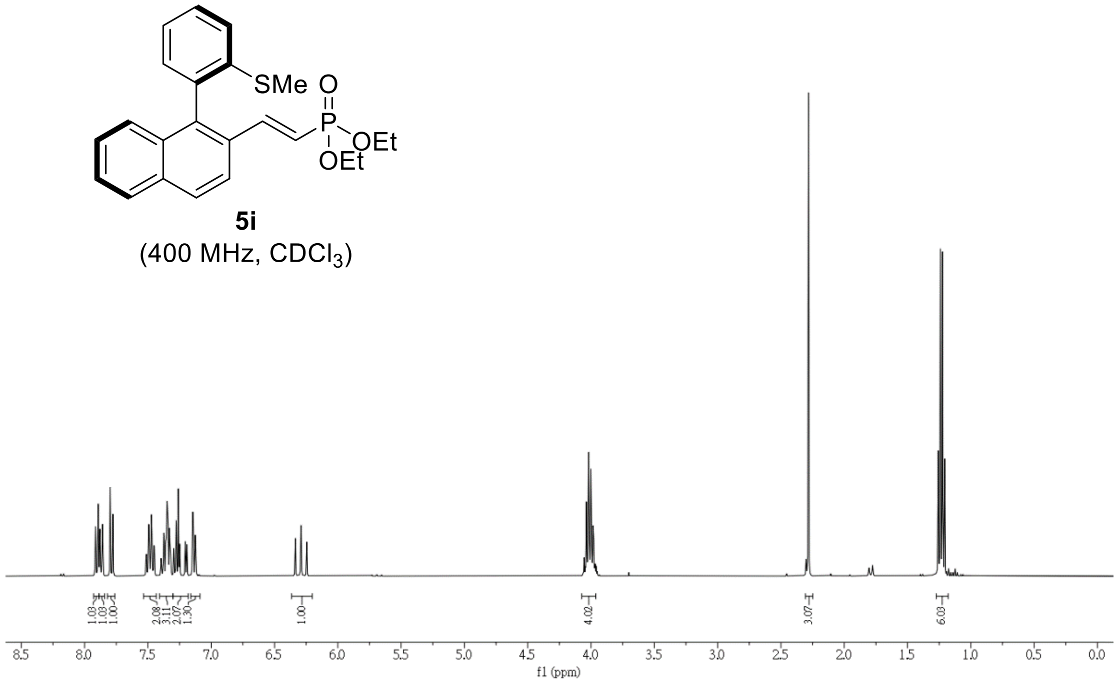
SMe
O
P
OEt
OEt
5i
(400 MHz, CDCl3)
1.03
1.03
1.00
2.08
3.11
2.07
1.30
1.00
4.02
3.07
6.03
f1 (ppm)

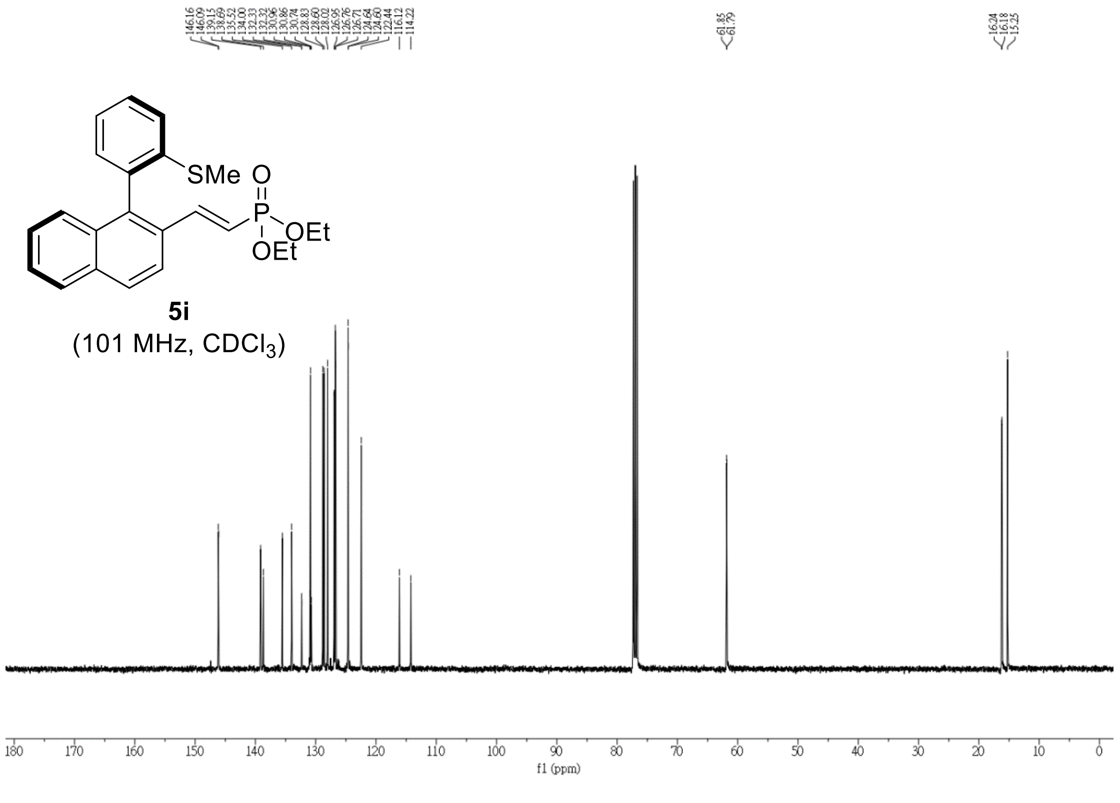
146.16
146.09
139.15
138.69
135.52
134.00
132.33
132.32
130.96
130.86
130.74
128.83
128.60
128.02
126.95
126.76
126.71
124.64
124.60
122.44
116.12
114.22
61.85
61.79
16.24
16.18
15.25
SMe
O
P
OEt
OEt
5i
(101 MHz, CDCl3)
f1 (ppm)

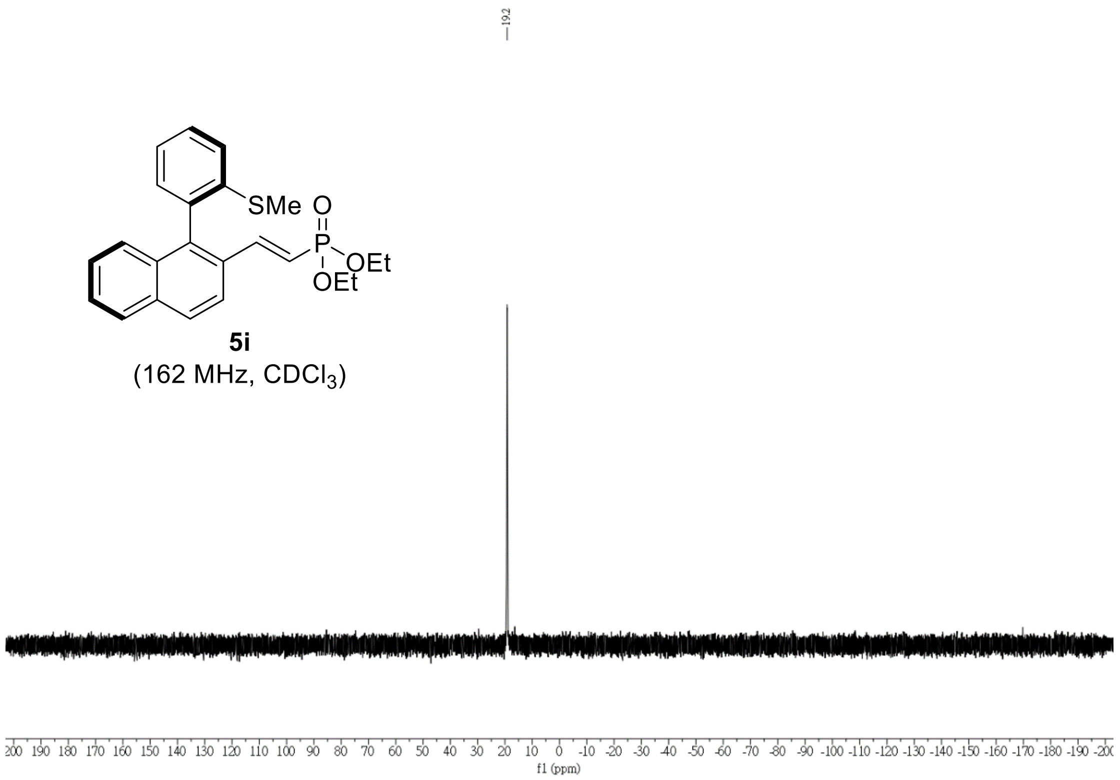
19.2
SMe
O
P
OEt
OEt
5i
(162 MHz, CDCl3)
200 190 180 170 160 150 140 130 120 110 100 90 80 70 60 50 40 30 20 10 0 -10 -20 -30 -40 -50 -60 -70 -80 -90 -100 -110 -120 -130 -140 -150 -160 -170 -180 -190 -200
f1 (ppm)

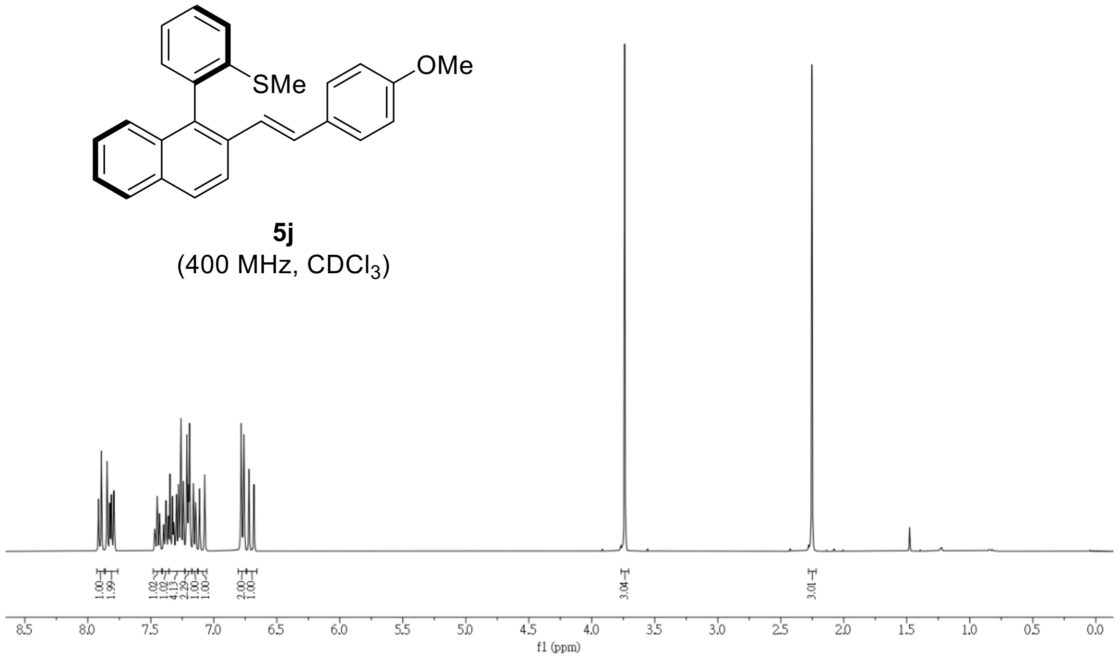
SMe
OMe
5j
(400 MHz, CDCl3)
f1 (ppm)

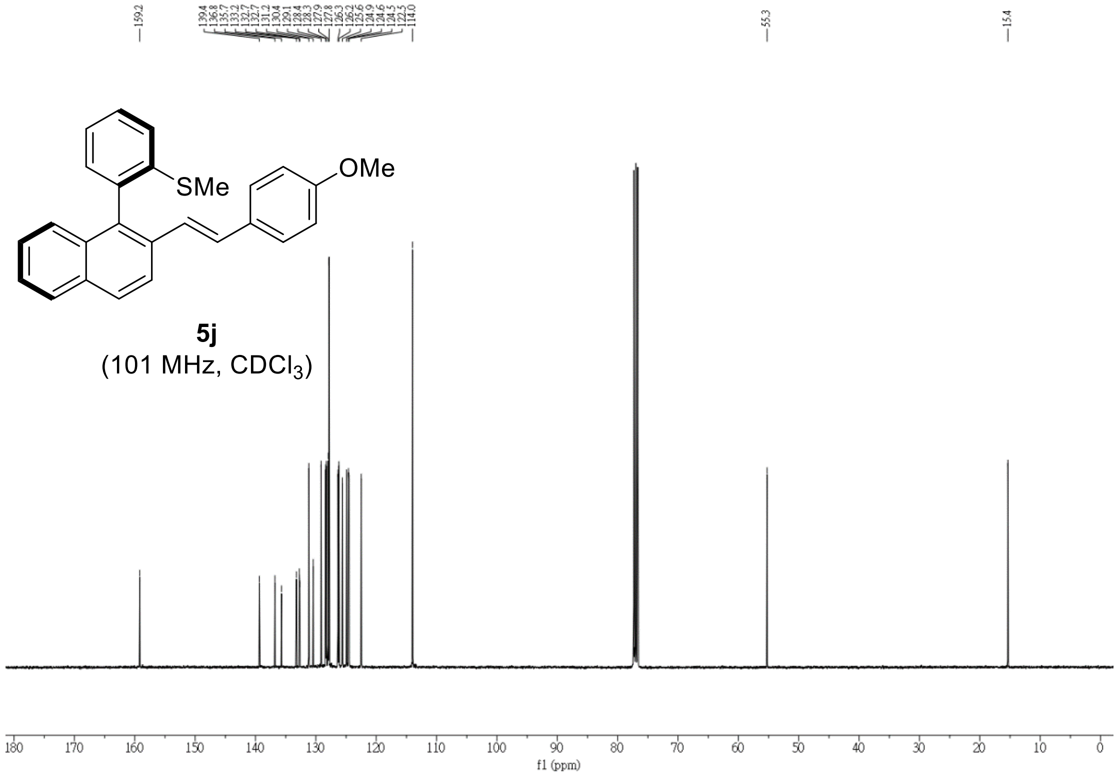
SMe
OMe
5j
(101 MHz, CDCl3)
f1 (ppm)

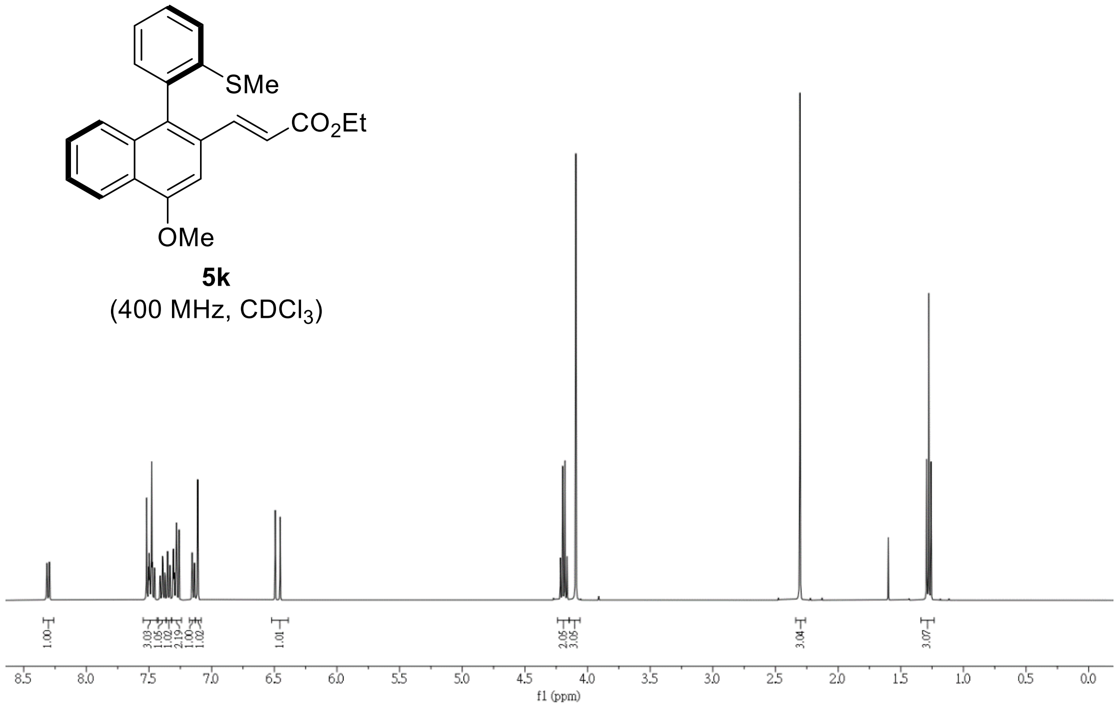
SMe
CO2Et
OMe
5k
(400 MHz, CDCl3)
1.00
3.03
1.06
1.02
2.19
1.00
1.02
1.01
2.08
3.08
3.04
3.07
8.5 8.0 7.5 7.0 6.5 6.0 5.5 5.0 4.5 4.0 3.5 3.0 2.5 2.0 1.5 1.0 0.5 0.0
f1 (ppm)

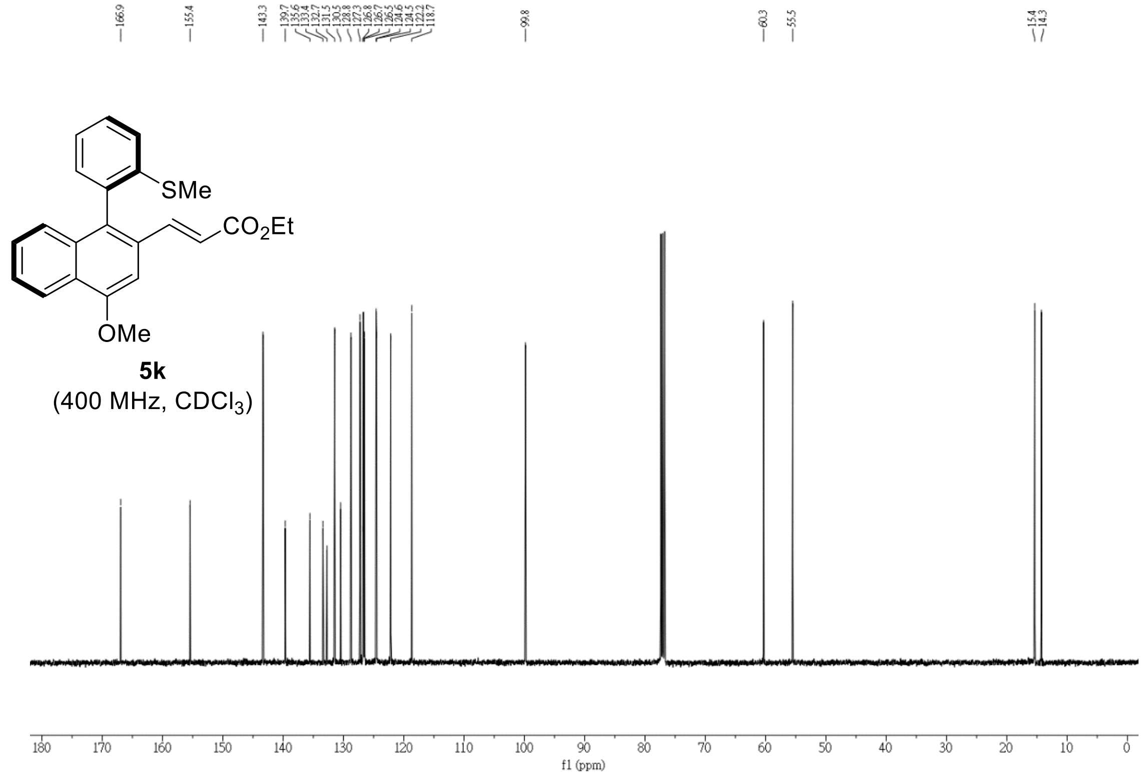
166.9
155.4
143.3
139.7
135.6
133.4
132.7
131.5
130.5
128.8
127.3
126.8
126.7
126.5
124.6
124.5
122.2
118.7
99.8
60.3
55.5
15.4
14.3
SMe
CO2Et
OMe
5k
(400 MHz, CDCl3)
180 170 160 150 140 130 120 110 100 90 80 70 60 50 40 30 20 10 0
f1 (ppm)

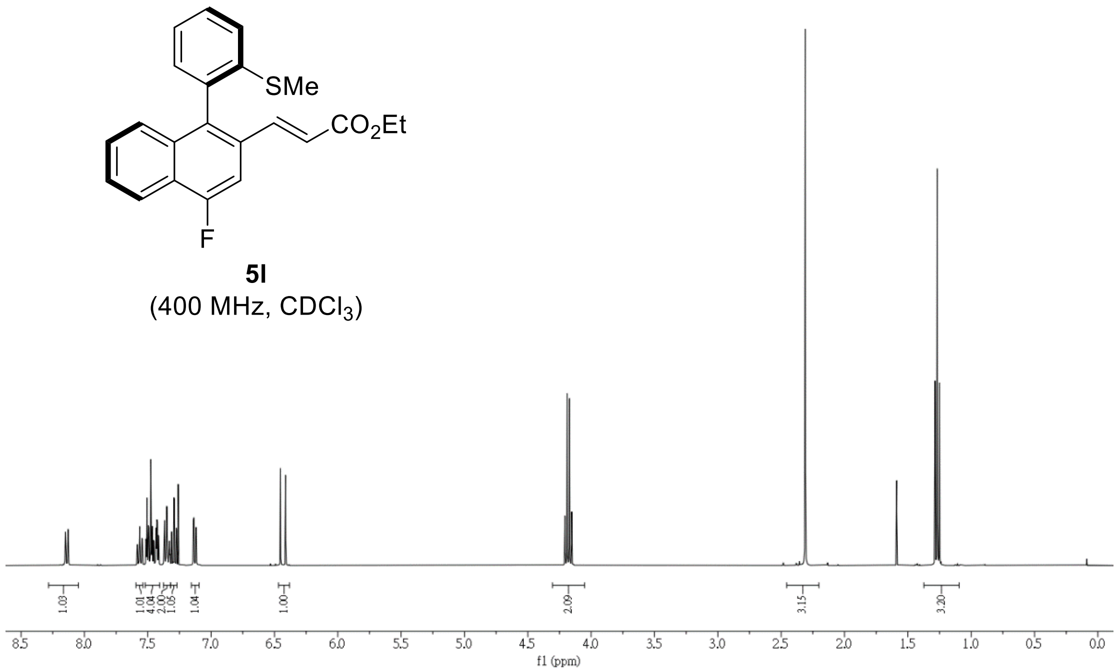
SMe
CO2Et
F
5l
(400 MHz, CDCl3)
f1 (ppm)

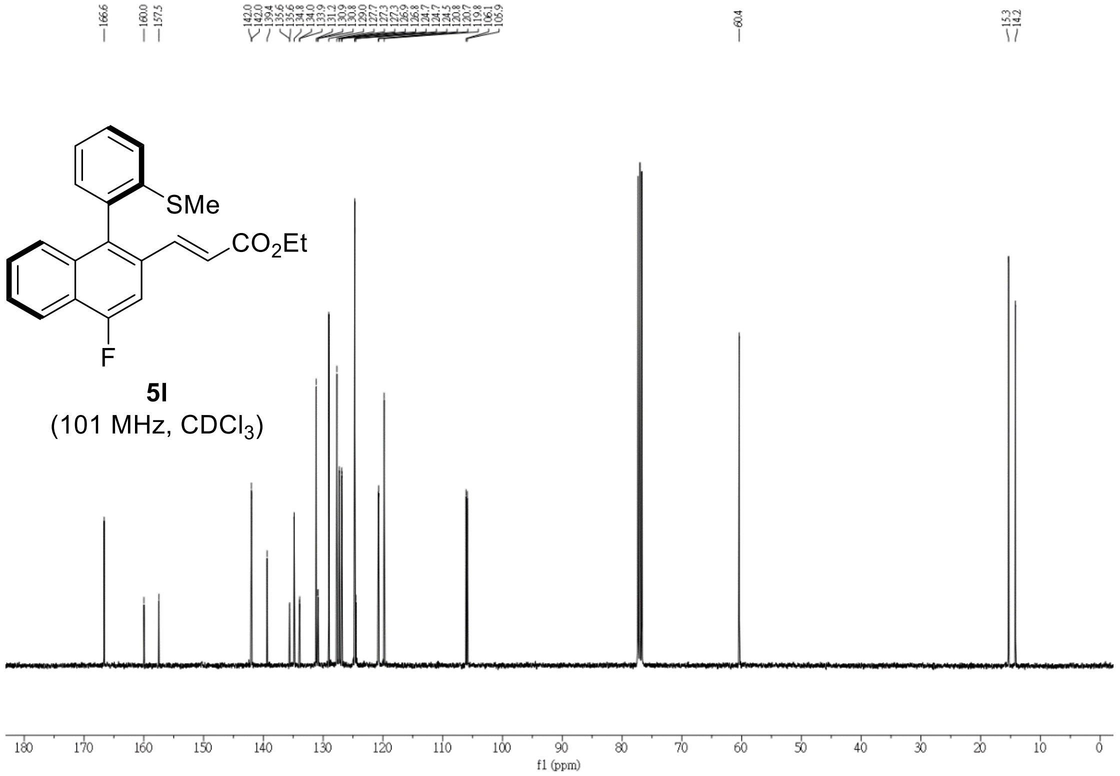
SMe
CO2Et
F
5l
(101 MHz, CDCl3)
f1 (ppm)

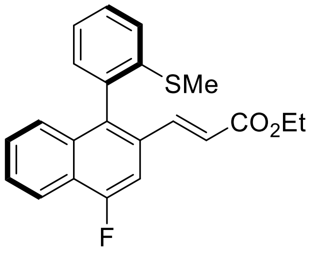

5l

(377 MHz, $CDCl_3$)

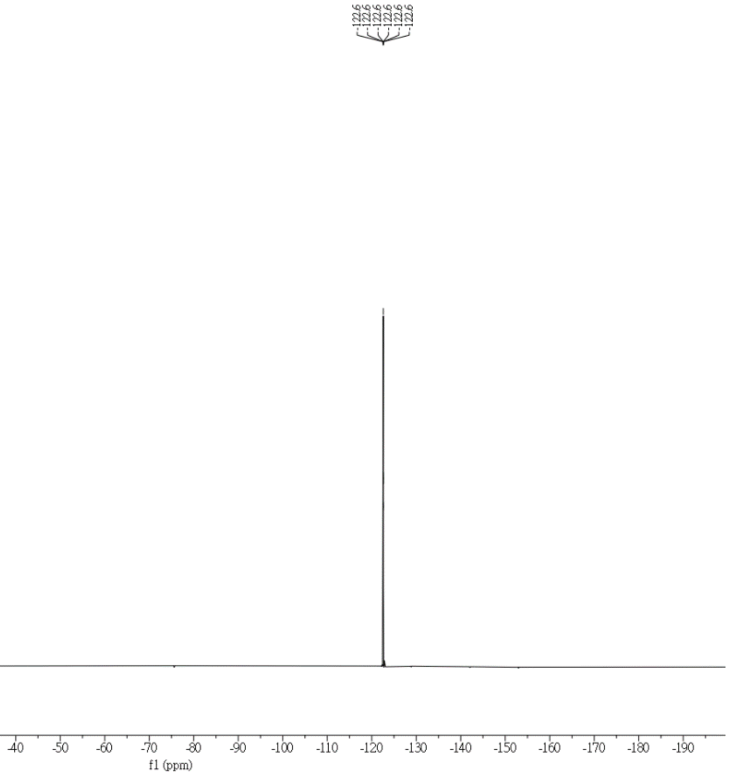

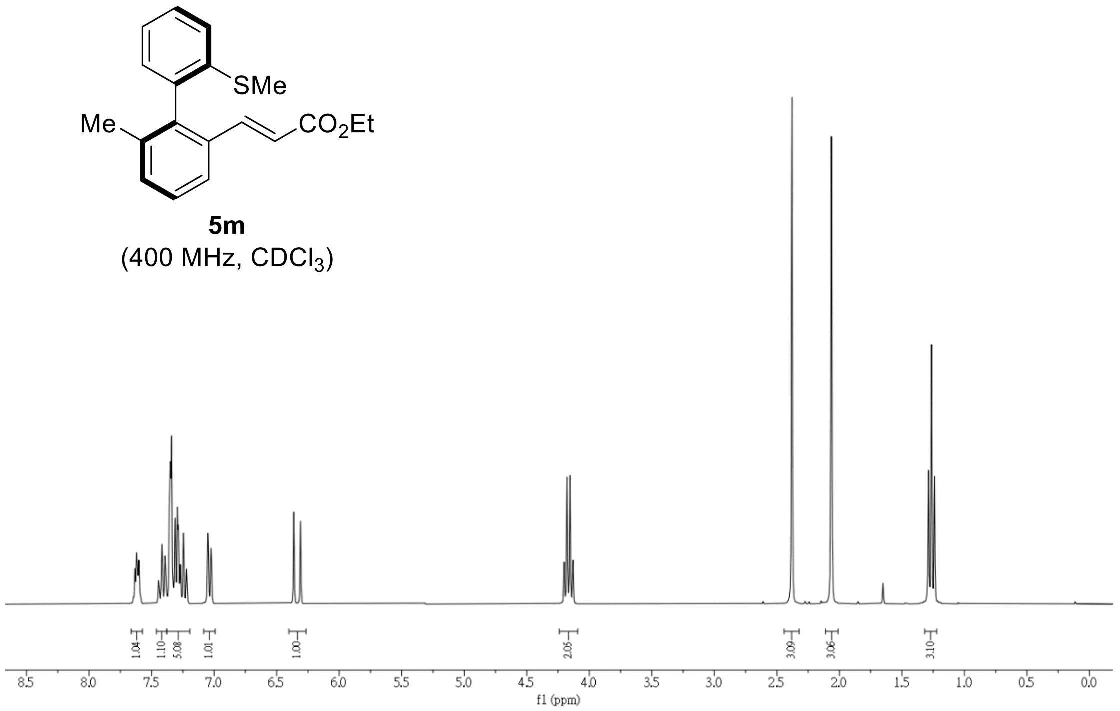
SMe
Me
CO2Et
5m
(400 MHz, CDCl3)
f1 (ppm)

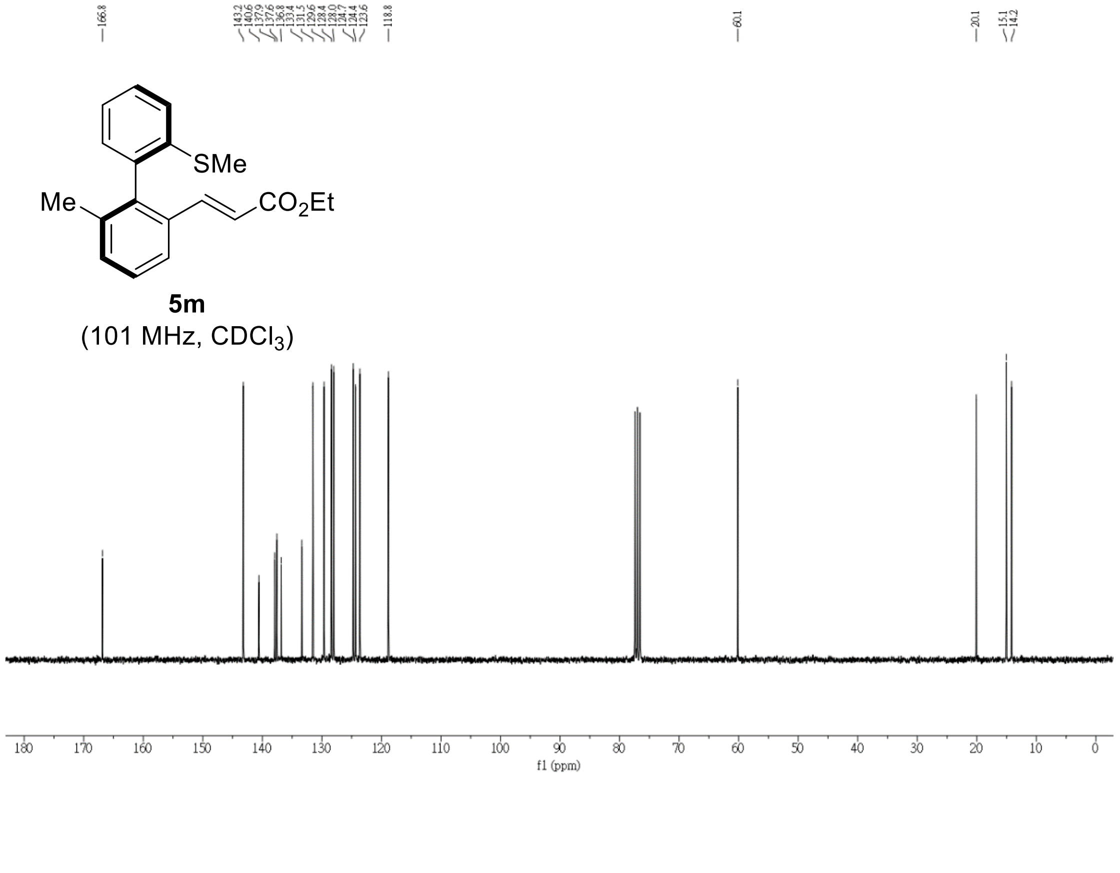
SMe
Me
CO2Et
5m
(101 MHz, CDCl3)
f1 (ppm)

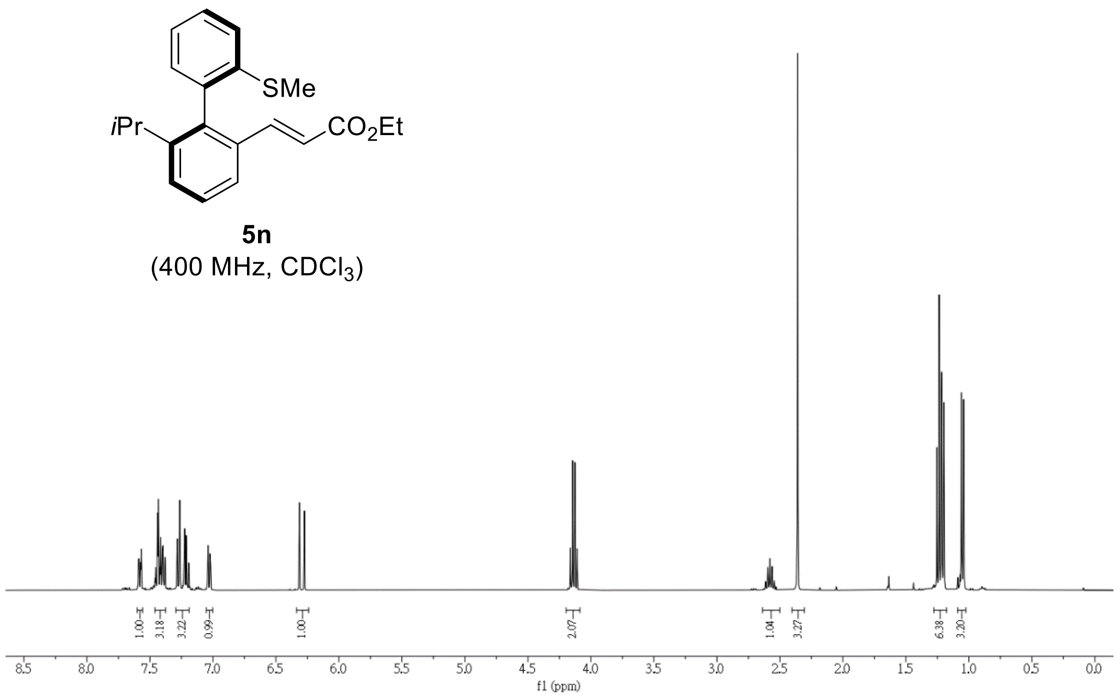

5n
(400 MHz, $CDCl_3$)

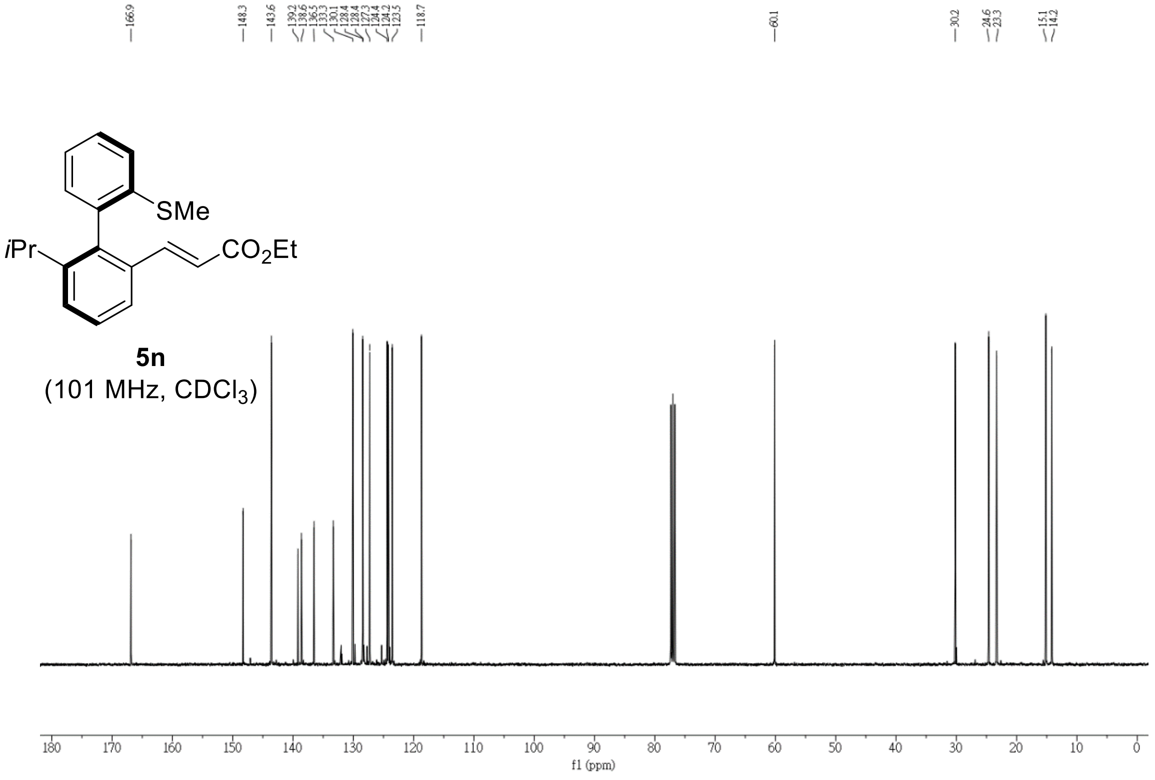

5n
(101 MHz, $CDCl_3$)

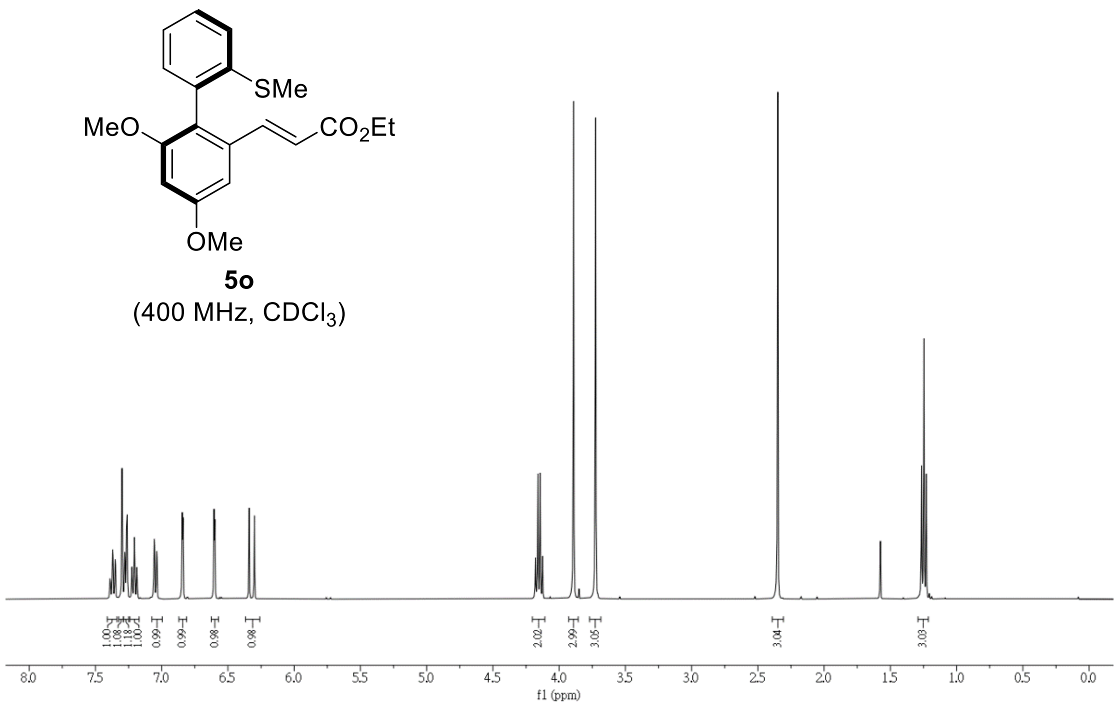
SMe
MeO
CO2Et
OMe
5o
(400 MHz, CDCl3)
f1 (ppm)

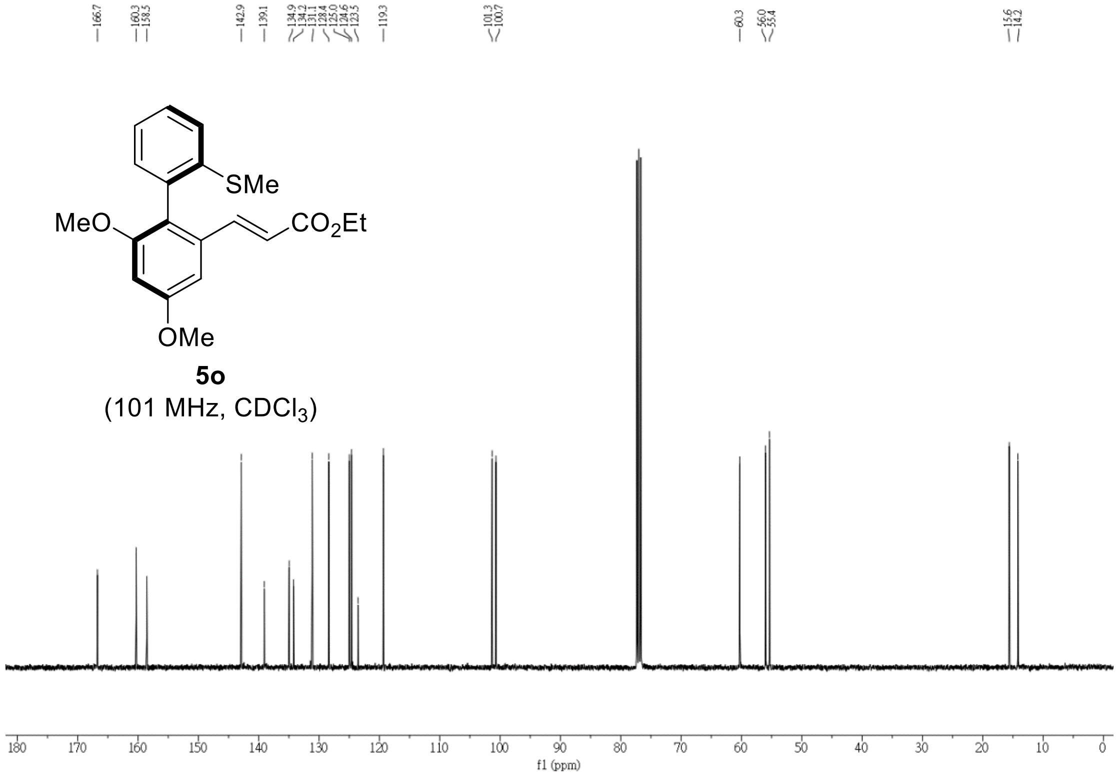
166.7
160.3
158.5
142.9
139.1
134.9
134.2
131.1
128.4
125.0
124.6
123.5
119.3
101.3
100.7
60.3
56.0
55.4
15.6
14.2
SMe
MeO
CO2Et
OMe
5o
(101 MHz, CDCl3)
f1 (ppm)

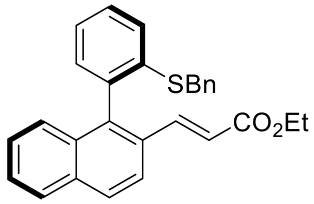

5p
(400 MHz, $CDCl_3$)

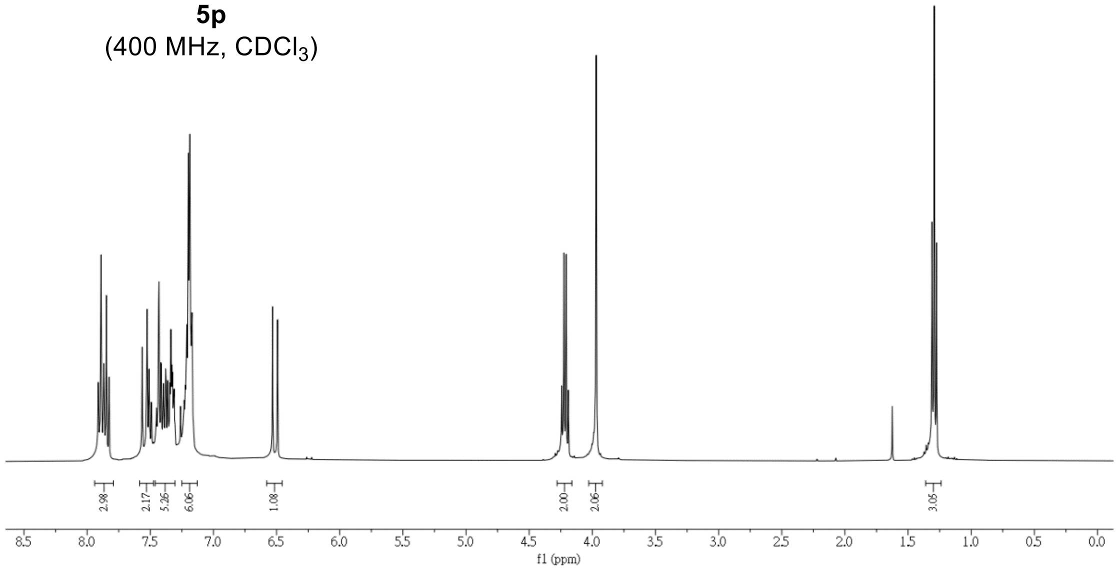

5p
(101 MHz, $CDCl_3$)

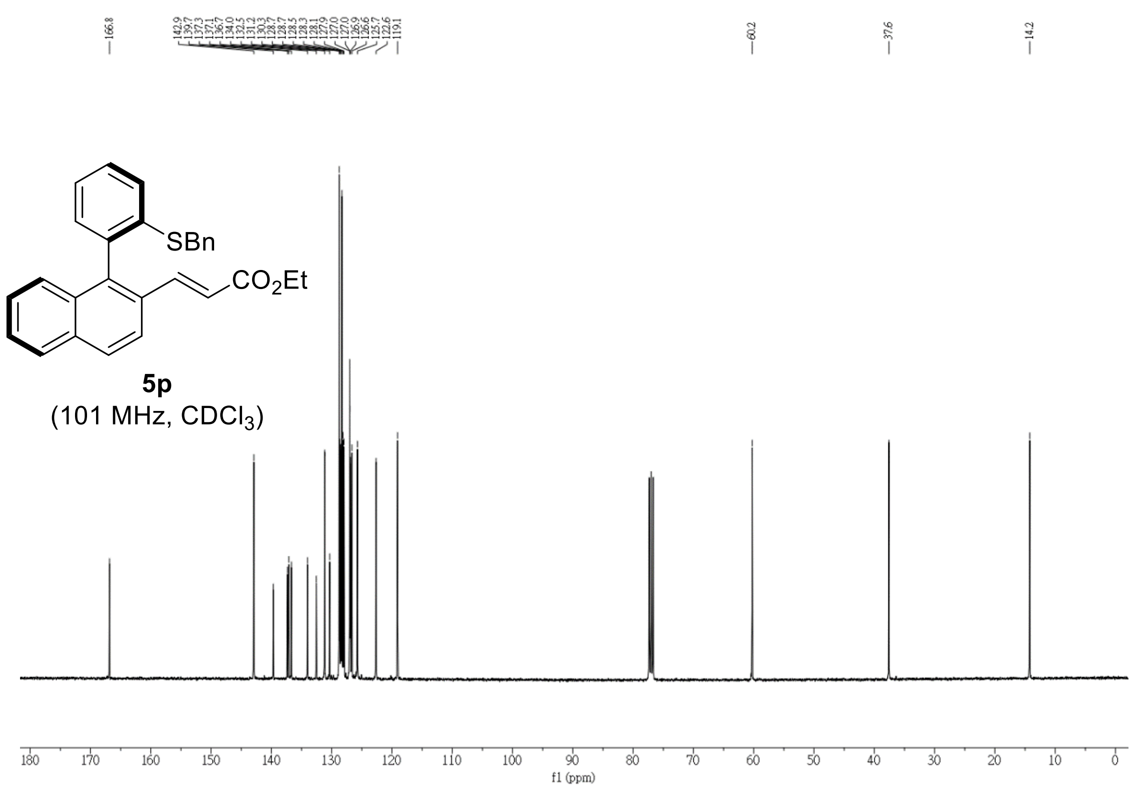

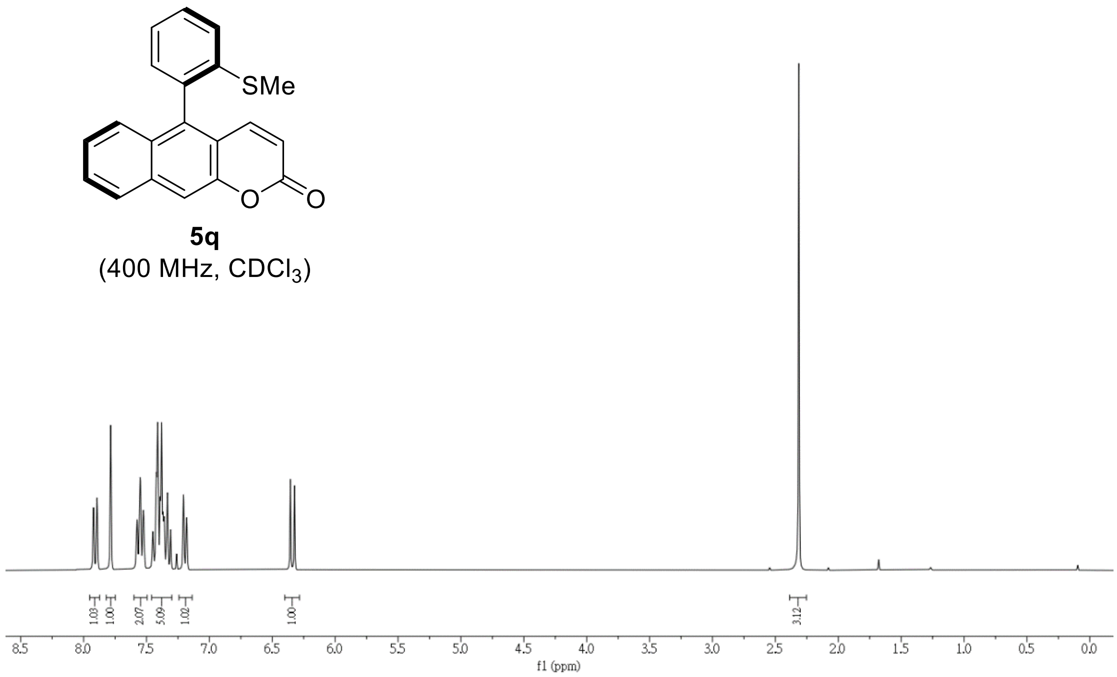
SMe
O
O
5q
(400 MHz, CDCl$_3$)
1.03
1.00
2.07
5.09
1.02
1.00
3.12
f1 (ppm)

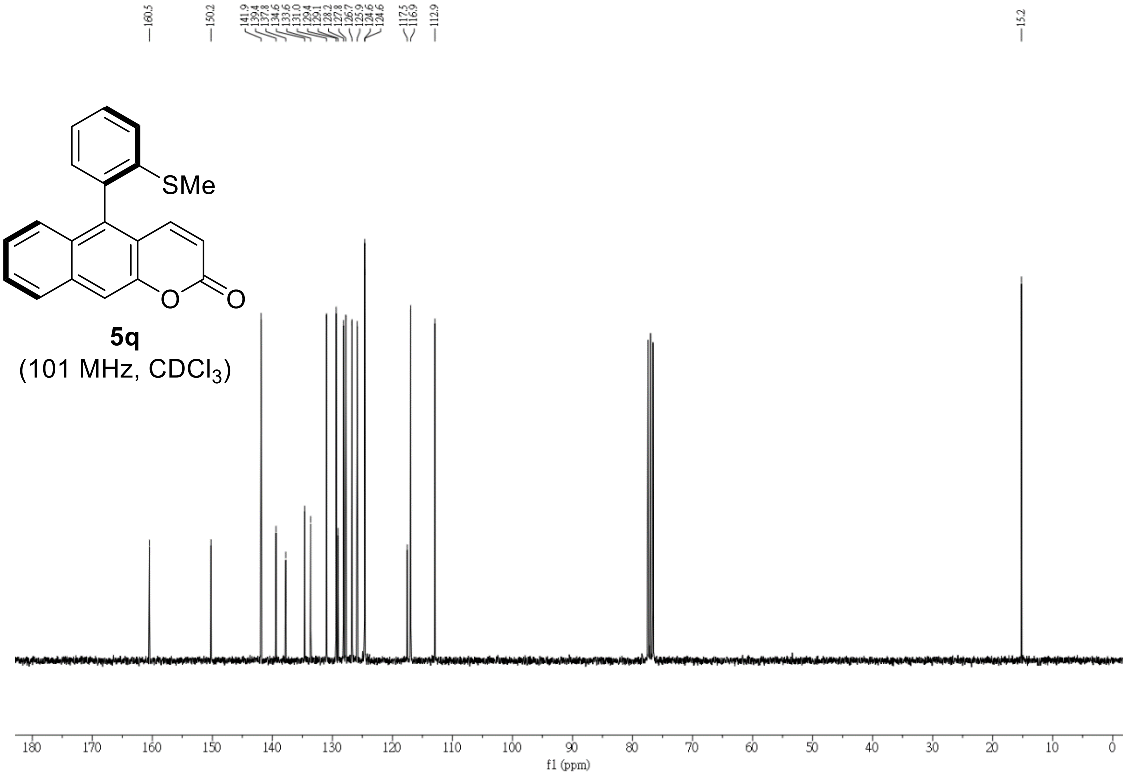
160.5
150.2
141.9
139.4
137.8
134.6
133.6
131.0
129.4
129.1
128.2
127.8
126.7
125.9
124.6
124.6
117.5
116.9
112.9
15.2
SMe
O
O
5q
(101 MHz, CDCl$_3$)
f1 (ppm)

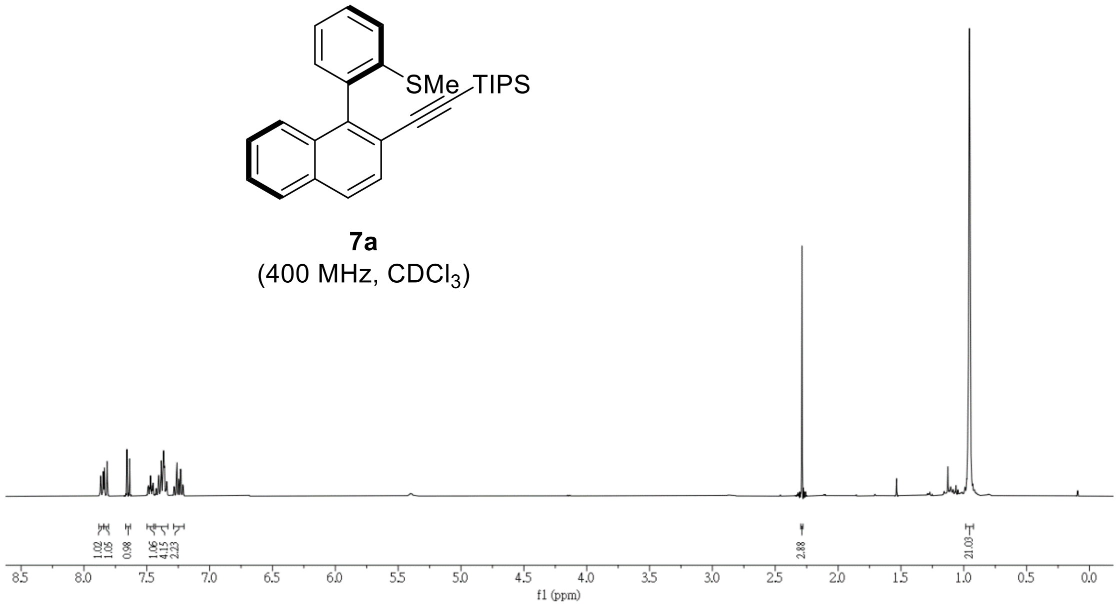
SMe
TIPS
7a
(400 MHz, CDCl3)
1.02
1.05
0.98
1.06
4.15
2.23
2.88
21.03
f1 (ppm)

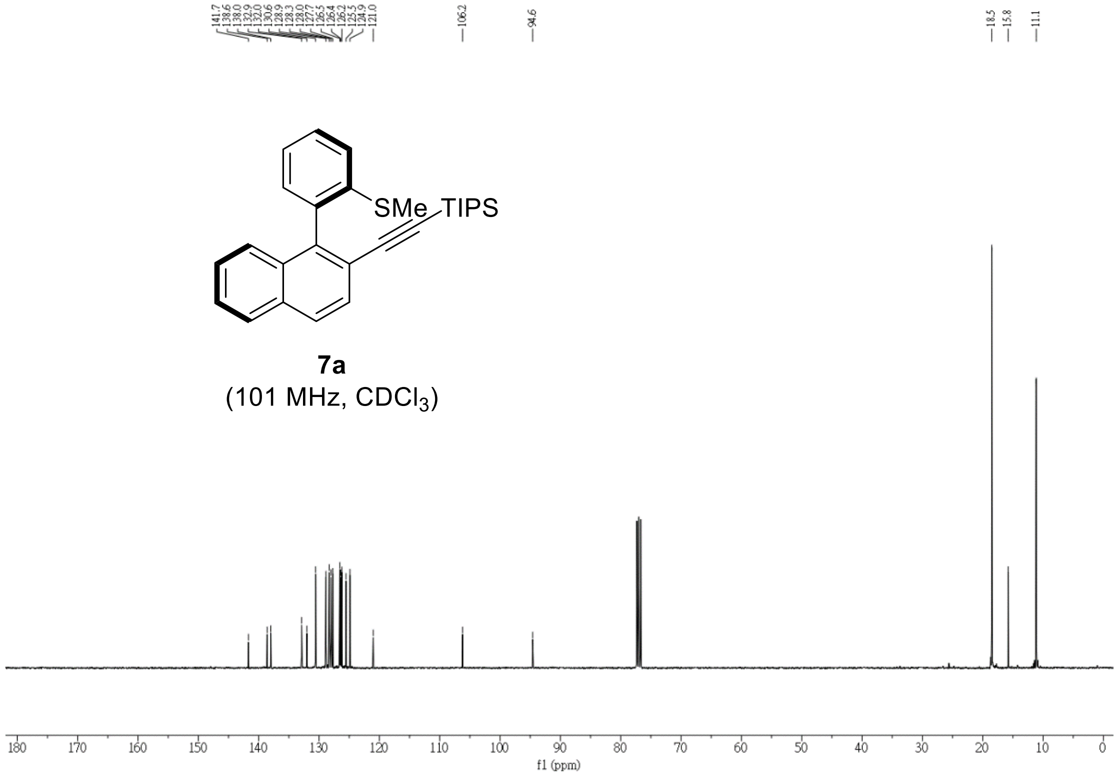
141.7
138.6
138.0
132.9
132.0
130.6
128.9
128.3
128.0
127.7
126.5
126.4
126.2
125.5
124.9
121.0
106.2
94.6
18.5
15.8
11.1
SMe
TIPS
7a
(101 MHz, CDCl3)
f1 (ppm)

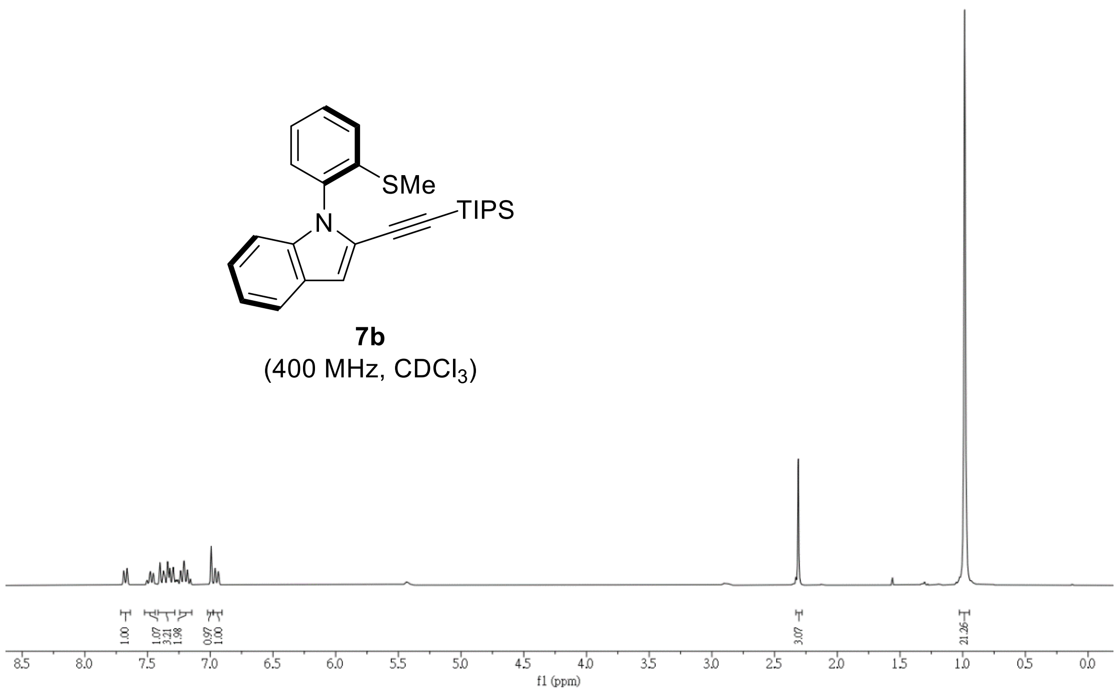
SMe
N
TIPS
7b
(400 MHz, $CDCl_3$)
1.00
1.07
3.21
1.98
0.97
1.00
3.07
21.26
8.5 8.0 7.5 7.0 6.5 6.0 5.5 5.0 4.5 4.0 3.5 3.0 2.5 2.0 1.5 1.0 0.5 0.0
f1 (ppm)

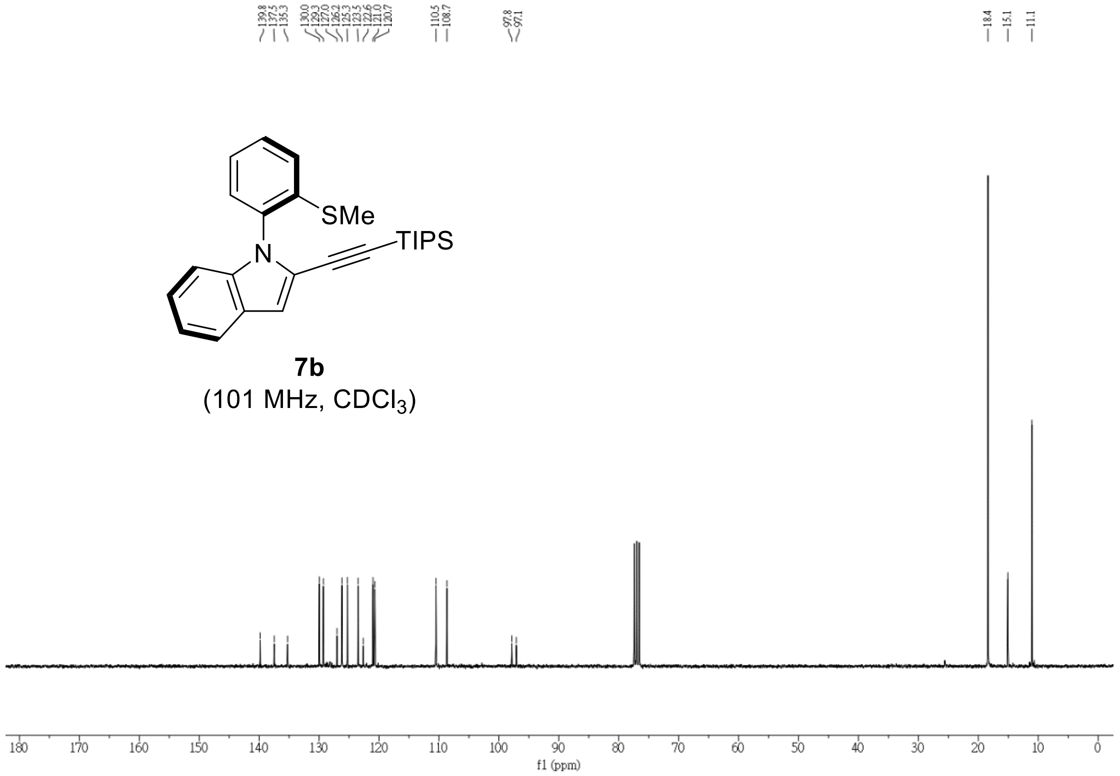
139.8
137.5
135.3
130.0
129.3
127.0
126.2
125.3
123.5
122.6
121.0
120.7
110.5
108.7
97.8
97.1
18.4
15.1
11.1
SMe
N
TIPS
7b
(101 MHz, $CDCl_3$)
180 170 160 150 140 130 120 110 100 90 80 70 60 50 40 30 20 10 0
f1 (ppm)
